# Diretriz Brasileira de Ergometria em Crianças e Adolescentes – 2024

**DOI:** 10.36660/abc.20240525

**Published:** 2024-09-03

**Authors:** Tales de Carvalho, Odilon Gariglio Alvarenga de Freitas, William Azem Chalela, Carlos Alberto Cordeiro Hossri, Mauricio Milani, Susimeire Buglia, Andréa Maria Gomes Marinho Falcão, Ricardo Vivacqua Cardoso Costa, Luiz Eduardo Fonteles Ritt, Maria Eulália Thebit Pfeiffer, Odwaldo Barbosa e Silva, Rodrigo Imada, José Luiz Barros Pena, Antônio Carlos Avanza, Carlos Alberto Cyrillo Sellera

**Affiliations:** 1 Clínica de Prevenção e Reabilitação Cardiosport Florianópolis SC Brasil Clínica de Prevenção e Reabilitação Cardiosport, Florianópolis, SC – Brasil; 2 Universidade do Estado de Santa Catarina Florianópolis SC Brasil Universidade do Estado de Santa Catarina, Florianópolis, SC – Brasil; 3 Minascor Centro Médico Belo Horizonte MG Brasil Minascor Centro Médico, Belo Horizonte, MG – Brasil; 4 Hospital das Clínicas da Faculdade de Medicina da Universidade de São Paulo Instituto do Coração do Hospital São Paulo SP Brasil Instituto do Coração do Hospital das Clínicas da Faculdade de Medicina da Universidade de São Paulo (InCor-HCFMUSP), São Paulo, SP – Brasil; 5 Sociedade Beneficente de Senhoras do Hospital Sírio-Libanês São Paulo SP Brasil Sociedade Beneficente de Senhoras do Hospital Sírio-Libanês, São Paulo, SP – Brasil; 6 Hospital do Coração São Paulo SP Brasil Hospital do Coração (HCOr), São Paulo, SP – Brasil; 7 Instituto Dante Pazzanese de Cardiologia São Paulo SP Brasil Instituto Dante Pazzanese de Cardiologia, São Paulo, SP – Brasil; 8 Universidade de Brasília Brasília DF Brasil Universidade de Brasília (UnB), Brasília, DF – Brasil; 9 Hasselt University Hasselt Bélgica Hasselt University, Hasselt – Bélgica; 10 Jessa Ziekenhuis Hasselt Bélgica Jessa Ziekenhuis, Hasselt – Bélgica; 11 Hospital Pró-Cardíaco Rio de Janeiro RJ Brasil Hospital Pró-Cardíaco, Rio de Janeiro, RJ – Brasil; 12 Escola Bahiana de Medicina e Saúde Pública Salvador BA Brasil Escola Bahiana de Medicina e Saúde Pública, Salvador, BA – Brasil; 13 Instituto D’Or de Pesquisa e Ensino Salvador BA Brasil Instituto D’Or de Pesquisa e Ensino, Salvador, BA – Brasil; 14 Hospital Cárdio Pulmonar Salvador BA Brasil Hospital Cárdio Pulmonar, Salvador, BA – Brasil; 15 Instituto Estadual de Cardiologia Aloysio de Castro Rio de Janeiro RJ Brasil Instituto Estadual de Cardiologia Aloysio de Castro, Rio de Janeiro, RJ – Brasil; 16 Faculdade de Ciências Médicas de São José dos Campos São José dos Campos SP Brasil Faculdade de Ciências Médicas de São José dos Campos, São José dos Campos, SP – Brasil; 17 Hospital Sírio-Libanês São Paulo SP Brasil Hospital Sírio-Libanês, São Paulo, SP – Brasil; 18 Faculdade Ciências Médicas de Minas Gerais Belo Horizonte MG Brasil Faculdade Ciências Médicas de Minas Gerais, Belo Horizonte, MG – Brasil; 19 Hospital Felício Rocho Belo Horizonte MG Brasil Hospital Felício Rocho, Belo Horizonte, MG – Brasil; 20 Universidade Vila Velha Vila Velha ES Brasil Universidade Vila Velha, Vila Velha, ES – Brasil; 21 Universidade Metropolitana de Santos Santos SP Brasil Universidade Metropolitana de Santos (UNIMES), Santos, SP – Brasil

## Abstract

**Classes de Recomendação:**

**Classe I:** Condições para as quais há evidências conclusivas e, na sua falta, consenso geral de que o procedimento é seguro e útil/eficaz.
**Classe II:** Condições para as quais há evidências conflitantes e/ou divergência de opinião sobre a segurança e utilidade/eficácia do procedimento.
**Classe IIa:** Peso ou evidência/opinião a favor do procedimento. A maioria aprova.
**Classe IIb:** Segurança e utilidade/eficácia menos estabelecidas, havendo opiniões divergentes.
**Classe III:** Condições para as quais há evidências e/ou consenso de que o procedimento não é útil/eficaz e, em alguns casos, pode ser prejudicial.

**Níveis de Evidência:**

**Nível A:** Dados obtidos a partir de múltiplos estudos randomizados de bom porte, concordantes e/ou de metanálise robusta de estudos randomizados.
**Nível B:** Dados obtidos a partir de metanálise menos robusta, a partir de um único estudo randomizado e/ou de estudos observacionais.
**Nível C:** Dados obtidos de opiniões consensuais de especialistas.

**Table:** 

Diretriz Brasileira de Ergometria em Crianças e Adolescentes – 2024	
O relatório abaixo lista as declarações de interesse conforme relatadas à SBC pelos especialistas durante o período de desenvolvimento deste posicionamento, 2023-2024.	
Especialista	Tipo de relacionamento com a indústria
Andréa Maria Gomes Marinho Falcão	Nada a ser declarado
Antonio Carlos Avanza Júnior	Nada a ser declarado
Carlos Alberto Cordeiro Hossri	Nada a ser declarado
Carlos Alberto Cyrillo Sellera	Nada a ser declarado
José Luiz Barros Pena	Nada a ser declarado
Luiz Eduardo Fonteles Ritt	Declaração financeira A - Pagamento de qualquer espécie e desde que economicamente apreciáveis, feitos a (i) você, (ii) ao seu cônjuge/ companheiro ou a qualquer outro membro que resida com você, (iii) a qualquer pessoa jurídica em que qualquer destes seja controlador, sócio, acionista ou participante, de forma direta ou indireta, recebimento por palestras, aulas, atuação como proctor de treinamentos, remunerações, honorários pagos por participações em conselhos consultivos, de investigadores, ou outros comitês, etc. Provenientes da indústria farmacêutica, de órteses, próteses, equipamentos e implantes, brasileiras ou estrangeiras: - Boehringer Lilly: Jardiance; Novonordis: pesquisador em estudos; AstraZeneca; Novartis; Bayer; Bristol; Pfizer. B - Financiamento de pesquisas sob sua responsabilidade direta/pessoal (direcionado ao departamento ou instituição) provenientes da indústria farmacêutica, de órteses, próteses, equipamentos e implantes, brasileiras ou estrangeiras: - MDI Medical. Outros relacionamentos Financiamento de atividades de educação médica continuada, incluindo viagens, hospedagens e inscrições para congressos e cursos, provenientes da indústria farmacêutica, de órteses, próteses, equipamentos e implantes, brasileiras ou estrangeiras: - Novo Nordisk: Ozempic
Maria Eulália Thebit Pfeiffer	Nada a ser declarado
Mauricio Milani	Nada a ser declarado
Odilon Gariglio Alvarenga de Freitas	Nada a ser declarado
Odwaldo Barbosa e Silva	Nada a ser declarado
Ricardo Vivacqua Cardoso Costa	Nada a ser declarado
Rodrigo Imada	Nada a ser declarado
Susimeire Buglia	Nada a ser declarado
Tales de Carvalho	Nada a ser declarado
William Azem Chalela	Nada a ser declarado

## Parte 1 – Indicações, Aspectos Legais e Formação em Ergometria

### 1. Introdução

O Teste Ergométrico ou Teste de Exercício (TE) é um exame médico complementar, no qual o indivíduo é submetido a um esforço físico programado e individualizado, com a finalidade de avaliar as respostas clínica, hemodinâmica, autonômica, eletrocardiográfica, metabólica indireta e eventualmente enzimática. Recebe a denominação de Teste Cardiopulmonar de Exercício (TCPE) quando são adicionados ao TE parâmetros ventilatórios e a análise dos gases no ar expirado. A denominação Ergometria contempla o TE e o TCPE.^1^

Em crianças e adolescentes, o TE e o TCPE são métodos de avaliação diagnóstica, prognóstica e do desempenho cardiorrespiratório em diversas situações clínicas. São procedimentos considerados seguros e de custo-efetividade comprovada na população pediátrica.^1^

No Brasil, as anomalias congênitas e as doenças cardiovasculares (DCV) representam nas crianças, respectivamente, a segunda e a nona causa de morte. Nos adolescentes, as DCV são a terceira maior causa de morte, e as anomalias congênitas são a oitava causa. Esses dados demonstram a importância do cuidado da saúde cardiovascular (CV) na população pediátrica.^2^

O Ministério da Saúde do Brasil segue como definição de adolescência a prescrita pela Organização Mundial da Saúde (OMS), que caracteriza o período de vida entre 10 e 19 anos. Entretanto, a legislação brasileira considera como adolescente a pessoa que estiver na faixa etária entre 12 e 18 anos. Publicações científicas também adotam outras faixas etárias para crianças (1 a 13 anos) e adolescentes (13 a 18 anos).^3 – 5^

Esta diretriz buscou consolidar as informações mais recentes das publicações científicas sobre TE/TCPE em crianças e adolescentes quanto às indicações, contraindicações, riscos, aspectos metodológicos, respostas hemodinâmicas e eletrocardiográficas, critérios diagnósticos e particularidades dos exames em doenças específicas na população pediátrica. Adicionalmente, foram abordadas as variáveis ventilatórias e metabólicas provindas da análise dos gases expirados no TCPE e, também, TE e TCPE associados a métodos de imagem.

Ao longo do documento, enfatizamos as particularidades dos exames considerando a faixa etária do paciente, sexo, composição corporal, níveis de aptidão física e estados de saúde CV e pulmonar basais.^6 – 12^

A presente diretriz tem o potencial de se tornar forte referência e relevante fonte de consultas para os cardiologistas, visando à difusão do TE e do TCPE na perspectiva de aprimorar a saúde CV de crianças e adolescentes.

### 2. Indicações e Contraindicações do TE e do TCPE em Crianças e Adolescentes

#### 2.1. Indicações Gerais do TE

O TE na população pediátrica é uma ferramenta que contribui para o diagnóstico e avaliação da repercussão de DCV (congênitas, genéticas e adquiridas), estratificação de risco, determinação de prognóstico, ajustes terapêuticos e liberação/prescrição de exercícios, inclusive na reabilitação cardiovascular (RCV).

As indicações e objetivos gerais do TE na população pediátrica são:^6 – 12^

1. Avaliar sintomas relacionados ao esforço.
2. Avaliar o comportamento hemodinâmico [pressão arterial (PA), frequência cardíaca (FC), duplo-produto (DP), resistência arterial periférica etc.].
3. Identificar respostas anormais ao esforço em crianças e adolescentes com DCV congênitas e adquiridas (valvopatias, cardiomiopatias etc.), pulmonares ou de outros órgãos.
4. Detectar isquemia miocárdica decorrente de anomalias coronárias congênitas, ateromatose (muito rara) ou no contexto da doença de Kawasaki.
5. Reconhecer as arritmias cardíacas quanto ao tipo, densidade e complexidade.
6. Avaliar o comportamento da pré-excitação e canalopatias ao esforço.
7. Estabelecer o prognóstico em determinadas DCV, inclusive através de exames seriados.
8. Na indicação e ajustes terapêuticos.
9. Avaliar a condição aeróbica, a tolerância ao esforço e o condicionamento físico.
10. Fornecer subsídios para a liberação e prescrição de exercícios físicos, incluindo RCV e atividades esportivas.

#### 2.2. Indicações do TE em Situações Clínicas Específicas

Apresentaremos as situações clínicas específicas nas quais o TE tem sua efetividade estudada e testada, permitindo determinar o grau de indicação e nível de recomendação estabelecidos na literatura.

##### 2.2.1. Na Suspeita de Isquemia Miocárdica e Doença Arterial Coronariana

Em crianças e adolescentes, a isquemia miocárdica e doença arterial coronariana (DAC) apresentam etiologias diferentes das encontradas nos adultos. Nesse contexto, o TE apresenta reconhecida utilidade na investigação diagnóstica, no seguimento, em decisões terapêuticas e na estratificação de risco ( Tabela 1 ).^6 , 13 , 14^

**Tabela 1:** Indicações do TE na suspeita de isquemia miocárdica e doença arterial coronariana em crianças e adolescentes

Indicação	GR	NE
Na doença de Kawasaki, para investigação de arritmias esforço-induzidas, avaliação funcional, quantificação de repercussão de lesões coronarianas, estratificação de risco e liberação para exercícios.^15 – 18^	I	B
Investigação de queixa de dor torácica anginosa típica.^6 , 13 , 14 , 19 , 20^	I	C
Avaliação de isquemia miocárdica residual e tolerância ao esforço após o tratamento cirúrgico para correção da transposição das grandes artérias.^21 – 23^	I	C
Após cirurgia em artéria coronária (operação de troca arterial, procedimento de Ross, substituição da aorta ascendente, correção da síndrome de Bland-White-Garland), para detecção de isquemia miocárdica e arritmias esforço-induzidas e liberação de exercícios incluindo RCV.^6 , 24 – 27^	IIa	B
Nas anomalias das artérias coronárias para triagem, investigação de isquemia esforço-induzida, indicação de correção cirúrgica e liberação para exercícios físicos.^28 , 29^	IIa	B
Avaliação da capacidade funcional e decisão terapêutica de paciente em que outro método tenha detectado isquemia miocárdica.^6 , 30^	IIb	B
Na ponte miocárdica, para investigação de sintomas e arritmias esforço-induzidas e estratificação de risco.^31 , 32^	IIb	B
Seguimento de pacientes com arterite de Takayasu ou lúpus eritematoso sistêmico, para investigação de doença arterial coronariana secundária.^33 , 34^	IIb	C
Diagnóstico de DAC em pacientes com BRE, WPW, ritmo de MP e terapêutica com digitálicos.	III	B
Investigação de dor torácica tipicamente não anginosa.^13 , 35 , 36^	III	B
Na doença de Kawasaki, TE sem outro método associado, para avaliação de isquemia miocárdica.^37^	III	C

GR: grau de recomendação; NE: nível de evidência; RCV: reabilitação cardiovascular; DAC: doença arterial coronariana; BRE: bloqueio de ramo esquerdo; WPW: síndrome de Wolff-Parkinson-White; MP: marca-passo; TE: teste ergométrico.

##### 2.2.2. Indicações na Hipertensão Arterial Sistêmica

O TE permite a avaliação do comportamento pressórico ao esforço e diagnóstico de hipertensão arterial na população pediátrica, com e sem cardiopatia congênita (CC). O comportamento da pressão arterial (PA) durante o TE tem poder preditivo adicional às medidas esporádicas no consultório ( Tabela 2 ).^38^

**Tabela 2:** Indicações do TE na hipertensão arterial sistêmica em crianças e adolescentes

Indicação	GR	NE
Avaliação do comportamento da PA ao esforço (com ou sem cardiopatia) para diagnóstico de HAS.^41 – 43^	I	C
Avaliação pré-participação de hipertensos para esporte competitivo.^44 , 45^	I	B
Suspeita de hipertensão do avental branco.^41 , 46^	IIa	B
Avaliação da resposta terapêutica e estratificação de risco em hipertensos.^4 , 47 , 48^	IIa	B
Após correção de coarctação da aorta, para avaliação do comportamento pressórico, índice tornozelo-braquial pós-esforço e estratificação de risco de hipertensão. * ^8 , 49 – 51^	IIa	B
Suspeita de hipertensão mascarada em adolescente.^41 , 52 , 53^	IIa	C
Avaliação de pacientes com HAS descompensada.^11^	III	C

GR: grau de recomendação; NE: nível de evidência; PA: pressão arterial; HAS: hipertensão arterial sistêmica.

* Exame associado adicionalmente ao TE em esteira ergométrica com avaliação do índice tornozelo-braquial em repouso e pós-esforço.

A hipertensão na infância está relacionada ao risco elevado de DCV, aterosclerose, hipertrofia ventricular esquerda (HVE) e insuficiência renal no adulto.^39 , 40^

##### 2.2.3. Indicações em Assintomáticos

Estudos realizados nos últimos anos vêm elucidando o papel do TE na avaliação de pacientes pediátricos assintomáticos e em relação à sua utilidade na estratificação de risco e determinação prognóstica ( Tabela 3 ).

**Tabela 3:** Indicações do TE em crianças e adolescentes assintomáticos

Indicação	GR	NE
Triagem e acompanhamento nas hiperlipidemias genéticas e/ou presença de aterosclerose carotídea.^54 – 56^	IIa	B
História familiar de morte súbita inexplicada em indivíduos jovens.^57 , 58^	IIa	B
Assintomáticos com fatores de risco cardiometabólico na avaliação da aptidão cardiorrespiratória. * ^59 – 61^	IIa	C
Assintomáticos, aparentemente saudáveis, em avaliação pré-participação de exercício físico como lazer e/ou esporte recreacional.^62^	III	C

GR: grau de recomendação; NE: nível de evidência.

* Vide estratificação de risco pré-TE (Parte 2, Sessão 2).

##### 2.2.4. Indicações em Atletas

O TE em crianças e adolescentes atletas permite avaliar a aptidão cardiorrespiratória (ACR), o comportamento hemodinâmico e o diagnóstico/repercussão de eventuais DCV ( Tabela 4 ).^8 , 63 , 64^

**Tabela 4:** Indicações do TE em crianças e adolescentes atletas

Indicação	GR	NE
Investigação de sintomas relacionados ao esforço.^8 , 64^	I	A
Doenças e condições de alto risco de morte súbita cardíaca.^64 – 66^	I	C
Diagnóstico e seguimento de asma esforço-induzida.^67 , 68^	IIa	B
Diagnóstico e seguimento de hipertensão arterial sistêmica.^44 , 53 , 69^	IIa	B
Diabetes tipo I para investigação de sintomas, avaliação da aptidão cardiorrespiratória e estratificação de risco.^70 – 72^	IIa	B
Diagnóstico e seguimento de cardiomiopatias.^73 , 74^	IIa	B
Avaliação pré-participação de esporte competitivo em assintomáticos, sem fatores de risco e aparentemente saudáveis.^8^	III	B

GR: grau de recomendação; NE: nível de evidência.

##### 2.2.5. Indicações nas Cardiopatias Congênitas

A prevalência mundial de CC varia entre 2,4 e 13,7 por 1.000 nascidos vivos, sendo que a maioria (85%) atinge a idade adulta.^75 , 76^ Habitualmente, as crianças com CC, mesmo quando reparadas, são menos ativas fisicamente, inclusive por superproteção familiar.^77 , 78^ Até 15 a 20% dos pacientes com CC apresentam algum comprometimento valvar, sendo as valvopatias isoladas aórtica (bicúspide, estenótica) e pulmonar as mais comuns.^79 – 81^

O TE é recomendado para avaliação clínica, determinação da ACR, decisões terapêuticas, seguimento, estratificação de risco/prognóstico e liberação/prescrição de programas de exercícios, inclusive na RCV ( Tabela 5 ).^7 , 9 , 82 – 85^

**Tabela 5:** Indicações do TE nas cardiopatias congênitas em crianças e adolescentes

Indicação	GR	NE
Avaliação da aptidão cardiorrespiratória e estratificação de risco/prognóstico na CC acianótica, pré e pós-operatório de cirurgia corretiva.^7 , 10 , 86 , 87^	IIa	B
Avaliação da aptidão cardiorrespiratória e estratificação de risco/prognóstico na CC cianótica após cirurgia corretiva.^7 , 86 , 87^	IIa	B
Avaliação do comportamento de arritmia e estratificação de risco.^82 , 83 , 88^	IIa	B
Prescrição e adequação de programa de exercícios, incluindo programa de reabilitação cardiovascular.^89 , 90^	IIa	B
Após cirurgia de Fontan, para avaliação da aptidão cardiorrespiratória e estratificação de risco/prognóstico.^84 , 91 , 92^	IIa	B
IC compensada após tratamento intervencionista para ajustes terapêuticos, estratificação de risco/prognóstico e liberação/prescrição de reabilitação cardiovascular.^93 , 94^	IIa	B
Avaliação de sintomas desencadeados ou agravados pelo esforço.^95 , 96^	IIa	C
Em assintomáticos, após reparo de tetralogia de Fallot, para avaliação de eventual substituição da valva pulmonar.^97 , 98^	IIb	B
Na tetralogia de Fallot reparada, para estratificação de risco/prognóstico.^99 , 100^	IIb	B
Na transposição corrigida das grandes artérias, para avaliação da aptidão cardiorrespiratória e estratificação de risco/prognóstico.^101 , 102^	IIb	B
Na doença de Fabry, para avaliação da aptidão cardiorrespiratória.^103 , 104^	IIb	B
Avaliação do grau de dessaturação com o esforço na CC cianótica clinicamente estável. * ^7^	IIb	B
CC com IC descompensada.	III	C
CC cianótica descompensada.	III	C

GR: grau de recomendação; NE: nível de evidência; CC: cardiopatia congênita; IC: insuficiência cardíaca.

* Recomenda-se realizar exame adicional de monitorização oximétrica não invasiva concomitantemente ao TE.

##### 2.2.6. Indicações no Contexto das Arritmias e Distúrbios de Condução

O TE está indicado no contexto das arritmias e distúrbios de condução em crianças e adolescentes para a avaliação de sintomas, diagnóstico de arritmias, definição de condutas terapêuticas, estratificação de risco e prescrição de exercícios físicos ( Tabela 6 ).^9 , 11 , 105 – 109^

**Tabela 6:** Indicações do TE no contexto das arritmias e distúrbios de condução em crianças e adolescentes

Indicação	GR	NE
Palpitação, síncope, pré-sincope, equivalente sincopal, mal-estar indefinido ou palidez relacionada ao esforço físico e/ou imediatamente após.^105 , 110 , 111^	I	B
Na suspeita de taquicardia ventricular paroxística catecolaminérgica.^112 , 113^	I	B
No BAVT congênito, para avaliação da resposta ventricular e consequente indicação de implante de marca-passo.^114 , 115^	I	B
No BAVT congênito, para escolha do tipo de marca-passo através da avaliação da resposta da frequência atrial.^116 , 117^	I	C
Avaliação e diagnóstico de disfunção do nó sinusal secundária a CC ou após correção cirúrgica.^118 , 119^	IIa	B
Eficácia da terapêutica farmacológica e/ou ablação de arritmia.^7 , 120 , 121^	IIa	B
Arritmia conhecida e controlada, para liberação e prescrição de exercícios físicos (incluindo reabilitação cardiovascular).^122 , 123^	IIa	B
Na síndrome do QT longo, para confirmação diagnóstica, estratificação de risco, avaliação de potencial arritmogênico e ajustes terapêuticos.^57 , 109 , 124^	IIa	B
Na suspeita de síndrome de Brugada, para auxílio diagnóstico e estratificação de risco.^125 – 127^	IIa	B
Avaliação da resposta da frequência cardíaca em portador de marca-passo com biossensor.^7 , 116 , 120 , 128^	IIa	C
Avaliação do comportamento de via anômala (pré-excitação) e do potencial arritmogênico.^7 , 120 , 121^	IIb	B
Portador de marca-passo de frequência fixa.	III	B
BAVT não congênito.	III	B
Avaliação de extrassístoles atriais e/ou ventriculares isoladas em crianças e adolescentes aparentemente saudáveis, sem comorbidades ou queixas.^7 , 120 , 129^	III	C
Arritmia não controlada, sintomática ou com comprometimento hemodinâmico.	III	C

GR: grau de recomendação; NE: nível de evidência; BAVT: bloqueio atrioventricular total; CC: cardiopatia congênita.

##### 2.2.7. Indicações em Valvopatias nas Crianças e Adolescentes

As valvopatias representam importante porcentagem das doenças cardíacas na população pediátrica, seja de origem congênita ou adquirida. A doença cardíaca reumática (DCR) e seu comprometimento valvar é uma das principais causas de morbidade e mortalidade cardíaca entre crianças nos países subdesenvolvidos e em desenvolvimento. Em 2019, cerca de 40 milhões de casos de DCR foram relatados em todo o mundo, com 2.789.443 novos casos e 305.651 mortes anualmente.^130 , 131^

A valvopatia pode gerar distúrbios hemodinâmicos dependendo do grau de acometimento valvar e miocárdico. As lesões estenóticas geralmente cursam com sobrecarga de pressão da câmara cardíaca envolvida, enquanto as lesões regurgitantes acarretam sobrecarga de volume. Muitas lesões são mistas, resultando em sobrecarga de pressão e volume, com potencial para desenvolvimento de insuficiência cardíaca (IC). Também é comum a ocorrência de valvopatias secundárias à cardiomiopatias adquiridas, miocardites e IC. Evolutivamente, o aumento do estresse da parede ventricular causa estiramento e fibrose miocárdica, resultando em cicatrizes com potencial arritmogênico. As arritmias podem complicar o quadro clínico da valvopatia e aumentar a morbimortalidade nas crianças e adolescentes.^132 – 134^

A Tabela 7 apresenta as indicações do TE em valvopatias específicas em crianças e adolescentes e os respectivos graus de recomendação.

**Tabela 7:** Indicações do TE em valvopatias nas crianças e adolescentes

Indicação	GR	NE
Em valvopatia discreta/moderada para avaliação de sintomas, arritmias, aptidão cardiorrespiratória e liberação de exercícios físicos.^81 , 135^	I	B
EAo, EAo supravalvular e estenose subaórtica, para avaliação de sintomas, na estratificação de risco e definição de intervenção.^81 , 134 , 136^	IIa	B
IAo compensada, moderada a grave, para avaliação de sintomas e aptidão cardiorrespiratória, indicação de intervenção e liberação de exercícios.^137 , 138^	IIa	B
Valva aórtica bicúspide, para avaliação da resposta inotrópica e estratificação de risco.^62 , 139^	IIa	B
Pós-intervenção valvar, para avaliação de sintomas, aptidão cardiorrespiratória, prognóstico e liberação de exercícios.^140 , 141^	IIa	B
EAo ou mitral grave sintomática, para avaliação da aptidão cardiorrespiratória.	III	B

GR: grau de recomendação; NE: nível de evidência; IAo: insuficiência aórtica; EAo: estenose aórtica.

##### 2.2.8. Indicações em Crianças e Adolescentes com Cardiopatias Adquiridas e Cardiomiopatias

As cardiomiopatias em crianças abrangem uma ampla gama de doenças que se manifestam como doença primária ou secundária a doenças sistêmicas (exemplos: distúrbio neuromuscular, secundária a HIV e COVID-19).^142 – 144^

As cardiomiopatias têm incidência anual estimada de 1,1 a 1,5 casos por 100.000 pacientes da faixa etária de 0 a 18 anos.^145^ Podem cursar com IC sistólica e/ou diastólica progressivas. A IC afeta 0,87 a 7,4 por 100.000 crianças e tem uma mortalidade em 5 anos de 40%.^146^ Nesses pacientes e nos recuperados de miocardites, o TE é indicado no acompanhamento clínico, decisões terapêuticas e prescrição/adequação de programa de exercícios ( Tabela 8 ).^6 , 143 , 147 , 148^

**Tabela 8:** Indicações do TE em crianças e adolescentes com cardiopatias adquiridas e cardiomiopatias

Indicação	GR	NE
Jovens recuperados de miocardite (incluindo viral), após 6 meses, para avaliação de arritmias esforço-induzidas.^62 , 149 , 150^	IIa	B
IC compensada secundária a cardiopatia para avaliação prognóstica, ajustes terapêuticos e liberação/prescrição de exercícios (incluindo reabilitação cardiovascular).^151 – 153^	IIa	B
Cardiomiopatia hipertrófica, para avaliação da aptidão cardiorrespiratória e de marcadores prognósticos (sintomas, arritmia ventricular e resposta inotrópica).^154 – 157^	IIa	B
Nas cardiomiopatias compensadas (exemplos: doença de Chagas e amiloidose), para seguimento e ajustes terapêuticos.^158 , 159^	IIb	B
Após transplante cardíaco, para avaliação da aptidão cardiorrespiratória, liberação/prescrição de programa de exercícios (incluindo reabilitação cardiovascular).^160 , 161^	IIb	B
Miocardite e pericardite agudas ou IC descompensada.	III	B
Diagnóstico de insuficiência cardíaca.	III	C
Seleção para transplante cardíaco pelo TE (com base nos valores de VO_2_ estimados e não medidos).	III	C

GR: grau de recomendação; NE: nível de evidência; IC: insuficiência cardíaca; VO_2_: consumo de oxigênio; TE: teste ergométrico.

##### 2.2.9. Outras Situações Clínicas em Crianças e Adolescentes

Há outras situações clínicas em que o TE está indicado no diagnóstico, avaliar a ACR e o estado hemodinâmico, auxiliar nas decisões terapêuticas e estratificar o risco em doenças específicas ( Tabela 9 ).

**Tabela 9:** Indicações do TE em outras situações clínicas de crianças e adolescentes

Indicação	GR	NE
Na suspeita de asma e obstrução laríngea esforço-induzidas e adequação de programa de exercícios.^162 – 164^	IIa	B
Após pelo menos 6 meses de miocardite ou pericardite recuperadas (incluindo por COVID-19), para avaliação pré-participação/liberação de prática de esportes.^150 , 165 , 166^	IIa	B
Anemia falciforme, para esclarecimento de sintomas, avaliar a aptidão cardiorrespiratória e liberar/prescrever exercícios físicos.^167 , 168^	IIa	C
Na hipertensão arterial pulmonar primária, para avaliação de sintomas, aptidão cardiorrespiratória e estratificação de risco/prognóstico.^169 , 170^	IIb	B
Pacientes em hemodiálise e pós-transplante renal, para prescrição e adequação de programa de exercícios (incluindo reabilitação cardiovascular).^171 – 173^	IIb	B
Avaliação de risco e prognóstico em pacientes oncológicos com suspeita de cardiotoxicidade.^104 , 174 , 175^	IIb	B
Após pelo menos 6 meses de recuperação de síndrome inflamatória multissistêmica (incluindo miocardite e pericardite) secundária a COVID-19.^150 , 165^	IIb	C

GR: grau de recomendação; NE: nível de evidência.

#### 2.3. Indicações do TCPE nas Crianças e Adolescentes

O TCPE, além das informações obtidas no TE, possibilita a avaliação dos volumes pulmonares (ergoespirometria) e a análise dos gases no ar expirado com a mensuração direta do consumo de oxigênio (VO_2_) e produção de dióxido de carbono (VCO_2_).^100 , 176 , 177^ Dessa forma, o TCPE pode ajudar a identificar a fisiopatologia da dispneia inexplicável, identificar características fisiopatológicas específicas de doenças e fornecer informações relevantes para decisões terapêuticas.^11 , 178^

Temos como indicações gerais do TCPE em crianças e adolescentes:^100 , 176 – 178^

1. Todas as indicações do TE descritas nesta diretriz em que seja necessária a quantificação das variáveis ventilatórias e metabólicas de maneira direta.
2. Aprimoramento da avaliação de sinais e/ou sintomas cardiorrespiratórios esforço-induzidos (dispneia, estridor laríngeo, sibilos etc.).
3. Avaliação aprimorada de doenças cardíacas (CC, valvopatias, IC, cardiomiopatias, arritmias), pulmonares e de outros órgãos (anemia falciforme, insuficiência renal, doenças neurodegenerativas etc.).
4. Contribuição para indicação e seguimento de tratamentos cirúrgicos específicos.
5. Avaliação da eficácia e ajustes terapêuticos.
6. Avaliação da ACR na indicação de transplante cardíaco.
7. Avaliação pré-participação e seguimento no esporte competitivo.
8. Avaliação prognóstica nas doenças cardiovasculares, pulmonares e em outros órgãos.
9. Pré-participação e seguimento na RCV.

As indicações específicas do TCPE e respectivos graus de recomendação e nível de evidência encontram-se na Tabela 10 .

**Tabela 10:** Principais indicações específicas para TCPE em crianças e adolescentes

Indicação	GR	NE
Avaliação de aptidão cardiorrespiratória.^176 , 179^	I	A
Ajuste de intensidade de treinamento aeróbico em esporte competitivo.^63 , 149 , 177 , 180 , 181^	I	A
Liberação e prescrição de exercícios em programa de reabilitação cardiovascular.^141 , 182 – 184^	I	A
Seleção de candidatos ao transplante cardíaco. * ^185 , 186^	I	A
Avaliação de dispneia ou asma esforço-induzidas.^178 , 179 , 187 , 188^	I	B
CC cianótica. ** ^81 , 100 , 189^	I	B
Seguimento após transplante cardíaco. ** ^190 – 192^	I	B
Doença de Kawasaki e arterite de Takayasu estabilizadas, para avaliação da aptidão cardiorrespiratória e liberação/prescrição de exercícios, incluindo reabilitação cardiovascular.^15 , 17 , 18 , 193^	I	C
Lesões assintomáticas de *shunt* direita-esquerda. ** ^184 , 194 , 195^	IIa	A
Hipertensão arterial pulmonar. ** ^169 , 196 – 198^	IIa	A
Lesões valvares regurgitantes moderadas a graves assintomática. ** ^81 , 199 , 200^	IIa	B
Estenose aórtica grave assintomática. ** ^81 , 199 , 200^	IIa	B
Fibrose cística, para avaliação da aptidão cardiorrespiratória e prognóstico.^67 , 201 , 202^	IIa	B
Doenças neuromusculares (esclerose múltipla, distrofia muscular de Becker e de Duchenne), para avaliação da aptidão cardiorrespiratória e prescrição de exercícios na reabilitação.^203 – 206^	IIa	B
Cardiomiopatia hipertrófica obstrutiva moderada assintomática. ** ^207 – 209^	IIa	B
Avaliação de pacientes em tratamento oncológico na suspeita de cardiotoxicidade, estratificação de risco e liberação/prescrição de exercícios.^104 , 174 , 175 , 210^	IIa	B
Lesões obstrutivas leves a moderadas do coração direito. ** ^81 , 199 , 211^	IIb	B
Após correção cirúrgica de CC, assintomática, estável hemodinamicamente. ** ^212 , 213^	IIb	B

GR: grau de recomendação; NE: nível de evidência; CC: cardiopatia congênita.

* Em indivíduos com idade, tamanho corporal, capacidade de compreensão e adaptação/colaboração imprescindíveis à correta realização do exame.

** Para avaliação da aptidão cardiorrespiratória, decisões terapêuticas e prognóstico.

#### 2.4. Indicações do TE e TCPE Associados a Métodos de Imagem

##### 2.4.1. Cintilografia de Perfusão Miocárdica

A cintilografia de perfusão miocárdica na população pediátrica tem seu papel estabelecido na avaliação de perfusão/viabilidade miocárdica e função ventricular. Pode ser útil na identificação de isquemia induzível ou residual, anormalidade do movimento de parede ventricular e estratificação de risco ( Tabela 11 ).

**Tabela 11:** Indicações da cintilografia miocárdica em crianças e adolescentes

Indicação	GR	NE
Avaliação tardia da doença de Kawasaki com comprometimento coronariano (com ou sem sintomas).^18 , 214 – 217^	I	C
Seguimento pós-operatório da transposição das grandes artérias, para pesquisa de isquemia miocárdica, estratificação de risco e indicação de reintervenção.^22 , 214 , 220 – 222^	IIa	B
Avaliação tardia da doença de Kawasaki sem comprometimento coronariano.^18 , 30 , 214 , 218 , 219^	IIb	B
Cardiomiopatia hipertrófica, para pesquisa de isquemia, estratificação de risco e manejo terapêutico.^214 , 223^	IIb	B
Pós-transplante cardíaco, para avaliação de doença vascular do enxerto.^214 , 224 , 225^	IIb	C
Na tetralogia de Fallot, após cirurgia de Fontan, para identificação de isquemia residual.^225 , 226^	IIb	C
Identificação de isquemia/fibrose miocárdica em pacientes com origem anômala da artéria coronária esquerda (origem na artéria pulmonar).^225 , 227 , 228^	IIb	C

GR: grau de recomendação; NE: nível de evidência.

##### 2.4.2. Indicações da Ecocardiografia sob Estresse

Na população pediátrica, a ecocardiografia sob estresse (EcoE) é mais comumente utilizada para a detecção de isquemia em pacientes com DAC, na doença de Kawasaki e origem anômala de coronárias. Outras indicações pediátricas incluem: pós-transplante cardíaco; cardiopatias congênitas para avaliação da resposta hemodinâmica e miocárdica; detecção precoce de disfunção miocárdica em populações específicas (exemplo: uso de antraciclinas); avaliação da resposta funcional do ventrículo direito (VD) e pressão da artéria pulmonar ( Tabela 12 ).^229 – 233^

**Tabela 12:** Indicações do ecocardiograma sob estresse em crianças e adolescentes com sintomas ou DCV^234 , 235^

Indicação	GR	NE
Pesquisa de insuficiência coronária em crianças pós-transplante cardíaco tardio.^160 , 236 – 238^	IIa	B
Avaliação tardia da doença de Kawasaki com comprometimento coronariano.^239 , 240^	IIa	B
No pós-operatório de cirurgia de Jatene, de origem e trajetos anormais das artérias coronárias e de fístulas coronário-cavitárias.^241 , 242^	IIa	B
Avaliação da função ventricular nas cardiomiopatias e nas insuficiências valvares (mitral e aórtica).^229 , 232 , 243^	IIa	B
Pacientes oncológicos com suspeita de cardiotoxicidade, para avaliação da função ventricular.^233 , 244 , 245^	IIa	B
Avaliação da função ventricular no pós-transplante.^160 , 237 , 238 , 246^	IIa	B
Pacientes sob risco de doença coronariana aterosclerótica precoce (hipercolesterolemia familiar homozigótica, diabetes mellitus I etc.).^247 , 248^	IIb	B
Pesquisa de insuficiência coronária na atresia pulmonar com septo ventricular íntegro ou estenose aórtica supravalvar.^229 , 249^	IIb	B
Avaliação do gradiente de pressão na cardiomiopatia hipertrófica e nas estenoses valvares (pulmonar e aórtica).^229 , 231 , 250 , 251^	IIb	B
Avaliação pós-operatória de cirurgias em plano atrial para transposição dos grandes vasos e de correção da tetralogia de Fallot.^222 , 252 , 253^	IIb	B

GR: grau de recomendação; NE: nível de evidência.

#### 2.5. Contraindicações Relativas e Absolutas do TE e TCPE em Crianças e Adolescentes

O TE/TCPE em população pediátrica não é um exame isento de riscos de complicações ou eventos adversos. Em algumas situações clínicas, o risco dos exames atinge uma magnitude que supera o benefício das informações obtidas, contraindicando-os. O TE em população pediátrica tem baixa morbidade e mortalidade, sendo que a incidência geral de complicações varia entre 0,5 e 1,79%.^7 , 11 , 254 , 255^ As complicações mais frequentes: dor torácica (0,69%), tontura ou síncope (0,29%), hipotensão (0,35%) e arritmias graves (0,46%).^254^ Em crianças e adolescentes com CC, a incidência de taquicardia ventricular (TV) variou de 1,9 a 7,3%.^256 , 257^

##### 2.5.1. Contraindicações Relativas do TE e TCPE em Crianças e Adolescentes

São situações clínicas de alto-risco ( Quadro 1 ) para a execução do TE/TCPE em população pediátrica e exigem a realização do exame em ambiente hospitalar (com pediatra emergencista de retaguarda) e adoção de cuidados especiais. Esses cuidados incluem: adequação de protocolos e carga de esforço a ser atingida; monitorização da saturação de oxigênio (oximetria); presença de pessoal e equipamento para possível reprogramação de marca-passo (MP)/cardioversor-desfibrilador implantável (CDI); etc.^6 – 11^

**Quadro 1:** Contraindicações relativas e precauções na realização do TE e TCPE em crianças e adolescentes^1 , 6 – 11^

Ambiente hospitalar + cuidados especiais
Estenoses valvares graves em assintomáticos *
Insuficiências valvares graves em assintomáticos *
Cardiopatias congênitas complexas (cianóticas ou acianóticas)
Hipertensão pulmonar moderada/grave
Síndromes arrítmicas genéticas (QT longo, taquicardia catecolaminérgica e síndrome de Brudaga) documentadas
Suspeita de arritmias complexas (taquiarritmias e bradiarritmias)
Síncope por provável etiologia arritmogênica ou suspeita de bloqueio atrioventricular de alto grau esforço-induzido
Cardiomiopatia arritmogênica de ventrículo direito ** ^258 , 259^
Marca-passo e CDI
Cardiomiopatia dilatada/restritiva com IC ou arritmia, quando estáveis clinicamente
Cardiomiopatia hipertrófica obstrutiva assintomática, com gradiente de repouso grave *
Obstrução grave da via de saída do ventrículo direito ou ventrículo esquerdo, quando assintomáticas *
Insuficiência cardíaca congestiva estável (Classe II ou III da NYHA)
TCPE na seleção de candidatos ao transplante cardíaco
Insuficiência renal dialítica
Suspeita de obstrução grave das vias aéreas *
SpO_2_ >85% em repouso, em ar ambiente, em uso de oxigênio suplementar *

NYHA: New York Heart Association; CDI: cardioversor-desfibrilador implantável; IC: insuficiência cardíaca; TCPE: teste cardiopulmonar de exercício; SpO_2_: saturação arterial de oxigênio.

* Situação onde o risco/benefício do exame deverá ser criteriosamente avaliada.

** Na suspeita e/ou para confirmação diagnóstica e investigação do desaparecimento da arritmia ventricular.

##### 2.5.2. Contraindicações Absolutas do TE e TCPE em Crianças e Adolescentes

As situações apresentadas no Quadro 2 são consideradas contraindicações absolutas, não devendo ser realizado o TE/TCPE em crianças e adolescentes.^7 , 9 , 11 , 105 , 181 , 188 , 260^

**Quadro 2:** Contraindicações absolutas do TE e TCPE em crianças e adolescentes^1 , 7 , 9 , 11 , 105 , 181 , 188 , 260^

Contraindicações absolutas
Enfermidade aguda, febril ou grave
Deficiência mental ou física com incapacidade de se exercitar adequadamente
Intoxicação medicamentosa
Deslocamento recente de retina, em fase de recuperação
Distúrbios hidroeletrolíticos e metabólicos não corrigidos
Hipertireoidismo descontrolado
Diabetes mellitus descompensada
Estenoses valvares graves sintomáticas
Insuficiências valvares graves sintomáticas
Cardiopatia congênita descompensada
Insuficiência cardíaca descompensada
Infarto do miocárdio recente
Embolia pulmonar aguda ou infarto pulmonar *
Febre reumática ativa, com ou sem cardite
Miocardite, pericardite ou endocardite agudas
Doença de Kawasaki na fase aguda
Arritmia cardíaca não controlada
Síndrome de Marfan com suspeita de dissecção aórtica
Aneurisma de aorta ou em outras artérias com indicação de intervenção
Hipertensão arterial sistêmica grave não controlada
Cardiomiopatia hipertrófica com história de síncope e/ou arritmia complexa
Estágio final de fibrose cística
Marca-passo unicameral, ventricular, sem resposta de frequência (VVI)
SpO_2_ ≤85% em repouso, em ar ambiente, com ou sem uso de oxigênio suplementar

SpO_2_: saturação arterial de oxigênio.

* Recente ou na fase crônica com grande repercussão clínica/hemodinâmica.

### 3. Aspectos Legais da Prática do TE e TCPE em Crianças e Adolescentes

Além dos aspectos legais e éticos do TE e TCPE já apresentados na Diretriz Brasileira de Ergometria para população adulta, devem ser considerados os aspectos específicos para a população pediátrica descritos a seguir.^1^

#### 3.1. Aspectos Legais da Prática do TE e TCPE

O TE e o TCPE são métodos não invasivos, com baixo risco de complicações em populações não selecionadas, fácil acessibilidade e reprodutibilidade. Por se tratarem de ato médico, são regidos pelo Código de Ética Médica e, assim, o médico deve conhecer as possíveis implicações éticas e jurídicas devidamente abordadas no próprio Código de Ética Médica do Conselho Federal de Medicina (CFM), Código Civil Brasileiro, Código de Proteção ao Consumidor e demais leis vigentes ( Anexo 1 ).^261 – 264^

#### 3.2. Condições Imprescindíveis à Realização do TE e TCPE em Crianças e Adolescentes

Baseado no exposto, são necessárias adoções de condições imprescindíveis às crianças e aos adolescentes:

1. O TE e o TCPE são atos médicos, de exclusiva competência do médico habilitado, inscrito no Conselho Regional de Medicina e apto ao exercício profissional. O Departamento de Ergometria, Exercício, Cardiologia Nuclear e Reabilitação Cardiovascular da Sociedade Brasileira de Cardiologia (DERC/SBC) recomenda que o médico possua Título de Especialista em Cardiologia e Título de Atuação em Ergometria da Associação Médica Brasileira (AMB) devidamente registrados no CFM e tenha realizado treinamento em TE/TCPE em população pediátrica.
2. Em se tratando de exame com potencial risco de complicações (que embora raras, inclui morte), quando realizado em menores de idade ou legalmente incapazes, recomenda-se que um dos pais ou seu representante legal permaneça na sala de exame. O médico executante deve reconhecer o adolescente, entre 12 e 18 anos de idade, como indivíduo possivelmente capaz e atendê-lo de forma diferenciada, respeitando sua individualidade, mantendo uma postura centrada na orientação e participação do adolescente.
3. É obrigatória a obtenção prévia de termo de consentimento livre e esclarecido (TCLE) assinado por um dos pais ou responsável legal. No exame em adolescente, recomenda-se que o mesmo seja incluído no processo de decisão, de forma a obter sua concordância na realização do exame dando seu assentimento livre e esclarecido (ALE). Caso haja recusa na assinatura do TCLE e/ou do ALE, o médico executante não poderá realizar o exame. Quando se tratar de pesquisa científica, tanto o TCLE quanto o ALE são obrigatórios. O termo assentimento é empregado para diferenciá-lo do consentimento, que é fornecido por pessoas adultas e totalmente capazes para tomar decisões.
4. O local destinado à realização dos exames deve dispor dos equipamentos adaptados à população pediátrica, bem como de equipamentos/medicamentos essenciais para o atendimento de emergências nessa população, conforme recomenda esta diretriz.^265 – 268^
5. O médico executante deverá seguir expressamente as recomendações das autoridades públicas e sanitárias e das entidades médicas referentes às possíveis endemias, epidemias e pandemias, assim como as normas dos núcleos de segurança do paciente.^269 – 271^
6. Todos os procedimentos pertinentes ao TE e TCPE descritos nesta diretriz devem ser observados e cumpridos.
7. O TE e o TCPE somente devem ser realizados com a solicitação formal médica.
8. Avaliar a presença de contraindicações relativas e absolutas para a realização do exame.
9. Na eventualidade de eventos adversos de natureza grave decorrentes do exame, o médico executante assumirá o suporte ao paciente até contato efetivo com o médico assistente e/ou eventual encaminhamento ao serviço de emergência. Sugere-se, em casos de evento fatal, a comunicação e solicitação de parecer da comissão de ética e do Conselho Regional de Medicina.
10. Orientar os pais/responsável legal a retornar ao médico solicitante para as devidas condutas. Caso seja arguido pelo paciente, pais ou seu representante legal sobre o resultado do exame, o médico executante deverá prestar as informações pertinentes.
11. A remuneração pelo exame realizado deve contemplar honorários médicos justos e todos os custos operacionais.
12. A realização de TE e/ou TCPE envolve a obtenção e o tratamento de dados sensíveis dos pacientes, devendo os serviços de ergometria respeitarem a Lei Geral de Proteção de Dados (LGPD) e legislações do CFM.^272 – 274^

#### 3.3. Termo de Consentimento e de Assentimento para o TE e TCPE em Crianças e Adolescentes

O modelo e o processo de obtenção de TCLE para a realização de TE e TCPE devem observar os critérios norteadores do Código de Ética Médica e Recomendação do CFM N° 1/2016, assinado por um dos pais ou pelo representante legal.^275^ No caso de adolescentes, é também recomendada a obtenção do ALE.

### 4. Condições Imprescindíveis à Capacitação em TE/TCPE em População Pediátrica

O TE/TCPE na população pediátrica é diferente dos exames realizados em adultos devido à prevalência específica de DCV (incluindo cardiopatias congênitas), às adequações de protocolos e parâmetros necessários aos exames, bem como às particularidades envolvidas na interpretação, no diagnóstico e definição de prognóstico.

Recomenda-se que os cardiologistas passem por capacitação/treinamento específico em TE/TCPE em população pediátrica:^276 , 277^

1. O treinamento poderá ocorrer durante (simultâneo) ou após a formação na área de atuação em ergometria (consultar a Parte 1, Sessão 4 da Diretriz Brasileira de Ergometria em População Adulta), de maneira adicional e complementar, incorporando as cargas horárias e os requisitos abaixo descritos. Esse treinamento não substitui a formação na área de atuação em ergometria, não confere titulação adicional e não cria nova área de atuação.^1^
2. O treinamento deverá ocorrer em instituição com serviço de ergometria atuante na população pediátrica, legalmente constituído, com inscrição nos órgãos públicos, documentação sanitária e registros regulares e atualizados. Essa instituição poderá ser submetida a processo de cadastramento, avaliação e credenciamento por parte do DERC/SBC.
3. Como pré-requisito obrigatório ao treinamento, o candidato deverá ter concluído Residência Médica em Cardiologia ou ser detentor do Título de Especialista em Cardiologia da AMB/CFM e estar em formação ou possuir o Título na Área de Atuação em Ergometria da AMB/CFM.
4. O treinamento deverá permitir que o cardiologista adquira experiência no TE e TCPE em população pediátrica (crianças e adolescentes), de modo a ser responsável pela organização de serviços, realização e interpretação desses exames. O programa deverá ser teórico-prático, com carga horária mínima de 100 horas.
5. O programa teórico poderá ser feito na própria instituição ou em parceria com o DERC/SBC, devendo incluir no mínimo todos os tópicos e assuntos abordados nesta diretriz. Recomenda-se conteúdo programático teórico adicional:
  – Revisão das DCV em crianças e adolescentes (incluindo cardiopatias congênitas), seus tratamentos e exames complementares.
  – Revisão das medicações utilizadas em população pediátrica e ajustes posológicos.
  – Fisiologia cardiovascular e fisiologia do exercício em população pediátrica saudável e com doenças cardíacas (incluindo cardiopatias congênitas não reparadas, tratadas ou sob tratamento paliativo).
6. O treinamento prático deverá corresponder a pelo menos 80% do período total do treinamento, devendo contemplar TE e TCPE. Deve ocorrer sob supervisão direta e presencial de preceptor que possua Título de Especialista em Cardiologia, Título da Área de Atuação em Ergometria e experiência na realização dos exames em população pediátrica. O treinamento prático deve ter uma proporção de um preceptor para no máximo dois alunos.
7. É recomendado o treinamento regular em atendimento de urgências correspondendo aos cursos de *Pediatric Advanced Life Support* (PALS) e de *Advanced Cardiovascular Life Support* (ACLS).
8. A instituição deverá se responsabilizar pela elaboração e submissão da avaliação dos alunos, durante e/ou ao final do programa do treinamento. Recomenda-se transparência nas avaliações, definindo previamente os critérios objetivos que serão exigidos. Caso não haja aprovação, sugere-se que a instituição forneça opções de treinamento adicional para sanar pendências, seguido de nova avaliação. A instituição deverá fornecer certificado oficial ao aluno aprovado e declaração de cumprimento de todos os itens correspondentes ao treinamento teórico-prático.
9. É imprescindível que, após o término da formação, haja participação periódica em eventos científicos/programas de atualização em TE e TCPE em crianças e adolescente, em âmbito nacional e/ou internacional, para aperfeiçoamento constante dos conhecimentos adquiridos.

## Parte 2 – Teste Ergométrico

### 1. Metodologia do TE em Crianças e Adolescentes

#### 1.1. Condições Básicas para a Programação do TE/TCPE

##### 1.1.1. Equipe

O TE/TCPE deve ser realizado por médico habilitado, com experiência no método em crianças e adolescentes e com treinamento em PALS.

Profissionais da área de saúde (auxiliar de enfermagem ou técnica de enfermagem ou enfermeira) que estejam auxiliando o médico executante deverão estar treinados no cuidado de crianças e adolescentes, bem como no auxílio ao atendimento de eventuais emergências nessa população.^265 – 268^

Nos casos de TE/TCPE em pacientes com cardiopatias congênitas complexas ou de risco aumentado de complicações (vide Quadro 1 ), recomenda-se a realização do exame em ambiente hospitalar com pediatra emergencista de retaguarda.

A instituição e/ou o médico executante deverão orientar e treinar adequadamente outros possíveis profissionais envolvidos no TE/TCPE na marcação/orientação do exame, higienização de equipamentos, limpeza da área física e cuidado/transporte de pacientes.

##### 1.1.2. Área Física

Ambiente planejado, adequadamente iluminado e ventilado, com dimensões suficientes para a acomodação de todos equipamentos do TE/TCPE (incluindo maca, cadeiras para paciente e acompanhante e carro de emergência) e a aparelhagem adicional necessária aos exames em crianças e adolescentes. Deve permitir circulação de pelo menos quatro pessoas (no mínimo de 10 m^2^), com temperatura ambiente entre 18 e 22 ^o^C, sendo desejável umidade relativa do ar em pelo menos 40%. É necessária a presença de um dos pais ou responsável legal na sala de exame.^264 , 278 – 284^

##### 1.1.3. Equipamentos

Equipamentos básicos recomendados: ergômetro; sistema de ergometria com monitor para observação do eletrocardiograma (ECG); impressora (ou acesso para servidor de impressão); esfigmomanômetro calibrado e estetoscópio; termômetro de parede e higrômetro; oxímetro digital; cadeiras destinadas ao paciente, acompanhante e médico; maca ou cama; carro de emergência (se sala única); cilindro de oxigênio (junto ao carro de emergência) ou ponto de oxigênio em cada sala de TE/TCPE; aspirador portátil (junto ao carro de emergência) ou ponto de aspiração em cala sala; lixeiras (lixo comum e hospitalar).^149 , 264 , 278 – 280 , 285 , 286^

Os equipamentos devem ser customizados para a população pediátrica:

1. Os ergômetros devem ser adaptados à idade, estatura e peso da criança/adolescente:
  – Esteira ergométrica com barras laterais de segurança e barra frontal com altura ajustável para permitir o apoio das mãos de crianças de menor estatura. Deve permitir velocidade inicial menor para que as crianças mais novas se adaptem à caminhada.
  – Colocar tapete acolchoado no chão, logo após o final da esteira, para proteção da criança.
  – A bicicleta ergométrica deve permitir ajustes na altura do banco, na altura e posição do guidão, no tamanho da alça do pedal e menor frenagem para adaptação ao ato de pedalar em crianças mais novas.
  – Sugere-se a utilização de arnês de segurança em crianças mais novas, constituído por conjunto de tiras ligadas entre si, envolvendo o tronco e a cintura, fixadas na esteira ou suporte próprio.
2. No TCPE, utilizar máscara facial ou sistema bucal para população pediátrica, que permita os necessários ajustes.
3. Eletrodo de monitorização cardíaca infantil/pediátrico (em crianças mais novas). Em adolescentes com maior estatura e circunferência torácica, poderá ser utilizado eletrodo de monitorização cardíaca para adultos.
4. Conjunto de manguitos de vários tamanhos para aferição da PA em população pediátrica.^287^
5. Sistema de ergometria que adote os critérios e parâmetros para a população pediátrica e que permita ampliação do ECG para adequada visualização do sinal.
6. Em caso de realização de oximetria não invasiva simultaneamente/adicionalmente ao TE/TCPE, utilizar sensores de modelo pediátrico.

##### 1.1.4. Material e Medicamentos para Emergência Médica

O serviço deverá manter disponível um carro de emergência pediátrica, com material e medicação para suporte básico e avançado de vida, no local de realização do TE e/ou TCPE, conforme padronização do carro de emergência da Diretriz de Ressuscitação Cardiopulmonar e Cuidados Cardiovasculares de Emergência da Sociedade Brasileira de Cardiologia (Quadro 17.3 – Padronização do carro de emergência pediátrica na unidade de internação, terapia intensiva e pronto-socorro).^266^

##### 1.1.5. Orientações ao Paciente e Pais/Responsáveis na Marcação do TE/TCPE

É necessário fornecer à família e ao paciente orientações no momento da marcação (idealmente por escrito) sobre o preparo pré-teste que permitirá a execução adequada do exame. Quando o exame for de crianças muito novas, sugere-se orientar os pais a explicarem as recomendações de modo a obter uma maior cooperação.

Recomendações:^7 , 177^

1. O paciente deverá vir descansado para o exame (evitar realizar esforços físicos no dia do exame).
2. Evitar jejum ou alimentação excessiva antes do exame; fazer uma refeição leve 2 horas antes. Não consumir bebidas com cafeína (incluindo refrigerante) no dia do exame.
3. O paciente deverá comparecer com roupas confortáveis para a prática de exercícios físicos: shorts (ou bermuda), camiseta e calçado esportivo (de preferência tênis; evitar sapatos abertos ou sandálias). Recomenda-se que adolescente do sexo feminino utilize sutiã ou *top* .
4. Trazer o pedido médico do exame.
5. Sugere-se trazer TE/TCPE realizado anteriormente.
6. A suspensão ou manutenção de uso de medicações ficará a critério do médico assistente do paciente.
7. Um dos pais ou representante legal deverá acompanhar a criança/adolescente na realização do exame.
8. No caso de TCPE, orientar o paciente que será necessário o uso de máscara facial durante o exame, o que não interfere na respiração.

Observação: no caso de adolescentes, verificar se fazem uso de bebida alcoólica e/ou tabagismo (mesmo que esses sejam proibidos por lei) e, quando for o caso, orientar a interrupção para a realização do exame.

##### 1.1.6. Orientações ao Paciente e Pais/Responsáveis no Momento da Realização do Exame

Quando a criança/adolescente e seus pais (ou representante legal) chegarem para a realização do exame, todo o procedimento deve ser explicado em linguagem e termos de modo que ambos possam entender. A criança e os pais devem ter a oportunidade de realizar todas as perguntas que desejarem visando esclarecer qualquer possível dúvida sobre o exame.^11^

A equipe deve demonstrar à criança/adolescente o modo de utilização do ergômetro e deixar claro que o exame normalmente não provoca dor e pode ser até divertido. Orientar os pais de que a criança/adolescente:

– Realizará um esforço físico (andar na esteira ou pedalar), dentro das suas habilidades e capacidade, podendo interromper o esforço a qualquer momento que ela desejar ou precisar.
– O médico e a equipe realizarão vários procedimentos necessários à monitorização e registro dos dados do exame.
– Com o esforço, poderão ocorrer sintomas como cansaço e outros associados às doenças da criança/adolescente.

##### 1.1.7. Orientações quanto ao Uso de Medicações

Diferentemente do que ocorre em adultos, são raras as indicações de suspensão de medicações para a realização do TE/TCPE em crianças e adolescentes. Geralmente, a população pediátrica só faz uso de medicamentos de comprovada necessidade para o controle e estabilidade clínica de suas doenças. Quando necessária, a suspensão deve ser indicada pelo médico assistente da criança/adolescente, levando em consideração os riscos envolvidos. Para determinação do período de suspensão considerar o tempo de eliminação de cada medicamento e suas variações na faixa etária pediátrica.^6 – 12^

Nos pacientes com asma, a manutenção do uso de medicamentos deve considerar a indicação do exame.^288^ Na suspensão de outras medicações considerar possíveis interferências no desempenho físico, na resposta cronotrópica, no limiar de isquemia e angina, na resposta do segmento ST, nas arritmias esforço-induzidas etc.

#### 1.2. Procedimentos Básicos para a Realização do Exame

O TE/TCPE em crianças é mais desafiador do que em adolescentes, especialmente naquelas com comprometimento crônico da saúde. As dificuldades em testá-las surgem por três razões:^289^

1. Tamanho corporal muito pequeno mesmo quando o equipamento é adaptado à população pediátrica.
2. Capacidade física muito baixa, dificultando a adaptação, mesmo com a utilização de protocolos com pequenos incrementos da carga de esforço.
3. Geralmente apresentam um menor período de atenção, menor motivação durante exames longos e baixa colaboração, tornando difícil diferenciar a limitação ao esforço de falta de cooperação.

##### 1.2.1. Fase Pré-teste, Avaliação Inicial, Exame Físico Sumário e Específico

Recomenda-se ao médico executante avaliar a indicação do exame e os sintomas atuais do paciente, constatar se as orientações pré-teste foram cumpridas, realizar anamnese e exame físico dirigidos aos sistemas cardiovascular e pulmonar ( Quadro 3 ).^264 , 279 , 280^

**Quadro 3:** Recomendações quanto à anamnese e exame físico dirigido em paciente pediátrico^1 , 264 , 279 , 280^

Anamnese	Exame Físico
Sintomas atuais *	Ectoscopia geral (anemia, faces sindrômicas, palidez cutânea, cianose)
Antecedentes patológicos/cirúrgicos **	FC/PA *3
História familiar de morte súbita ou doença arterial coronariana precoce **	Ausculta cardíaca e pulmonar
Fatores de risco (vide Parte 2, Sessão 2 – Estratificação de Risco Pré-TE) **	Oximetria não invasiva *4
Medicações em uso **	Pulsos periféricos e índice tornozelo-braquial *5
Tolerância ao esforço físico *	
Realizou TE/TCPE anteriormente? Teve alguma anormalidade? **	

TE: teste ergométrico; TCPE: teste cardiopulmonar de esforço; FC: frequência cardíaca; PA: pressão arterial.

* Verificados tanto com a criança/adolescentes quanto com seus pais/representante legal.

** Verificados principalmente através de informações dos pais/representante legal.

*3 Utilizar manguito adequado a circunferência do membro superior.

*4 Exame adicional. Indicado principalmente nos casos de cardiopatia congênita cianótica, insuficiência cardíaca, valvopatas, pós-COVID.

*5 Exame adicional. Indicado na investigação de coarctação de aorta, síndrome de Takotsubo e claudicação de membros inferiores.

É imprescindível verificar a possibilidade de eventuais contraindicações relativas e absolutas para a realização do TE/TCPE, bem como informações sobre tratamentos realizados anteriormente (especialmente nos casos de CC). Não é recomendado o uso de escore clínico pré-teste de adultos (não são validados para a população pediátrica).

##### 1.2.2. Sistema de Monitoração e Registro Eletrocardiográfico

A monitorização contínua do ECG e a realização de registros são obrigatórios em todas as etapas do exame (repouso, esforço e recuperação). Recomenda-se a utilização de sistema computadorizado de ergometria para a monitorização do ECG e de *software* que permita a obtenção, registro e interpretação de dados em população pediátrica. Sugere-se utilizar eletrodo para monitorização de ECG de longa duração, hipoalergênico, extra-aderente e em crianças pequenas utilizar o eletrodo infantil/pediátrico.^264 , 279 , 280^

Recomenda-se seguir as orientações da Diretriz Brasileira de Ergometria para a População Adulta quanto ao número de derivações a serem utilizadas (12 ou 13) e ao posicionamento dos eletrodos. Nos sistemas de 12 derivações utilizar o posicionamento clássico de Mason-Likar ou sua versão modificada preservando CM5. Nos sistemas de 13 derivações utilizar o posicionamento das derivações clássicas com adição de CM5. Não é mais recomendado o uso de sistemas de três derivações, tendo em vista a superioridade dos sistemas com 12, 13 ou mais derivações.^1^

Os procedimentos de preparo da pele são similares aos dos adultos, inclusive quanto à eventual necessidade de retirada de excesso de pelos nos adolescentes do sexo masculino nas regiões de fixação dos eletrodos. Em crianças pequenas, no momento da limpeza da pele com álcool, devido à maior sensibilidade, deve-se ter o cuidado de evitar o excesso de abrasão da pele e, também, assegurar que o procedimento não indica recebimento de uma injeção (muitas crianças associam compressas com álcool a injeções). Pode-se utilizar um colete ou uma camisa de rede para ajudar a manter os eletrodos e fios firmemente no lugar.

##### 1.2.3. Ergômetros

A escolha do ergômetro deve ser individualizada levando-se em conta a idade da criança e adolescente, estatura, capacidade de adaptação, segurança e eventuais limitações físicas. São três os principais tipos de ergômetros utilizados no TE/TCPE: bicicleta ergométrica, esteira ergométrica e cicloergômetro de braço. Tanto a bicicleta ergométrica quanto a esteira produzem cargas máximas adequadas, confiáveis e reprodutíveis, permitindo a coleta de informações diagnósticas e de desempenho físico.^290^

###### 1.2.3.1. Bicicleta Ergométrica

A utilização de bicicleta ergométrica (cicloergômetro de membros inferiores) é mais frequente em crianças a partir dos 6 anos de idade. Crianças que não estão acostumadas ao ciclismo geralmente apresentam:

– Fadiga muscular precoce nos membros inferiores, podendo não atingir o esforço máximo.
– Dificuldade em manter a cadência do pedalar entre 40 e 70 rotações por minuto (rpm).
– Dificuldade em manter os pés nos pedais, mesmo quando ajustados para o tamanho da criança.

Para acomodar adequadamente as crianças e adolescentes na bicicleta ergométrica, deve-se ajustar a altura do assento, a altura e a posição do guidão e o tamanho da alça do pedal. Crianças e adolescentes com estatura ≥125 cm poderão realizar o TE/TCPE em bicicleta ergométrica padrão para adultos.^200^ A utilização de bicicleta ergométrica é preferida quando for necessária uma avaliação mais precisa da PA.

###### 1.2.3.2. Esteira Ergométrica

O TE/TCPE em esteira ergométrica é possível em crianças a partir dos 3 anos de idade, pois as mesmas estão mais familiarizadas com o andar rápido ou até mesmo correr. Entretanto, o esforço na esteira não é uma forma natural de caminhar, sendo recomendável previamente avaliar a capacidade de adaptação e a coordenação ao ergômetro. Recomenda-se ajustar a altura da barra frontal de apoio conforme a estatura da criança.

Em TE de crianças muito pequenas ou limitadas sugere-se:^6 – 12^

– Utilização de arnês de segurança de modo a proteger a criança em caso de mal súbito ou desequilíbrio.
– Utilização de grades laterais e tapete acolchoado colocado no chão ao final da esteira para proteção da criança.
– Permanência de um membro extra da equipe executora, posicionado imediatamente atrás da esteira, para auxílio e proteção da criança durante o esforço.

O TE em esteira geralmente apresenta consumo máximo de oxigênio (VO_2_max) ≈10% superior ao obtido na bicicleta ergométrica.^291 – 293^

###### 1.2.3.3. Cicloergômetro de Braço

Em nosso meio, o cicloergômetro de braço é raramente utilizado em crianças e adolescentes. Geralmente, é empregado em pacientes com deficiência de mobilidade nos membros inferiores causada por lesão medular (torácica ou lombar superior), amputação de membro inferior, meningocele, espinha bífida etc.^294 , 295^

O TE com cicloergômetro de braço, usando um protocolo de rampa validado, permite avaliar adequadamente a aptidão cardiorrespiratória de crianças e adolescentes.^295 , 296^

##### 1.2.4. Escolha do Protocolo

A escolha do protocolo deve ser individualizada considerando a finalidade do exame, a prática de atividades físicas diárias, eventuais limitações físicas e visando tempo de esforço de ≈10 minutos (entre 6 e 12 minutos). O protocolo também deverá respeitar as características do paciente: idade, tamanho corporal, capacidade de adaptação ao incremento de carga de esforço etc.^6^

Os protocolos são divididos quanto à forma do esforço:

1. Incrementais (aumento gradativo de carga):
  – Escalonado (em degraus): com aumento de cargas em estágio (etapas) em tempo predeterminado (a cada um ou mais minutos por estágio).
  – Rampa: com incrementos pequenos de carga, frequentes (tendendo a linear) e em curtos intervalos de tempo (incrementos em segundos, sempre inferiores a 1 minuto).
2. Sem incremento (carga fixa): sem aumento de cargas. Em esteira ergométrica, mantém velocidade e inclinação fixas.^1 , 12 , 285^

###### 1.2.4.1. Protocolos para Bicicleta Ergométrica

Os principais protocolos para bicicleta ergométrica são apresentados na Tabela 13 . A carga de trabalho realizada na bicicleta normalmente é expressa em watts (W). A maioria dos protocolos requer uma cadência de pedalar entre 50 e 60 rpm, limitando a variação entre 40 e 70 rpm.

**Tabela 13:** Comparação dos principais protocolos para bicicleta ergométrica

Protocolo	Indicado para	Carga inicial	Incremento de carga
Balke	Crianças e adolescentes saudáveis	25 W	25 W a cada 2 minutos
Astrand^299^	Crianças e adolescentes	25 W	25 W a cada 3 minutos
McMaster * ^300^	Crianças ** e adolescentes ***	12,5 W a 25 W	5 modos de incremento, dependendo da estatura e do sexo, a cada 2 minutos
James * ^301 , 302^	Crianças e adolescentes, ativos	33 W	3 modos de incremento, dependendo da área de superfície corporal, a cada 3 minutos
Godfrey * ^303 , 304^	Crianças* e adolescentes	10 W a 20 W	3 modos de incremento, dependendo da estatura, a cada 1 minuto
Rampa^297 , 305^	Todas as populações sendo o ideal para atletas	10 W a 20 W	5 a 20 W a cada 1 minuto. Subdividir o aumento em valores iguais e o incremento em intervalos regulares (<60 segundos) ****

* Vide Tabela 14 para descrição detalhada dos protocolos;

** Baseado na estatura, correspondendo a crianças a partir dos 6 anos de idade;

*** Pacientes com doenças cardíacas, pulmonares ou musculares podem exigir reduções na carga inicial de trabalho;

**** Exemplo: protocolo de rampa com incremento de carga de 15 W a cada 1 minuto = aumentar a carga em 5 W a cada 20 segundos.

Os protocolos de Balke e de Astrand apresentam a desvantagem de serem fixos, não considerarem o tamanho corporal, podendo ser muito intensos para crianças mais novas (principalmente as cardiopatas).

Nos protocolos de McMaster, James e Godfrey, as cargas iniciais e os incrementos são feitos de acordo com o tamanho corporal [estatura ou área de superfície corporal (ASC)] e/ou sexo ( Tabela 14 ). Em adolescentes, utilizam-se elevadas cargas de esforço, o que pode ser uma limitação para os sedentários ou doentes (cardiopatas, pneumopatas etc.).^297 , 298^

**Tabela 14:** Descrição dos Protocolos de Godfrey, McMaster e James para bicicleta ergométrica^300 – 304^

	Protocolo de Godfrey Estágio: 1 min Ritmo: 60 rpm			Protocolo de McMaster Estágio: 2 min Ritmo: 50 rpm					Protocolo de James Estágio: 3 min Ritmo: 60-70 rpm		
	Altura <120 cm *	Altura 120-150 cm *	Altura >150 cm *	Altura ≤119,9 cm *	Altura 120-139,9 cm *	Altura 140-159,9 cm *	Altura ≥160 cm (masc)	Altura ≥160 cm (femin)	ASC <1,0 *	ASC 1,0-1,2 *	ASC >1,2 *
Tempo (min)	10 W/est	15 W/est	20 W/est	12,5 W/est	25 W/est	50 W/est	50 W/est	25 W/est	16,5 W/est	33 W/est	49,5 W/est
**0**	10 W	15 W	20 W	12,5 W	12,5 W	25 W	25 W	25 W	33 W	33 W	33 W
**1**	10 W	15 W	20 W								
**2**	20 W	30 W	40 W	25 W	37,5 W	50 W	75 W	50 W			
**3**	30 W	45 W	60 W						49,5 W	66 W	82,5 W
**4**	40 W	60 W	80 W	37,5 W	62,5 W	75 W	125 W	75 W			
**5**	50 W	75 W	100 W								
**6**	60 W	90 W	120 W	50 W	87,5 W	100 W	175 W	100 W	66 W	99 W	132 W
**7**	70 W	105 W	140 W								
**8**	80 W	120 W	160 W	62,5 W	112,5 W	125 W	225 W	125 W			
**9**	90 W	135 W	180 W						82,5 W	132 W	181,5 W
**10**	100 W	150 W	200 W	75 W	137,5 W	150 W	275 W	150 W			
**11**	110 W	165 W	220 W								
**12**	120 W	180 W	240 W	87,5 W	162,5 W	175 W	325 W	175 W	99 W	165 W	231 W
**13**	130 W	195 W	260 W								
**14**	140 W	210 W	280 W	100 W	187,5 W	200 W	375 W	200 W			
**15**	150 W	225 W	300 W						115,5 W	198 W	280,5 W
**16**	160 W	240 W	320 W	112,5 W	212,5 W	225 W	425 W	225 W			
**17**	170 W	255 W	340 W								
**18**	180 W	270 W	360 W	125 W	237,5 W	250 W	475 W	250 W	132 W	231 W	330 W
**19**	190 W	285 W	380 W								
**20**	200 W	300 W	400 W	137,5 W	262,5 W	275 W	525 W	275 W			

ASC: área de superfície corporal – metros quadrados (m^2^); W: Watts; min: minuto; rpm: rotações por minuto; est: estágio; cm: centímetro; masc: masculino; femin: feminino.

* Para ambos os sexos.

###### 1.2.4.2. Protocolos para Esteira Rolante

####### 1.2.4.2.1. Protocolos Escalonados

######## 1.2.4.2.1.1. Protocolo de Bruce

É o protocolo escalonado mais utilizado ( Tabela 15 ). É mais apropriado para o TE em crianças sem cardiopatia grave e em adolescentes aparentemente saudáveis, inclusive pré-escolares. Pode ser utilizado em TE seriados para a comparação de dados à medida que a criança cresce. Desvantagens potenciais:

**Tabela 15:** Principais protocolos escalonados para a população pediátrica e suas características^1 , 7 , 306^

Estágio	Bruce			Bruce modificado			Ellestad			Balke			Naugthon			Naugthon modificado		
	Min	mph/km/h	E%	Min	mph/km/h	E%	Min	mph/km/h	E%	Min	mph/km/h	E%	Min	mph/km/h	E%	Min	mph/km/h	E%
**01**	3	1,7/2,7	10	3	1,7/2,7	0	3	1,7/2,7	10	1	3,5/5,6	1	2	1,0/1,6	0	2	3,0/4,8	0
**02**	6	2,5/4,0	12	6	1,7/2,7	5	5	3,0/4,8	10	2	3,5/5,6	2	4	2,0/3,2	0	4	3,0/4,8	2,5
**03**	9	3,4/5,5	14	9	1,7/2,7	10	7	4,0/6,4	10	3	3,5/5,6	3	6	2,0/3,2	3,5	6	3,0/4,8	5,0
**04**	12	4,2/6,7	16	12	2,5/4,0	12	10	5,0/8,0	10	4	3,5/5,6	4	8	2,0/3,2	7,0	8	3,0/4,8	7,5
**05**	15	5,0/8,0	18	15	3,4/5,5	14	12	6,0/9,7	15	5	3,5/5,6	5	10	2,0/3,2	10,5	10	3,0/4,8	10
**06**	18	5,5/8,8	20	18	4,2/6,7	16	14	7,0/9,6	15	6	3,5/5,6	6	12	2,0/3,2	14,0	12	3,0/4,8	12,5
**07**	21	6,0/9,7	22	21	5,0/8,0	18	16	8,0/11,2	15	7 a 21	3,5/5,6	+1%/min *	14	2,0/3,2	17,5	14	3,0/4,8	15,0
**08**	24	6,5/10,5	24	24	5,5/8,8	20	18	9,0/12,8	15	22	3,5/5,6	22,0	16	2,0/3,2	21,0	16	3,0/4,8	17,5

Min.: minutos; mph: milhas por hora; km/h: quilômetros por hora; E%: elevação/inclinação da esteira (em %); MET: metabolic equivalent of task (equivalente metabólico da tarefa).

* Aumentar 1% na inclinação a cada minuto do exame (velocidade constante).

– Em crianças mais novas ou mais limitadas, os incrementos de esforço entre os estágios podem ser muito grandes, levando frequentemente à desistência no primeiro minuto de um novo estágio.
Pode ser demasiadamente longo em jovens treinados/atletas, causando, inclusive, tédio.

######## 1.2.4.2.1.2. Protocolo de Bruce Modificado

O protocolo de Bruce modificado, sem inclinação inicial, é mais adequado para crianças menores ou mais limitadas fisicamente. É utilizado em crianças a partir de 3 anos, portadoras de cardiopatia ou pneumopatia. A maior limitação é que após o terceiro estágio ocorrem incrementos abruptos de esforço a cada estágio (semelhante ao Protocolo de Bruce).

######## 1.2.4.2.1.3. Protocolo de Ellestad

Emprega aumentos expressivos de velocidade, sendo indicado preferencialmente para adolescentes fisicamente ativos e atletas. As principais limitações desse protocolo são: altas velocidades em cargas iniciais, dificultando a adaptação de quem não está acostumado a correr; dificuldade para realização de medições pressóricas.

######## 1.2.4.2.1.4. Protocolo de Balke

O protocolo de Balke incorpora uma velocidade constante da esteira (3,5 mph) com inclinação crescente de 1% a cada minuto. É mais adequado para crianças obesas, crianças muito novas, cronicamente doentes e/ou muito limitadas.^7 , 306^

Uma desvantagem é que, em pacientes fisicamente ativos, a duração do exame é muito longa. Para esses pacientes, é preferível a versão modificada do protocolo de Balke (" *running Balke* ") utilizando velocidade constante mais rápida, visando a manter o tempo de esforço entre 8 e 10 minutos.

######## 1.2.4.2.1.5. Protocolo de Naughton

Existem várias adaptações do protocolo de Naughton para a população pediátrica, variando a velocidade inicial e inclinação, envolvendo pequenos incrementos de carga por estágio, permitindo melhor adaptação de crianças menores e/ou com limitações físicas. Não deve ser utilizado em crianças e adolescentes saudáveis por prolongar demasiadamente o exame.^307^

####### 1.2.4.2.2. Protocolo em Rampa

O protocolo em rampa pode ser totalmente individualizado às características da criança/adolescente, variando a velocidade, a inclinação (iniciais e finais) e a duração do exame. Permite a melhor determinação do consumo máximo de oxigênio (direto ou estimado), limiares ventilatórios, medição ou estimativa da potência máxima, avaliação das causas da limitação do esforço, avaliação de isquemia e arritmias. Deverá manter a meta de duração do exame entre 8 e 12 minutos com a inclinação da rampa ajustada ao tamanho e às habilidades físicas da criança.

Em crianças cardiopatas, sugere-se programar o protocolo com velocidade inicial de 1 km/hora (sem inclinação) e, posteriormente, realizar pequenos e constantes incrementos na intensidade do esforço.

A Tabela 16 apresenta a individualização do protocolo de rampa baseada em estudo na população infanto-juvenil brasileira, que se mostrou mais confortável do que o protocolo de Bruce.^121^

**Tabela 16:** Individualização do protocolo em rampa por sexo e faixa etária, baseado em estudo na população pediátrica brasileira

Faixa etária (anos)	Sexo feminino					Sexo masculino				
	Velocidade (km/h)		Inclinação (%)		VO_2_max	Velocidade (km/h)		Inclinação (%)		VO_2_max	
	Inicial	Final	Inicial	Final	Média ± DP	Inicial	Final	Inicial	Final	Média ± DP
**4-7**	3,0	6,5	4,0	14,0	39,4±4,7	3,5	7,5	5,0	15,0	45,3±9,2
**8-11**	3,5	7,0	5,0	15,0	43,9±6,2	4,0	8,0	5,0	15,0	48,6±7,9
**12-14**	4,0	8,0	5,0	15,0	48,3±7,3	4,0	8,5	6,0	16,0	53,2±9,0
**15-17**	4,0	8,0	5,0	15,0	47,8±10,1	4,5	9,0	6,0	16,0	55,1±9,4

DP: desvio padrão; km/h: quilômetros por hora; VO_2_max: consumo máximo de oxigênio. Duração final prevista de 10 minutos. Adaptado de: Silva et al. Teste ergométrico em crianças e adolescentes: maior tolerância ao esforço com o protocolo em rampa.^121^

##### 1.2.5. Monitorização da Frequência Cardíaca

A FC é monitorada e medida diretamente do traçado eletrocardiográfico durante todas as fases do TE/TCPE. Recomenda-se o registro da FC, pelo menos no pré-esforço, ao final de cada estágio de protocolo escalonado ou a cada 2 minutos em protocolo de rampa e na recuperação (1, 2, 4 e 6 minutos). Caso necessário, o registro deve ser mantido por tempo maior na recuperação.

Recomenda-se a realização de ECG convencional de 12 derivações de forma adicional, precedendo o TE/TCPE. O ECG convencional é um exame complementar que permite avaliar a condição cardíaca, podendo, inclusive, contribuir para a eventual contraindicação do exame. O ECG convencional de 12 derivações é um procedimento médico previsto na Classificação Brasileira Hierarquizada de Procedimentos Médicos (Código: 4.01.01.01-0).^1 , 278 , 308^

No TE, conceitua-se como:^95 , 264^

– FC máxima (FCmax) é aquela atingida em nível de exaustão ao esforço.
– FC de pico (FCpico) é a maior FC observada no pico do esforço, mesmo que não haja exaustão.

É importante ressaltar que, em crianças aparentemente saudáveis, a FCmax praticamente não muda ao longo da infância, sendo, portanto, limitado o uso das equações de regressão para estimar a FCmax na população pediátrica (predição menos precisa e dispersão média entre 5 e 10 bpm). Na adolescência, por volta dos 16 anos, a FCmax começa a declinar a uma taxa de 0,7 ou 0,8 bpm por ano de idade.^177^

Sugere-se a adoção de um valor médio de FC máxima prevista para toda a faixa etária pediátrica (crianças e adolescentes) correspondendo a 197 bpm, e uma FC submáxima prevista de 180 bpm (valor correspondente a −2 desvios padrão).^309 , 310^

Na eventualidade de uso de equações para estimar a FCmax na população pediátrica, considerar que:^309 , 311^

– Equação de Karvonen (FCmax = 220 – idade) geralmente superestima a FCmax.^312 , 313^
– Equação de Tanaka [FCmax = 208 – (0,7 x idade)] pode tanto subestimar quanto superestimar a FCmax, sendo considerada a equação de melhor precisão.^311 , 314 , 315^
– Equação de Nikolaidis [FCmax = 223 – (1,44 x idade)], que foi elaborada para atletas adolescentes, também não se mostrou adequada.^315 , 316^

##### 1.2.6. Monitorização da Pressão Arterial Sistêmica

A medida da PA deve ser executada em todas as fases do TE (pré-teste, esforço e recuperação) por médico devidamente treinado e com experiência em população pediátrica.

A medição manual com utilização de esfigmomanômetro aneroide é a mais utilizada. Medições com equipamentos semiautomáticos e/ou automáticos são possíveis, entretanto podem não fornecer medições precisas em determinadas circunstâncias devido a:^317 , 318^

– Presença de excesso de movimento e vibração que ocorre comumente em crianças mais novas.
– Alguns dispositivos funcionam medindo a PA média e calculando as PA sistólica (PAS) e diastólica (PAD) por meio de algoritmos, que podem apresentar limitações na avaliação da PAD em crianças por não distinguirem as fases IV e V de Korotkoff.^319 , 320^
– A maioria dos equipamentos automáticos não foi validada na população pediátrica para medições em repouso e esforços intensos.^4^

Independentemente do método de medição adotado, utilizar manguito de velcro com dimensões adequadas ao tamanho do braço do paciente. A largura do manguito deve ser de pelo menos 40% da circunferência do braço e cobrir de 80 a 100% do comprimento do braço.^4 , 321^ Recomendamos a utilização das dimensões do manguito preconizadas nas "Diretrizes Brasileiras de Medidas da Pressão Arterial Dentro e Fora do Consultório – 2023" e "Diretrizes Brasileiras de Hipertensão Arterial – 2020" ( Tabela 17 ).^319 , 322^

**Tabela 17:** Dimensões do manguito de acordo com a circunferência do braço

Circunferência do braço	Largura do manguito	Comprimento da bolsa
≤6 cm	3 cm	6 cm
6-15 cm	5 cm	15 cm
16-21 cm	8 cm	21 cm
22-26 cm	10 cm	24 cm
27-34 cm	13 cm	30 cm
35-44 cm	16 cm	38 cm

Adaptado de: Feitosa ADM et al. Brazilian Guidelines for In-office and Out-of-office Blood Pressure Measurement – 2023.^319^

O esfigmomanômetro e os manguitos devem ser limpos e inspecionados regularmente para evitar problemas técnicos que limitem a qualidade e precisão das medidas.^323^

Na medição manual, deve-se observar os sons de Korotkoff considerando que:

– A PAS corresponde ao reaparecimento do fluxo sanguíneo (fase I de Korotkoff).
– A PAD corresponde ao ponto em que o som se abafa (Korotkoff fase IV). A fase IV é usada no lugar da fase V (desaparecimento dos sons) porque, em crianças, na maioria das vezes, os sons de Korotkoff são percebidos até 0 mmHg.

No pré-teste, preferencialmente realizar as medições da PA:

– Em repouso, em posição sentada, com o braço apoiado ao nível do coração.
– Na posição em que a criança/adolescente realizará o esforço físico.

Recomenda-se a medição da PA ao final de cada estágio de protocolo escalonado ou a cada 2 minutos em protocolo de rampa e na recuperação (1, 2, 4 e 6 minutos). Caso haja necessidade, realizar medições por tempo maior na recuperação. Reavaliar sempre que houver discrepâncias ou dúvidas em relação às medições.

Contraindica-se a medição da PA no membro superior com fístula arteriovenosa, esvaziamento ganglionar, trombose, linfedema e/ou coarctação de aorta.

### 2. Estratificação de Risco Pré-TE

Estudos e diretrizes têm trazido novas informações sobre fatores de risco CV na infância, sua relação com aterosclerose e DCV prematuras e também índices e escores de risco específicos para doenças [por exemplo: índice de massa corporal (IMC);^324^ doença de Kawasaki;^37^ lúpus eritematoso sistêmico (LES)^325^]. O risco em crianças e adolescentes também pode ser descrito com base na magnitude do risco de doença aterosclerótica na população geral.^59 , 61 , 326^

No pré-teste, recomenda-se a estratificação de risco para DCV em população pediátrica baseada na presença de doenças ( Tabela 18 ).^59^ Os índices e escores específicos para doenças devem ser utilizados quando julgados pertinentes.

**Tabela 18:** Estratificação de risco da população pediátrica baseada na presença de doenças

Categoria	Condição
**Alto risco**	HF homozigótica, DM1, DM2, doença renal terminal, doença de Kawasaki com aneurismas persistentes, vasculopatia de transplante de órgão sólido, sobrevivente de câncer infantil (receptor de células-tronco).
**Risco moderado**	Obesidade grave, HF heterozigótica, hipertensão confirmada, coarctação de aorta, Lp(a) aumentada, DRC pré-dialítica, EAo, sobrevivente de câncer infantil (radiação torácica).
**Risco leve**	Obesidade, resistência à insulina com comorbidades (dislipidemia, DHGNA, SOP), hipertensão do avental branco, CMH e outras cardiomiopatias, hipertensão pulmonar, condições inflamatórias crônicas (ARJ, LES, doença inflamatória intestinal, HIV), após cirurgia de correção de artérias coronárias anômalas ou transposição dos grandes vasos da base, câncer infantil (somente quimioterapia cardiotóxica), doença de Kawasaki com aneurismas regredidos (EzMax ≥5).

HF: hipercolesterolemia familiar; DM1: diabetes mellitus tipo 1; DM2: diabetes mellitus tipo 2; EAo: estenose aórtica; DRC: doença renal crônica; CMH: cardiomiopatia hipertrófica; ARJ: artrite reumatoide juvenil; Lp(a): lipoproteína (a); DHGNA: doença hepática gordurosa não alcoólica; SOP: síndrome do ovário policístico; LES: lúpus eritematoso sistêmico; HIV: síndrome de imunodeficiência adquirida; EzMax: pontuação z máxima no escore em qualquer momento durante o curso da doença. Adaptado de: de Ferranti et al. Cardiovascular Risk Reduction in High-Risk Pediatric Patients: A Scientific Statement From the American Heart Association.^59^

Em adolescentes, além da estratificação de risco por doença, sugere-se verificar a presença de fatores de risco CV tradicionais: perfil lipídico; tabagismo; história familiar de DAC precoce em parentes de 1° grau (homens ≤55 anos; mulheres ≤65 anos); PA; IMC; glicemia de jejum; histórico de atividade física.^59^

Estudos têm reforçado a relevância de fatores de risco cardiometabólico na população pediátrica: PAS, PAD, circunferência abdominal, IMC, soma das quatro dobras cutâneas, triglicerídeos (TG), colesterol total (CoT), colesterol ligado às lipoproteínas alta densidade (HDL-C), colesterol ligado às lipoproteínas de baixa densidade (LDL-C), relação CoT/HDL-C, glicemia, insulinemia, escore de avaliação do modelo homeostático (HOMA-score) e aptidão cardiorrespiratória (mL/kg/min; estimado ou medido).^60^ A determinação da aptidão cardiorrespiratória aumenta a precisão da quantificação do risco, sendo preconizada principalmente quando presentes outros fatores.^327 – 329^

Na maioria das doenças cardíacas congênitas, existe um maior risco de DCV adquirida precocemente (desde a infância até a fase adulta jovem), vide Tabela 19 . As crianças e adolescentes nessas condições apresentam maior risco de complicações durante o TE/TCPE.^59 , 82 , 83 , 88 , 119 , 330 – 333^

**Tabela 19:** Risco de desenvolvimento de doenças cardiovasculares associadas às doenças cardíacas congênitas

DCC	Doença arterial coronariana	Doença cerebrovascular	Doença vascular periférica	Arritmias cardíacas
**CIA/CIV reparados**	Desconhecido se há aumento de risco.	Aumento do risco se houver *shunt* residual.	Desconhecido se há aumento de risco.	Risco aumentado de taquicardia juncional e arritmia ventricular.
**Valva aórtica bicúspide**	Risco potencial após procedimento de Ross com reimplante de artérias coronárias.	Desconhecido se há aumento de risco.	Aumento do risco relacionado ao aneurisma da aorta.	Risco potencial de arritmia ventricular.
**Coarctação da aorta**	Risco aumentado, relacionado à aterosclerose acelerada e hipertensão tardia.	Aumento do risco relacionado à hipertensão residual ou aneurismas intracranianos.	Aumento do risco relacionado à coarctação residual ou aneurisma da aorta.	Risco de arritmias malignas e morte súbita em seguimento de 10 anos.
**Anomalia de Ebstein**	Desconhecido se há aumento de risco.	Risco aumentado se houver *shunt* interatrial.	Desconhecido se há aumento de risco.	Risco aumentado de taquicardia por reentrada atrioventricular.
**Tetralogia de Fallot**	O aumento do risco pode estar relacionado às anomalias coronarianas.	Risco aumentado se houver *shunt* intracardíaco residual.	Aumento do risco relacionado à dilatação da aorta.	Risco aumentado de taquiarritmias atriais, taquicardia juncional e arritmias ventriculares que podem surgir décadas após a cirurgia.
**TGA**	Aumento do risco relacionado à redução da reserva de fluxo coronariano, espessamento da íntima proximal e anomalias coronarianas.	No *switch* atrial, aumento do risco se houver vazamento residual do reparo.	No *switch* atrial, o aumento do risco pode estar relacionado a cateterizações anteriores. No *switch* arterial, aumento do risco relacionado à dilatação neoaórtica.	Risco de arritmias malignas e morte súbita. Nos pacientes adultos com TGA corrigida, risco aumentado de arritmia ventricular e morte súbita cardíaca.
**Fontan**	Aumento do risco relacionado às anomalias coronarianas.	Aumento do risco se houver fenestração do Fontan.	Aumento do risco relacionado a pressões venosas do Fontan e cateterizações anteriores.	Maior risco de *flutter* atrial nos primeiros 30 dias após cirurgia. No período pós-operatório tardio, são comuns taquicardias atriais por mecanismo de reentrada ( *flutter* e FA, taquicardia reentrante intra-atrial). Risco aumentado de arritmia ventricular.
**CC cianótica**	Risco potencial reduzido.	Aumento do risco relacionado à eritrocitose secundária e síndrome de hiperviscosidade.	Aumento do risco relacionado à eritrocitose secundária e síndrome de hiperviscosidade.	Risco maior de QTc prolongado e de arritmia ventricular.
**Síndrome de Eisenmenger**	Risco potencial reduzido.	Aumento do risco relacionado à eritrocitose secundária e síndrome de hiperviscosidade.	Aumento do risco relacionado à eritrocitose secundária e síndrome de hiperviscosidade.	Risco aumentado de arritmias e morte súbita.

CC: cardiopatia congênita; CIA: comunicação interatrial; CIV: comunicação interventricular; FA: fibrilação atrial; DCC: doença cardíaca congênita; QTc: intervalo QT corrigido; TGA: transposição das grandes artérias. Adaptado de: Ferranti et al. Cardiovascular Risk Reduction in High-Risk Pediatric Patients: A Scientific Statement from the American Heart Association.^59^

### 3. Respostas Clínicas, Hemodinâmicas, Eletrocardiográficas ao Esforço

#### 3.1. Respostas Clínicas

##### 3.1.1. Tolerância ao Esforço

A determinação da tolerância ao esforço permite quantificar a intensidade do esforço físico e os sintomas (cansaço, dispneia, fadiga dos membros inferiores e outros sintomas). A tolerância ao esforço pode ser quantificada de forma objetiva, em qualquer faixa etária, através da potência desenvolvida em watts, duração do esforço ou equivalente metabólico (do inglês, *metabolic equivalent of task* – MET). Quando comparadas aos adultos, as crianças toleram melhor esforço físico de curta duração e são menos fatigáveis durante exercícios dinâmicos.^334^

A quantificação subjetiva pode ser feita através da escala de esforço percebido (EEP), como a escala de Borg, escala de Borg modificada, tabela de avaliação do esforço infantil ilustrada (P-CERT) ou escala OMINI.^335 , 336^ Todas as escalas apresentam limitações relacionadas ao grau de desenvolvimento cognitivo das crianças e dos adolescentes:^337 , 338^

– Crianças de 0 a 3 anos não conseguem avaliar adequadamente seu esforço percebido mesmo durante atividades diárias.
– Dos 4 aos 7 anos, as crianças progressivamente passam a ser capazes de avaliar as alterações sensoriais periféricas dos exercícios, entretanto a quantificação de EEP é menos precisa.^339^
– Dos 8 aos 12 anos, as crianças são capazes de estimar a intensidade de esforço e distinguir a origem das alterações sensoriais referentes às diferentes partes do seu corpo. O tipo de exercício e a EEP utilizada podem influenciar na resposta relatada, principalmente em esforços intensos.^337 , 340 – 342^
– Durante a adolescência, a EEP é útil, entretanto sua relação com a FC atingida é menos pronunciada do que em adultos.^341 , 343 , 344^

A tabela P-CERT foi projetada para avaliar o esforço percebido de crianças de 6 a 9 anos, utilizando escala perceptiva com texto e imagens ilustrativas, melhorando a correlação com a FC atingida, sendo de uso limitado em crianças sem condições de leitura.^345 – 347^

A escala de esforço percebida OMNI utiliza ilustrações de crianças de ambos os sexos realizando exercícios físicos (andar, pedalar, subir escada, nadar etc.) em várias intensidades, facilitando a compreensão e colaboração da criança.^348 – 350^

##### 3.1.2. Aptidão Cardiorrespiratória/Capacidade Funcional

A avaliação da ACR/capacidade funcional em crianças e adolescentes é um importante instrumento clínico para quantificação de sintomas, avaliação prognóstica e avaliação de resposta a tratamentos. Também serve para quantificar as disfunções CV e pulmonares e suas repercussões em crianças portadoras de CC ou adquiridas.^80^

A ACR pode ser avaliada:

– De maneira indireta no TE, através do VO_2_ máximo (VO_2_max) estimado, expresso em METs e seu respectivo percentual em relação ao previsto para a idade.
– De maneira direta no TCPE, através da apresentação do VO_2_ mensurado e seu respectivo percentual em relação ao valor previsto para a idade.^351^

As crianças saudáveis apresentam respostas cardiorrespiratórias e metabólicas diferentes das observadas em adultos. Normalmente, durante o esforço máximo, apresentam respostas cronotrópicas maiores, inotrópicas menores, menor eficiência CV e ventilatória. No entanto, as crianças apresentam maior eficiência metabólica e níveis semelhantes de capacidade de esforço quando comparadas aos adultos.^352 , 353^

A ACR é influenciada por idade, sexo, nível de atividade física diária, obesidade, presença de cardiopatias e pneumopatias, tratamentos instituídos etc.^80 , 91 , 354^

Crianças com CC ou cardiopatias adquiridas frequentemente apresentam comprometimento de sua ACR, independentemente de estarem em pré-operatório, pós-operatório ou acompanhamento de longo prazo. Esse comprometimento pode estar associado à cardiopatia primária, aos tratamentos dessa cardiopatia, à hipoatividade/sedentarismo e fatores comportamentais (tal como a superproteção dos pais). Adolescentes com CC podem ter conceito errôneo sobre níveis seguros e desejáveis de atividade física, mantendo o círculo vicioso de sedentarismo.^355 – 357^

**Figura 1:**
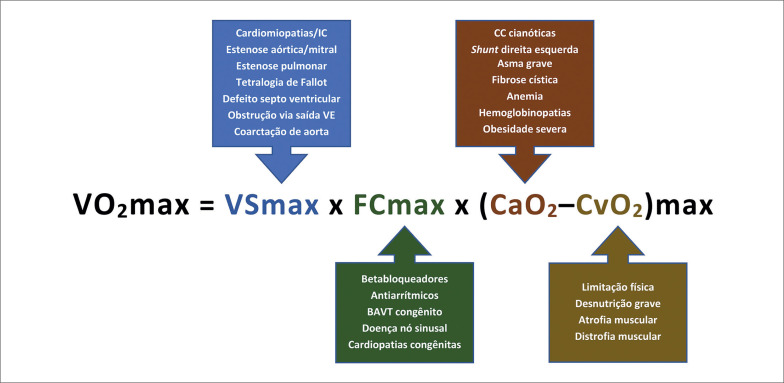
Doenças pediátricas que afetam componentes específicos da equação de Fick, comprometendo a aptidão cardiorrespiratória. VSmax: volume sistólico máximo durante o exercício; FCmax: frequência cardíaca máxima; (CaO_2_-CvO_2_)max: diferença arteriovenosa do conteúdo de oxigênio; CaO_2_: conteúdo arterial de oxigênio; CvO_2_: conteúdo venoso de oxigênio; CC: cardiopatia congênita; BAVT: bloqueio atrioventricular total; IC: insuficiência cardíaca; VE: ventrículo esquerdo. Adaptado de: Bar-Or O. Pathophysiological Factors Which Limit the Exercise Capacity of the Sick Child.^355^

A Figura 1 apresenta as doenças pediátricas, fatores fisiopatológicos e situações clínicas (comorbidades, tratamentos etc.) que comprometem os componentes específicos da equação de Fick utilizada para a determinação da ACR (VO_2_max).^355^

##### 3.1.3. Sintomas, Ectoscopia e Ausculta

A observação clínica de sintomas, a ectoscopia e o exame físico durante o TE/TCPE são fundamentais nas crianças e adolescentes ( Quadro 3 ), pois:

– Crianças mais novas apresentam limitações quanto ao esforço percebido e à interpretação de suas alterações sensoriais periféricas.^337 , 338 , 358^
– A queixa de cansaço constitui a principal razão de interrupção do esforço na população pediátrica, requerendo correlação com os achados físicos (padrão respiratório, sinais de tiragem intercostal, dispneia etc.), inclusive para a determinação da tolerância ao esforço e classe funcional.
– A ocorrência de dor torácica esforço-induzida requer avaliação e caracterização pormenorizadas, auxiliando no diagnóstico diferencial de possíveis origens não cardíacas (exemplo: asma esforço-induzida). A dor anginosa típica está geralmente associada à origem anômala de coronárias, estenose aórtica e doença de Kawasaki.^15 , 35 , 37 , 359 , 360^
– No pré-teste, especialmente em crianças com CC e valvopatias, os pulsos femorais e periféricos devem ser palpados para identificar alterações de amplitude, atraso radiofemoral e possíveis obstruções.^361 , 362^
– A ausculta cardíaca realizada logo após o pico do esforço permitirá avaliar a ocorrência de novos sopros cardíacos ou modificações do padrão de sopros da ausculta pré-teste. Frequentemente, crianças e adolescentes têm B3 audível na ausculta basal, e caso surja durante o exercício, geralmente é considerada adaptação fisiológica sem correlação com cardiopatia estrutural.^363 – 365^ A ocorrência de sopro sistólico e/ou desdobramento de B3 está frequentemente associada às CC e valvopatias.^362 , 366^
– Quanto à ausculta do aparelho respiratório, a ocorrência de roncos e sibilos pulmonares pode indicar broncoespasmo esforço-induzido associado à asma.^367^ A ausculta de estridor inspiratório e/ou sibilos na parte superior do tórax e também na região da traqueia pode auxiliar no diagnóstico de obstrução laríngea esforço-induzida. Nesses casos, recomenda-se a visualização das estruturas laríngeas através de laringoscopia, que contribui para o diagnóstico do tipo de obstrução laríngea e para a condução da crise obstrutiva.^368 , 369^

Particularidades dos sintomas, ectoscopia e ausculta durante o TE e TCPE na população pediátrica:^177 , 361 , 362 , 370^

– Crianças e adolescentes sedentários podem apresentar aumento desproporcional da frequência respiratória (FR) em relação à intensidade de esforço e dispneia. O exame físico geralmente é normal, sem sinais de causas restritivas ou obstrutivas para a dispneia.^286 , 371^
– Crianças com anormalidades da parede torácica (por exemplo: escoliose, *pectus excavatum* e *pectus carinatum* ) podem apresentar dispneia esforço-induzida e, dependendo da gravidade da deformidade, processo restritivo.^372 – 375^
– Na distrofia muscular e outras miopatias, é comum a ocorrência de dispneia e baixa tolerância ao esforço associada à doença pulmonar restritiva e ao comprometimento da musculatura respiratória.^203 – 206^
– Crianças com cardiomiopatia hipertrófica obstrutiva podem apresentar dor torácica esforço-induzida associada à isquemia miocárdica. Geralmente, na ausculta cardíaca realizada no pré-teste, observa-se um sopro cardíaco mais intenso na posição ortostática ou após manobra de Valsalva.^13^
– Crianças com hipertensão arterial pulmonar (HAP) podem apresentar dor torácica esforço-induzida, sendo esse o sintoma inicial mais comum da HAP na forma idiopática.^196 , 376 , 377^
– Nas cardiomiopatias dilatadas, pode ocorrer dor torácica geralmente associada à fadiga intensa ao esforço. Também é necessária atenção quanto à possibilidade de dessaturação e ocorrência de cianose.^378 , 379^
– A estenose pulmonar grave pode produzir dor torácica opressiva associada à isquemia miocárdica.^87 , 380 , 381^
– As estenoses valvar aórtica, supra-aórtica e subaórtica podem causar dor torácica esforço-induzida, tontura e fadiga. Essas crianças geralmente têm sopro de ejeção rude, às vezes acompanhado por clique de ejeção em válvula aórtica bicúspide.^134 , 382 , 383^
– Em crianças, as taquicardias supraventriculares e ventriculares geralmente se apresentam como palpitação, que pode ser exacerbada pelo esforço e, também, cursar com dor torácica breve e aguda.^361 , 370 , 384^

#### 3.2. Respostas Hemodinâmicas

##### 3.2.1. Frequência Cardíaca

###### 3.2.1.1. Frequência Cardíaca de Repouso

A FC de repouso, em condição basal, diminui com o aumento da idade e varia de uma média de 85 bpm aos 4 anos para 60 bpm aos 16 anos. Essa redução da FC é diretamente relacionada ao declínio da taxa metabólica da criança com o envelhecer.^385 – 387^ Os valores de FC de repouso (mínima e máxima) devem ser correlacionados aos previstos para as faixas etárias pediátricas.

Na população pediátrica, a bradicardia em repouso geralmente é observada em atletas altamente treinados, secundária aos medicamentos (particularmente betabloqueadores), no hipotireoidismo e na disfunção do nó sinusal.^370 , 388 , 389^ A taquicardia sinusal em repouso geralmente ocorre em: condições de calor/clima quente; hipertireoidismo; anemia; obesidade; ansiedade pré-teste; taquicardia sinusal inapropriada, raramente estando associada à taquiarritmia supraventricular.^390 – 393^

Em crianças com cardiomiopatia dilatada, a FC de repouso mais elevada associa-se ao risco de morte e à necessidade de transplante cardíaco. O controle medicamentoso da FC associou-se à melhora da função ventricular e evolução da doença.^394 – 396^

###### 3.2.1.2. Resposta Cronotrópica

A avaliação da resposta cronotrópica é fundamental no esforço e na fase de recuperação. Em crianças e adolescentes, durante um TE progressivo, a FC aumenta linear e proporcionalmente ao VO_2_, desde os níveis basais até a FCpico. A FC máxima geralmente não é afetada pelo nível de aptidão cardiorrespiratória ou sexo, permanecendo constante ao longo dos anos pediátricos. Entretanto, em TE seriado, à medida que a criança cresce, observa-se redução da FC submáxima em determinada carga de trabalho.^8 , 177 , 397 , 398^

Na recuperação, normalmente ocorre uma queda progressiva da FC com retorno ao padrão basal até o 6° minuto. No 1 ° minuto da recuperação, adolescentes aparentemente saudáveis do sexo masculino apresentam redução de ≈44 bpm e do sexo feminino ≈36 bpm. Crianças do sexo masculino também costumam apresentar maior redução da FC no 1 ° minuto da recuperação do que as do sexo feminino.^302 , 352 , 399^ Crianças com excesso de peso e/ou com menor resistência ao esforço geralmente apresentam recuperação mais lenta da FC no 1 ° minuto.^400 , 401^

Pacientes com disfunção do nó sinusal (DNS) ou no pós-operatório de CC podem não aumentar adequadamente sua FC com o esforço e terem FCpico menor. O aumento lento na FC à medida que a intensidade do trabalho aumenta é normalmente observado em jovens atletas treinados. A resposta cronotrópica deprimida na população pediátrica geralmente ocorre no elevado grau de tônus vagal, DNS, pós-operatório de CC, uso de medicamentos como betabloqueadores, bloqueadores dos canais de cálcio e antiarrítmicos.^177 , 389 , 400^

A Tabela 20 apresenta os termos referentes ao comportamento da FC no TE/TCPE em população pediátrica, bem como os respectivos critérios e possíveis interpretações.

**Tabela 20:** Definições, critérios e interpretação do comportamento da FC no TE/TCPE em crianças e adolescentes

Termo		Critérios	Interpretação
**Comportamento da FC no ECG de repouso**			
	Comportamento normal da FC	ECG de repouso com FC entre a mínima e máxima prevista para a faixa etária ( Tabela 24 ).	Crianças e adolescentes em ritmo sinusal.
	Bradicardia sinusal em repouso	ECG de repouso com FC abaixo da mínima esperada para a faixa etária ( Tabela 24 ).	Comum em adolescentes atletas e vagotônicos, assintomáticos. Caso secundária à utilização de betabloqueador ou antiarrítmico, referir essa interferência no laudo. Em pacientes que não utilizam medicações inotrópicas negativas, avaliar possibilidade de doença do nó sinusal ou outras causas secundárias (exemplo: hipotireoidismo). Afastar BAV de segundo grau e BAV avançado.
	Taquicardia sinusal em repouso	ECG de repouso com FC acima da máxima esperada para a faixa etária ( Tabela 24 ).	Usualmente encontrada em pacientes obesos, com elevado grau de ansiedade, no hipertireoidismo, na anemia e após ingestão excessiva de cafeína ou álcool.
**Comportamento da FC ao esforço**			
	Resposta cronotrópica normal	Atingir pelo menos a FC submáxima prevista de 180 bpm (valor correspondente a −2 desvios padrão) entre 8 e 12 minutos de esforço. * ou Na eventualidade de uso de equações para estimar a FCmax (equação de Tanaka ou equação de Karvonen), atingir ≥80% da FCmax prevista entre 8 e 12 minutos de esforço. **	Os valores médios previstos para toda a faixa etária pediátrica (crianças e adolescentes) da FC máxima é 197 bpm e da FC submáxima de 180 bpm, são contantes, não sendo afetados pelo nível de ACR, sexo e idade.^8 , 177 , 397 , 398 , 402 , 403^
	Queda da FC intraesforço	Queda da FC com a progressão do esforço associada a sinais e sintomas sugestivos de baixo débito cardíaco (fadiga extrema, tontura, queda de PAS etc.).	Critério de interrupção do esforço.^7 , 11^
	Resposta cronotrópica deprimida ou incompetência cronotrópica ***	FC máxima atingida <175 bpm (TE em esteira) e <170 bpm (TE em bicicleta) ** ou FC máxima <80% da FC prevista para idade,^404^ ou < percentil 2,5 do índice cronotrópico para idade e sexo,^405^ ou índice cronotrópico <0,80.	Relativamente comum em crianças após correção cirúrgica de CC. Associa-se a redução da tolerância ao esforço, pior ACR e maior morbidade em cardiopatas.^177 , 405 – 409^ Em pacientes com Fontan, associa-se à disfunção do nó sinusal.^401^
	Platô da FC intraesforço	Manutenção assintomática da FC (durante 1 a 2 estágios) e subsequente incremento com a continuidade do esforço.	Pode ocorrer em crianças aparentemente saudáveis, não tendo significado clínico.
**Comportamento da FC na recuperação**			
	Comportamento normal da FC na recuperação	Na recuperação, normalmente ocorre uma queda progressiva da FC com retorno ao padrão basal até o 6 ° minuto.	Quando em ritmo sinusal. Crianças do sexo masculino costumam apresentar maior redução da FC no 1 ° minuto da recuperação do que as do sexo feminino.^302 , 352 , 399^
	Recuperação lenta da FC (pós-esforço)	Avaliado através do ΔFC 1 ° min = FCmax esforço – FC no 1 ° minuto recuperação.	Comum após cirurgia de Fontan.^410^ Em crianças com CC, pode estar associada à incompetência cronotrópica. Pode ser explicada pela reativação lenta da atividade vagal, retirada tardia da atividade simpática e/ou baixa ACR.^410 – 415^
		Não há consenso sobre o valor do ΔFC, geralmente considerado anormal se ≤35 bpm.^302 , 352^	
	Queda súbita e acentuada da FC na recuperação	Geralmente assintomática. Não há consenso sobre o valor de referência, mas geralmente corresponde a queda >55 bpm no primeiro minuto da recuperação.	Achado comum em crianças mais novas e atletas pediátricos.^416 , 417^

ECG: eletrocardiograma; FC: frequência cardíaca; bpm: batimentos por minuto; PAS: pressão arterial sistólica; BAV: bloqueio atrioventricular; CC: cardiopatia congênita; TE: teste ergométrico; ACR: aptidão cardiorrespiratória.

* FC submáxima prevista para toda a faixa etária pediátrica (crianças e adolescentes).

** Os valores de FC máxima podem apresentar variação individual significativa entre 5 e 10 bpm.

*** Descrever o uso de medicamentos que possam afetar o comportamento da FC.

##### 3.2.2. Resposta da Pressão Arterial

O comportamento da PA é uma variável importante do TE/TCPE em população pediátrica, por refletir as adaptações do débito cardíaco e resistência vascular periférica com o esforço.^418 , 419^

Em relação à avaliação da PA no pré-teste, em repouso, recomenda-se a adoção dos critérios de PA da Tabela 21 , baseados na Diretriz Brasileira de Hipertensão, que consideram a idade, o sexo e o percentil de estatura das crianças e adolescentes (consultar Anexos 2 e 3 ).^322^

**Tabela 21:** Definição da pressão arterial em repouso no TE/TCPE de acordo com a faixa etária^322^

Crianças de 1 a <13 anos de idade	Crianças com idade ≥13 anos
**PA em repouso normal:** <P90 para idade, sexo e estatura.	**PA em repouso normal:** <120/<80 mmHg.
**PA em repouso elevada:** – PAS ≥P90 e/ou PAD ≥P95 para idade, sexo e estatura.	**PA em repouso elevada:** – PA ≥120/≥80 mmHg.

PA: pressão arterial; P: percentil; PAS: pressão arterial sistólica; PAD: pressão arterial diastólica. Consultar os percentis nos Anexos 2 e 3 .

Em relação ao comportamento da PA com o esforço, normalmente observa-se um aumento progressivo da PAS, que contribui para o aumento do débito cardíaco, cuja magnitude está diretamente relacionada à intensidade do esforço. Os valores de PAS atingidos no pico do esforço (PASpico), mesmo que não estejam associados à exaustão física (esforço máximo), também são proporcionais à idade (quanto maior a idade, maior a PASpico), à ASC (quanto maior a área, maior a PASpico) e à PAS no pré-teste. A PAS máxima (PASmax) é considerada a PAS medida no esforço máximo. Entretanto, ocasionalmente, pacientes pediátricos aparentemente saudáveis podem apresentar apenas discreto aumento da PAS com o esforço.^403 , 420^

A ASC tem sido utilizada como critério para definição do percentil de normalidade e avaliação do comportamento da PAS. Por exemplo, crianças do mesmo sexo e idade com ASC diferentes terão comportamento distintos da PASmax: a criança com ASC de 1,25 m^2^ apresentará PASmax de 140 mmHg, enquanto outra com ASC de 1,75 m^2^ atingirá 160 mmHg.^421 , 422^

A PASmax/PASpico, ou quando medida imediatamente após a interrupção do esforço, são consideradas padrão de avaliação da capacidade inotrópica cardíaca. Alterações do comportamento da PA são úteis para diagnóstico, definição terapêutica e estratificação de risco em crianças e adolescentes com CC, valvopatias, IC ou suspeita de hipertensão arterial sistêmica (HAS).^53 , 418 , 419^

No período de recuperação, observa-se um declínio progressivo da PAS com retorno aos níveis de repouso em cerca de 6 minutos. Após esse período, a PAS geralmente permanece mais baixa do que os níveis pré-esforço por várias horas.^423^

A PAD normalmente permanece constante com o esforço, independentemente de idade e sexo, devido à vasodilatação esforço-induzida. O limite de variação da PAD situa-se em torno de ±10 mmHg. Pode ocorrer queda discreta da PAD em crianças aparentemente saudáveis.^424^

Estudo em adolescentes brasileiros normotensos sobre o comportamento da PA no TE demonstrou aumento da PAS e queda da PAD durante o esforço em todas as faixas etárias e ambos os sexos.^425^ Outros estudos demonstraram que o incremento da PAS e a resposta cronotrópica foram significativamente menores em crianças portadoras de CC complexas e cardiomiopatia dilatada.^155 , 395 , 426^

A ausência de aumento adequado da PAS com o esforço pode ser indicativa de possível disfunção cardíaca. A queda persistente da PAS com a progressão do esforço pode estar relacionada à IC ou obstrução de via de saída do ventrículo esquerdo (exemplos: estenose aórtica grave, cardiomiopatia hipertrófica assimétrica).

Estudos nacionais e internacionais buscaram avaliar o comportamento da PA em crianças e adolescentes submetidas ao TE e definir valores de referência e equações preditivas desse comportamento. Devido à grande heterogeneidade das populações estudadas e dos resultados obtidos, ainda não foi possível estabelecer um critério único de normalidade para o comportamento da PA no TE.^38 , 305 , 403 , 419 , 422 , 425 , 427 , 428^

**Figura 2:**
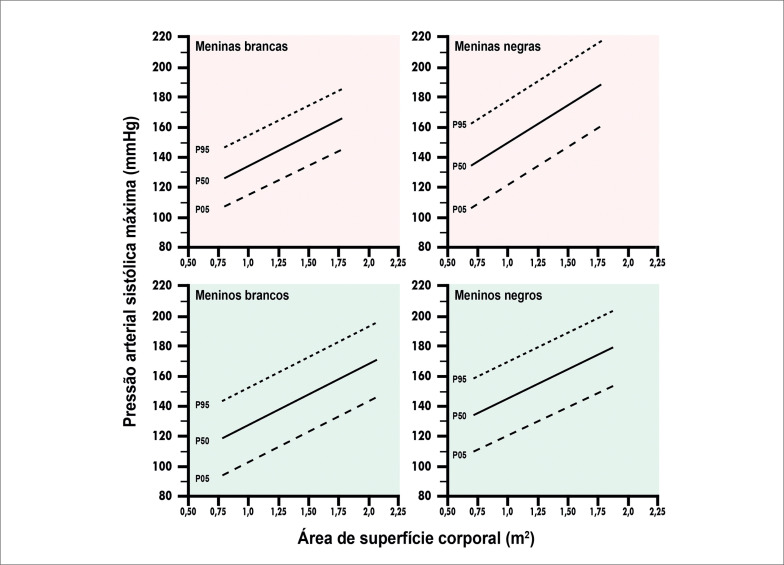
Nomogramas de comportamento da pressão arterial sistólica máxima em relação ao sexo, raça e área de superfície corporal. A linha contínua representa o percentil 50 (P50) da pressão arterial sistólica. A linha tracejada superior representa o percentil 95 (P95), enquanto a linha tracejada inferior representa o percentil 5 (P05). Área de superfície corporal (m^2^). Adaptado de: Alpert BS et al. Responses to Ergometer Exercise in a Healthy Biracial Population of Children.^422^

Para a avaliação da PASmax, sugerimos a utilização de:

– Equação de predição baseada em sexo e idade ( Tabela 22 ) para a faixa etária de 7 a 17 anos; ou
– Normograma baseado no sexo e ASC ( Figura 2 ) para a faixa etária de 6 a 15 anos.

**Tabela 22:** Valores preditos da PASpico baseados em modelo de regressão linear para idade e sexo

PASpico (mmHg)						
Idade	Meninos			Meninas		
	P90	P95	Média prevista	P90	P95	Média prevista
**7**	161	167	132	169	174	142
**8**	166	171	136	170	175	143
**9**	170	176	141	172	177	145
**10**	175	180	145	173	178	146
**11**	179	185	150	174	179	147
**12**	184	189	154	176	181	149
**13**	188	194	159	177	182	150
**14**	192	198	163	178	184	151
**15**	197	203	168	180	185	153
**16**	201	207	172	181	186	154
**17**	206	212	177	183	188	156
**Fórmulas** *	PAS P95 = 135,40 + 4,48 x idade PAS P90 = 129,75 + 4,48 x idade PAS média prevista = 100,39 + 4,48 x idade			PAS P95 = 164,39 + 1,37 x idade PAS P90 = 159,21 + 1,37 x idade PAS média prevista = 132,27 + 1,37 x idade		

P: percentil; PAS: pressão arterial sistólica.

* Idade em anos. Adaptado de: Sasaki T et al. Blood Pressure Response to Treadmill Cardiopulmonary Exercise Test in Children with Normal Cardiac Anatomy and Function.^424^

Os critérios recomendados para a avaliação e descrição do comportamento da PA no TE em crianças e adolescentes são apresentados na Tabela 23 .

**Tabela 23:** Comportamento da pressão arterial ao TE/TCPE em crianças e adolescentes

Termo	Critérios **
**PA normal em repouso**	– 1 a <13 anos de idade: PA em repouso normal: <P90 para idade, sexo e estatura. – ≥13 anos: PA em repouso normal: <120/<80 mmHg ( Tabela 21 ). *
**PA elevada em repouso**	– 1 a <13 anos de idade: PAS ≥P90 e/ou PAD ≥P95 para idade, sexo e estatura. – ≥13 anos: PA ≥120/≥80 mmHg ( Tabela 21 ).*
**Resposta normal da PA ao esforço e recuperação**	PA normal em repouso. Esforço: *** – PAS ≤P95 (baseado em tabelas de referência ajustadas para idade e sexo), ou – PAS ≤P90 na faixa de 7 a 17 anos ( Tabela 22 ), ou – PAS menor que os valores máximos previstos para idade e sexo: 12 a 13 anos = ♀ e ♂ 172; 14 a 15 anos = ♀174,7/♂177,3; 16 a 17 anos = ♀ 178,5/♂ 201,3 mmHg.^38 , 41^ – Variação máxima da PAD de ±10 mmHg (♀ e ♂). Recuperação: queda da PAS gradativa até retorno aos padrões de repouso em torno dos 6 minutos.
**Hipertensão pré-teste com resposta normal da PA ao esforço**	PA elevada em repouso. Esforço: *** – PAS ≤P95 (baseado em tabelas de referência ajustadas para idade e sexo), ou – PAS ≤P90 na faixa de 7 a17 anos ( Tabela 22 ), ou – PAS menor que os valores máximos previstos para idade e sexo: 12 a 13 anos = ♀ e ♂ 172; 14 a 15 anos = ♀174,7/♂177,3; 16 a 17 anos = ♀178,5/♂201,3 mmHg.^38 , 41^ – Variação máxima da PAD de ±10 mmHg (♀ e ♂).
**Resposta hipertensiva/exagerada ao esforço**	PA em repouso poderá estar normal ou elevada. Esforço: *** – PAS >P95 (baseado em tabelas de referência da PA ao esforço ajustadas para idade e sexo), ou – PAS >P90 na faixa de 7 a 17 anos ( Tabela 22 ), ou – PAS ≥ que os valores máximos previstos para idade e sexo: 12 a 13 anos = ♀ e ♂ 172; 14 a 15 anos = ♀ 174,7/♂ 177,3; 16 a 17 anos = ♀ 178,5/♂ 201,3 mmHg.^38 , 41^ – Elevação de PAD ≥15 mmHg (♀ e ♂).
**Hipotensão/queda da PA intraesforço** ****	PAS no esforço com valor inferior ao da PAS de repouso,^8^ ou PAS com aumento inicial no esforço e subsequente queda ≥20 mmHg.
**Reserva pressórica deprimida**	ΔPAS = PASpico esforço – PAS repouso.^424^ ♂: idade 7 a 11 anos = ΔPAS <10 mmHg; idade 12 a 17 anos = ΔPAS <20 mmHg; ♀: ΔPAS <10 mmHg (7 a 17 anos).
**Resposta normal da PA na recuperação**	– PAS apresenta redução progressiva. Aos 6 minutos, a PAS e a PAD retornam aos valores de repouso.

PA: pressão arterial; PAS: pressão arterial sistólica; PAD: pressão arterial diastólica; ΔPAS: variação da PAS com o esforço; P: percentil; ♂ = masculino; ♀ = feminino.

* Consultar os valores da PA em relação aos percentis nos Anexos 2 e 3 .

** Descrever se a resposta da PA é em vigência de uso ou não de anti-hipertensivos.

*** Relatar a tabela de referência e o valor do percentil utilizados.

**** Raramente crianças e adolescentes sem doença cardíaca clinicamente significativa apresentarão hipotensão esforço-induzida; pode estar relacionada à desidratação, dose inadequada de anti-hipertensivo ou esforço extenuante prolongado.

Particularidades da resposta da PA no TE em população pediátrica:

– **Hipertensão do avental branco:** geralmente apresentam resposta exagerada da PAS com o esforço, podendo representar um estado pré-hipertensivo.^46^
– **Risco futuro de hipertensão:** há evidências de que a resposta exagerada da PA ao esforço em crianças e adolescentes aparentemente saudáveis seja preditora de HAS futura.^429 , 430^
– **Associação com** **HVE:** resposta hipertensiva da PAS e/ou da PAD em crianças e adolescentes normotensos (principalmente naqueles com história familiar de hipertensão) se correlacionam com grau de HVE.^47 , 431 – 433^
– **Estenose aórtica:** à medida que a estenose da valva aórtica (subvalvar ou supravalvar) se torna mais grave, o aumento da PAS ao esforço é significativamente menor. Na estenose grave, a elevação da PAS geralmente situa-se entre 10 e 20 mmHg.^134 , 383 , 434 , 435^ Raramente ocorre queda da PAS no esforço, que se relaciona ao comprometimento da função ventricular (gradiente >70 mmHg).^436^ O ΔPAS ao esforço ≥35 mmHg demonstrou melhor prognóstico.^437^
– **Cardiomiopatia hipertrófica** (CMH) **:** o ΔPAS ao esforço <20 mmHg ou queda da PAS >20 mmHg em crianças e adolescentes estão associados ao maior risco de morte cardíaca.^155 , 438^
– **Coarctação da aorta (** CoAo **):** após correção cirúrgica bem-sucedida, até um terço dos pacientes permanece ou se torna hipertenso. A reposta hipertensiva ao esforço é comum, mesmo na ausência de obstrução residual significativa.^49 , 439 , 440^
– **Atletas:** a elevação da PAS em crianças e adolescentes fisicamente ativos costuma ser mais lenta do que em sedentários e obesos.^441^ Adolescentes aparentemente saudáveis e altamente treinados geralmente apresentam ΔPAS maior do que os não treinados. Equações para predição da PAS em atletas (entre 10 e 18 anos) em qualquer momento do TE:^442^

**Sexo masculino:** PAS esforço (mmHg) = −1,92 × idade + 0,55 × carga de trabalho + 120,84

**Sexo feminino:** PAS esforço (mmHg) = −0,88 × idade + 0,48 × carga de trabalho + 111,22

Observação: idade em anos; carga de trabalho em watts (W).

##### 3.2.3. Duplo-Produto

O duplo-produto (DP) expressa o VO_2_ miocárdico, sendo calculado através da multiplicação da FC pela PAS a qualquer momento do TE/TCPE:


DP(bpm.mmHg)= FC x PAS

Em crianças e adolescentes, o DP em repouso geralmente é influenciado pelo sexo (menor no sexo feminino), pelos índices antropométricos (IMC, relação cintura quadril e percentual de gordura corporal) e nível de ACR. O Anexo 4 apresenta informações sobre valores do DP em repouso e no pico do esforço de população pediátrica aparentemente saudável, com IC e com CoAo.^428 , 443 , 444^

Comportamento do DP:

– Em crianças aparentemente saudáveis, correlacionou-se positivamente com a idade.
– No segundo estágio de protocolo incremental e no pico do esforço, é útil na previsão de hipertensão sistólica na adolescência, independentemente da PAS de repouso e dos fatores de risco CV convencionais.^445^
– Pacientes com doença de Kawasaki apresentam DP máximo significativamente menor.^15^

#### 3.3. Respostas Eletrocardiográficas

Para a adequada análise, descrição e interpretação das respostas eletrocardiográficas na população pediátrica, recomenda-se:

– Verificar o posicionamento e a correta fixação dos eletrodos para minimizar erros e artefatos.^446 , 447^
– Considerar os efeitos dos filtros de ECG que estiverem sendo utilizados (alta, média, baixa frequência) para a estabilização da linha de base, redução de artefatos musculares e de rede elétrica. Em adolescentes, utilizar filtros de alta frequência de no mínimo 150 Hz e, em crianças, até 250 Hz. Filtros com frequências mais baixas podem interferir na captação das espículas de MP.^308 , 448 , 449^
– Sugere-se utilizar sistemas de medições automatizados para os intervalos, durações e amplitudes das ondas e segmentos do ECG adaptados e validados para a população pediátrica.^11 , 188 , 450^
– Utilizar a normatização para a emissão de laudos eletrocardiográficos da Diretriz da Sociedade Brasileira de Cardiologia sobre a Análise e Emissão de Laudos Eletrocardiográficos – 2022 e os valores de referência dos principais parâmetros eletrocardiográficos ajustados às diversas faixas etárias da população pediátrica ( Tabela 24 ).^308 , 451^
– Revisar os valores das medidas automatizadas de modo a afastar erros por possíveis interferências, artefatos ou anormalidades do traçado subjacente.^452 , 453^
– Descrever de maneira detalhada e contextualizada o registro eletrocardiográfico para a população pediátrica e suas doenças.

**Tabela 24:** Valores de referência dos principais parâmetros eletrocardiográficos em repouso de crianças e adolescentes

	1-3 anos		3-5 anos		5-8 anos		8-12 anos		12-16 anos	
	Mín	Máx	Mín	Máx	Mín	Máx	Mín	Máx	Mín	Máx
FC (bpm)	89	152	73	137	65	133	62	130	60	120
P Dll amplitude (mV)	0,07	0,25	0,03	0,25	0,04	0,25	0,03	0,25	0,03	0,25
P duração (ms)	63	113	67	102	73	108	78	117	78	122
SâP	-12	19	-13	69	-54	72	-17	76	-24	76
PRi Dll (ms)	80	150	80	160	90	160	90	170	90	180
SâQRS	7	102	6	104	10	139	6	116	9	128
QRS V5 (ms)	30	80	30	70	30	80	40	90	40	90
Q aVF (mV)	0,00	0,32	0,00	0,29	0,00	0,25	0,00	0,27	0,00	0,24
Q V1 (mV)	0,00	0,00	0,00	0,00	0,00	0,00	0,00	0,00	0,00	0,00
Q V6 (mV)	0,00	0,28	0,01	0,33	0,01	0,46	0,01	0,28	0,00	0,29
R V1 (mV)	0,20	1,80	0,10	1,80	0,10	1,40	0,10	1,20	0,10	1,00
R V6 (mV)	0,60	2,30	0,80	2,50	0,80	2,60	0,90	2,50	0,70	2,30
S V1 (mV)	0,10	2,10	0,20	2,20	0,30	2,30	0,30	2,50	0,30	2,20
S V6 (mV)	0,00	0,70	0,00	0,60	0,00	0,40	0,00	0,40	0,00	0,40
T V1 (mV)	-0,60	-0,10	-0,60	0,00	-0,50	0,20	-0,40	0,30	-0,40	0,30
T V6(mV)	0,10	0,60	0,15	0,70	0,20	0,75	0,20	0,70	0,10	0,70
R/S V1	0,10	4,30	0,03	2,70	0,02	2,00	0,02	1,90	0,02	1,80
R/S V6	0,30	27,00	0,60	30,00	0,90	30,00	1,50	33,00	1,40	39,00
QTc (ms)	381	455	377	448	365	447	365	447	362	449

Mín: mínima; Máx: máxima; ms: milissegundos; mV: milivolts; FC: frequência cardíaca; bpm: batimentos por minuto; PRi: intervalo PR; QTc: intervalo QT corrigido; SâP: eixo da onda P; SâQRS: eixo dos complexos QRS. Adaptado de: Samesima N et al. Diretriz da Sociedade Brasileira de Cardiologia sobre a Análise e Emissão de Laudos Eletrocardiográficos – 2022.^308^

Quanto ao ECG na população pediátrica:^451^

– Deve ser avaliado de acordo com a idade da criança. Crianças menores apresentam padrão precordial com domínio do VD e, com a progressão dos anos, assume padrão do ECG do adulto com predomínio fisiológico do VE.
– Nas CC, o ECG reflete as alterações anatômicas e suas repercussões hemodinâmicas sobre as câmaras cardíacas.
– As deformidades torácicas, má posição cardíaca e/ou alterações do ritmo cardíaco limitam a interpretação.

##### 3.3.1. ECG de Repouso

Existem alterações no ECG de repouso em crianças e adolescentes que estão associadas a condições patológicas, maior risco de complicações durante o TE/TCPE e risco de morte súbita ( Tabela 25 ). Essas alterações podem interferir na interpretação de alterações esforço-induzidas.

**Tabela 25:** Alterações no ECG de repouso em crianças e adolescentes associadas a condições patológicas, maior risco de complicações ao TE/TCPE e risco de morte súbita^64 , 462^

Componente do ECG	Alteração	Associação
**Onda P**	Aumento, sobrecarga do átrio esquerdo: porção negativa da onda P na derivação V1 com profundidade ≥0,1 mV e duração ≥0,04 s.	Valvopatia; CC.
	Aumento, sobrecarga do átrio direito (SAD): onda P pontiaguda nas derivações II e III ou V1 com amplitude ≥0,25 mV.	Valvopatia; CC.
**Complexo QRS**	Desvio do eixo do plano frontal: para direita ≥ +120° ou esquerda −30° a -90°.	CC; CMD; distúrbio da condução intraventricular.
	Aumento de amplitudes: amplitude da onda R ou S em uma das derivações dos membros ≥2 mV, onda S na derivação V1 ou V2 ≥3 mV, ou onda R na derivação V5 ou V6 ≥3 mV.	CC; HVE; valvopatia.
	Ondas Q anormais (duração ≥0,04 s ou ≥25% da altura da onda R subsequente) ou padrão QS em duas ou mais derivações.	CMH; CMD; VENC; miocardite; IAM prévio.
	Bloqueio de ramo direito ou esquerdo com duração de QRS ≥0,12 s.	CMD; CMH; VENC; sarcoidose; miocardite; CC.
	Onda épsilon (deflexão positiva no final do QRS nas derivações V1 e V2).	CAVD.
**Segmento ST, Onda T e QTc**	Infradesnivelamento do segmento ST.	CC; CMH; CMD; VENC; CAVD; miocardite.
	Inversão ou achatamento da onda T em duas ou mais derivações (derivações laterais).	CMH; CMD; VENC; CAVD; miocardite.
	Prolongamento do intervalo QT corrigido pela frequência cardíaca >0,44 s em no sexo masculino e >0,46 s no sexo feminino.	Síndrome do QT longo.
**Anormalidades de ritmo e condução**	Batimentos ventriculares prematuros ou arritmias ventriculares mais graves.	CMH; CMD; VENC; CAVD; miocardite; sarcoidose.
	Taquicardias supraventriculares, *flutter* atrial ou fibrilação atrial.	Doença miocárdica ou elétrica.
	Intervalo PR curto (<0,12 s) com ou sem onda "delta".	WPW; síndrome do PR curto.
	Bradicardia sinusal com frequência cardíaca de repouso ≤40 bpm.	CC; doença do nó sinusal; doença miocárdica ou elétrica.
	Bloqueio atrioventricular de primeiro (PR ≥0,21 s), segundo ou terceiro grau.	Doença miocárdica ou elétrica; bloqueio atrioventricular congênito.

BRE: bloqueio de ramo esquerdo; CAVD: cardiomiopatia arritmogênica do ventrículo direito; IAM: infarto agudo do miocárdio; CMD: cardiomiopatia dilatada; CMH: cardiomiopatia hipertrófica; VENC: ventrículo esquerdo não compactado; CC: cardiomiopatia congênita; WPW: Síndrome de Wolff-Parkinson-White; HVE: hipertrofia ventricular esquerda; QTc: intervalo QT corrigido.

O padrão de repolarização precoce (PRP) é comum na população pediátrica, devendo ser contextualizado:^454 , 455^

– O padrão ascendente difuso é comum entre os jovens, naqueles de etnia europeia, sendo encontrado igualmente em ambos os sexos e sem correlação aparente com arritmias atriais ou ventriculares.^456^
– Atletas pediátricos costumam apresentar ponto J com entalhe e segmento ST rapidamente ascendente e côncavo, principalmente nas derivações ínfero-laterais. Outras alterações: bradicardia sinusal em repouso, aumento da voltagem da onda R em derivações precordiais e periféricas, aumento do índice Sokolow.^457^
– Nos atletas com idade ≥14 anos, sugere-se utilizar os critérios de Seattle para o aprimoramento diagnóstico do PRP.^458 – 460^

Outras causas de PRP: padrão de ST juvenil, hipotermia ou hipertermia, hipocalcemia, hiperpotassemia, doença pericárdica (pericardite, cisto pericárdico, tumor pericárdico), tumor do miocárdio (lipoma), cardiomiopatia hipertensiva, isquemia miocárdica, timoma, cardiomiopatia arritmogênica do VD, cardiomiopatia de Takotsubo, miocardite e doença de Chagas.^458 , 459 , 461^

##### 3.3.2. Respostas ao Esforço e Recuperação

Na população pediátrica saudável, as respostas eletrocardiográficas do TE/TCPE (esforço e recuperação) são geralmente diferentes das observadas em adultos, inclusive quanto aos critérios de isquemia, cujas particularidades serão apresentadas a seguir.

###### 3.3.2.1. Onda P e Intervalo PR

No ECG de repouso, as ondas P representam a despolarização atrial e são melhor visualizadas em DII e V1. A condução normal do nó sinoatrial resultará em uma onda P positiva nas derivações I, II e aVF. A amplitude máxima da onda P não muda significativamente durante a infância ( Tabela 24 ), e sua duração geralmente é <100 ms. É considerada como anormal se amplitude >0,25 mV (2,5 mm) em DII, em qualquer idade.^450 , 463 – 465^

Em crianças, os critérios de amplitude e duração na hipertrofia atrial só devem ser aplicados quando o ritmo for sinusal com eixo de onda P entre 0 e 90°. Onda P com amplitude >0,25 mV (2,5 mm) sugere aumento do átrio direito. Onda P entalhada e alargada (duração >110 ms) em DII e/ou bifásica em V1 com deflexão terminal negativa >40 ms sugere aumento do átrio esquerdo.^466 , 467^

A dispersão da onda P corresponde à diferença entre os valores máximo e mínimo de duração das ondas P nas derivações do ECG. A dispersão da onda P e a duração máxima da onda P no ECG de repouso são úteis para avaliar o padrão de distribuição do impulso sinusal, os tempos de condução intra e interatrial, apresentando valor preditivo positivo (VPP) para arritmias em crianças com CC.^468 , 469^

O intervalo PR (PRi) varia com a faixa etária da criança (vide Tabela 24 ). Seu limite inferior encontra-se entre 80 e 90 ms, e o limite superior entre 150 e 180 ms. Principais alterações quanto à duração do PRi:

– Prolongado geralmente associado a CC, miocardite e hipercalemia.
– Curto, associado a síndrome de Wolff-Parkinson-White (WPW) e suas variantes, e doença de armazenamento de glicogênio.

Durante o TE, geralmente observam-se:

– Aumento da amplitude da onda P. Em crianças aparentemente saudáveis, de ambos os sexos entre 5 e 12 anos (média 10,3 anos), a amplitude da onda P no pico do esforço pode atingir 2,57±0,76 mm (ECG de repouso: 1,84±0,48 mm; p<0,001).^470^
– Diminuição progressiva do PRi com o aumento da FC por aceleração na transmissão dos potenciais através dos átrios e do nó atrioventricular (ativação simpática). No pico do esforço, o PRi geralmente situa-se entre 100 e 140 ms.^177^
– Na recuperação, frequentemente observa-se aumento da duração do PRi com diminuição da FC, que podem estar associadas a arritmia sinusal, curtos períodos de ritmo juncional e ritmo atrial ectópico.

Respostas anormais durante o TE:

– Duração prolongada da onda P, duração máxima da onda P aumentada e aumento da dispersão da onda P no ECG de repouso têm sido descritas associadas à comunicação interatrial *ostium secundum* em crianças saudáveis, hipertrofia atrial, estenose pulmonar, tetralogia de Fallot, síndrome de Eisenmenger, pós-cirurgia de Fontan, bloqueio interatrial, cardiomiopatia induzida pela quimioterapia, arritmias, hipertensão e infecções virais.^464 , 468 , 471 – 475^
– Ritmo atrial ectópico (ondas P invertidas nas derivações II e/ou aVF) no ECG de repouso, normalmente retorna ao ritmo sinusal com o esforço/aumento da FC. A persistência do ritmo atrial ectópico geralmente é observada em pacientes com CC.^476 , 477^
– Em crianças e adolescentes com bloqueio atrioventricular (BAV) de primeiro grau acentuado (PRi extremamente prolongado) no ECG de repouso e sua persistência com a progressão do esforço, costumam ocorrer intolerância ao esforço, palpitações, pré-síncope e síncope associados ao fenômeno de dissociação atrioventricular, diagnosticando a pseudossíndrome do marca-passo.^478 , 479^

###### 3.3.2.2. Onda Q

A onda Q apresenta comportamento distinto durante as fases de crescimento da criança. Crianças na faixa etária de 6 meses a 3 anos podem apresentar ondas Q anormais (em DIII e V6) de até 0,6 a 0,8 mV. A amplitude da onda Q atinge seu máximo por volta dos 3 a 5 anos, com subsequente diminuição, mas sem normalizar.^450 , 451 , 463 , 480 , 481^

Em crianças aparentemente saudáveis, entre 8 e 16 anos, as ondas Q em V6 podem atingir até 0,23 a 0,5 mV. Em adolescentes, sugere-se adotar os "Critérios Internacionais" em detrimento dos "Critérios de Seattle" quanto às ondas Q patológicas, em que:^482 , 483^

– Nos "Critérios de Seattle", ondas Q patológicas são definidas como >3 mm de profundidade ou >40 ms de duração em duas ou mais derivações (excluindo derivações DIII e aVR).^460^
– Nos "Critérios Internacionais", as ondas Q patológicas são definidas como uma relação Q/R ≥0,25 ou uma onda Q ≥40 ms de duração em duas ou mais derivações (excluindo DIII e aVR).^484^
– A adoção dos "Critérios Internacionais" resultou em redução de ≈84% de ondas Q patológicas falso-positivas, em consequência do aumento da voltagem dos complexos QRS secundários à prática esportiva e/ou baixa impedância em adolescentes magros.^485 , 486^

Particularidades da onda Q:

– Na avaliação pré-teste, ondas Q anormais no ECG de repouso sugerem via acessória a ser confirmada. Ondas Q patológicas isoladas nas derivações V1 e V2 geralmente são decorrentes de posicionamento inadequado dos eletrodos. A ocorrência de ondas Q patológicas em duas ou mais derivações contíguas pode estar associada a cardiomiopatia dilatada, CMH, VE não compactado e infarto prévio (decorrente de doença de Kawasaki, origem anômala de coronárias etc.).^476 , 484 , 487 , 488^
– Em estudo caso-controle com 44 pacientes com doença de Kawasaki (DK), idade de 7,7±4,8 anos, 22 pacientes foram submetidos a TE para investigação de isquemia miocárdica, dos quais 50% apresentaram alterações isquêmicas (7 com onda Q anormal) com DAC significativa à cineangiocoronariografia. O escore de gravidade das lesões coronarianas no SPECT foi significativamente maior naquelas com ondas Q anormais (51,0±38,8 *versus* 20,0±12,1, p<0,05).^489^

###### 3.3.2.3. Onda R e Onda S

Em crianças >3 anos de idade (similarmente ao observado em adultos), observa-se ativação ventricular normal no plano horizontal (derivações precordiais), com onda S dominante em V1, amplitudes de R e S semelhantes em V2 e V3 e ondas R dominantes de V4 a V6.^490^

Ao esforço, em crianças aparentemente saudáveis, a amplitude da onda R em derivações nas quais é normalmente proeminente (V5 e V6) geralmente diminui em média 5 mm, entretanto, a amplitude da onda R pode permanecer inalterada ou mesmo aumentar. As respostas da amplitude da onda R aparentemente não têm significado diagnóstico, diferentemente do que ocorre na população adulta.^470 , 491^

No ECG de repouso de crianças, a observação de onda R >25 mm em V6, onda Q >5 mm em V6 e onda S >20 mm em V1 sugere HVE. No esforço, a onda S geralmente mantém sua amplitude ou apresenta pequeno aumento, enquanto, na recuperação, costuma ocorrer aumento.^470^

Particularidades das ondas R e S na população pediátrica:

– Estudo em 170 crianças negras aparentemente saudáveis de 7 a 14 anos (idade média de 10,5 anos; 56% do sexo feminino) para determinar o padrão de resposta eletrocardiográfica infantil ao esforço, demonstrou que a amplitude da onda R diminuiu de 27±8 para 22±8 mm (p<0,01), e a amplitude da onda S aumentou de 6,9±4,4 para 7,8±5 mm (p<0,01).^491^
– Estudo em 46 adolescentes (idade média 16,1 anos; sexo masculino) para avaliar a mudança na amplitude da onda R (em V5) durante TE demonstrou que nos normotensos ocorreu redução progressiva da amplitude da onda R (de até 3,8 mm), enquanto nos hipertensos não houve redução (p<0,001).^492^
– Estudo em 55 adolescentes (idade média 15,9 anos; 29 com HAS) para avaliação do efeito de terapia farmacológica no comportamento da amplitude da onda R durante TE demonstrou que, após 16 semanas de tratamento, a amplitude apresentou redução com padrão similar ao dos normotensos.^493^
– A duração do QRS normalmente permanece estável ou diminui ligeiramente durante o esforço progressivo.

###### 3.3.2.4. Onda T e Onda U

Na infância, o padrão da onda T, particularmente nas derivações precordiais, é diferente dos adultos, havendo mudança progressiva no eixo da onda T com a idade. A persistência de onda T positiva em V1 ou V3R além da primeira semana de vida geralmente ocorre na hipertrofia ventricular direita (HVD). Geralmente, a onda T permanece negativa em V1 e V3R na faixa etária de 12 a 16 anos.^494 , 495^

Na primeira infância, a onda T é frequentemente negativa nas derivações V2 e V3, positivando com a progressão da idade. Na faixa etária de 8 a 12 anos, apenas 5 a 10% apresentam ondas T negativas em V2.^496 – 499^ Em V5 e V6, a onda T geralmente é positiva em todas as faixas etárias.^388 , 500^

Presença de onda T negativa no ECG de repouso:

– São consideradas anormais se em duas ou mais derivações contíguas (excluindo V1, aVR e DIII) e com uma profundidade de ≥1 mm. Nas derivações ínfero-laterais (DII, DIII, aVF, V4-V6) geralmente está associada a CMH e HVE.^501 – 503^ Nos atletas adolescentes, em derivações laterais também costuma associar-se a hipertrofia e deslocamento apical dos músculos papilares, podendo ser normal.^504 – 506^
– Assimétricas ou bifásicas, sem depressão do segmento ST e nas derivações V1–V4 são relativamente comuns em adolescentes assintomáticos (idade <16 anos) e atletas jovens negros.^484 , 496 , 501 , 507^
– Em derivações anteriores precedida de elevação do ponto J com supradesnivelamento do segmento ST (SSTs) estão presentes em até 25% dos atletas jovens afro-caribenhos, sendo característica do "coração do atleta negro".^501 , 508 , 509^ Entretanto, a ocorrência de SSTs sem elevação do ponto J precedendo a onda T negativa pode estar associada a cardiomiopatia.^508 , 510^
– Em derivações inferiores e anteriores (V1–V3) seguidas de ondas T positivas em V5 (fenômeno da descontinuidade da onda T) estão geralmente associadas a cardiomiopatia arritmogênica do ventrículo direito (CAVD).^511 , 512^

Particularidades da onda T no TE:

– Em crianças saudáveis, a duração da onda T diminui progressivamente com o esforço. Enquanto a amplitude geralmente diminui no esforço leve, subsequentemente aumenta com a progressão do esforço, podendo ultrapassar a amplitude basal no pico do esforço (em V5, de 4,8 mm em repouso para 7,3 mm).^177 , 470 , 491 , 500^
– O TE é normalmente utilizado para avaliação do comportamento da onda T negativa e sua associação com arritmia esforço-induzida, inclusive em atletas adolescentes.^462 , 484 , 504 , 513^
– Na população pediátrica com onda T negativa, assintomática e sem cardiopatias, é frequente a ocorrência de "pseudonormalização da onda T" (positivação da onda T) de forma total (em todas as derivações) ou parcial (em derivações laterais). Esse comportamento é geralmente benigno, não estando associado a risco de eventos.^514 , 515^
– Em atletas jovens com onda T negativa, a ocorrência de TV ou aumento da densidade de extrassístoles ventriculares (EVs) esforço-induzidas é considerada sugestiva de cardiomiopatia arritmogênica.^516 , 517^
– Na síndrome do QT longo congênito (SQTL), pode ocorrer alternância da onda T associada a incompetência cronotrópica, taquiarritmias ventriculares e comportamento paradoxal do QTi (aumentando em vez de diminuir).^6^

###### 3.3.2.5. Segmento ST/Infradesnivelamento do Segmento ST

As alterações esforço-induzidas do segmento ST têm sido utilizadas para identificar isquemia miocárdica em crianças, adolescentes e adultos. Na população pediátrica, o critério para isquemia é diferente dos critérios em adultos, correspondendo ao infradesnivelamento do segmento ST (ISTs), de morfologia horizontal (retificado) ou descendente ≥1 mm (≥0,10 mV) medido no ponto Y (em 60 ms do ponto J) em relação à linha de base.^7 , 11 , 177 , 300^

**Figura 3:**
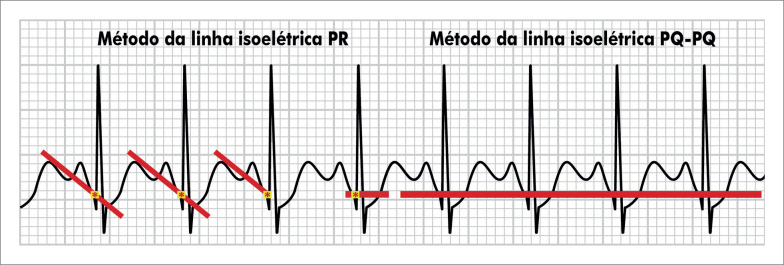
Métodos de definição da linha de base isoelétrica para medição de alterações do segmento ST. Independentemente do método utilizado, as linhas de base encontradas possuem pontos semelhantes para a medição/quantificação de eventuais desnivelamentos do segmento ST. *Ponto no qual se deve basear a medição do infradesnivelamento pelo método da linha isoelétrica PR.

Nessa população, são utilizados dois critérios de definição de linha de base para a medição do ISTs ( Figura 3 ):^7 , 11^

1. Método da linha isoelétrica PR: a linha de base (linha isoelétrica PR) é sobreposta ao segmento PR do complexo QRS para identificar o ponto J.
2. Método da linha isoelétrica PQ-PQ: a linha de base é estipulada conectando os pontos PQ de pelo menos três complexos QRS consecutivos para identificar o ponto J.

O infradesnivelamento isolado do ponto J (sem ISTs) esforço-induzido não deve ser valorizado no diagnóstico de isquemia. Na população pediátrica assintomática e aparentemente saudável, o infradesnivelamento do ponto J pela linha isoelétrica PQ foi observado em 9% dos meninos e em 18% das meninas, enquanto que, pela linha isoelétrica PR, foi de 2,3% em ambos os sexos.

**Figura 4:**
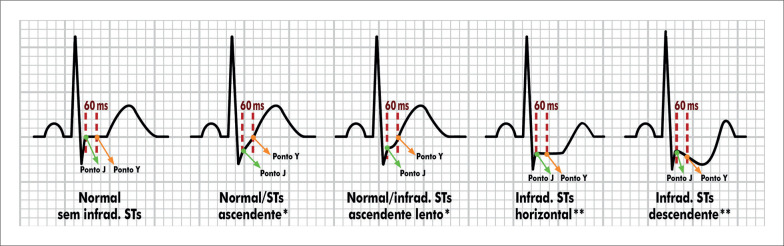
Comportamento do segmento ST e tipos de infradesnivelamento. Infrad.: infradesnivelamento; STs: segmento ST. O infradesnivelamento do segmento ST de qualquer morfologia (<1 mm no ponto Y), principalmente se apresentar normalização precoce (no primeiro minuto da recuperação), é considerado normal (não isquêmico). *Ascendente (depressão do ponto J seguido de infradesnível do segmento ST rapidamente ascendente e sem depressão no ponto Y medido em 60 ms do ponto J) ou ascendente lento (ponto J infradesnivelado com segmento ST ascendendo lentamente além do ponto Y) são considerados normais, não isquêmicos. **Horizontal (retificado) ou descendente, com ISTs ≥1 mm (≥0,10 mV) medido no ponto Y, são considerados alterados, isquêmicos.

Na população pediátrica aparentemente saudável, é considerado normal e não isquêmico o infradesnivelamento do segmento ST esforço-induzido (ISTE) ( Figura 4 ) com:^7 , 11 , 300^

– Morfologia ascendente (depressão do ponto J seguido de infradesnível do segmento ST rapidamente ascendente e sem depressão no ponto Y, medido em 60 ms do ponto J) ou ascendente lento (ponto J infradesnivelado com segmento ST ascendendo lentamente além do ponto Y).
– Qualquer morfologia com infradesnível <1 mm no ponto Y, principalmente se apresentar normalização precoce (no primeiro minuto da recuperação).

As seguintes situações invalidam a interpretação de alterações da repolarização quanto à isquemia: síndrome de WPW; variantes da síndrome de pré-excitação; BRE; MP artificial estimulando o ventrículo; ISTs ≥1 mm no ECG de repouso; terapêutica com digitálicos; ECG com qualidade técnica insatisfatória.^7^

Particularidades do ISTE na população pediátrica:

– ISTE não associado à isquemia por DAC pode ocorrer na hiperventilação, distúrbios hidroeletrolíticos, anemia, *pectus excavatum* e prolapso da válvula mitral (PVM).^177 , 286^
– Na estenose aórtica valvar adquirida, o ISTE ocorre em ≈83% dos pacientes, estando relacionado à pressão sistólica do VE, ao gradiente de saída do VE e ao desequilíbrio entre oferta-consumo de O_2_. Após a correção cirúrgica da estenose aórtica valvar grave, costuma ocorrer redução ou desaparecimento do ISTE.^434 , 518 , 519^
– Na estenose aórtica congênita, também é comum a ocorrência de ISTE. Entretanto, após cirurgia de Ross, não ocorre redução significativa do ISTE. Após valvotomia aórtica (cirúrgica ou por balão), tem sido observado aumento do ISTE.^140^
– Após cirurgia de Fontan, na síndrome do coração esquerdo hipoplásico (SCEH), o ISTE que ocorre em ≈48% dos pacientes não está associado à disfunção ventricular, DAC ou anormalidades de origem coronariana.^520^
– A isquemia esforço-induzida em pacientes com CMH associa-se ao maior risco de morte súbita cardíaca (MSC) [risco relativo (RR): 3,32; intervalo de confiança de 95% (IC95%): 1,27-8,70] e de morte por todas as causas e/ou transplante (RR: 4,86; IC95%: 1,69-13,99).^157^

###### 3.3.2.6. Supradesnivelamento do Segmento ST

**Figura 5:**
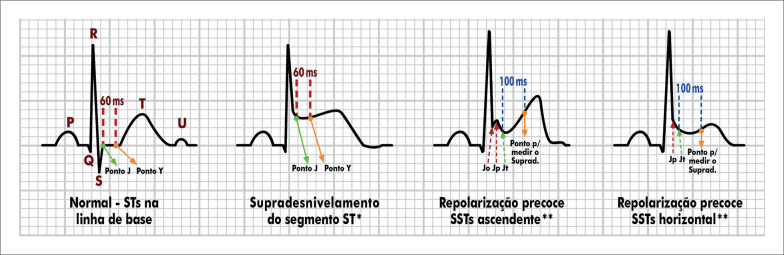
Padrões de supradesnivelamento do segmento ST incluindo a repolarização precoce. STs: segmento ST; Suprad.: supradesnivelamento; SSTs: supradesnivelamento do segmento ST; ms: milissegundos. *Supradesnivelamento do segmento ST esforço-induzido (≥1,0 mm medido a 60 ms após o ponto J). **No padrão de repolarização precoce, o supradesnivelamento do segmento ST deve ser medido a 100 ms após o ponto Jt, sendo também utilizado para avaliar o padrão de supradesnivelamento (ascendente, horizontal/descendente).

O supradesnivelamento do segmento ST esforço-induzido (SSTE) é definido como uma elevação do segmento ST ≥1,0 mm (≥0,10 mV) em 60 ms após o ponto J, ocorrendo em duas ou mais derivações, independentemente de presença de onda Q ( Figura 5 ).^1 , 7 , 521^

Na população pediátrica, o SSTE geralmente está associado a: isquemia miocárdica grave (geralmente transmural) em pacientes com DK, origem anômala de coronárias, após cirurgia de reimplante de coronárias, entre outras; espasmo da artéria coronária por angina vasoespástica/Prinzmetal; aneurisma ventricular esquerdo; isquemia peri-infarto.^521 – 524^

Sugere-se a utilização da descrição das derivações comprometidas por manifestações isquêmicas:^1 , 308^

- V1, V2, V3 (provável parede anterosseptal).
- V1, V2, V3 e V4 (provável parede anterior).
- V3, V4 ou V3-V5 (provável parede anterior localizada).
- V4, V5, V6, D1 e aVL (provável parede ântero-lateral).
- V1 a V6, D1 e aVL (provável parede anterior extensa).
- V5 e V6 (provável parede lateral).
- D1 e aVL (provável parede lateral alta).
- D2, D3 e aVF (provável parede inferior).

Particularidades do SSTE:^1 , 7 , 521^

– No ECG de repouso, a presença de SSTs geralmente está associada a padrão de repolarização precoce, síndrome de Brugada (SBr), miocardite/pericardite e infarto prévio (com onda Q patológica).
– Nos padrões de repolarização precoce e de SBr, geralmente observa-se a redução/desaparecimento do SSTs com o esforço.^126 , 525 , 526^
– O SSTE ≥0,3 mV (3 mm) em derivações sem ondas Q é critério de interrupção do esforço.

###### 3.3.2.7. Repolarização Precoce

A repolarização precoce, na maioria dos pacientes, é uma variante assintomática e benigna do ECG, com elevação do ponto J e supradesnivelamento característico do segmento ST. Entretanto, parte dos pacientes apresenta quadro clínico e padrões eletrocardiográficos específicos de repolarização precoce que estão associados a MSC, configurando a síndrome de repolarização precoce. O padrão de repolarização precoce (PRP) pode ser observado em 1 a 13% da população geral.^527 , 528^

As particularidades do ECG de repouso no PRP são:^529 , 530^

1. Duração dos complexos QRS <120 ms.
2. Presença de entalhe ou de ligadura no final do QRS em inclinação descendente de onda R proeminente. Se houver um entalhe, ele deve ficar totalmente acima da linha de base. O início de uma ligadura (Jo) também deve estar acima da linha de base ( Figura 5 ).
3. O ponto Jp (pico do entalhe do ponto J) deve ser ≥0,1 mV em duas ou mais derivações contíguas do ECG, exceto de V1 a V3.^531^
4. Atletas pediátricos costumam apresentar ponto J com entalhe e segmento ST rapidamente ascendente e côncavo, principalmente nas derivações ínfero-laterais. Outras alterações: bradicardia sinusal em repouso, aumento da voltagem da onda R em derivações precordiais e periféricas e aumento do índice Sokolow.^457^
5. Nos atletas com idade ≥14 anos, sugere-se utilizar os critérios de Seattle para o aprimoramento diagnóstico.^458 – 460 , 484 , 501 , 532^

No PRP, o SSTs deve ser medido a 100 ms após o ponto Jt (final do entalhe do ponto J). Além da magnitude do supradesnivelamento, deve ser descrito o seu padrão:^454 , 455^

– "repolarização precoce com segmento ST ascendente", quando o segmento ST estiver inclinado para cima e seguido por uma onda T vertical.
– "repolarização precoce com segmento ST horizontal ou descendente", quando o segmento ST for horizontal ou descendente (inclinado descendente).

Comportamento e significado do PRP no TE:

– Comum em adolescentes, geralmente apresenta redução progressiva com o esforço, podendo ocorrer seu desaparecimento em cargas moderadas. O PRP com SSTs ascendente rápido, em derivações ântero-laterais, tem sido encontrado em atletas.^533^
– Em TE realizado após MSC abortada, arritmia ventricular sustentada e/ou síncope inexplicada, observou-se PRP persistente ao esforço.^534^
– A ocorrência de TV polimórfica esforço-induzida é marcador de alto risco para MSC.^527 , 535^
– Na população em geral, o retorno do PRP na recuperação é progressivo e lento.^536 , 537^

###### 3.3.2.8. Intervalo QT

O intervalo QT (QTi) é medido do início do QRS ao término da onda T, representando a duração total da atividade elétrica ventricular.^538 – 540^

A avaliação do QTi durante o esforço e recuperação apresenta vários desafios em crianças e adolescentes:

– A medição precisa do QTi é, muitas vezes, dificultada pelo retorno irregular da porção terminal da onda T à linha de base.
– Em FC elevada, é comum observar a fusão das ondas T e P, dificultando identificar o final da onda T.

O aumento da velocidade de repolarização miocárdica ventricular associada ao esforço se reflete na diminuição progressivamente do QTi até o esforço máximo, aumentando linearmente na recuperação.^541^

Devido à variação do QTi com a FC, recomenda-se corrigir o QTi pela FC (QTc) segundo a fórmula de Bazzet:

$$QTc=\frac{QTi}{\sqrt{RR}}$$

*QT medido em milissegundos e distância entre RR em segundos.

A Tabela 24 apresenta os valores de referência do QTc por faixa etária pediátrica.

A fórmula ideal de ajuste do QTc no TE permanece controversa. A interpretação do QTc e sua comparação aos resultados dos estudos da literatura dependem da fórmula utilizada.^124 , 542 – 544^

Em estudos que investigam alterações de repolarização (por exemplo, nas síndromes do QT longo, defeitos cardíacos congênitos ou na avaliação de novos medicamentos), a fórmula de Bazzet apresenta limitações para FC <60 bpm ou >90 bpm, situações em que se sugere a utilização das fórmulas de Fridericia ou Framingham:^308 , 544 – 546^


QTc(fórmuladeFridericia)=QT/RR3QTc(fórmuladeFramingham)=QT+0,154(1-RR)

Em crianças de 1 a 15 anos, um QTc >440 ms é considerado o limite superior/limítrofe, enquanto o QTc >460 ms é considerado prolongado (independente do sexo). O QTc é considerado curto quando <340 ms.^308 , 451^

A avaliação do comportamento do QTc tem importância no diagnóstico da SQTL congênita, na qual o seu aumento pode ocorrer durante o esforço e a recuperação.

Particularidades do QTi e do QTc na população pediátrica:

– O QTi na recuperação aumenta à medida que a FC diminui em ≈15 ms a cada redução de 10 batimentos na FC, retornando ao padrão de repouso em torno de 4 a 5 minutos.^547^
– Em crianças com QTc limítrofe/escore de Schwartz intermediário, o TE permite estratificar o risco, selecionando as que devem ser submetidas a testes genéticos de modo seletivo.^57 , 547 – 549^
– O QTc absoluto ≥460 ms na recuperação ou com aumento paradoxal do QTc (ΔQTc = QTc recuperação – QTc basal, com valor ≥30 ms), distinguiu pacientes com SQTL1 com fenótipo manifesto do tipo oculto.^542^
– Na triagem para SQTL em crianças, o uso da fórmula de Bazett associa-se a elevado número de falsos-positivos, especialmente se a FC estiver aumentada. Nesses casos, sugere-se utilizar a fórmula de Fridericia.^544^

##### 3.3.3. Distúrbios Condução Atrioventricular, Intraventricular e da Formação do Impulso na População Pediátrica

###### 3.3.3.1. Distúrbios Condução Atrioventricular

Em crianças e adolescentes, o BAV de primeiro grau e o BAV de segundo grau Mobitz tipo I geralmente estão associados à atividade parassimpática acentuada, sendo comum em adolescentes altamente treinados e em indivíduos que possuem tônus vagal acentuado. No ECG de repouso, é observado em 0,65 a 1,1% das crianças e em até 12% de adolescentes aparentemente saudáveis.^550 , 551^ Geralmente, desaparecem com o esforço progressivo devido à retirada da atividade vagal e o aumento da atividade simpática. Raramente são desencadeados pelo esforço.^105^

O BAV de segundo grau tipo II geralmente representa doença do sistema de condução AV (infranodal). Raramente é encontrado em atletas jovens aparentemente saudáveis. Pode estar associado ao bloqueio de ramo e ser secundário à cirurgia cardíaca prévia. O TE é útil na identificação do nível anatômico do bloqueio AV e estratificação de risco.^552 , 553^

Na população pediátrica, o BAV de terceiro grau ou completo:^108 , 115 , 554 , 555^

– No bloqueio atrioventricular total (BAVT) congênito, há indicação de MP definitivo: sintomáticos; FC de repouso <55 bpm ou <70 bpm quando associado à cardiopatia estrutural. A Tabela 28 apresenta as principais causas de BAVT.^107 , 114^
– O TE pode ser realizado no BAVT congênito se não houver doenças (congênitas ou não) que reduzam a segurança do paciente.
– O TE é empregado para documentar a sintomatologia, avaliar o aumento da resposta do escape ventricular, determinar eventual ocorrência de ectopias e sua repercussão hemodinâmica.
– Muitos pacientes podem apresentar ACR normal.
– Não devem ser utilizadas equações de predição de VO_2_max e nem de FCmax.
– Existe variabilidade considerável na FC de escape que pode ser gerada pelo marca-passo ventricular intrínseco, normalmente entre 50 e 145 bpm.
– A evolução natural do BAVT congênito consiste no declínio progressivo das frequências ventriculares ao longo da vida. No ECG de repouso, entre 6 e 10 anos, observa-se FC média de 50 bpm; entre 16 e 20 anos, de 45 bpm; e acima de 40 anos, de 38 bpm.
– Fadiga, dispneia, tontura e ectopias ventriculares esforço-induzidas foram responsáveis por 26,5% dos implantes de MP.^556^
– A ectopia esforço-induzida é frequente (50 a 70% dos pacientes) e associou-se ao aumento de risco de morte súbita.
– Nos pacientes com BAVT e graves anormalidades estruturais cardíacas a morte súbita, geralmente está associada a arritmia ventricular complexa. O BAVT localizado no sistema His-Purkinje apresenta pior prognóstico.^116 , 557 , 558^

###### 3.3.3.2. Distúrbios da Condução Intraventricular

Anormalidades da condução intraventricular podem estar associadas à doença sistêmica ou cardiopatia subjacente.

O bloqueio de ramo direito (BRD) é comum em crianças aparentemente saudáveis (entre 6 e 17 anos), com incidência variando de 0,16 a 2,9%, sendo mais comum no sexo feminino. O BRD pode também ocorrer: na anomalia de Ebstein (prevalência entre 80 e 95%); defeito do septo atrial (DSA) do tipo *ostium secundum* (entre ≈90 e 100%); na displasia arritmogênica do VD; após cirurgia de correção da ToF (≈11%) ou do defeito do septo ventricular (DSV; ≈6%). O BRD associado ao bloqueio divisional anterossuperior esquerdo (BDASE) ocorre principalmente nas CC com defeitos do coxim endocárdico. A presença de BRD no ECG de repouso invalida a interpretação de alterações do segmento ST ao esforço apenas nas derivações de V1–V3.^1 , 7 , 279 , 370 , 553^

No bloqueio do ramo esquerdo (BRE) no ECG de repouso deve ser feito o diagnóstico diferencial com WPW (via acessória de parede livre direita). Como achado isolado, o BRE em adolescente é raro e pode estar associado a doença progressiva do sistema de condução intraventricular, com ou sem cardiomiopatia. Também pode ser observado após cirurgia da via de saída do VE. A presença de BRE no ECG de repouso é uma limitação para a análise do segmento ST em relação à isquemia miocárdica, com redução da especificidade e acurácia do TE.^7 , 149 , 388 , 559^

Os distúrbios da condução intraventricular esforço-induzidos, caracterizados por bloqueio do ramo direito ou do ramo esquerdo, raramente ocorrem na população pediátrica. Sua ocorrência tem sido observada tanto em crianças aparentemente saudáveis quanto naquelas com cardiopatia estrutural.^177^

###### 3.3.3.3. Distúrbios da Formação do Impulso

É comum a ocorrência de anormalidades do ritmo cardíaco em pacientes pediátricos, com e sem DCV. Frequentemente, as arritmias são isoladas, transitórias, episódicas e assintomáticas. As classificações quanto a morfologia, interrelações e densidade são similares às aplicadas para adultos e encontram-se descritas na Diretriz Brasileira de Ergometria em População Adulta – 2024.^1 , 149 , 278 , 280^

Principais marcadores de risco para desenvolvimento de arritmias esforço-induzidas: disfunção grave do VE; MP; história de arritmia ou distúrbio do ritmo; ritmo basal não sinusal; CC; cirurgia de correção de CC.^105 , 560 , 561^ Estudo demonstrou que 28% dos pacientes pediátricos submetidos ao TE desenvolveram anormalidades do ritmo cardíaco, sendo 3% clinicamente importantes (TV, taquicardia supraventricular, BAV de segundo grau, fibrilação atrial etc.), associadas a disfunção grave de VE e história prévia de arritmia.^105^

####### 3.3.3.3.1. Arritmias Ventriculares

No ECG de repouso da população pediátrica, as EVs monomórficas isoladas ocorrem com uma frequência de 0,3 a 2,2%. Nas crianças assintomáticas, sem doença cardíaca subjacente, com ECG normal e sem história familiar de MSC, essa arritmia é quase sempre benigna. As EVs tendem a desaparecer com o crescimento da criança.^562 – 565^

O TE está indicado para avaliação de arritmias ventriculares em crianças e adolescentes com:

– EVs (isoladas ou pareadas) identificadas em ECG durante consulta.
– Palpitação, taquicardia, síncope, convulsões e/ou tonturas durante a prática de esportes ou outras atividades físicas.
– Suspeita de canalopatias, via anômala ou TV catecolaminérgica.

O TE fornece informações úteis quanto ao comportamento das EVs e riscos associados. EVs são consideradas benignas quando apresentarem redução de sua densidade ou supressão com o esforço em consequência da taquicardia sinusal.^279 , 390 , 566 , 567^

Crianças aparentemente saudáveis ocasionalmente apresentam raras EVs isoladas esforço-induzidas que podem ser consideradas benignas. Entretanto, a ocorrência de EVs frequentes, polimórficas ou complexas (pareadas e TV não sustentadas) sugerem instabilidade elétrica ventricular.

A TV é rara na população pediátrica. Quando presente, geralmente está associada a cardiopatia estrutural (particularmente na HVE), hereditária (TV polimórfica catecolaminérgica), relacionada a distúrbios elétricos (SQTL) ou idiopática (em jovens aparentemente saudáveis).

As arritmias ventriculares malignas geralmente ocorrem precocemente no esforço devido à excitação elétrica desencadeada pela atividade simpática. Nesses casos, há risco aumentado de taquiarritmias hemodinamicamente instáveis e MSC.^565 , 566^

####### 3.3.3.3.2. Arritmias Supraventriculares

As extrassístoles atriais isoladas no ECG de repouso geralmente são benignas e desaparecem com o esforço.^568^ As extrassístoles supraventriculares (ESVs) isoladas ocorrem em ≈2% das crianças aparentemente saudáveis e em ≈4% das crianças com lesões estruturais cardíacas.^569^

Paciente assintomáticos com ESVs isoladas esforço-induzidas geralmente apresentam boa evolução. Entretanto, extrassístoles atriais esforço-induzidas em crianças com história de síncope ou taquicardia inexplicada requerem maior atenção, pois podem ser gatilho para episódio de taquicardia supraventricular.^568^

A incidência de taquicardia paroxística supraventricular (TPSV) em crianças é de 0,1 a 0,4%, e os principais tipos de apresentação, de acordo com a idade, encontram-se na Tabela 26 .^570^

**Tabela 26:** Prevalência da TPSV em crianças e adolescentes de acordo com a idade^570^

Tipo	>1 a <10 anos	10 a 18 anos	>18 anos
Via anômala	60-65%	50-60%	40-50%
Reentrada nodal	15-20%	20-50%	50-70%
Atrial ectópica	4-6%	3-4%	3%

Em crianças, a taquicardia supraventricular esforço-induzida (TPSV-EI) é rara, geralmente associada a reentrada por via de condução ventricular dentro do nó AV ou por via extranodal acessória (pré-excitação ventricular, síndrome de WPW). Em crianças e adolescentes sintomáticos, a TPSV-EI ocorreu em 12% dos TE.^569^

**Figura 6:**
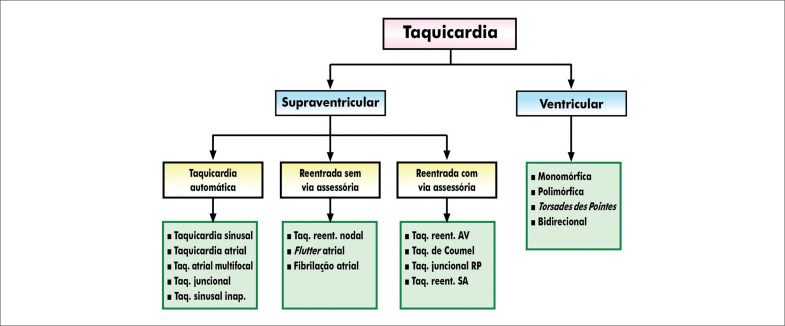
Diagnósticos de ritmo taquicárdico não sinusal na população pediátrica.^570 , 571^ Taq.: taquicardia; Taq. sinusal inap.: taquicardia sinusal inapropriada; Taq. reent. nodal: taquicardia por reentrada nodal; Taq. reent. AV: taquicardia por reentrada atrioventricular; Taq. jncional RP: taquicardia juncional reciprocante persistente; Taq. reent. SA: taquicardia por reentrada sinoatrial (reentrante do nódulo sinusal).

O correto diagnóstico de TPSV-EI em FC elevadas é desafiador pela dificuldade na identificação de alterações nas ondas P, mesmo com complexos QRS normais ( Tabela 27 e Figura 6 ). Na população pediátrica, geralmente o achado inicial da TPSV associa-se ao aumento inesperado e abrupto da FC e/ou outras respostas inadequadas da FC com a modificação da carga de esforço.^570 , 571^

**Tabela 27:** Características eletrocardiográficas das taquicardias (sinusal e supraventriculares) na população pediátrica^570 , 571^

Tipo	Onda P	PR > RP’	FC (bpm)	BAV	Tipo	Idade de início	Particularidades
**Taquicardia sinusal**	Sim, padrão sinusal	--	> FCmax prevista para idade	Não	NP	Qualquer	Valores de referência FCmax na Tabela 24 .
**Taquicardia sinusal inapropriada**	Sim, padrão sinusal	--	>100	Não	P ou PE	>15 anos	Com queixa de palpitações e associada à ansiedade, tontura, pré-síncope e síncope.
**Taquicardia atrial focal/TEA**	Onda P invertida/entalhada e duração >90 ms em V1	Não	Frequência atrial >150% da FC média prevista	Sim	I	≈7 anos	Pode evoluir para taquicardiomiopatia, geralmente reversível com o controle da arritmia.^572 , 573^
**Taquicardia atrial multifocal**	Várias morfologias	--	>100	Não	P	Qualquer	Presença de pelo menos três morfologias de ondas P e três intervalos PR diferentes.
**Taquicardia juncional** *	Pode apresentar-se sem onda P visível (dentro ou após o QRS)	--	>100	Não	P ou I	≈1 ano	Congênita é rara e, nos pacientes sem cirurgia cardíaca prévia, pode ser refratária à terapia com alta morbimortalidade. Após cirurgia cardíaca, ocorre em até 5% dos pacientes.^574^
**Reentrada NS**	Padrão sinusal	Não	170-300	Sim	P	Qualquer	--
**Reentrada atrioventricular antidrômica**	Não visível	Sim	170-200	Não	P	>6 anos	ECG de repouso geralmente com onda delta. Complexos QRS durante a taquicardia são alargados e aberrantes, podendo mimetizar taquicardia ventricular.
**Reentrada atrioventricular ortodrômica**	Morfologia diversa, dependendo da localização da via acessória	Sim	220-360	Não	P	<3 meses ou >6 anos	O QRS da taquicardia geralmente é estreito e a onda P retrógrada.
**TJRP**	Negativas em derivações inferiores	Não	<170	Não	I	≈6 anos	--
**Reentrada intra-atrial**	Oscilante tipo *flutter*	--	160-220	Sim	I	≈12 anos	--

BAV: bloqueio atrioventricular; NS: nó sinusal; TJRP: taquicardia juncional reciprocante persistente; TEA: taquicardia ectópica atrial; NP: não é paroxística, acelera e termina gradualmente; P: paroxística; I: incessante; PE: persistente; FC: frequência cardíaca; FCmax: frequência cardíaca máxima; ECG: eletrocardiograma. *Também denominada como taquicardia ectópica juncional.

*Flutter* e fibrilação atrial (FA) são relativamente comuns em crianças portadoras de cardiomiopatias e CC. O *flutter* atrial pode ser conduzido para os ventrículos na proporção de 1:1 (frequência ventricular >300 bpm) ou na proporção de 2:1 (frequência entre 150 e 200 bpm). O *flutter* atrial atípico (com ondas P mais lentas, arredondadas, de menor voltagem e separadas por linha isoelétrica) é uma arritmia potencialmente letal, geralmente presente em cardiopatias complexas.

A FA esforço-induzida é incomum em crianças, podendo ocorrer de forma paroxística e assintomática nas cardiopatias.

####### 3.3.3.3.3. Bradiarritmias/Disfunção do Nó Sinusal

A bradiarritmia na população pediátrica é definida como uma FC abaixo do menor valor normal para a idade (vide Tabela 24 ). Manifesta-se, comumente, como bradicardia sinusal, ritmo juncional (de escape) ou ritmo decorrente de BAV (segundo grau, avançado ou total).^370 , 575 – 578^

Cerca de 15 a 25% das crianças assintomáticas saudáveis podem apresentar arritmia sinusal, ritmo atrial ectópico, ritmo atrial multifocal e ritmo juncional. O ritmo juncional é comum na população pediátrica com tônus vagal acentuado, ocorrendo em ≈45% das crianças entre 7 e 10 anos, em ≈13% dos meninos entre 10 e 13 anos (durante o sono) e em ≈20% dos adolescentes atletas.^579^

A DNS é caracterizada pelo espectro de distúrbios eletrocardiográficos e eletrofisiológicos que envolvem o nó sino-atrial e suas conexões com uma ou mais das seguintes alterações do ECG: bradicardia sinusal, bradicardia juncional, parada ou pausa sinusal, bloqueio sino-atrial, ritmos de substituição etc. Crianças com DNS podem ser completamente assintomáticas ou podem apresentar queixa de fraqueza, palidez, pré-síncope/síncope ou IC. A DNS sintomática normalmente requer implante de MP.^576 , 580 – 582^

As principais causas de bradiarritmias na população pediátrica são apresentadas na Tabela 28 . Na população pediátrica, as bradiarritmias podem desencadear aos esforços: dor torácica, dor precordial típica, fadiga, dispneia, intolerância aos esforços, palpitações, tontura, síncope e IC.^577 , 578 , 582^

**Tabela 28:** Etiologia das bradiarritmias na população pediátrica^575 , 578 , 583 – 585^

Bradicardia sinusal/juncional	
Origem	Causas
Adaptativa	Hipervagotonia em atletas altamente treinados; indivíduos com tônus vagal acentuado.
Respiratória	Hipóxia; apneia/bradicardia da prematuridade.
Cardíaca	Disfunção do nó sinusal (hereditária ou secundária); cardiopatias congênitas; defeito do septo atrial; após cirurgia cardíaca/reparo transcateter.
Genética	Distúrbios hereditários progressivos da condução cardíaca: SCN5A, TBX5, SCN1B-LOF, CASQ2, HCN4 etc.
Neurocardiogênica	Aumento do tônus vagal; reflexo de Bezold-Jarisch; situacional (tosse, crises de retenção da respiração, sono etc.); estimulação esofágica, nasofaríngea, peritoneal ou retal.
Neurológica	Aumento da pressão intracraniana; malformação de Chiari.
Psiquiátrica	Anorexia nervosa.
Endócrina	Hipotireoidismo.
Medicamentosa	Betabloqueadores; agonistas alfa-2; fentanil; fenilefrina; metoxamina.
Miscelânia	Hipotermia. Hipoglicemia. Anormalidades eletrolíticas: hipo/hipercalemia; hipo/hipercalcemia; hipomagnesemia.
**Bloqueio atrioventricular total/terceiro grau**	
**Origem**	**Causas**
Cardíaca	Congênito; cardiopatias congênitas; distúrbios genéticos; síndrome do QT longo; transposição das grandes artérias; cirurgia cardíaca; doença arterial coronariana.
Imunológica	Doença do tecido conjuntivo materno; lúpus eritematoso sistêmico; síndrome de Sjögren.
Infecciosa	Miocardite; endocardite; doença de Lyme; doença de Chagas; difteria; rubéola; caxumba; triquinose; febre maculosa; vírus da imunodeficiência humana; doença reumática aguda.
Metabólica	Síndrome de Kearns-Sayre; deficiência de carnitina; doença de armazenamento de glicogênio.
Miscelânia	Distrofia muscular; cardiomiopatia eosinofílica; idiopática.

Marcadores de risco elevado de morbimortalidade na população pediátrica com bradiarritmias:^576 , 578 , 582^

– Histórico de sopro cardíaco ou CC.
– Síncope, especialmente desencadeada por esforços, ruídos altos, medo ou estresse emocional extremo.
– Lipotimia ou síncope sem sintomas premonitórios ou fatores precipitantes.
– Dor torácica, palpitações ou dispneia.
– História familiar de MSC, SQTL, perda auditiva neurossensorial e implante de MP.
– Uso de medicamentos que podem resultar em bradicardia.

Particularidades do TE/TCPE nas bradiarritmias e DNS na população pediátrica:

– Fornece informações sobre a capacidade do nó sinusal e nó AV de responderem ao aumento da atividade adrenérgica com o esforço.^43^
– Permite avaliar sintomas esforço-induzidos, a resposta cronotrópica ao esforço, arritmias associadas, ACR e estratificação de risco.^43 , 404 , 586^
– Em pacientes com bradicardia em repouso, a ocorrência de resposta cronotrópica normal auxilia afastar a DNS.
– Pacientes com CC complexa geralmente apresentam DNS associada, incompetência cronotrópica e comprometimento da ACR.^189 , 587 , 588^
– Pacientes com defeito do septo atrial geralmente apresentam incompetência cronotrópica após reparo transcateter ou cirúrgico.^589 , 590^
– Na ToF reparada é frequente a incompetência cronotrópica e a disfunção grave do nó sinusal, que ocorrem em ≈4% dos pacientes.^591^
– Na cirurgia de Fontan a incompetência cronotrópica ocorre em até 62% dos pacientes e contribui para o comprometimento da ACR.^592 , 593^

#### 3.4. Avaliação Metabólica Indireta

##### 3.4.1. VO_2_/Aptidão Cardiorrespiratória/Classificação Funcional

No TE, a determinação indireta (estimativa) do consumo de oxigênio (VO_2_) é considerada a principal avaliação metabólica ao esforço. O VO_2_ é um dos principais parâmetros de gravidade das CC, sendo relevante para estratificação de risco e prognóstico. Recomenda-se a apresentação dos resultados do VO_2_ em mL/kg/min (também aceitável mL.kg^-1^.min^-1^). Também pode ser expresso por meio do equivalente metabólico (do inglês, *metabolic equivalent of task* - MET). Cada 1 MET corresponde a 3,5 mL/kg/min de VO_2_.^1^

O consumo máximo de oxigênio (VO_2_max) expressa a maior quantidade de oxigênio extraído do ar inspirado durante a realização de TE considerado de esforço máximo (exemplos: sinais ou sintomas de exaustão física; incapacidade de prosseguir o esforço etc.). No TE em que não houver as características de um esforço máximo, o VO_2_ obtido deve ser denominado VO_2_pico.^1^

Até os 12 anos de idade, não há diferenças significativas no VO_2_max em relação aos sexos. Após essa idade, os adolescentes do sexo masculino podem alcançar valores de VO_2_ até 25 a 30% maiores do que os observados no sexo feminino.^22^

A aptidão cardiorrespiratória (ACR)/classificação funcional é uma estratificação de desempenho físico baseada no VO_2_max (estimado no TE e medido no TCPE). Quanto à obtenção do valor de VO_2_max previsto, sugere-se a adoção de tabelas de referência específicas para a faixa etária pediátrica (crianças e adolescentes), baseadas em sexo, idade e IMC. A utilização de tabelas de referências específicas para CC e/ou doença pulmonar é útil e contribui para a estratificação de risco ( Anexo 4 ).^80 , 176^

Na eventualidade de uso de equações para estimar o VO_2_max previsto na população pediátrica, considerar:

1. Para bicicleta ergométrica, protocolo escalonado:^594^
  – Masculino: VO_2_max previsto = peso x (50,75 – 0,372 x idade)
  – Feminino: VO_2_max previsto = (peso + 43) x (22,78 – 0,17 x idade)
  Quando o peso real for maior que o previsto para idade e sexo, o peso previsto deve ser utilizado nas equações: peso previsto para sexo masculino = (0,79 x estatura) – 60,7; peso previsto para sexo feminino = (0,65 x estatura) – 42,8. Idade em anos; estatura em centímetros; peso em kg.
2. Para esteira ergométrica (teste incremental):^594^
  VO_2_max previsto = (0,046 x estatura) – (0,021 x idade) – (0,62 x sexo) – 4,31
  Sendo que: sexo masculino = 0 e sexo feminino = 1; idade em anos; estatura em centímetros.
3. Bicicleta ergométrica, protocolo de rampa. Crianças e adolescentes saudáveis entre 12 e 17 anos:^595 , 596^
  – Masculino: VO_2_max previsto = (– 0,297 x estatura^2^) + (105,9 x estatura) + (36,6 x massa corporal) – 8.660
  – Feminino: = VO_2_max previsto = (– 0,24 x estatura^2^) + (86,8 x estatura) + (14,7 x massa corporal) – 6.424

Sendo a estatura em centímetros, e a massa corporal em quilogramas. Se o IMC for ≤ ao percentil 85 para a idade, utilizar a massa corporal real. Se o IMC for > que o percentil 85 para a idade, utilizar a massa corporal corrigida, através da estimativa do valor da massa corporal correspondente ao percentil 85 para a idade.

Devido à grande heterogeneidade da população pediátrica, inclusive entre países, ainda não foi possível estabelecer uma classificação unificada de normalidade para o VO_2_ e o nível de ACR.^595^

Em crianças portadoras de cardiomiopatia, CC, IC e valvopatias, sugere-se aplicar, no pré-teste, a avaliação para determinação da classificação funcional de acordo com a faixa etária: Ross modificada (crianças <6 anos) ou a *New York Heart Association* (NYHA) (crianças >6 anos) – vide Tabela 29 .^379 , 793^

**Tabela 29:** Classificações funcional baseada na história clínica, por faixa etária^379 , 793^

Classe	Ross modificada para crianças <6 anos	NYHA para crianças >6 anos
I	Sem limitações ou sintomas.	Sem limitações na atividade física.
II	Lactentes: taquipneia leve ou sudorese com alimentação. Crianças maiores: dispneia aos esforços.	Pode apresentar fadiga, palpitações, dispneia ou angina durante o exercício moderado, mas não durante o repouso.
III	Lactentes: taquipneia ou sudorese acentuada com a alimentação. Tempos de alimentação prolongados com déficits de crescimento. Crianças mais velhas: dispneia acentuada aos esforços.	Sintomas com esforço mínimo. Limitação acentuada da atividade física.
IV	Sintomas em repouso, tais como taquipneia, retrações, grunhidos ou sudorese.	Incapaz de realizar qualquer atividade física porque normalmente apresentam sintomas de IC em repouso e que pioram com qualquer esforço.

NYHA: New York Heart Association; IC: insuficiência cardíaca.

A Tabela 30 apresenta a proposta de classificação da ACR para população brasileira, pelo VO_2_max, por sexo, para a faixa etária de 10 a 14 anos.^597^ A Tabela 31 apresenta o comportamento da ACR nas principais cardiopatias e cardiomiopatias congênitas na população pediátrica.

**Tabela 30:** Classificação da aptidão cardiorrespiratória pelo VO_2_ máximo (mL/kg/min) medido diretamente no TCPE para a faixa etária de 10 a 14 anos

	Sexo feminino	Sexo masculino
Muito fraca	<33,0	<38,7
Fraca	33,0-36,4	38,7-43,3
Regular	36,5-38,7	43,4-47,9
Boa	38,8-42,4	48,0-52,2
Excelente	42,5	52,3

Adaptado de: Rodrigues AN et al. Maximum oxygen uptake in adolescents as measured by cardiopulmonary exercise testing: a classification proposal.^597^

**Tabela 31:** Comportamento da aptidão cardiorrespiratória nas cardiopatias e cardiomiopatias congênitas em crianças e adolescentes^10 , 79 , 80 , 95^

Cardiopatia/cardiomiopatia congênita	Aptidão cardiorrespiratória
DSA ou DSV, pequenos e não reparados	Normal.
DSA grande, não reparado	Levemente reduzida.
DSA ou DSV, reparados	Normal ou levemente reduzida.
DSA ou DSV, grandes e reparados	Normal ou levemente reduzida.
Obstrução da via de saída ventrículo esquerdo	Normal, exceto em casos graves.
ToF reparada	Leve a moderadamente reduzida.
TGA – cirurgia/interruptor arterial	Normal ou levemente reduzida.
TGA – cirurgia/interruptor atrial	Moderadamente reduzida.
PCA com HAP	Moderada a acentuadamente reduzida.
Síndrome de Eisenmenger	Acentuadamente reduzida.
Ventrículo único	Moderada a acentuadamente reduzida.
Cirurgia de Fontan	Moderada a acentuadamente reduzida.
BAVT congênito	Pode ser normal ou leve a moderadamente reduzida.
CMH	Leve a acentuadamente reduzida.
EAo congênita moderada/grave	Leve a acentuadamente reduzida.
Válvula aórtica bicúspide *	Leve a acentuadamente reduzida.
Prolapso da válvula mitral	Pode ser normal ou leve a moderadamente reduzida.

DSA: defeito do septo atrial; DSV: defeito do septo ventricular; ToF: tetralogia de Fallot; TGA: transposição das grandes artérias; PCA: persistência do canal arterial; HAP: hipertensão arterial pulmonar; CMH: cardiomiopatia hipertrófica; BAVT: bloqueio atrioventricular total; EAo: estenose valvar aórtica.

* Com EAo moderada/grave, com insuficiência aórtica ou com CoAo associadas.

### 4. Critérios para Interrupção do Esforço (Clínicos, Hemodinâmicos, Eletrocardiográficos)

Os principais critérios de interrupção do esforço em população pediátrica são apresentados na Tabela 32 .^7 , 11 , 176 , 177^ A interrupção também pode ocorrer em outras situações não descritas, mas consideradas de risco de intercorrências graves e que deverão ser detalhadas no laudo do exame.

**Tabela 32:** Critérios de interrupção do esforço no TE/TCPE em população pediátrica^7 , 11 , 176 , 177^

Parâmetro	Critérios
**Objetivo do exame**	Os achados diagnósticos foram atingidos, e a continuação do esforço não fornecerá informação adicional relevante.^7^
**Sintomatologia** *	Sinais ou sintomas indicam que a continuação do esforço pode comprometer o bem-estar do paciente: – Exaustão física. – Dor e/ou exaustão da musculatura dos membros inferiores. – Claudicação de membros inferiores (limitante), ataxia. – Vertigem persistente e limitante, náusea, pré-síncope, síncope. – Desconforto ou dor torácica crescente com incremento das cargas do esforço (limitante) ou angina típica (moderada a forte intensidade). – Dispneia precoce e desproporcional à intensidade do esforço. – Sensação de taquicardia intolerável.
**Exame físico/variáveis cardiovasculares e respiratórias**	– Palidez cutânea e de mucosas, sudorese profusa e desproporcional, má perfusão periférica. – Taquipneia desproporcional ao esforço, broncoespasmo, estertores crepitantes em bases pulmonares. – Queda progressiva e persistente da PAS com aumento da carga de esforço. ** – Elevação acentuada da PAS ≥250 mmHg.^7 , 200 , 418^ *** – Elevação da PAD ≥125 mmHg. *** – Queda de 10 pontos em relação à saturação de repouso associada a sintomas ou SpO_2_ <85%.
**Eletrocardiográficas**	– Modificações do segmento ST: infradesnivelamento (horizontal e descendente) ou supradesnivelamento do segmento ST de 0,3 mV (3,0 mm). – Taquicardia supraventricular não sustentada sintomática ou com repercussão hemodinâmica. – Taquicardia supraventricular sustentada (≥30 segundos) mesmo que assintomática ou sem repercussão. – Fibrilação atrial ou *flutter* atrial esforço-induzidos. – Aumento da densidade e complexidade de arritmia ventricular com progressão do esforço. – Taquicardia ventricular não sustentada (≥3 batimentos/<30 segundos) ou episódio polimórfico. – Taquicardia ventricular sustentada (≥30 segundos). – Fibrilação ventricular. – Bloqueio atrioventricular de 2 ° e 3 ° graus. – Prolongamento do QTc >500 ms. – Bloqueio de ramo esforço-induzido não distinguível de taquicardia ventricular. – Em paciente com CDI, interromper o esforço 10 batimentos abaixo da FC de acionamento do desfibrilador. – Queda persistente da FC com o incremento de carga, principalmente em presença de sintomas de baixo débito cardíaco.
**Outras**	– A pedido do paciente, independente da ocorrência de anormalidades. – Falência dos sistemas de monitorização e/ou registro eletrocardiográfico. – Inadaptação e/ou falta de coordenação ao ergômetro.

PAS: pressão arterial sistólica; PAD: pressão arterial diastólica; FC: frequência cardíaca; CDI: cardioversor-desfibrilador implantável; SpO_2_: saturação de oxigênio por oximetria digital.

* Crianças, principalmente entre 3 e 7 anos, podem apresentar limitações (associadas ao grau de desenvolvimento cognitivo) para avaliar adequadamente as alterações sensoriais periféricas decorrentes do esforço, sua intensidade e sintomas associados.

** Queda da pressão arterial sistólica no esforço com valor inferior ao da PAS de repouso ou aumento inicial da PAS no esforço e subsequente queda ≥20 mmHg.

*** Em crianças e adolescentes aparentemente saudáveis e sem intercorrências ou sintomas durante o esforço. Nas cardiomiopatias e CC, atentar para repercussões hemodinâmicas e sintomas, principalmente se a PAS exceder 200 mmHg e a PAD exceder 110 mmHg.

### 5. Elaboração do Laudo do TE

O laudo do TE de crianças e adolescentes deve seguir o mesmo padrão de estruturação e requisitos mínimos recomendados para o laudo do TE apresentado na Diretriz Brasileira de Ergometria em População Adulta – 2024:^1^

1. Descrição dos dados gerais do exame.
2. Dados observados, mensurados e registrados.
3. Relatório descritivo do TE.
4. Conclusões.
5. Registros eletrocardiográficos.

Adicionalmente, recomenda-se:

– Não utilizar escores de risco pré- e pós-teste específicos para população adulta em população pediátrica, pois os mesmos não são válidos ou extrapoláveis.
– Referir ajustes nos protocolos, critérios e variáveis referentes às características das crianças e adolescentes (tais como doenças, idade, sexo, IMC, superfície corporal, medicações em uso etc.).
– Preferencialmente, apresentar os valores de referência utilizados para as variáveis medidas.
– Quando pertinente e disponível, comentar os achados em relação às doenças de base, incluindo prognóstico e riscos.
– Em caso de TE seriado, quando possível, realizar comentário sobre a evolução dos achados.

### 6. TCPE de Crianças e Adolescentes

#### 6.1. Respostas Metabólicas, Ventilatórias e de Trocas Gasosas em Crianças e Adolescentes

##### 6.1.1. Metabolismo Celular, Resposta Fisiológica e Hormonal ao Exercício

Crianças e adolescentes apresentam respostas metabólicas ao esforço diferentes das dos adultos. As reservas de trifosfato de adenosina (ATP) e fosfocreatina não estão relacionadas à idade. Os níveis de glicogênio muscular em repouso são menores em crianças, atingindo na adolescência os níveis de adultos.^1 , 598 , 599^

Na comparação com adultos, a massa muscular em crianças é menor, com diferente utilização de fontes de energia e distintas adaptações metabólicas/hormonais e maior dependência da oxidação da gordura, repercutindo em grande mobilização de ácidos graxos livres. A liberação de glicerol e o aumento do hormônio do crescimento em crianças pré-adolescentes corroboram esses achados.^600 , 601^

A imaturidade do metabolismo anaeróbico lático em crianças, com atividade glicolítica reduzida, decorre de:^598 , 599 , 602^

– Diferenças na tipagem das fibras musculares esqueléticas com maiores proporções de fibras de contração lenta (tipo I) do que em adultos não treinados.
– A via anaeróbia lática para ressíntese de ATP geralmente está reduzida em indivíduos jovens durante esforços de alta intensidade.
– Em crianças pré-púberes, observam-se atividade reduzida das enzimas fosfofrutoquinase-1 e lactato desidrogenase e produção limitada de lactato muscular em relação aos adultos.

Portanto, crianças e adolescentes adaptam-se bem ao exercício prolongado, moderado e intenso, apresentando rápida recuperação após o esforço.^603 , 604^

Em crianças, as adaptações hormonais para a utilização do substrato energético durante exercício prolongado apresentam menor redução dos níveis de insulina, com aumento das catecolaminas e glucagon. Essa resposta corresponde à regulação menos efetiva da glicemia com maior risco de hipoglicemia.^598 , 605 , 606^

Na fase do estirão de crescimento pubertário, ocorre a liberação de hormônios (exemplo: somatotropina, fatores de crescimento semelhantes à insulina e hormônios esteroides sexuais) responsáveis por mudanças na composição corporal e aumento da massa corporal magra, acarretando melhora da aptidão e desempenho físico, particularmente de natureza anaeróbica.^599 , 607 – 609^

##### 6.1.2. Ventilação Pulmonar, Gases no Ar Expirado, Espirometria e Variáveis Derivadas

As principais variáveis do TCPE (metabólicas, ventilação pulmonar, gases no ar expirado, espirometria e variáveis derivadas) na população pediátrica e suas respectivas unidades e interpretações encontram-se na Tabela 33 . As diferenças de comportamento das variáveis entre crianças e adultos constam na Tabela 34 .^1 , 11 , 176 , 179 , 610^

**Tabela 33:** Principais variáveis do TCPE e respectivas interpretações^11 , 176 , 179 , 610 , 611^

Parâmetros do TCPE	Sigla/abreviatura	Unidade	Interpretações
Consumo de oxigênio	VO_2_	mL/kg/min	É definido como o volume de O_2_ extraído do ar inspirado em um determinado período de tempo. Pode ser obtido pela equação de Fick ( Figura 1 ).
Consumo máximo de oxigênio previsto	VO_2_max previsto	mL/kg/min	Sugere-se utilização de tabelas de VO_2_max previsto por faixa etária e sexo, em população aparentemente saudável ou com cardiopatia ( Anexo 4 ).
Consumo de oxigênio no pico do esforço	VO_2_pico	mL/kg/min	Nível máximo de VO_2_ medido durante o TCPE. Pode ser expresso como % do VO_2_max previsto (padrões de idade e sexo correspondentes). É considerado normal quando >20 mL/kg/min. Se >80% do previsto, indica aptidão cardiorrespiratória adequada.
Consumo máximo de oxigênio	VO_2_max	mL/kg/min	Platô de VO_2_ obtido apesar do aumento da intensidade do esforço (esforço máximo). Pode ser expresso como % do VO_2_max previsto.
Limiar ventilatório 1 /limiar anaeróbico	LV1/LA	mL/kg/min ou % do VO_2_max previsto	O LV1 corresponde ao valor do VO_2_ acima do qual a produção de energia passa a contar com crescente participação do metabolismo anaeróbico lático. Pode ser expresso como % do VO_2_max previsto (normalmente ocorre >40% do VO_2_pico). É o ponto a partir do qual ocorre aumento desproporcional de VEmin e VCO_2_ em relação ao VO_2_.
Ventilação por minuto	VEmin	L/min	Ventilação (com base no volume corrente e frequência respiratória) durante o esforço. Em indivíduos saudáveis, o valor da VEmin é mais do que suficiente para manter a PaCO_2_ em qualquer carga de trabalho. Na insuficiência cardíaca, a perfusão pulmonar é alterada e a VEmin aumenta, o que se correlaciona com mau prognóstico.
Equivalente ventilatório de O_2_	VE/VO_2_	-	VE/VO_2_ corresponde ao número de litros de ar que estão sendo respirados para cada litro de absorção de O_2_.
Equivalente ventilatório de CO_2_	VE/VCO_2_	-	VE/VCO_2_ corresponde ao número de litros de ar que estão sendo respirados para eliminar 1 litro de CO_2_. Os valores normais são geralmente <30.
Eficiência ventilatória (ventilação/produção de CO_2_)	Inclinação VE/VCO_2_	-	Durante o TCPE incremental normal, a VEmin se correlaciona linearmente com o VCO_2_. A inclinação VE/VCO_2_ em indivíduos normais é de cerca de 25 a 30. Também chamada de eficiência ventilatória, aumenta na insuficiência cardíaca, hipertensão pulmonar e/ou doenças pulmonares intrínsecas e se correlaciona com o prognóstico.
Inclinação da eficiência da captação do oxigênio	OUES	L/min	Relação logarítmica entre VO_2_ e VEmin durante o TCPE (VO_2_ = a log10 VEmin + b, onde a = valor de referência de OUES calculado). Quanto mais acentuada a inclinação, melhor a eficiência ventilatória. O valor de OUES depende da idade e da área de superfície corporal. Sugere-se expressar esse valor para a área de superfície corporal ou peso corporal. OUES/área de superfície corporal ≥1.200 ou OUES ≥35/peso corporal (kg) correlaciona-se com o VO_2_pico >80% do previsto. O valor de OUES diminui significativamente em crianças com CC e doença vascular pulmonar.
Pulso de oxigênio	PuO_2_	mL/kg/min/bpm	É obtido pela divisão do VO_2_ pela FC (VO_2_/FC), refletindo a quantidade de O_2_ que é transportada a cada sístole cardíaca, tendo relação direta com o volume sistólico, permitindo avaliar a função do ventrículo esquerdo. O valor absoluto normal do PuO_2_ é >80%.
Quociente respiratório	QR	-	Também conhecido como razão de trocas respiratórias, corresponde à razão entre VCO_2_ e o VO_2_, permitindo identificar a intensidade do esforço e o macronutriente utilizado para gerar energia. O TCPE pode ser considerado máximo quando o QR é ≥1,10.
Oximetria de pulso	SpO_2_	%	Deve ser >95% ao longo do TE/TCPE. O declínio nos níveis de oxigenação da hemoglobina <90% indica capacidade prejudicada de aumentar adequadamente a transferência de oxigênio alvéolo-pulmonar para o capilar sanguíneo durante o esforço. A diminuição de ≥4% é considerada dessaturação, ocorrendo mais comumente em pacientes com anormalidade de difusão pulmonar. Outras anormalidades como *shunts* da direita para a esquerda ou incompatibilidade entre ventilação e perfusão, podem resultar em dessaturação associada ao esforço.
Relação de consumo de oxigênio pela taxa de trabalho	ΔVO_2_/ΔWR	mL/min/W	Reflete a capacidade muscular de extrair O_2_ e gerar ATP. Em determinado momento do esforço, o achatamento da curva e/ou queda do valor do ΔVO_2_/ΔWR (<10 mL/min/W) sugerem problema no transporte de O_2_ (isquemia miocárdica/disfunção ventricular).
Pressão parcial expiratória final de dióxido de carbono	PETCO_2_	mmHg	Obtida a partir da medida da FECO_2_, refletindo a PaCO_2_. Durante o esforço, a PETCO_2_ aumenta de 3 a 8 mm e, subsequentemente, diminui levemente até o esforço máximo. O seu valor varia de 36 a 42 mmHg na ausência de doenças pulmonares. A PETCO_2_ medida no LV1 correlaciona-se com o DC, refletindo a gravidade da IC crônica. Valores reduzidos indicam incompatibilidade ventilação/perfusão e pior prognóstico. A PETCO_2_ <36 é encontrada na CC com *shunt* direita-esquerda, padrão ventilatório taquipneico e na IC com resposta atenuada do DC ao esforço.
Ventilação voluntária máxima	VVM	L/min	É o volume máximo de ar em repouso mobilizado no esforço voluntário em um minuto. Pode ser calculada pelas fórmulas: no sexo feminino VVM = VEF1 X 35; no sexo masculino VVM = VEF1 X 40.
Reserva ventilatória máxima	RV máxima	-	É a relação entre a VVM em repouso e a VEmin máxima no esforço. Crianças saudáveis têm uma RV de pelo menos 11 L/min ou 20 a 40% de sua VVM. RV máxima <30% sugere limitação ventilatória, sendo útil no diagnóstico diferencial de dispneia relacionada à IC e às doenças respiratórias crônicas.
Volume expirado forçado no 1° segundo	VEF1	%	VEF1 é o volume expirado medido no primeiro segundo durante a manobra de capacidade vital forçada (CVF – volume obtido em uma única inspiração máxima seguida de uma expiração também máxima). O VEF1 é uma das principais variáveis no diagnóstico de distúrbio ventilatório obstrutivo (asma esforço-induzido, broncoespasmo esforço-induzido etc.).

TCPE: teste cardiopulmonar de exercício; O_2_: oxigênio; CO_2_: dióxido de carbono; PaCO_2_: pressão parcial de CO_2_; FC: frequência cardíaca; ATP: trifosfato de adenosina; VCO_2_: produção de dióxido de carbono; DC: débito cardíaco; CC: cardiopatia congênita; IC: insuficiência cardíaca; CFV: capacidade vital forçada.

**Tabela 34:** Comparação das variáveis cardiovasculares, ventilatórias e metabólicas do TCPE entre crianças e adultos, durante esforços em qualquer intensidade (em níveis submáximo e máximo)^1 , 11 , 176 , 179 , 599 , 610^

Variável		Comparação com adultos *	Esforço submáximo		Esforço máximo	
			Crianças	Adultos	Crianças	Adultos
**Cardiovascular**						
	VO_2_pico (mL/kg/min)	Mais alto	↑↑	↑	↑↑	↑
	FCpico (bpm)	Mais alta	↑↑	↑	↑↑	↑
	Volume sistólico (mL/bpm)	Mais baixo	↑	↑↑	↑	↑↑
	Débito cardíaco (L/min)	Mais baixo	↑	↑↑	↑	↑↑
	Diferença arteriovenosa O_2_	Mais alta no esforço submáximo	↑↑	↑	↑	↑↑
	Pressão arterial sistólica e diastólica	Mais baixa	↑	↑↑	↑	↑↑
**Pulmonar**						
	Frequência respiratória (respirações/min)	Mais alta	↑↑	↑	↑↑	↑
	Volume corrente (L)	Mais baixo	↑	↑↑	↑	↑↑
	VEmin pico (L/min)	Mais baixo	↑	↑↑	↑	↑↑
	VE/VCO_2_ **	Mais alta	↑↑	↑	↑↑	↑
	VE/VO_2_ **	Mais alta	↑↑	↑	↑↑	↑
**Metabólica**						
	Oxidação de gordura	Mais alta	-	-	-	-
	Oxidação de carboidratos	Mais baixa	-	-	-	-
	Pico de lactato sanguíneo	Mais baixo	-	-	-	-
	Capacidade glicolítica	Mais baixa	-	-	-	-
	Capacidade alática	Mais baixa	-	-	-	-
	Depuração de lactato	Mesma	-	-	-	-

↑ = aumento;

↑↑ = aumento de maior magnitude;

FC: frequência cardíaca; VO_2_: consumo de oxigênio; O_2_: oxigênio; VEmin: ventilação por minuto; VE: ventilação pulmonar; VCO_2_: produção de dióxido de carbono.

* Independente da intensidade do esforço.

** Equivalentes ventilatórios que determinam a eficiência ventilatória.

##### 6.1.2.1. Consumo de Oxigênio (VO_2_)

A ACR através da medida direta do VO_2_pico ou VO_2_max no TCPE é considerada a principal variável metabólica ao esforço. O VO_2_ nos limiares ventilatórios (principalmente no primeiro limiar ventilatório – LV1) tem importância diagnóstica e prognóstica em crianças e adolescentes. O VO_2_ no LV1 e o VO_2_max são geralmente superiores aos observados em adultos.^1 , 177 , 286 , 594^

No caso de um esforço máximo, a ACR da criança pode ser avaliada através do VO_2_pico (mL/kg/min), considerado dentro dos limites da normalidade quando ≥2 DP. Em adolescentes, não se recomenda adotar o valor de 80% do VO_2_max previsto como limite inferior da normalidade, pois poderia estar superestimado.

A capacidade anaeróbica de crianças é menor do que em adultos, mesmo que seja expressa por unidade de massa corporal total ou magra.

Em testes submáximos, nem sempre é possível avaliar a ACR com base no VO_2_pico. Outros parâmetros do TCPE, como o LV1 e a inclinação da eficiência do VO_2_, podem ser usados para fornecer uma melhor definição da aptidão.

O descondicionamento físico geralmente é caracterizado como capacidade reduzida de transporte de oxigênio pelo sistema CV e/ou eficiência reduzida na extração periférica de oxigênio acarretando um LV1 precoce. A observação de LV1 <50% do VO_2_max previsto está associada ao descondicionamento físico e <40% geralmente está relacionada a doença com comprometimento significativo da ACR.^1 , 177 , 286 , 594^

##### 6.1.2.2. Pulso de Oxigênio (PuO_2_)

O pulso de O_2_ (PuO_2_ = VO_2_/FC) é uma variável não invasiva que reflete o débito cardíaco, sendo útil na avaliação da disfunção ventricular, com ou sem isquemia associada. Normalmente, o PuO_2_ aumenta com o esforço devido ao incremento linear da FC e VO_2_, estabilizando-se em platô próximo ao esforço máximo.^1 , 177 , 286 , 594^

A diminuição no PuO_2_ (normal ≥2 DP) em cargas submáximas sugere disfunção ventricular, sendo indicativo de um volume sistólico ejetado reduzido. Essa diminuição, associada à queda no ΔVO_2_/ΔWR, indica disfunção ventricular grave, frequentemente de natureza isquêmica.

No TCPE, a combinação de PuO_2_ diminuído no pico do esforço (<2 DP do previsto), LV1 diminuído (<40 a 50% do VO_2_max previsto), VO_2_pico diminuído e aumento rápido da FC, pode estar associada ao descondicionamento físico.

##### 6.1.2.3. Quociente Respiratório (Relação VCO_2_/VO_2_)

Na população pediátrica o quociente respiratório (QR) em repouso varia de 0,70 a 0,85. Durante o esforço progressivo, após LV1, o VCO_2_ aumenta desproporcionalmente em relação ao VO_2_, o que se traduz em um aumento no QR, em função das modificações de substratos energéticos. É fundamental que o QR seja avaliado no ponto do VO_2_pico, pois os seus valores continuam a aumentar após a interrupção do esforço, inclusive no início da fase de recuperação. Quando o QR é ≥1,1, o TCPE preenche as condições para ser considerado máximo.^1 , 177 , 286 , 594^

Em populações pediátricas, a FCpico e o QR no pico do esforço (QRpico) são recomendados como critérios objetivos para avaliar a qualidade do esforço realizado. Considera-se como ideal:

– Atingir FC ≥180 bpm no VO_2_pico (ou pelo menos ≥95% da FCmax prevista).
– Atingir QR de pelo menos 1,00 no VO_2_pico. Esse valor representa o limite inferior da normalidade no TCPE em bicicleta ergométrica.

O QR no VO_2_pico ≥1,00 caracteriza utilização exclusiva de carboidrato (glicose), fornecendo energia por meio de metabolismo predominantemente anaeróbico. Valores de QR <1,00 no VO_2_pico podem indicar um esforço submáximo ou podem ser patológicos (por exemplo: pneumopatia; CC cianótica descompensada; doença de armazenamento de glicogênio). Em crianças e adolescentes aparentemente saudáveis, os valores de QR diminuirão dentro de 2 a 3 minutos na recuperação.^1 , 177 , 286 , 594^

##### 6.1.2.4. Inclinação da Eficiência da Captação do Oxigênio

A inclinação da eficiência da captação do oxigênio (OUES: do inglês *oxygen uptake efficiency slope* ) é uma relação não linear da resposta ventilatória ao esforço, correspondendo ao aumento absoluto do VO_2_ associado ao aumento da ventilação pulmonar (VE). Expressa a eficiência da extração alveolar do O_2_ no ar ventilado. Sugere-se apresentar os valores de OUES relativos à ASC, peso ou à massa livre de gordura.^612 – 621^ O Anexo 4 apresenta informações sobre os valores/percentis de OUES e equações de predição para população pediátrica aparentemente saudável.^622^

Um estudo brasileiro envolvendo crianças saudáveis e portadores de CC sugere o uso de OUES indexada ao peso (OUES/kg) e propôs que o valor de OUES >35 está relacionado com capacidade funcional normal.^623^ Entretanto, estudo multicêntrico internacional encontrou pontos de corte do valor de OUES de 38,4 para meninos e 31,0 para meninas.^624^

O valor de OUES submáximo correlaciona-se com o VO_2_pico, VEpico e VO_2_ do LV1, sendo uma medida válida para a determinação da ACR e a estratificação de risco em testes submáximos.^613 , 625 , 626^

##### 6.1.2.5. Equivalente Ventilatório de O_2_ e CO_2_

No TCPE, os equivalentes ventilatórios de O_2_ (VE/VO_2_) e de CO_2_ (VE/VCO_2_) indicam, respectivamente, a ventilação por minuto (VEmin) necessária para consumir 1 L/min de O_2_ e produzir/eliminar 1 L/min de CO_2_. Durante o esforço progressivo, a razão VE/VO_2_ diminui até o LV1, a partir do qual aumenta progressivamente, com inflexões positivas nas curvas no LV1 e LV2. A razão VE/VCO_2_ diminui até o LV2, aumentando em seguida.

Os equivalentes ventilatórios contribuem para a avaliação da eficiência cardiorrespiratória, identificação dos limiares ventilatórios, tendo valor diagnóstico e prognóstico na população pediátrica com CC, IC e HAP. Anormalidades cardiocirculatórias com baixo débito cardíaco apresentam uma inclinação acentuada na curva de VE/VCO_2_. A relação VE/VO_2_ costuma estar elevada na IC.

Em estudo com 700 pacientes (entre 5 e 18 anos), divididos em aparentemente saudáveis e portadores de CC, a inclinação da curva de VE/VCO_2_ foi significativamente maior nos cardiopatas (maior aumento nos pacientes com obstrução de via de saída de VD). Esse estudo sugere 29 como ponto de corte de normalidade.^624 , 627^

##### 6.1.2.6. Outras Considerações sobre Variáveis Ventilatórias e Metabólicas^1 , 177 , 286 , 594^

A ventilação por minuto (VEmin) aumenta com o progredir do esforço, dependendo da intensidade da carga imposta, do condicionamento físico e relacionando-se ao VO_2_ e VCO_2_.

A FR excessiva pode ser indicativa de sedentarismo ou de anormalidades na mecânica ventilatória. A FR nas crianças costuma ser maior do que a observada nos adultos, sendo de ≈65 incursões respiratórias/min em crianças de 5 a 8 anos e ≈50 a 55 incursões respiratórias/min em crianças >11 anos.

Em comparação com os adultos, as crianças apresentam uma maior relação entre a FR e o volume corrente (VC), geralmente associada à redução da ventilação/perfusão. Essa situação é comumente observada em algumas CC cianóticas.

A limitação ventilatória é tradicionalmente definida por reserva ventilatória (RV) <20% durante o esforço. Crianças saudáveis têm RV ≥11 L/min ou de 20 a 40% de sua ventilação voluntária máxima (VVM).^179 , 628^

Equações de predição da RV:

$$\begin{array}{c} VVM=VEF1\;x\;\times 35\\ RV=\frac{VVM-VEmax}{VVM}x100 \end{array}$$

VVM: ventilação voluntária máxima

VEF1: volume expiratório forçado em um segundo

RV: reserva ventilatória

VEmax: ventilação máxima de esforço

A RV contribui para o diagnóstico diferencial entre doença cardíaca e doença pulmonar. A RV baixa é característica de doença pulmonar primária e doença pulmonar obstrutiva, enquanto a RV elevada ocorre em condições CV que limitam o desempenho físico.^629^

Em geral, as crianças com doenças pulmonares restritivas têm capacidade de esforço reduzida (baixo VO_2_pico e baixo VO_2_ no LV1) e aumento do VC (50% da capacidade vital e/ou 80% da capacidade inspiratória), com RV relativamente baixa.^630^ Qualquer aumento adicional na VEmin é devido a um aumento da FR. Se houver limitação ventilatória durante o esforço, a saturação arterial de oxigênio (SpO_2_) diminui com o aumento de carga de trabalho.^1 , 177 , 286 , 594^

Na HAP, ocorre redução acentuada da eficiência ventilatória, com relações VE/VO_2_ e VE/VCO_2_ elevadas, indicando trocas gasosas anormais nos pulmões.^631^

O PETO_2_ e PETCO_2_ refletem as tensões gasosas arteriais. O PETCO_2_ baixo associado a elevações do PETO_2_ e do QR indica hiperventilação.

Queda ≥5% na SpO_2_ durante o TE/TCPE é definida como uma hipoxemia esforço-induzida. Queda de 10 pontos em relação à saturação de repouso associada a sintomas ou SpO_2_ <85% são critérios para interrupção do esforço. A dessaturação é considerada grave quando a SpO_2_ for <80% e estiver acompanhada de sinais e sintomas de hipoxemia grave, ocorrendo geralmente em crianças com doenças pulmonares graves ou IC.^260 , 594^

### 7. Elaboração do Laudo do TCPE em Crianças e Adolescentes

O laudo do TCPE de crianças e adolescentes deve seguir o mesmo padrão de estruturação do laudo de adulto apresentado na Diretriz Brasileira de Ergometria em População Adulta – 2024.^1^

No TCPE, o laudo deve conter, resumidamente, as principais variáveis ergoespirométricas (hemodinâmicas, ventilatórias e metabólicas), com descrição das alterações que foram determinantes para a interrupção do esforço e eventuais diagnósticos e prognósticos.

No laudo, devem constar:

– A apresentação da FC, PA, comportamento eletrocardiográfico, VO_2_ e equivalente metabólico (MET), relacionando-os aos valores previstos para a faixa etária e sexo.
– A apresentação do limiar anaeróbio ventilatório (LV1) normalizado para a massa corporal (expresso como porcentagem do VO_2_pico atingido e VO_2_max previsto), relacionando-o à FC e carga de esforço.
– ACR aferida e sua repercussão em relação às indicações e achados do exame.
– Quando pertinente, apresentar os valores de normalidade utilizados em relação ao sexo, idade, peso, IMC e presença ou não de doenças.

Observação: as informações referidas nos itens anteriores têm grande relevância diagnóstica e prognóstica, sendo aplicáveis na prescrição de exercícios físicos, particularmente na RCV.

## Parte 3 – Particularidades do TE/TCPE em Condições Clínicas Específicas

### 1. Cardiopatias Congênitas e Cardiopatias Adquiridas

Dentro das indicações mais comuns do TE/TCPE na população infantil e de adolescentes/adultos jovens, estão as abordagens clínicas, hemodinâmicas e eletrocardiográficas em portadores de CC, especialmente após a correção parcial ou completa do defeito cardíaco. A ACR pode estar baixa em pacientes com CC complexas (mesmo em pacientes supostamente assintomáticos), especialmente na hipertensão arterial pulmonar (HAP) e IC crônica.^77 , 632 , 633^

A Tabela 35 apresenta o comportamento das principais variáveis do TE/TCPE em relação às doenças CV mais prevalentes na faixa etária pediátrica.

**Tabela 35:** Principais variáveis do TE/TCPE e seu comportamento nas doenças cardiovasculares na população pediátrica

Variável	Interpretação	Doenças cardiovasculares
FCmax	↓ na incompetência cronotrópica	CC corrigida; SQTL; transplante cardíaco.
	↓ em uso de betabloqueador/antiarrítmico	CC/CM com insuficiência cardíaca; arritmia.
Pressão arterial sistólica	↓ na disfunção ventricular	CC; CM; CM hipertrófica; HAP.
	↑ na resposta hipertensiva	Coarctação da aorta; hipertensão essencial.
ECG	Arritmia esforço-induzida	CC; arritmias primárias; BAVT.
	Alterações isquêmicas da repolarização	Doença de Kawasaki; anomalias coronarianas (congênitas ou pós-reparo).
	Outras alterações induzidas pelo esforço	SQTL; Síndrome de Brugada; WPW.
VO_2_max/VO_2_pico	↓ na disfunção cardiopulmonar e/ou descondicionamento físico	CC; CM; HAP; potenciais receptores de transplante cardíaco; bloqueio atrioventricular total.
Pulso de oxigênio	↓ na disfunção ventricular e isquemia miocárdica	CC; IC; CCPT; CM; doença de Kawasaki; anomalias coronarianas e valvares.
Saturação de oxigênio	↓ na doença pulmonar, *shunts* cardíacos e/ou pulmonares	CC cianótica; IC; asma brônquica; fibrose pulmonar.
VE/VO_2_ e VE/VCO_2_	↑ na ineficiência ventilatória (anormalidades da ventilação/perfusão)	CC com insuficiência cardíaca ou *shunt* direita-esquerda; Fallot operado; HAP.

↓ = diminui;

↑ = aumenta;

CC: cardiopatia congênita; CM: cardiomiopatia, ECG: eletrocardiograma; SQTL: síndrome do QT longo; HAP: hipertensão arterial pulmonar; CCPT: conexão cavopulmonar total; VE: ventilação minuto; VCO_2_: produção de dióxido de carbono; VO_2_: consumo de oxigênio; WPW: Síndrome de Wolff-Parkinson-White; VO_2_max: consumo máximo de oxigênio (mL/kg/min); VO_2_pico: VO_2_ obtido nos exames que não houver as características de um esforço máximo; FCmax: frequência cardíaca máxima; VE/VCO_2_: equivalente ventilatório de dióxido de carbono; VE/VO_2_: equivalente ventilatório de oxigênio; VO_2_max: BAVT: bloqueio atrioventricular total; IC: insuficiência cardíaca. Adaptado de: Massin MM. The role of exercise testing in pediatric cardiology.^6^

#### 1.1. Defeitos do Septo Atrial

Nos defeitos do septo atrial/comunicação interatrial (DSA) a maioria dos pacientes permanece assintomática durante a maior parte da infância, mesmo em presença de grande *shunt* esquerda para a direita (esquerda-direita). São cinco os tipos principais de DSA: *ostium secundum, ostium primum* , seio venoso, defeitos do seio coronário e forame oval patente. Esses defeitos serão tratados como uma entidade única (DSA) em relação ao TE, pois os sintomas, comportamento das variáveis e interpretações são semelhantes, dependendo do predomínio do *shunt* (se direita-esquerda ou esquerda-direita), tamanho do defeito e presença de HAP e/ou IC.^634 , 635^

Particularidades do ECG de repouso na DSA em população pediátrica:^370 , 388 , 636 , 637^

– Na maioria dos pacientes, a amplitude e a duração da onda P são normais. No *ostium secundum* , costumam ocorrer ondas P apiculadas em DII por aumento do átrio direito.
– No *ostium secundum* e nos *shunts* esquerda-direita significativos, pode ocorrer prolongamento do PRi (BAV de 1 ° grau) e atraso na condução intraventricular (padrão de BRD) associado à HVD.
– Após o reparo cirúrgico de DSA tipo *ostium secundum* , geralmente ocorre diminuição da duração e dispersão da onda P, sem atingir os níveis normais.
– Após reparo transcateter, observa-se regressão parcial ou completa das anormalidades do ECG na maioria dos pacientes.^638 , 639^
– Após reparo cirúrgico de defeitos do tipo seio venoso, observa-se taxa relativamente alta de DNS (6%) e FA (14%).^640^

Particularidades do TE/TCPE no DSA não corrigido:

– Crianças geralmente têm ACR preservada.^641^
– Adolescentes e adultos jovens podem apresentar redução da ACR, principalmente quando sintomáticos. Nesses pacientes, observa-se redução de até 60% do VO_2_max previsto.^610^
– Pacientes com DSA assintomáticos, sem sobrecarga de volume e função normal de VD em repouso, podem apresentar aumento significativo da pós-carga e/ou disfunção do VD esforço-induzidas.^642^
– A inclinação VE/VCO_2_ geralmente é normal. Entretanto, nos pacientes com DSA associado à IC, disfunção do VD, HAP e/ou doença pulmonar, a inclinação pode aumentar devido à incompatibilidade ventilação-perfusão.^610 , 627^

Particularidades do TE após o reparo do DSA:

– No reparo cirúrgico precoce após 6 meses, observa-se ACR normal, que se mantém durante a vida adulta.^643 – 645^
– Sintomas associados às arritmias e/ou dispneia esforço-induzidas são raros e determinantes da gravidade da CC.^646 , 647^
– A ocorrência de resposta cronotrópica deprimida (incompetência cronotrópica) é mais frequente após reparo cirúrgico do que após reparo transcateter.^589 , 590^
– Após o reparo cirúrgico, a capacidade aeróbica geralmente é reduzida e o desempenho do VD é significativamente menor.^648^
– Reparos cirúrgicos em idade mais avançada (adolescência) e/ou em presença de HAP geralmente evoluem com menor ACR e maior incidência de arritmias atriais esforço-induzidas.^649 , 650^
– Arritmias esforço-induzidas em crianças após reparo da DSA são raras, podendo manifestar-se como bradicardia sinusal, taquicardia sinusal, taquicardia supraventricular, batimentos atriais prematuros, batimentos prematuros ventriculares, DNS, BAV, *flutter* atrial e FA.^651 – 654^
– Outras particularidades encontram-se na Tabela 36 .

**Tabela 36:** Comportamento das principais variáveis do TE/TCPE nos defeitos do septo atrial/comunicação interatrial reparados e não reparados

Parâmetros TE/TCPE	DSA não reparado	DSA reparado
ECG de repouso	Geralmente, observam-se aumento da duração e amplitude da onda P, aumento do PRi e duração do QRS (padrão BRD).^655 , 656^ Raramente ocorre BAV (primeiro e segundo grau).	– No reparo cirúrgico e transcateter, observam-se: redução da duração da onda P, da dispersão da onda P, do PRi, da duração QRS e dispersão QT.^656 – 660 , 661^ – BAV em 2 a 4% dos pacientes, incluindo BAVT.^662^ – Reparo cirúrgico frequentemente evolui com arritmias cardíacas (precoce e tardiamente), benignas e/ou significativas (requerendo terapia farmacológica).^663^
Sintomas esforço-induzidos	Raros em crianças, mas, quando presentes, estão relacionados à gravidade de HAP e/ou IC.^634^ Mais comuns em adolescentes e adultos, podendo comprometer a aptidão cardiorrespiratória.	– Geralmente, apresenta melhora dos sintomas. – A persistência depende de HAP, IC residuais e/ou CC complexa parcialmente corrigida.^664^
VO_2_max (mL/kg/min)	Normal em crianças, a menos que tenham descondicionamento físico. Diminuído, dependente da gravidade de HAP e/ou IC.	– Normal após >6 meses do reparo. – Melhora parcial na persistência de descondicionamento físico, HAP e/ou IC.^665 – 667^
FCmax	Normal. Na mutação NKX 2.5 (rara), pode ocorrer bradiarritmia por disfunção do nó sinusal e/ou disfunção do nó atrioventricular.^668^	– Geralmente normal. – A incompetência cronotrópica é rara, geralmente no reparo cirúrgico ou na mutação NKX 2.5.^589 , 651^
Arritmia esforço-induzida	Rara em crianças. Mais frequente em adolescentes e/ou na HAP.	– Redução nas correções precoces.^669^ – Mantida mais frequentemente nas correções em adolescentes.^670^
Oximetria de pulso (SpO_2_, %)	Diminuída no *shunt* direita-esquerda e/ou na HAP.	– Normal, a menos que haja *shunt* residual com HAP.
Ventilação por minuto (VE, L/min)	Aumentada.	– Normal, a menos que haja HAP ou IC.
Pulso de oxigênio (mL)	Normal; diminuído na IC.	– Normal; diminuído na persistência de IC.
VE/VO_2_	Aumentada.	– Normal, a menos que haja HAP ou IC.
VE/VCO_2_	Aumentada.	– Normal, a menos que haja HAP ou IC.
Pressão parcial de CO_2_ expirado (PETCO_2_, mmHg)	Normal; diminuído no *shunt* direita-esquerda.	– Normal; diminuído no *shunt* direita-esquerda residual.

CC: cardiopatia congênita; CM: cardiomiopatia, ECG: eletrocardiograma; HAP: hipertensão arterial pulmonar; IC: insuficiência cardíaca; BRD: bloqueio de ramo direito; VE/VCO_2_: equivalente ventilatório de dióxido de carbono; VE/VO_2_: equivalente ventilatório de oxigênio; VO_2_max: consumo máximo de oxigênio; PRi: intervalo PR; TE: teste ergométrico; TCPE: teste cardiopulmonar de exercício; DSA: defeito do septo atrial; BAV: bloqueio atrioventricular; BAVT: bloqueio atrioventricular total; FC: frequência cardíaca. Adaptado de: Amedro et al. Atrial septal defect and exercise capacity: value of cardio-pulmonary exercise test in assessment and follow-up.^610^

#### 1.2. Defeito do Septo Ventricular

A ausculta cardíaca no pré-teste pode permitir a detecção dos defeitos do septo ventricular (DSV). Os sopros são tipicamente descritos como holossistólicos (pansistólicos). O grau do sopro depende da velocidade do fluxo, com defeitos menores sendo mais ruidosos e podendo causar frémito.^671^

O ECG de repouso geralmente reflete o grau de anormalidade hemodinâmica do DSV:^388 , 672 , 673^

– Se normal, sugere pequeno DSV, isolado e com pequeno *shunt* esquerda-direita.
– O padrão de HVE com aumento de átrio esquerdo indica um *shunt* esquerda-direita moderado/grave, mas sem HAP.
– O padrão combinado de HVE e HVD, com complexos QRS bifásicos, de grande amplitude nas derivações periféricas e precordiais médias (padrão de Katz-Wachtel), é frequentemente encontrado em pacientes com DSV grande e grau variável de HAP.
– Na HAP acentuada (exemplo: síndrome de Eisenmenger), observa-se predomínio de padrão de HVD, desvio do eixo do QRS para direita e aumento do átrio direito.
– Aproximadamente 10% dos pacientes com DSV apresentam BRD (completo ou incompleto).
– Mesmo em pacientes com pequenos DSV, o risco de arritmia grave e morte súbita é maior do que em crianças aparentemente saudáveis.
– A minoria dos pacientes submetidos a reparo transcateter do DSV perimembranoso pode evoluir com BRD, BDASE e BAVT.

Particularidades do DSV no TE/TCPE:

– DSV pequenos na população pediátrica geralmente apresentam *shunt* esquerda-direita hemodinamicamente insignificante, inclusive durante o esforço, não comprometendo significativamente a capacidade funcional.^674^
– Crianças com comunicação interventricular (CIV) patente ou reparada cirurgicamente geralmente têm ACR normal, apesar de discreto comprometimento da resposta cronotrópica.^9 , 675^
– Adultos jovens com DSV pequenos não reparados na infância podem evoluir com ACR comprometida relacionada ao tamanho do *shunt* e disfunção biventricular.^676 , 677^
– O reparo cirúrgico de DSV importante nos primeiros 2 anos de vida reduz o risco de persistência de sintomas e de desenvolvimento de anormalidades cardiopulmonares, secundários à disfunção ventricular e/ou doença vascular pulmonar progressiva.^678^
– HAP antes do reparo e/ou persistente após o reparo reduz a tolerância ao esforço e piora a qualidade de vida.^679 , 680^
– Na síndrome de Eisenmenger, geralmente observa-se comprometimento acentuado da ACR e risco aumentado de morte súbita.^681 , 682^
– Outras particularidades encontram-se na Tabela 37 .

**Tabela 37:** Comportamento das principais variáveis do TE/TCPE no defeito do septo ventricular reparado e não reparado

Parâmetros TE/TCPE	DSV não reparado	DSV reparado
Alterações hemodinâmicas com maior risco de complicações esforço-induzidas	Grande *shunt* esquerda-direita; dilatação VE, com função de VE comprometida; insuficiência aórtica; doença vascular pulmonar/HAP; síndrome de Eisenmenger.	– *Shunt* residual; IC; insuficiência aórtica; obstrução da via de saída do VD ou VE; persistência de HAP; síndrome de Eisenmenger.
ECG de repouso	Geralmente, reflete o grau de anormalidade hemodinâmica do DSV (vide texto).	– Reparo transcateter do DSV perimembranoso, raramente evolui com BRD, BDASE e BAVT.^683 , 684^ – No reparo cirúrgico, é frequente a ocorrência de BRD relacionado à disfunção de VD e disfunção diastólica do VE.^685 – 687^ – A arritmia ventricular é frequente e sua prevalência aumenta com a idade na época do reparo e com tempo de seguimento;^688^ – O BAVT é raro no reparo cirúrgico e frequente no reparo transcateter.
Sintomas esforço-induzidos	O DSV pequeno* e sem insuficiência aórtica geralmente é assintomático;^675^ Grandes defeitos, HAP, IC e/ou síndrome de Eisenmenger são sintomáticos.	– Após reparo geralmente assintomático. – Geralmente sintomático na persistência da HAP, IC, insuficiência aórtica, obstrução de via de saída de VD ou VE e na síndrome de Eisenmenger.
VO_2_max	Na CIV patente pequena geralmente normal; Reduzido nos defeitos grandes, HAP e/ou síndrome de Eisenmenger.	– Após reparo geralmente normal. – O reparo transcateter em adolescentes assintomáticos ou minimamente sintomáticos previne a deterioração da aptidão cardiorrespiratória e promove a remodelação reversa do VE.^689^ – Reduzido na persistência da HAP, IC, insuficiência aórtica, obstrução de via de saída de VD ou VE e na síndrome de Eisenmenger.
FCmax	Geralmente discreto comprometimento da resposta cronotrópica. A disfunção do nó sinusal requerendo colocação de marca-passo ocorre em 4% dos pacientes.	– No reparo transcateter geralmente normal. – No reparo cirúrgico, pode evoluir com menor FCmax e incompetência cronotrópica.^407^
Arritmia esforço-induzida	Rara em defeitos pequenos. Frequente nos grandes defeitos, IC, HAP e síndrome de Eisenmenger.	– Redução nas correções precoces. – Frequente quando ocorre arritmia complexa após reparo transcateter, na HAP residual, na disfunção ventricular persistente e no BRE após o reparo.^683 , 690^
Oximetria de pulso	Normal nos pequenos defeitos. Diminuída nos grandes defeitos, no *shunt* direita-esquerda, na síndrome de Eisenmenger e/ou na HAP.	– Normal, a menos que haja persistência da HAP ou na síndrome de Eisenmenger.
LV1	Geralmente reduzido.^691^	– Após reparo apresenta aumento, podendo tornar-se normal. – Mantém-se reduzido na persistência da HAP, IC, insuficiência aórtica e na síndrome de Eisenmenger.
VEmin (L/min)	Geralmente reduzida.	– Pode manter-se reduzida mesmo após reparo devido à esternotomia com restrição da complacência da caixa torácica, exposição prolongada das pequenas vias aéreas ao alto fluxo sanguíneo pulmonar e alterações das propriedades viscoelásticas do pulmão.^691^
VE/VO_2_	Aumentada nos grandes defeitos e na síndrome de Eisenmenger.	– Normaliza, a menos que haja HAP ou IC.
VE/VCO_2_	Aumentada nos grandes defeitos e na síndrome de Eisenmenger.	– Normaliza, a menos que haja HAP ou IC.

FCmax: frequência cardíaca máxima; HAP: hipertensão arterial pulmonar; IC: insuficiência cardíaca; BRD: bloqueio de ramo direito; BRE: bloqueio de ramo esquerdo; VEmin: ventilação por minuto; VO_2_max: consumo máximo de oxigênio; VE/VCO_2_: equivalente ventilatório de dióxido de carbono; VE/VO_2_: equivalente ventilatório de oxigênio; VD: ventrículo direito; VE: ventrículo esquerdo; TE: teste ergométrico; TCPE: teste cardiopulmonar de exercício; DSV: defeito do septo ventricular; BDASE: bloqueio divisional anterossuperior esquerdo; BAVT: bloqueio atrioventricular total; CIV: comunicação interventricular; FC: frequência cardíaca; LV1: primeiro limiar ventilatório. *DSV pequenos com *shunt* esquerda-direita <50%, sem sinais de sobrecarga de volume do VE e pressão da artéria pulmonar normal.

#### 1.3. Persistência do Canal Arterial

As manifestações clínicas da persistência do canal arterial (PCA) dependem principalmente da quantidade de fluxo sanguíneo da aorta para a artéria pulmonar e ocorrência de HAP secundária.^692^

O TE/TCPE contribui com o acompanhamento clínico e com as decisões terapêuticas nas várias formas e apresentações da PCA:^693 , 694^

– Nas "silenciosas" (inaudíveis) e pequenas (pequeno *shunt* esquerda-direita, sem repercussão hemodinâmica), para confirmação da condição assintomática, esclarecimento de sintomas esforço-induzidos e de alterações eletrocardiográficas.
– Nas com repercussão hemodinâmica ou com HAP leve/moderada, a cada 12 a 24 meses, como parte do acompanhamento clínico seriado e adjuvante na decisão de intervenções.
– Caso haja HAP, realizar o exame para verificar a ocorrência de dessaturação dos membros inferiores, um critério de gravidade e possível contraindicação para o fechamento do canal.^695 – 697^
– Adolescentes e adultos jovens com PCA grave (com aumento de câmara do lado esquerdo, HAP grave e contraindicação para o fechamento do canal arterial) e/ou síndrome de Eisenmenger devem realizar o exame a cada 6 a 12 meses, para ajustes terapêuticos da IC e/ou HAP.
– Nas crianças e adolescentes com PCA grave e que evoluíram para IC avançada, o TCPE contribui particularmente na indicação de transplante cardíaco.
– Após correção, visando avaliar a persistência de sintomas, PCA residual, HAP residual e complicações cirúrgicas, tais como obstrução da artéria pulmonar esquerda e coarctação de aorta.
– Na avaliação pré-participação, para exercícios e esportes em pacientes com PCA silenciosa/pequena ou após correção evoluindo sem HAP.^695^

O exame físico pré-teste dos pacientes com PCA não corrigida varia de acordo com o tamanho do canal arterial e sua repercussão. Na PCA silenciosa o exame físico é normal.^698^

O ECG de repouso na PCA:^370 , 388^

– Nos *shunts* menores, o ECG geralmente é normal.
– Nos *shunts* moderados/grandes, geralmente observam-se taquicardia sinusal ou FA, sobrecarga atrial esquerda (SAE), HVE e ISTs.^698^
– Quando grande e com HAP, apresenta frequentemente sinais de dilatação do átrio direito e hipertrofia biventricular.
– Normalmente ocorre ritmo sinusal e BAV de I grau em ≈10% dos casos. Raramente observam-se BAV de II grau, BRE e BRD.

Particularidades do TE/TCPE na PCA em crianças e adolescentes:

– Nas silenciosas, geralmente são assintomáticos, sem sequelas hemodinâmicas ou anatômicas, com função pulmonar e ACR normais. Raramente apresentam intolerância ao esforço ou têm doença reativa das vias aéreas esforço-induzida.^693^
– PCA com HAP geralmente apresenta comprometimento significativo da capacidade aeróbica, queda da saturação de oxigênio com o esforço (geralmente >10%), *redução dos* valores de VO_2_pico e menor inclinação de VE/VCO_2_, correlacionados diretamente com a gravidade da HAP. Os sintomas esforço-induzidos mais frequentes são dispneia, dor torácica, tontura e palpitação (arritmia ventricular).^197 , 377 , 699^
– É necessária a monitorização da SpO_2_ nas extremidades superiores e inferiores, inclusive para a confirmação de ocorrência de dessaturação dos membros inferiores esforço-induzida.^695^
– Após correção cirúrgica, assintomáticos geralmente apresentam FCpico menor do que os aparentemente saudáveis. Em alguns pacientes, pode ocorrer incompetência cronotrópica.^700^
– Assintomáticos após correção (transcateter ou cirúrgica), sem evidências de cardiopatia estrutural (doença valvar, arritmia ou hipertrofia ventricular) e de doença pulmonar, geralmente apresentam comportamento pressórico normal e ACR preservada.^700^
– Pacientes após correção cirúrgica complicada por paralisia da prega vocal esquerda podem apresentar estridor laríngeo grave e obstrução laríngea esforço-induzidos.^701 , 702^
– Nascidos extremamente prematuros (<28 semanas de gestação ou peso ao nascer <1.000 g) e submetidos a correção cirúrgica, quando adolescentes, podem apresentar, ao TCPE, função pulmonar e ACR reduzidas.^701^

#### 1.4. Tetralogia de Fallot

A tetralogia de Fallot [(do inglês *tetralogy of Fallot* (ToF)] clássica consiste em um grupo de quatro defeitos: CIV; estenose pulmonar; hipertrofia VD; aorta cavalgante conectada tanto no VE quanto no VD. Existem variações da apresentação, incluindo ToF com atresia pulmonar e com agenesia da válvula pulmonar.^703 , 704^

Os desfechos de longo prazo na ToF reparada são muitos e graves, exigindo acompanhamento regular.^705^ A incidência de MSC arrítmica é estimada em 1 a 5%. Os principais fatores associados são: duração do QRS >180 ms; disfunção sistólica ou diastólica do VE; ventriculectomia; pressão diastólica final do VE ≥12 mmHg; história de arritmia supraventricular; taquicardia ventricular não sustentada (TVNS); TV induzível no estudo eletrofisiológico (EEF).^388 , 706 – 708^

O TE/TCPE apresenta papel relevante no seguimento, estratificação de risco, decisões terapêuticas e avaliação da repercussão das complicações pós-cirúrgicas: insuficiência pulmonar residual; insuficiência aórtica (IAo); dilatação e/ou disfunção do VD; estenose residual da artéria pulmonar; obstrução da via de saída do VD; arritmias complexas; IC.^100 , 709^

O exame físico no pré-teste da ToF reparada é importante para investigação de lesões anatômicas residuais e avaliar condições de risco potencial de complicações durante o exame.^370^

Particularidades do ECG de repouso na ToF reparada:^388 , 706 – 708 , 710^

– O aumento do átrio direito é observado em ≈30 a 50% dos pacientes.
– O padrão mais prevalente é o de BRD com ou sem BDASE. Geralmente, o BRD é assintomático e não requer intervenção.^711 , 712^
– A duração do QRS >150 ms está associada à disfunção de VD e insuficiência valvar pulmonar significativa no pós-operatório tardio.
– Arritmias supraventriculares, incluindo distúrbios de condução sinoatrial, FA e *flutter* atrial são encontradas em um terço dos pacientes.
– São frequentes as arritmias ventriculares, incluindo TVNS.

Particularidades do TE/TCPE na ToF ( Tabela 38 ):

**Tabela 38:** Comportamento das principais variáveis do TE/TCPE na ToF reparada e suas repercussões^100 , 716^

Parâmetros TE/TCPE	Comportamento	Interpretação/repercussão
Complicações pós-cirúrgicas com maior risco de eventos no exame	Insuficiência pulmonar residual; insuficiência aórtica; dilatação e/ou disfunção do VD; estenose residual da artéria pulmonar; obstrução da via de saída do VD; arritmias complexas; IC.^717^	– Associadas a sintomas esforço-induzidos e risco de complicações no exame: dessaturação importante, hipotensão, congestão/IC, arritmias complexas, pré-síncope e síncope.
VO_2_max	Geralmente, o VO_2_max e o VO_2_ no LV1 estão reduzidos;* A média da %VO_2_pico prevista é de 68±2,8% (IC95%: 62,3-74%).^716^ Na resposta cronotrópica normal, o VO_2_max apresenta-se menos reduzido. Redução mais acentuada é observada na persistência da HAP, IC, insuficiência aórtica, obstrução de via de saída de VD. A insuficiência pulmonar residual e a idade do reparo influenciam na redução do VO_2_max.^718^	– A aptidão cardiorrespiratória pode ser limitada, apesar da melhora na classe funcional (NYHA) após a correção.^719^ – Crianças do sexo masculino geralmente apresentam pior aptidão cardiorrespiratória.^720^ – Disfunção do VD e do VE relacionam-se linearmente à redução do VO_2_max.^721^ – VO_2_pico baixo ou limítrofe é útil na estratificação de risco de adolescentes e adultos jovens assintomáticos considerados para a troca da válvula pulmonar.^100^
Frequência cardíaca máxima	Geralmente é menor do que em crianças saudáveis, sendo frequente a incompetência cronotrópica. A disfunção grave do nó sinusal ocorre em 4% dos pacientes.^591^	– A reposta cronotrópica normal associa-se com maior aptidão cardiorrespiratória, independentemente da função sistólica de VD e/ou insuficiência pulmonar.^252 , 714^
Arritmia ventricular esforço-induzida	Geralmente relacionada ao reparo tardio e à função ventricular direita deprimida.	– Associa-se a alterações hemodinâmicas residuais importantes e risco aumentado de eventos cardiovasculares.
Oximetria de pulso	Normal nos pequenos defeitos residuais. Diminuída nos grandes defeitos, no *shunt* direita-esquerda, na síndrome de Eisenmenger e/ou na HAP e IC.	– Maior risco de eventos cardiovasculares e pior prognóstico.
Pulso de O_2_	Geralmente, mantém-se diminuído (em ≈85,3% dos casos). Em ≈10,3% dos pacientes, aumenta; e em ≈4,4%, ocorre redução adicional.^715 , 716^	– A manutenção da redução está associada a menor fração de ejeção do VD e aos menores volumes sistólicos biventriculares.^722^
VE/VCO_2_	Normalmente, um pouco aumentada. Na IC observa-se grande aumento.	– O aumento associa-se a redução do débito cardíaco, HAP e pior prognóstico.
OUES	Normalmente o valor está pouco alterado, mas com redução importante do VO_2_max. Valores de OUES mais baixos estão associados a disfunção ventricular.^715^	– Quando normal, indica razoável capacidade submáxima de esforço.

HAP: hipertensão arterial pulmonar; IC: insuficiência cardíaca; VD: ventrículo direito; VO_2_max: consumo máximo de oxigênio; VE/VCO_2_: equivalente ventilatório de dióxido de carbono; O_2_: oxigênio; VE: ventilação/minuto; OUES: inclinação da eficiência da captação do oxigênio (do inglês *oxygen uptake efficiency slope* ); VO_2_: consumo de oxigênio; VO_2_pico: VO_2_ obtido nos exames nos quais não houver as características de um esforço máximo; LV1: limiar ventilatório 1; IC95%: intervalo de confiança de 95%; NYHA: New York Heart Association. *Após correção cirúrgica completa e com bons resultados, sem CIV residual e gradiente de pressão entre VD e artéria pulmonar <20 mmHg.

– Pacientes após correção cirúrgica com bons resultados (sem CIV residual e gradiente de pressão entre VD e a artéria pulmonar <20 mmHg geralmente são assintomáticos em repouso.
– Após a correção cirúrgica completa, geralmente não se observam grandes limitações físicas nas atividades do cotidiano. Entretanto, frequentemente o TCPE mostra valores reduzidos de VO_2_max e de VO_2_ no LV1.^713^
– Crianças e adolescentes que mantém resposta cronotrópica normal apresentam maior ACR e reserva da FC, mesmo quando com insuficiência pulmonar e disfunção sistólica do VD no repouso.^714^
– Adolescentes apresentam redução na ACR relacionada aos volumes sistólicos biventriculares e volume diastólico final do VE indexado à ASC. O valor de OUES e do PuO_2_pico também estão relacionados aos volumes sistólicos biventriculares.^715^
– Após a correção cirúrgica, a PAS em membro superior, a PAS central e o índice de rigidez arterial apresentam comportamento normal ao esforço.^591^
– Em pacientes assintomáticos após correção da ToF, evoluindo com estenose pulmonar grave e diminuição da ACR, deve-se considerar a troca valvar.^81^
– Pode ocorrer arritmia ventricular esforço-induzida (AVEI), geralmente relacionada ao reparo tardio, disfunção do VD e risco aumentado de eventos CV.

#### 1.5. Transposição das Grandes Artérias

A transposição das grandes artérias (TGA) é uma CC cianótica grave, incompatível com a vida, requerendo obrigatoriamente a existência de *shunt* intracardíaco (forame oval patente, comunicação interatrial ou CIV) e/ou *shunt* extracardíaco (PCA ou circulação colateral broncopulmonar).^723^

A TGA pode ser dividida em:^724 , 725^

– Simples, sem defeitos cardíacos adicionais ao *shunt* .
– Complexa, com lesão adicional associada: obstrução da via de saída do VE (≈25% dos pacientes); anomalias das válvulas mitral e tricúspide; anomalias de artérias coronárias; nos pacientes com CIV (≈50%), observam-se estenose ou atresia pulmonar, sobreposição de uma válvula atrioventricular ou CoAo.

A TGA requer tratamento cirúrgico logo após o nascimento ou no máximo nos primeiros meses de vida. Desde o final da década de 1980, tem-se preconizado a realização de cirurgia de troca (CT) arterial (cirurgia de Jatene) em vez da cirurgia de troca atrial (Mustard/Senning). Nos casos de TGA complexa, podem ser necessárias outras formas de abordagem cirúrgica (exemplos: Rastelli e Nikaidoh).^724 , 726 , 727^

Os pacientes necessitam de acompanhamento a longo prazo, pois é frequente a ocorrência de complicações: reintervenção em até 25% (devido a estenose da artéria pulmonar, obstrução de artéria coronária, dilatação da raiz da aorta e/ou IAo); disfunção do VD; bradiarritmias e taquiarritmias; DAC; morte súbita.^726 , 728 , 729^

O TE/TCPE apresenta papel relevante no acompanhamento após reparo:^22 , 723 , 724 , 726 , 729 , 730^

– Recomenda-se a realização a cada 3 a 5 anos, como parte da investigação de isquemia miocárdica assintomática, principalmente nos pacientes submetidos a CT arterial.
– Investigação de episódios de síncope e palpitações, geralmente decorrentes de arritmias secundárias à isquemia miocárdica, obstrução da via de saída de VD e/ou disfunção do VE. As arritmias ocorrem em 2,4 a 9,6% dos pacientes e associam-se a risco de MSC.
– Investigação de queixa de alterações na tolerância às atividades físicas habituais ou sintomas de dor torácica esforço-induzida, geralmente associadas ao declínio da função do VE, DAC e obstrução da artéria pulmonar.
– Para a estratificação de risco, prognóstico e liberação/prescrição de reabilitação cardiovascular.

O ECG de repouso varia com a técnica de reparo e sintomatologia do paciente. Na CT atrial é comum a observação de: DNS; ritmo juncional; distúrbios de condução atrioventricular; hipertrofia do VD e desvio do eixo para a direita; ondas Q nas derivações precordiais direitas. Na CT arterial, geralmente observa-se ritmo sinusal (91,1%) e, raramente, ritmo atrial ectópico (5,4%) ou ritmo juncional (3,6%). Também não se costumam evidenciar sinais de isquemia ou extrassistolia.^724 , 731^

Particularidades do TE/TCPE após reparo da TGA:

– Independentemente do procedimento adotado no reparo, os pacientes geralmente apresentam algum grau de comprometimento da ACR (%VO_2_pico previsto de 87,5±2,9%).^21^ Entretanto, mesmo com a aptidão levemente reduzida, os pacientes geralmente encontram-se em classe funcional I da NYHA.^723 , 728^
– Pacientes submetidos a CT arterial têm melhor tolerância ao esforço em comparação com aqueles submetidos a CT atrial.^723 , 732^
– Pacientes submetidos a CT arterial e reparo de CIV ou com obstrução residual de via de saída de VD apresentam maior comprometimento da ACR.^23 , 733^
– Na fase tardia da CT arterial, a FCmax geralmente é normal ou levemente diminuída (FCmax: 92±2% do previsto).^21 , 23^ A incompetência cronotrópica na fase tardia ocorre entre ≈5 e 34% dos pacientes. A DNS geralmente é secundária ao comprometimento da artéria do nó sinusal durante septostomia por balão ou mesmo CT arterial.^728 , 733^
– Na fase tardia da CT arterial, geralmente a PAS é normal no repouso e no esforço. A PAD geralmente apresenta valores menores no repouso e no pico do esforço.^731^
– Na fase tardia da CT arterial, geralmente observa-se redução do pulso de O_2_ com VO_2_pico normal (sem redução da ACR). A boa correlação entre o valor de OUES e VO_2_pico permite o seu uso nos pacientes que não atingiram o esforço máximo.^626 , 734^
– Na fase tardia da CT atrial (Mustard ou Senning): geralmente observam-se: arritmia ventricular (repouso e esforço); redução da fração de ejeção do VD (em até 84% dos pacientes); redução do pulso de O_2_ e do LV1; normalização lenta do pulso de O_2_ na recuperação; retenção prolongada de CO_2_ com subsequente hiperpneia.^729 , 735 , 736^
– Exames seriados na fase tardia da CT atrial evidenciam redução progressiva do VO_2_pico e pulso de O_2_ na infância e adolescência, sugerindo incapacidade de aumentar o volume sistólico.
– Na fase tardia da CT atrial, o VO_2_pico e pulso de O_2_ permanecem relativamente estáveis nos adultos jovens. Entretanto, quando ocorre acentuação da disfunção do VD observam-se declínio rápido no pulso de O_2_, piora da tolerância ao esforço, arritmias e deterioração clínica com IC.^737 , 738^
– A ocorrência de arritmias no pós-operatório precoce de CT atrial representa risco de arritmias na fase tardia (RR: 3,8; IC95%: 1,5-9,5) e de desenvolvimento de IC (RR: 8,1; IC95%: 2,2-30,7).^739^
– Na fase tardia da CT atrial, é comum observar FCpico reduzida e a ocorrência de incompetência cronotrópica.^735 , 740^
– Independentemente da técnica de reparo, o ISTE é raro, mas, caso preencha os critérios para isquemia miocárdica, deve-se prosseguir na investigação de DAC (geralmente assintomática; acometendo 2 a 11,3% dos pacientes).^741^

#### 1.6. Cirurgia de Fontan

A cirurgia de Fontan é um procedimento paliativo nas CC com um único ventrículo funcional, permitindo uma quase normalização da saturação arterial e a remoção da sobrecarga crônica de volume. A história natural dos pacientes com cirurgia de Fontan é caracterizada por aumento progressivo da resistência vascular periférica, subsequente redução do débito cardíaco, hipertensão venosa crônica, estase periférica e congestão no sistema linfático. As principais complicações são: cianose, intolerância aos esforços físicos, IC, ascite, arritmias, disfunção hepática, enteropatia perdedora de proteínas, bronquite e anormalidades da coagulação. A ocorrência de IC na cirurgia de Fontan é comum e progressiva, podendo ser sistólica, diastólica ou ambas. Fatores contribuintes para desenvolvimento de IC: disfunção ventricular diastólica, resistência vascular pulmonar aumentada, taquicardia atrial, insuficiência valvar e *shunts* com sobrecarga de volume.^742 – 744^

O TE/TCPE é útil no acompanhamento dos pacientes com cirurgia de Fontan, tendo em vista:^744^

– Quantificar a ACR e inferir sobre fatores limitantes ao esforço.
– Avaliar reserva respiratória, ventilação-perfusão, SpO_2_, resposta cronotrópica e arritmias, que contribuem para a limitação aos esforços e complicações tardias.
– Ajustes terapêuticos, inclusive o fechamento da fenestração (por hipóxia sistêmica excessiva) e indicação de implante de MP (por doença do nó sinusal/incompetência cronotrópica grave).^745 , 746^
– O TCPE contribui na seleção de candidatos ao transplante cardíaco.
– A estratificação de risco, prognóstico e liberação/prescrição de RCV.^747 – 750^
– Estratégia de vigilância intensiva de adolescentes com realização do exame a cada 1 a 3 anos, devido ao alto risco de IC e morte precoce segundo a *American Heart Association* .^744^

Particularidades do TE/TCPE na cirurgia de Fontan:

– Nos pacientes com IC e/ou SpO_2_ baixa em repouso, recomenda-se a realização do exame a nível hospitalar com adoção de cuidados especiais: adequação de protocolos/carga de esforço, monitorização SpO_2_ etc.
– A ACR é predominantemente reduzida com VO_2_max atingindo ≈60 a 65% do previsto.^92 , 747 , 751^
– A ACR nos pacientes com cirurgia de Fontan pode ser classificada de acordo com a %VO2 prevista atingida: capacidade severamente prejudicada se <50%; moderadamente prejudicada entre 50 e 60%; ligeiramente prejudicada entre 60 e 80%; limítrofe entre 80 e 90%; normal se >90%.^752^
– É comum a SpO_2_ em repouso ser baixa (inclusive com níveis <90%). A SpO_2_ no esforço geralmente cai para <90% por descompensação dos mecanismos de controle da cianose e aumento do retorno venoso de sangue dessaturado.^588^
– Crianças e adolescentes comumente apresentam, ao esforço, incompetência cronotrópica e reserva cronotrópica diminuída. O tipo de procedimento paliativo, o subtipo de ventrículo dominante e/ou a anatomia cardíaca subjacente afetam o grau de incompetência cronotrópica. Geralmente, o comportamento da FC na recuperação é normal.^753 , 754^
– A PAS repouso mantém-se inalterada, enquanto a PAD aumenta significativamente no pós-operatório. No esforço, os comportamentos da PAS e da PAD são normais, consistentes com a carga de esforço, atingindo geralmente >85% da PAS predita para faixa etária.^755^
– O aumento na duração e na dispersão da onda P no ECG de repouso associam-se ao risco de taquiarritmias atriais sustentadas (acometendo de 9,4 a 20% dos pacientes; incluindo FA e taquicardia reentrante intra-atrial).^756 , 757^
– A ocorrência de extrassistolia ventricular é rara, podendo ser decorrente de piora da função ventricular ou secundária a distúrbios eletrolíticos/medicamentosos. Cerca de 3 a 12% dos pacientes evoluem tardiamente com TV.^758 , 759^
– As arritmias esforço-induzidas são raras e geralmente desaparecem com a suspensão do esforço.^751^
– O ECG de repouso geralmente apresenta padrão de HVE, sobrecarga ventricular e ISTs significativo (>1,0 mm). Frequentemente, observa-se aumento do ISTs com o esforço, entretanto sem associação com DAC.^520 , 760^
– Geralmente, observa-se redução do pulso de O_2_, do LV1, da ventilação pulmonar, do QR e incompetência cronotrópica (em até 62% dos pacientes). Essas alterações associadas à função ventricular sistólica comprometida se correlacionam com pior ACR.^588 , 592 , 593 , 761^
– A diminuição de reserva cardíaca, VO_2_pico, OUES e incompetência cronotrópica identificam pacientes com maior risco de morte e necessidade de transplante cardíaco.^91 , 748 , 749 , 762 , 763^
– Em adolescentes, a ventilação oscilatória ao esforço (EOV do inglês, *exercise oscilatory ventilation* ) está associada ao aumento do risco de morte/transplante (RR: 3,9; IC95%: 1,5-10,0).^764^
– Em adolescentes, foram marcadores de risco de hospitalização em 2 anos (por IC, arritmia e outras complicações): OUES ≤45% (RR: 7,645; IC95%: 2,317-25,230); inclinação VE/VCO_2_ ≥37 (RR: 10,777; IC95%: 1,378-84,259).^765^

#### 1.7. Cardiomiopatia Hipertrófica

A CMH é uma doença genética com padrão autossômico dominante (penetrância incompleta e expressividade variável), apresentando miócitos hipertrofiados, desorganizados e separados por áreas de fibrose intersticial. A hipertrofia cardíaca é geralmente assimétrica, envolvendo mais comumente o septo interventricular basal subjacente à valva aórtica. Ocasionalmente, restringe-se a outras regiões cardíacas, como o ápice, porção média e parede posterior do VE. A CMH pode ser classificada em primária, se a mutação nos genes sarcoméricos representar a causa da doença, e em secundária, se associada a causa não sarcomérica.^766 , 767^

Na infância, a idade média de início é de 8,9 anos, sendo mais frequente no sexo masculino. O risco de MSC em pacientes pediátricos é de ≈1 a 7% ao ano. Em adolescentes com história familiar de MSC, o tempo médio após o diagnóstico para evento cardíaco maior (incluindo morte, MSC) ou intervenção cardíaca (miectomia e/ou CDI) é de ≈18 meses.^768 – 770^

Os sintomas geralmente resultam de quatro condições fisiopatológicas: disfunção ventricular diastólica, obstrução ao fluxo de saída do VE, isquemia miocárdica e arritmias cardíacas.^770^

Nesse contexto, o TE/TCPE é útil na estratificação de risco e manejo clínico, principalmente em crianças >7 anos, por serem de maior risco. Aproximadamente um terço dos pacientes com CMH apresenta obstrução da via de saída de ventrículo esquerdo (VSVE) em repouso intensificada com o esforço, um terço tem obstrução esforço-induzida e o outro terço tem HVE sem obstrução (em repouso ou esforço-induzida).^771^

Em pacientes com obstrução da VSVE, geralmente é audível um sopro rude mesossistólico, de grau 3-4/6, mais alto entre o ápice e a borda esternal esquerda. O sopro aumenta de intensidade quando o volume do VE diminui durante a manobra de Valsalva, ao assumir a posição ereta e durante e imediatamente após o esforço.^771^

Particularidades do ECG de repouso na CMH:^388 , 772^

– Alterado em 75 a 95% dos pacientes, mesmo quando sem ou apenas leve obstrução da VSVE.
– Presença de SAE.
– As anormalidades mais comuns são o padrão de HVE, ondas Q profundas, ISTs e alterações da onda T.
– De 2 a 5% dos pacientes exibem pré-excitação e podem apresentar arritmias supraventriculares nodais AV e síndrome de WPW.^768^

Particularidades do TE/TCPE na CMH:

– Auxilia nas decisões sobre o escalonamento das terapias, principalmente se os sintomas não forem claros baseados na história clínica.
– Geralmente evidencia baixa ACR.
– Pacientes com obstrução grave da VSVE geralmente apresentam pressão diastólica ventricular elevada e dispneia esforço-induzida. Nos casos mais graves, pode ocorrer franca IC aguda.
– A síncope esforço-induzida ou no início da recuperação decorre de obstrução grave da VSVE, com ou sem arritmia ventricular associada.
– Frequentemente ocorre dor torácica isquêmica, que pode ou não ter as características anginosas típicas.
– Resposta anormal da PA ao esforço, caracterizada por aumento da PAS <25 mmHg ou queda >10 mmHg, estão associadas ao risco aumentado de MSC.^154 , 438 , 773^
– O TE anormal associa-se a maior risco de morte por todas as causas e/ou transplante: resposta isquêmica (RR: 4,86; IC95%: 1,69-13,99) e resposta pressórica deprimida (RR: 3,19; IC95%: 1,32-7,71). A isquemia esforço-induzida também foi associada de forma independente com MSC (RR: 3,32; IC95%: 1,27-8,70).^157^
– ESVs e EVs esforço-induzidas são frequentes, podendo ocorrer TVNS em 20 a 30% dos pacientes.
– A ocorrência de arritmia esforço-induzida (atrial e/ou ventricular) em qualquer densidade está associada ao risco aumentado de transplante cardíaco, implante de CDI e MSC (RR: 5,8; IC95%: 1,3-26,7).^157 , 773^
– A FA é encontrada em ≈25% dos pacientes com CMH, sendo mal tolerada e frequentemente responsável por sintomas de IC ao esforço.
– A ACR comprometida (%VO_2_pico geralmente <80%) correlacionou-se com disfunção diastólica ao ecocardiograma.^207^
– O TCPE mensura diretamente a ACR, sendo relevante na avaliação de pacientes com sintomas graves, particularmente para a indicação de transplante cardíaco.^774^ A redução do VO_2_pico <50% dos valores previstos para idade e sexo deve ser considerada no processo de indicação do transplante.^775^
– O VO_2_pico, pulso de O_2_ e FCpico geralmente estão reduzidos com piora gradual ao longo do tempo.^207^ A %VO_2_pico prevista ≤60% é marcadora de risco de IC e MSC.^770^

#### 1.8. Doença de Kawasaki

A doença de Kawasaki (DK) é uma vasculite sistêmica aguda que afeta principalmente crianças <5 anos do sexo masculino (proporção ≈1,5:1). É a maior causa de DAC adquirida em crianças, sendo mais frequente no Japão.^37 , 776^

A complicação mais relevante da DK aguda é o desenvolvimento de anormalidades vasculares em artérias de pequeno a médio calibre (principalmente no coração), caracterizada por três processos interligados: arterite necrotizante; vasculite subaguda/crônica; proliferação miofibroblástica luminal. A DAC pode se desenvolver durante a fase de cicatrização do episódio agudo ou mesmo tardiamente. Mesmo crianças com DK sem evidência de lesões coronarianas apresentam menor reserva de fluxo coronariano, com maior resistência coronariana total.^777 , 778^

O risco de desenvolvimento de aneurismas em artérias coronárias (AAC) é de ≈25% dos casos não tratados e 5% dos casos adequadamente tratados. Os AAC podem se manifestar inicialmente como uma ectasia e progredir para dilatação moderada (5 a 8 mm de diâmetro) ou mesmo na forma de grandes aneurismas (>8 mm). Os AAC são classificados comparando os diâmetros dos das artérias coronárias indexadas em unidades de desvio padrão da média pela área de superfície corporal (escore Z). Essa classificação é recomendada para o tronco da coronária esquerda, descendente anterior (DA) e coronária direita (CD). A classificação considera o AAC como: ausente se o escore Z for <2; dilatação isolada se 2 a <2,5; aneurisma pequeno se ≥2,5 a <5,0; médio se ≥5,0 a <10,0 e dimensão absoluta <8 mm; grande ou gigante se for ≥10,0 (ou dimensão absoluta ≥8 mm). Os aneurismas grandes e/ou gigantes não regridem, raramente se rompem e quase sempre contêm trombos, que podem inclusive calcificar ou se tornar oclusivos.^37 , 776^

Crianças com AAC podem evoluir na fase tardia da DK (FT-DK) com trombose, doença isquêmica miocárdica, infarto e morte súbita (≈0,2 a 0,8% nos primeiros 10 anos após a DK). As complicações mais frequentes da FT-DK são a doença isquêmica miocárdica (4,6 eventos/1.000 pessoas-ano) e arritmias ventriculares (4,5/1.000 pessoas-ano). Pacientes na FT-DK necessitam de acompanhamento regular e adoção de protocolos de estratificação de risco e de prevenção de complicações, sendo o TE/TCPE útil nesse contexto.^779 – 781^

O ECG de repouso varia conforme as complicações decorrentes da fase aguda da DK. Em pacientes com AAC ou após infarto agudo do miocárdio (IAM) na fase aguda, é comum a observação de ondas Q patológicas e alterações do segmento ST/onda T associadas às áreas de isquemia e/ou de necrose. Recomenda-se avaliar a dispersão do QTi (QTd), que quando alterada está associada a sequelas coronarianas e maior risco de arritmia ventricular durante o seguimento.^487 , 782 , 783^

Principais indicações do TE/TCPE na DK:^18 , 37^

– Na FT-DK, para investigação de sintomas sugestivos de isquemia (GR-NE: I-C).
– Na população pediátrica, não devem ser indicados isoladamente para investigação de isquemia miocárdica esforço-induzida. Nesses casos, recomenda-se a associação com método de imagem.
– Em pacientes com AAC com suspeita de eventos isquêmicos, sintomas esforço-induzidos ou baixa tolerância aos esforços (GR-NE: I-C).
– Em pacientes com AAC, na avaliação pré-participação de esportes competitivos ou atividades de alta intensidade, buscando detectar arritmias esforço-induzidas (GR-NE: IIa-C).
– No acompanhamento de crianças e adolescentes submetidos a revascularização (cirúrgica e/ou percutânea), para avaliação da ACR, ajustes terapêuticos e progressão da DAC/reestenose.^784^
– Para estratificação de risco/prognóstico e liberação/prescrição de RCV (GR-NE: I-B).

Particularidades do TE/TCPE na DK:

– Em pacientes sintomáticos, auxilia no processo de indicação de revascularização. São considerados fatores de má evolução as arritmias esforço-induzidas e/ou baixa tolerância ao esforço (<3 METs) associada a sintomas (angina e dispneia).^18 , 37^
– Pacientes na FT-DK e com DAC moderada a grave podem apresentar doença do nó sinusal e distúrbios da condução atrioventricular.^785^
– Pacientes na FT-DK com escore Z ≥2,0 na DA proximal ou CD geralmente apresentam redução dos METs atingidos proporcionalmente ao grau do escore, menores níveis de ACR, QR, PAS máxima e duplo-produto (DP) máximo, quando comparados aos pacientes com escore Z <2,0.^15 , 786^ A redução da ACR geralmente é mais grave em adolescentes com DK.^16 , 17^
– Pacientes na FT-DK com AAC e sem defeitos de perfusão miocárdica apresentam respostas da FC, PAS e PAD ao esforço similares às de pacientes sem AAC. Entretanto, os pacientes com AAC e defeitos de perfusão miocárdica costumam apresentar menor FC no 1 ° minuto da recuperação e menor PAD (no 1 ° e no 5 ° minuto da recuperação), que são achados de pior prognóstico.^398^
– ISTE é comum na FT-DK, entretanto, com baixa sensibilidade e alta especificidade para lesões coronarianas obstrutivas.^18 , 787^
– Pacientes na FT-DK raramente apresentam arritmias ventriculares esforço-induzidas (relacionadas ao escore Z ≥5). Arritmias ventriculares complexas e TV esforço-induzidas estão associadas a AAC grandes, TV prévia, CDI, DAC, pós-IAM (geralmente após 10 anos) e pós-cirurgia de revascularização do miocárdio (CRVM).^560 , 788^
– A QTd ao esforço geralmente é alterada na FT-KD, independentemente da QTd em repouso ou de sequelas coronárias. Essa alteração representa risco de desenvolvimento de arritmias esforço-induzidas.^789^

### 2. Insuficiência Cardíaca/Transplante Cardíaco

No Brasil, em 2017, a prevalência de IC na faixa etária dos 5 a 14 anos foi de 34,1/100.000 crianças.^790^ Na população pediátrica com CC, a prevalência varia de 6,2% a 39%. A IC na população pediátrica apresenta alta morbidade e taxa de mortalidade intra-hospitalar variando entre 7 e 26%.^791 , 792^

As principais causas de IC na população pediátrica são apresentadas na Tabela 39 . A apresentação clínica da IC está relacionada à idade: lactentes e crianças pequenas apresentam dificuldade na alimentação, cianose, taquipneia, taquicardia sinusal e diaforese; crianças maiores e adolescentes apresentam fadiga, falta de ar, taquipneia e intolerância aos exercícios, dor abdominal, oligúria e edema de membros inferiores. A gravidade da IC deve ser classificada de acordo com a faixa etária através das classificações modificada de Ross (crianças <6 anos) e/ou NYHA (crianças >6 anos) – vide Tabela 39 .^379 , 793^

**Tabela 39:** Principais causas da insuficiência cardíaca na população pediátrica^794 , 795^

Tipo das causas	Exemplos
**Mutações genéticas**	Lamin A-C; proteína C de ligação à miosina; troponina I; tafazzin (síndrome de Barth); distrofina; LAMP2 (doença de Danon); distúrbios mitocondriais; titina; desmina.
**Miocardite**	Enterovírus; parvovírus; adenovírus; influenza; vírus Epstein-Barr; vírus da imunodeficiência humana; citomegalovírus; varicela; caxumba; doença de células gigantes; doença de Lyme; micoplasma; doença de Chagas.
**Isquemia**	Origem anômala da artéria coronária; doença de Kawasaki com aneurismas coronarianos.
**Distúrbios metabólicos**	Distúrbios da oxidação de ácidos graxos; distúrbios do armazenamento de glicogênio (por exemplo: Pompe); deficiência de carnitina.
**Doença cardíaca estrutural**	Doença valvular; cardiopatia congênita.
**Distúrbios endócrinos**	Hipotireoidismo; tireotoxicose; feocromocitoma; doenças de armazenamento de glicogênio.
**Distúrbios hematológicos**	Deficiência de ferro; anemia falciforme; hemocromatose; talassemia.
**Doenças autoimunes**	Lúpus eritematoso sistêmico; dermatomiosite; cardiomiopatia reumática.
**Agentes cardiotóxicos**	Antraciclina; ciclofosfamida; radiação.

A IC direita não é comum em crianças, mas pode estar associada a CC, incluindo ToF, TGA, DSA, anomalia de Ebstein, cardiomiopatia arritmogênica do VD e disfunção ventricular na fisiopatologia do ventrículo único. As duas principais causas de IC terminal na população pediátrica são as cardiomiopatias e CC, cada uma contribuindo com cerca de metade dos casos de transplante cardíaco (TCard). O TCard na população pediátrica representa 13% de todos os transplantes, e mais de 60% dos receptores sobrevivem por pelo menos 10 anos.^191 , 796 , 797^

Indicações do TE/TCPE na IC em população pediátrica:^182 , 797 – 800^

– A determinação da ACR e a avaliação do comportamento das variáveis do TCPE fornecem informações objetivas sobre o estado funcional do coração, pulmões e musculatura periférica, evolução da IC e auxiliam nas decisões terapêuticas.^6^
– O TCPE deve fazer parte da avaliação de pacientes (idade ≥6 a 8 anos) com cardiomiopatia e IC (GR-NE: IIa-C).
– O TCPE deve ser usado para determinar a causa da limitação cardiorrespiratória ao esforço em pacientes com sintomas de IC (GR-NE: IIa-C).
– Em pacientes com IC em estágio C, o VO_2_pico <50% do previsto associado a grave limitação ao esforço constitui a base para eventual indicação de TCard (GR-NE: IIa-C).
– Avaliação pré-participação e a estratificação de risco precedendo programa de treinamento físico/RCV (GR-NE: I-C).
– Na avaliação em pacientes com dispositivo de suporte e/ou após TCard, para determinação da ACR, estratificação de risco, avaliação seriada do enxerto e prescrição de programa de atividades (incluindo reabilitação e atividades físicas escolares) (GR-NE: IIa-C).
– Nos pacientes com suspeita de cardiotoxicidade (quimioterapia/radioterapia) no diagnóstico diferencial de dispneia, rastreamento de disfunção cardíaca (inclusive subclínica), estratificação de risco, ajustes terapêuticos e prescrição/liberação de exercícios físicos e reabilitação.^801^

O ECG de repouso na IC é inespecífico, mas frequentemente anormal, podendo apresentar hipertrofia de VE, sobrecarga VD e/ou VE, alterações do segmento ST e/ou da onda T. Distúrbios do ritmo são comuns, incluindo taquicardia sinusal, taquicardia supraventricular, FA/ *flutter* atrial, BAV e TV. Os distúrbios da condução intraventricular ou prolongamento do QTc geralmente estão associados à disfunção ventricular, IC e cardiopatia estrutural (CC ou cardiomiopatia avançada).^559 , 802^ Na cardiomiopatia idiopática, a presença de BRE e SAE correlacionam-se com risco aumentado de mortalidade.^379 , 803^

Particularidades do TE/TCPE na IC:

– Normalmente, são feitos em vigência de medicações em uso, inclusive antiarrítmicos e betabloqueadores. A suspensão pode desencadear piora do quadro clínico e maior risco de complicações durante o exame. Pacientes em uso de antiarrítmico são mais graves e apresentam maior risco de MSC em acompanhamento de 5 anos (RR: 3,0; IC95%: 1,1-8,3).^803^
– Crianças com IC secundária a cardiomiopatia dilatada idiopática apresentam no LV1 e no pico do esforço: valores significativamente menores de PAS, VC, VO_2_, VCO_2_ e VEmin; valores aumentados do VE/VO_2_ e VE/CO_2_; anormalidade do pulso O_2_; inclinação do VE/VCO_2_ no pico do esforço significativamente maior. As variáveis permitem a quantificação da redução da ACR e os eventuais mecanismos limitantes ao esforço.^804^
– O TCPE seriado em crianças com cardiomiopatia dilatada demonstrou risco de hospitalização por IC descompensada, suporte circulatório/TCard e morte nas que apresentaram redução de 10 mmHg na PASpico (RR: 1,41; IC95%: 1,12-1,79) ou redução de 10% na %VO_2_pico previsto (RR: 1,59; IC95%: 1,16-2,17).^805^
– VO_2_pico <44% do previsto em crianças com circulação biventricular associou-se a risco maior de morte ou deterioração da IC (RR: 5,1; IC95%: 1,9-13,5).^806^
– Embora rara, deve-se atentar para a possibilidade de desenvolvimento de IC descompensada aguda esforço-induzida, com necessidade de intervenção imediata/cuidados intensivos.^800^
– Na fase tardia de quimioterapia (10 anos) com antraciclina (dose cumulativa >300 mg/m^2^) ≈32% dos pacientes evoluíram com comprometimento da ACR (%VO_2_max previsto <80%) e disfunção cardíaca subclínica.^798^

Particularidades do TE/TCPE no TCard na população pediátrica:

– Na avaliação dos receptores de TCard, sugere-se a conversão das variáveis (VO_2_, FCpico, carga de trabalho etc.) em porcentagem prevista para a idade, sexo e/ou peso, de modo a permitir comparações seriadas e aos dados disponíveis na literatura.
– VO_2_pico ≤62% do previsto em pacientes com IC está fortemente associado ao risco de TCard e morte em 2 anos (RR: 10,78; IC95%: 4,04-27,98).^807^
– Crianças com circulação biventricular apresentam risco de morte, necessidade de suporte circulatório e TCard de urgência quando o VO_2_pico <50% do previsto (RR: 4,7; IC95%: 1,8-12,3) e a inclinação de VE/VCO_2_ ≥34 (RR: 3,2; IC95%: 1,2-8,4).^806^
– O TCPE faz parte da investigação detalhada necessária para a seleção de TCard, sendo o VO_2_pico <50% do previsto indicação Classe I.^808^ Outras indicações são VO_2_ <14 mL/kg/min (sem uso de betabloqueador) e VO_2_ <12 mL/kg/min (em uso de betabloqueador).^809^
– Após o TCard, geralmente observa-se comprometimento da ACR, tanto na fase imediata quanto 3 a 6 anos após o transplante, mas de maneira estável. Quanto menor for a idade do paciente no momento do transplante, maiores serão os valores de VO_2_pico. A carga máxima de trabalho diminuída (<75% do valor previsto) é frequentemente observada. O comportamento seriado da FC (repouso, pico e reserva cronotrópica), da PAS e VO_2_pico fornecem informações quanto à reinervação (geralmente com estabilidade ou aumento de valores) e evolução do enxerto, sendo a redução progressiva do VO_2_pico associada à perda do enxerto devido à vasculopatia.^810 , 811^
– A FC em receptores de TCard geralmente em repouso é maior e, no pico do esforço, é menor (variando entre 66 e 86% da FCmax prevista). A FC no 1 ° e 3 ° minuto da recuperação, está diminuída nos pacientes com denervação persistente.^191^
– Em média, 57% dos receptores apresentam evidências de reinervação autonômica (predominantemente simpática) associada a melhor ACR, maior sobrevida e estabilidade do enxerto. Pacientes com denervação autonômica geralmente evoluem com incompetência cronotrópica. O descondicionamento e efeitos secundários do tratamento de imunossupressão também podem afetar a ACR.^182 , 191 , 409 , 811^
– O TCPE realizado no pós-operatório imediato de TCard (1 ° mês) em uma série de pacientes demonstrou valores reduzidos do VO_2_ no LV1 e no pico do esforço (ambos com valores abaixo do previsto).^192^
– O TCPE seriado em pós-operatório tardio de TCard demonstrou que, no primeiro exame (≈3 anos), a %VO_2_ atingida foi de 59,3% e a %FCmax foi de 75,8%, permanecendo diminuída em exame subsequente (≈5 anos).^810^
– Comportamento de outras variáveis do TCPE nos receptores TCard: a VEmin pico geralmente é reduzida; a carga de trabalho é menor (variando de 60 a 66%); o VO_2_pico variou em 56±14% do previsto.^191^

### 3. Arritmias Cardíacas

#### 3.1. Síndrome do QT Longo Congênito

A síndrome do QT longo congênito (SQTL) é uma doença genética caracterizada por prolongamento do intervalo QTc (QTc >440 ms no sexo masculino e QTc >460 ms no feminino), com prevalência de 1:2.000 a 1:5.000. Pode causar síncopes, arritmias ventriculares e parada cardíaca. A idade média de apresentação da síndrome é de 14 anos, apresentando taxa anual de MSC entre 0,33% e 0,9%. A SQTL deve ser investigada em crianças e adolescentes com esse quadro clínico, antecedentes familiares de morte súbita e/ou diagnóstico de SQTL. Na SQTL tipo 1, o gatilho mais importante de arritmias é o exercício físico. Os critérios de Schwartz são recomendados para o diagnóstico de SQTL em população pediátrica e adulta.^812 – 814^

É recomendado que o QTi seja mensurado nas derivações D2 e V5.^82^ O prolongamento do QTi em ECG de repouso é a principal forma diagnóstica da síndrome. Entretanto, de 20 a 25% dos pacientes com SQTL confirmada têm intervalo QTc normal no repouso.^6 , 308^ A fórmula ideal de ajuste do QTc no TE permanece controversa (vide sessão "3.3.2.8. Intervalo QT" desta diretriz). A interpretação do QTc depende da fórmula utilizada.^124 , 542 – 544^ A Tabela 24 apresenta os valores de referência do QTc por faixa etária pediátrica.

A Diretriz de Arritmias Cardíacas em Crianças e Cardiopatias Congênitas indica o TE em:^82^

1. Pacientes com escore de Schwartz igual a 3,0 (probabilidade intermediária), quando o prolongamento do intervalo QTc na recuperação do exame agrega valor para o diagnóstico.^57^
2. Familiares assintomáticos com QTc de repouso <440 ms.
3. Pacientes sem fenótipo ou genótipo definido para adequação terapêutica.
4. Avaliação de sintomas inespecíficos ao esforço.

O TE pode revelar incompetência cronotrópica, alternância da onda T, taquiarritmias ventriculares ou comportamento paradoxal do QTi no esforço e/ou recuperação (aumentando em vez de diminuir).^57 , 813 , 815^

O QTc na fase de recuperação tem sido preconizado devido à dificuldade da medição do intervalo QT em FC elevadas. O QTc é medido em 3 a 4 minutos da fase de recuperação, sendo o aumento de ≥30 ms considerado significativo.^109 , 542 , 548 , 816^

Na avaliação de eficácia terapêutica (com betabloqueador) em pacientes com SQTL, o objetivo é determinar se há redução da resposta cronotrópica e/ou supressão de arritmias no esforço máximo.^817 – 819^

#### 3.2. Síndrome de Brugada

A síndrome de Brugada (SBr) é uma canalopatia hereditária autossômica dominante (mutações principalmente no gene SCN5A) causada por um defeito dos canais de sódio no epicárdio do VD. Apresenta ECG com padrão típico de elevação do segmento ST nas derivações precordiais direitas (V1-V3) com risco aumentado de morte súbita. A prevalência da SBr na população pediátrica é baixa (≈1 em 20.000), sendo a maioria assintomática. Alguns pacientes aparentemente saudáveis apresentam expressão precoce da doença, sendo a manifestação inicial a DNS e arritmias atriais. A SBr também pode manifestar-se com síncope, arritmias ventriculares potencialmente letais [TV polimórfica/fibrilação ventricular (FV)] e parada cardíaca (durante o sono e/ou desencadeada por hipertermia e/ou medicações).^820 , 821^

São fatores de risco para eventos arrítmicos recorrentes: história prévia de morte súbita abortada ou síncope; DNS; arritmias atriais; distúrbio na condução intraventricular; grande onda S em DI; presença de mutação SCN5A em adolescente.^822^ Alguns medicamentos e substâncias são potencialmente desencadeadores de eventos arrítmicos: antiarrítmicos, bloqueadores dos canais de sódio, antidepressivos tricíclicos, anestésicos locais, álcool, cocaína etc. (consulte lista completa no site www.brugadadrugs.org ).^823 , 824^

Suspeitar da SBr quando, no ECG de repouso, for observado nas derivações V1 e V2:^308 , 825^

– Tipo 1 – SSTs ≥2 mm, seguido de onda T descendente e negativa (semelhante a barbatana de tubarão). Esses achados são diagnósticos para a SBr tipo 1.
– Tipos 2 e 3 (também denominadas não-tipo I) – SSTs com onda T ascendente e positiva, respectivamente com 2 mm e <2 mm, sugerem a presença da canalopatia, porém exigem investigação adicional.

Outro critério diagnóstico da SBr é escore de Shanghai ≥3,5 desde que inclua um ou mais critérios eletrocardiográficos.^824 , 826^

Particularidades do TE/TCPE na SBr:

– A partir dos 6 ou 7 anos de idade, o exame está indicado para investigar incompetência cronotrópica, considerada uma manifestação de DNS. A incompetência cronotrópica ocorre em ≈7% dos pacientes. Cerca de 30% das crianças sintomáticas apresentam história de FA e DNS.^820 , 827^
– Geralmente, observa-se atenuação do SSTs no pico do esforço, seguida de seu reaparecimento durante a fase de recuperação.^126 , 525 , 526^
– Alguns pacientes (geralmente com mutação SCN5A) apresentam aumento na elevação do segmento ST (≥0,05 mV) no pico do esforço e, principalmente, na fase inicial de recuperação (associada ao aumento do tônus parassimpático). Esse aumento é considerado fator de risco para eventos cardíacos, especialmente para pacientes com história de síncope e nos assintomáticos.^126 , 526 , 828^
– Pode ocorrer aumento da densidade e complexidade de arritmias ventriculares com o esforço.
– No acompanhamento de crianças com SBr, os exames podem ser considerados para avaliar sintomas como síncope e palpitações.^824^

#### 3.3. Taquicardia Ventricular Polimórfica Catecolaminérgica

A taquicardia ventricular polimórfica catecolaminérgica (TVPC) é uma síndrome arrítmica hereditária (canalopatia) manifestada por TV bidirecional, TV polimórfica e/ou FV, desencadeada por estímulos adrenérgicos (esforço físico ou estresse emocional). A TVPC normalmente ocorre em corações estrutural e funcionalmente normais.^829^ A prevalência é de ≈1:5.000/10.000 pessoas, com ocorrência familiar de ≈30% dos casos. Ela pode ser autossômica dominante (mutações do gene RyR2) e, mais raramente, recessiva (principalmente mutações nos genes CASQ2, TRDN e CALM1-3). A idade média de expressão da doença é ≈10 anos, sendo os principais sintomas tontura, palpitações e pré-síncope, que podem progredir para síncope, hipotonia, convulsão e MSC. A morte súbita ocorre em 30 a 50% dos pacientes na faixa etária entre 20 e 30 anos. Até 30% dos pacientes com TVPC tem história familiar de síncope esforço-induzida, convulsão ou morte súbita.^830 – 832^

O TE/TCPE é a ferramenta diagnóstica mais relevante na suspeita de TVPC, tendo papel primordial na orientação da terapia dos casos confirmados, inclusive quanto à prática de exercícios físicos.

O exame físico pré-teste geralmente é normal. Suspeitar de pacientes com história prévia de episódios de síncope que foram caracterizados como eventos vasovagais ou de causa neurológica (principalmente epilepsia), considerando que, nesses casos, pode ter ocorrido atraso no estabelecimento do diagnóstico da TVPC.^829^

O ECG de repouso geralmente apresenta ritmo sinusal com FC normal ou bradicardia sinusal (≈20% dos pacientes), sem anormalidades da condução atrioventricular ou intraventricular e QTc normal. Alguns pacientes podem apresentar ondas U proeminentes e arritmias supraventriculares acompanhadas de DNS.^308 , 833 , 834^

Particularidades do TE/TCPE na TVPC:

– Na suspeita, realizar em ambiente hospitalar e com cuidados especiais (contraindicação relativa ao exame – vide Quadro 1 ) devido às possíveis complicações esforço-induzidas.^835^
– A ocorrência de sintomas esforço-induzidos típicos (tontura, palpitações, pré-síncope, síncope e MSC) geralmente está associada a arritmia ventricular complexa.
– Inicialmente, ocorrem EVs isoladas. À medida que o esforço continua, as EVs evoluem para bigeminismo ventricular seguido de complexos polimórficos. Se o esforço for interrompido nesta fase, é provável que os complexos ventriculares desapareçam gradualmente. Essa arritmia pode ser a única anormalidade observada em alguns pacientes com TVPC levemente afetados pela doença. Caracteristicamente, a FC durante a qual ocorrem as EVs situa-se entre 100 e 130 bpm, sendo tipicamente reprodutíveis.^829^
– Certas características das EVs podem, potencialmente, ajudar a distinguir a TVPC de arritmias ventriculares em controles saudáveis: densidade maior de EVs; primeiras EVs em carga de esforço intensa (≥10 METs); EVs com padrão de BRE e eixo inferior; bigeminismo ou trigeminismo no pico de esforço; duração dos complexos QRS >120 ms; intervalo de acoplamento >400 ms; desaparecimento das EVs no primeiro minuto da recuperação.^836 , 837^
– A complexidade e densidade da arritmia ventricular pode piorar com a progressão das cargas de esforço, sendo a FC associada a ocorrência da TV geralmente de ≈192 bpm. A ocorrência de TV bidirecional esforço-induzida, com rotação de 180° no eixo dos complexos QRS (batimento a batimento), é altamente característica de TVPC. O desenvolvimento de TV polimórfica seguida de FV ocorre em ≈7% dos exames.^838^
– Pacientes com TVPC e incompetência cronotrópica apresentam arritmia ventricular com maior densidade e complexidade, síncope e/ou parada cardíaca mais frequentemente quando comparados aos com resposta cronotrópica normal.^408^
– Alguns pacientes com TVPC podem apresentar taquiarritmias supraventriculares esforço-induzidas (incluindo FA), as quais não são diagnósticas da síndrome.^839^
– Exame com TV bidirecional ou polimórfica é altamente preditivo de TVPC (especificidade de 97%), tendo associação significativa com mutação genética. No entanto, a sensibilidade costuma ser de ≈50%, não permitindo descartar o diagnóstico de TVPC somente com um único exame normal, principalmente em crianças na primeira infância.^112 , 840^
– Em pacientes com suspeita de TVPC e TE/TCPE anterior normal, é possível a utilização de protocolos modificados de " *sprint* " (alta carga de esforço desde o início do exame em cicloergômetro e duração de 3 a 6 minutos) ou de " *burst* " (esforço de alta intensidade, desde o início do exame, equivalente ao estágio máximo alcançado no TE anterior) na tentativa de desmascarar a síndrome. Apenas 28% dos portadores da variante patogênica RyR2 apresentam TE anormal em protocolo padrão. Entretanto, no protocolo modificado verificam-se 83% de exames anormais.^113 , 839 , 841^
– A realização do exame é fundamental na triagem familiar de parentes de primeiro grau (e se possível de segundo grau), devido à gravidade das manifestações clínicas, prognóstico desfavorável e possibilidade de identificação precoce de portadores assintomáticos que se beneficiariam de terapêutica específica. O rastreamento geralmente é feito em protocolo atenuado. É importante observar que alguns pacientes com TVPC podem ter um exame normal na primeira infância, que pode tornar-se positivo posteriormente. Portanto, é indicado o acompanhamento regular e exames seriados.^82 , 842 – 844^
– O acompanhamento seriado com TE/TCPE é obrigatório para avaliar a efetividade da terapêutica instituída no controle da arritmia ventricular e na manutenção da FC em níveis inferiores ao limiar desencadeante. Os exames são feitos em vigência de medicação (inclusive betabloqueador). Em pacientes que mantenham arritmias ventriculares esforço-induzidas na forma pareada, TV não sustentada, TV polimórfica ou bidirecional, deve-se avaliar a terapia adicional (com flecainamida). Caso persista a AVEI e/ou os sintomas, deve-se considerar CDI, com ou sem denervação simpática cardíaca esquerda.^842 , 845 – 847^
– O exame também deve ser feito na avaliação pré-participação de exercícios físicos como lazer. Assintomáticos por um período mínimo de 3 meses (incluindo pacientes com CDI), com exame sem qualquer ectopia ventricular ou arritmia e mantendo o tratamento medicamentoso adequado, poderão ser liberados para exercícios físicos como lazer (de intensidade baixa a moderada). Durante os exercícios físicos, os pacientes deverão permanecer abaixo da FC correspondente ao limiar desencadeante das arritmias. Considerar também a necessidade de evitar desidratação, distúrbios eletrolíticos e hipertermia.^829 , 848^

#### 3.4. Cardiomiopatia Arritmogênica Ventricular/Displasia Arritmogênica do Ventrículo Direito

A cardiomiopatia arritmogênica ventricular (CAV) é uma cardiomiopatia hereditária caracterizada pela substituição fibroadiposa dos miócitos ventriculares, resultando em anormalidades elétricas, disfunção cardíaca, IC, arritmias ventriculares e/ou morte súbita. Embora se manifeste predominantemente no VD (displasia arritmogênica do VD – CAVD), é uma doença pancardíaca. Nos adolescentes que se tornaram sintomáticos, o envolvimento biventricular é o mais comum. A prevalência na população geral é de ≈1:5.000, afetando mais o sexo masculino (proporção ≈3:1). Representa uma das causas mais comuns de morte súbita juvenil, principalmente entre atletas.^849 – 851^

Na população pediátrica, a apresentação da CAV varia com a idade, sexo e herança genética, sendo as principais manifestações: FV/MSC, geralmente a primeira manifestação da doença em adolescentes; queixa de palpitações e síncope; IC como primeira manifestação clínica nos pré-púberes (≈37% com envolvimento biventricular) ou nos estágios avançados da doença (alta prevalência).^852 , 853^

A CAVD em crianças ≤12 anos apresenta evolução desfavorável com alta incidência de eventos cardíacos, incluindo transplante cardíaco e arritmias ventriculares graves. Em jovens, exercícios físicos extenuantes (estimulação adrenérgica) podem atuar como modificador fenotípico da CAV, tornando-se gatilho para arritmias malignas e MSC.^854 , 855^

Nos casos suspeitos, recomenda-se a utilização dos critérios diagnósticos da CAV revisados da Força-Tarefa Internacional de 2010 (FIT-2010) e também os "critérios de Padua" (CPa). Nas crianças, os critérios eletrocardiográficos do FIT-2010 apresentam menor aplicabilidade, subestimando a ocorrência de CAV. Os CPa melhoram a acurácia nas crianças pelo uso da ressonância magnética cardíaca (RMC), estratificando a doença pelas variantes fenotípicas (dominante direita, dominante esquerda e variante biventricular).^856 – 858^

Particularidades do TE/TCPE na CAV:

– Corrado et al. propuseram a atualização dos CPa, incluindo o TE como parte da avaliação clínica não invasiva, visando a registrar a densidade e morfologia das arritmias ventriculares. Portanto, caso ocorra arritmia ventricular durante o exame, recomenda-se registrar sua densidade, morfologia dos complexos QRS ectópicos e o comportamento em cada fase (repouso, esforço e recuperação).^856^
– Arritmias ventriculares esforço-induzidas são relativamente comuns, sendo consideradas típicas da CAVD a TV monomórfica com padrão de BRE. Entretanto, a ausência ou supressão de arritmias ventriculares ao esforço não exclui o diagnóstico da CAVD.^258 , 859^
– Outras indicações: na investigação inicial; auxilio nas decisões terapêuticas; em adolescentes na avaliação pré-participação esportiva; diferenciação entre alterações miocárdicas na CAV daquelas relacionadas ao remodelamento fisiológico dos atletas; prescrição/restrição de exercícios nos pacientes com diagnóstico firmado; otimização da vigilância médica de portadores assintomáticos com genes da doença.^258 , 516 , 860 , 861^
– Deve fazer parte da avaliação periódica (a cada 6 meses) em adolescentes e adultos jovens com diagnóstico firmado, que realizam exercícios/esportes recreativos de baixa/moderada intensidade, para avaliação da capacidade funcional e estratificação de risco. O exame não deve ser realizado durante os períodos mais sintomáticos da doença ("fases quentes"). A presença de sintomas ou arritmias esforço-induzidas devem resultar em recomendações mais conservadoras e maiores restrições das atividades físicas.^62 , 862^
– Na maioria dos pacientes, são encontradas anormalidades no ECG de repouso que, em muitos casos, precedem as anormalidades estruturais. Pacientes sintomáticos geralmente apresentam ECG mais alterado do que os assintomáticos. Principais alterações em pacientes >14 anos: presença de ondas T invertidas nas derivações precordiais direitas (de V1-V3 ou além), na ausência de BRD; ondas épsilon (entre 7 e 30% dos pacientes); arritmias ventriculares.^863 , 864^
– A intolerância aos esforços é uma das manifestações dos pacientes com IC, sendo o exame indicado para avaliar a ACR e quantificar o grau de comprometimento.^865^
– Observou-se no TE em adolescentes e adultos jovens com CAVD: sintomas esforço-induzidos (dor torácica limitante, dispneia grave, pré-síncope e palpitações) em 11,4%; pseudonormalização das ondas T em 40,0%; ISTE em 8,6%; aumento da densidade da ectopia ventricular em 31,4% e TV não sustentada em 11,4%.^516^
– Pacientes com sintomas (palpitações e síncope) e/ou TV esforço-induzidas devem avançar na investigação diagnóstica da CAV em caráter de urgência.^866^
– Pacientes assintomáticos portadores de mutações no gene PKP2 e ECG de repouso normal podem apresentar ondas épsilon esforço-induzidas.^861 , 867^
– Anormalidades esforço-induzidas da despolarização ventricular são comuns em portadores assintomáticos de genes: ondas épsilon (em 14%); aumento na duração da ativação terminal dos complexos QRS (≥55 ms; em 32%); arritmias ventriculares esforço-induzidas com eixo superior do QRS (em 57%).^861^
– O TCPE é útil em crianças e adolescentes que evoluíram com IC (geralmente por envolvimento biventricular) para estratificação prognóstica, ajustes terapêuticos e seleção de pacientes para terapias avançadas da IC (transplante cardíaco ou dispositivos de assistência ventricular).^852 , 868^

#### 3.5. Bloqueio Atrioventricular Total (Congênito e na Infância)

O bloqueio atrioventricular total (BAVT) é definido como congênito se diagnosticado no útero, no nascimento ou no primeiro mês de vida; como BAVT na infância se diagnosticado entre o primeiro mês e 18 ° ano de vida. O BAVT adquirido decorre de situação aguda, reversível ou não. A prevalência do BAVT congênito é de 1 para 15.000 a 20.000 nascidos vivos (60% sexo feminino), com malformação cardíaca em ≈25 a 50% dos casos.^107 , 869^

Mais da metade dos BAVTs congênitos são causados por autoanticorpos que, em fetos suscetíveis, danificam os cardiomiócitos e o tecido de condução do nó AV. As gestantes podem ser assintomáticas sendo que, ≈⅓ tinham diagnóstico prévio de doença reumática (principalmente LES e artrite reumatóide). A prevalência é de 2 a 5% das gestações com anticorpos anti-Ro/SSA positivos (o mais comum) e/ou anti-La/SSB. A taxa de recorrência em gravidez subsequente é de 12 a 25%. O BAVT congênito está associado a mortalidade de ≈16 a 30% (predominante intraútero e nos primeiros meses de vida) e ao desenvolvimento de cardiomiopatia dilatada (em 5 a 30% dos casos).^107 , 870 , 871^

O BAVT na infância geralmente decorre de BAVT congênito não diagnosticado anteriormente, BAVT adquirido, por doença de condução cardíaca progressiva hereditária (associada a mutações nos genes SCN5A, SCN1B, SCN10A, TRPM4 e KCNK17) ou idiopático. Na maioria dos casos, não está associado com doença cardíaca estrutural ou doença autoimune.^583^

Pacientes com BAVT congênito isolado (sem malformação cardíaca associada) requerem acompanhamento clínico criterioso. Inicialmente, são assintomáticos, podendo desenvolver cardiomiopatia dilatada por disfunção ventricular secundária a bradicardia, sendo essa a principal causa de morbidade e mortalidade. A bradicardia significativa e/ou episódios de Stokes-Adams são as principais indicações para implante de MP.^872^

O BAVT adquirido geralmente é decorrente de: trauma cirúrgico (em 3 a 8% dos pacientes com CC) ou durante procedimento transcateter; processos infecciosos agudos ou crônicos; miocardite; cardite reumática aguda; febre reumática aguda; doença de Chagas; anormalidades metabólicas (hipotireoidismo); processos infiltrativos; mecanismo neurocardiogênico patológico. Embora o BAVT adquirido seja raro e potencialmente transitório, o TE/TCPE é útil para a estratificação de risco e no processo de indicação de MP.^107^

Nos pacientes com BAVT, o TE está indicado para investigar a sintomatologia, avaliar o aumento da resposta do escape ventricular, determinar eventual ocorrência de ectopias e documentar a repercussão hemodinâmica.^114 , 554^

Particularidades do TE no BAVT:

– No ECG de repouso, observa-se, no BAVT de origem supra-hissiana, complexos QRS do escape ventricular com duração normal (e nos casos adquiridos, semelhante ao do ECG anterior ao BAVT); nos de origem infra-hissiana, os complexos QRS são largos. O prolongamento do QTi em pacientes com BAVT congênito geralmente é uma manifestação fenotípica de SQTL congênito latente, sendo fator de risco para síncope e/ou morte súbita.^115 , 873^
– A evolução natural do BAVT congênito consiste no declínio progressivo das frequências ventriculares ao longo da vida. No ECG de repouso, entre 6 e 10 anos, observa-se FC média de 50 bpm e, entre 16 e 20 anos, de 45 bpm.^556^
– A ACR fornece informações relevantes sobre o estado de saúde e a capacidade de realizar atividades físicas adequadas à idade. A ACR comprometida, com ou sem sintomas esforço-induzidos, é um dos critérios para indicação de implante de MP.
– Principais sintomas esforço-induzidos: intolerância ao esforço, dispneia, pré-síncope, síncope, Stokes-Adams (principalmente se o QTi for prolongado).^556 , 874^
– Não são recomendadas a utilização de equações de predição de VO_2_max e da FCmax.
– O aumento da atividade simpática sem o correspondente aumento efetivo da FC pelo ritmo de escape pode resultar em arritmias ventriculares complexas e complicações graves, principalmente se associado a CC ou IC. Reserva cronotrópica <50 bpm associada ou não a capacidade funcional reduzida (<7 METs) associa-se com má evolução e necessidade de implante de MP. A AVEI é frequente (50 a 70% dos pacientes) com sua densidade e complexidade relacionadas à duração dos complexos QRS e ao aumento da idade (independente da resposta da FC ao esforço). O BAVT localizado no sistema His-Purkinje está associado a ocorrência de ectopia ventricular esforço-induzida, com maior risco de morte súbita.^115 , 555 , 557^
– Fadiga, dispneia, tontura e ectopias ventriculares esforço-induzidas foram responsáveis por ≈26,5% dos implantes de MP. Nos pacientes assintomáticos, outras indicações foram bradicardia pronunciada persistente (inclusive ao esforço) e/ou QTc prolongado.^107 , 115^

Particularidades do TE/TCPE após implante de MP no BAVT:

– O TE permite investigar sintomas esforço-induzidos, avaliar a ACR, avaliar o comportamento da frequência atrial do paciente, verificar a efetividade da programação da resposta de frequência de estimulação, avaliar possíveis falhas esforço-induzidas do MP e contribuir para eventual atualização do MP dupla-câmara/transvenoso.^872 , 875 , 876^
– Após o implante do MP, ≈20% das crianças permanecem sintomáticas e/ou com comprometimento da ACR. Essa situação ocorre principalmente nos MP em modo de estimulação VVIR (epicárdico) no ápice do VD.^877^
– Geralmente, a definição do local de estimulação (epicárdico ou transvenoso) dependerá do peso do paciente. A abordagem epicárdica será necessária se o peso for <10 a 15 kg, enquanto a utilização da via transvenosa será possível nos pacientes com >20 kg. Em pacientes com peso entre 15 e 20 kg pode-se utilizar qualquer um dos dois locais de implante. A estimulação transvenosa em dupla-câmara apresenta melhores resultados quanto à ACR.^872 , 878^
– Crianças com MP com eletrodo único posicionado no ápice do VD podem evoluir com dessincronização da ativação e contração do VE, resultando em diminuição da função do VE, redução da ACR e incompetência cronotrópica. A estimulação apical crônica do VD pode evoluir para IC em ≈7% das crianças.^879^
– Pacientes com estimulação apical do VE apresentam maiores VO_2_pico, tempo de esforço, FCpico, índice cronotrópico e menos sintomas esforço-induzidos do que os pacientes com estimulação apical do VD.^877^

### 4. Isquemia Miocárdica

A isquemia miocárdica na população pediátrica geralmente é parte de um conjunto de condições e doenças (congênitas ou adquiridas), que podem provocar obstrução da circulação coronariana (dinâmica ou fixa) e/ou disfunção da microcirculação (vide Tabela 40 ). Embora a isquemia não seja frequente, é um evento grave e com risco de morte, necessitando investigação diagnóstica adequada, monitoramento de sua evolução e das doenças associadas.^19 , 20 , 880^

**Tabela 40:** Principais condições para isquemia miocárdica na população pediátrica^19 , 20 , 880 – 882^

Mecanismos	Patologias
**Aterosclerose**	DAC em sobreviventes de CC até idades mais avançadas.
	DAC em CC por aumento de fatores de risco coronariano, por exemplo, coarctação de aorta reparada com hipertensão persistente.
	DAC precoce por hipercolesterolemia familiar, doença renal crônica avançada/insuficiência renal terminal e lúpus eritematoso sistêmico.
**Cirurgia de reimplante coronário**	Transposição de grandes vasos com cirurgia de troca arterial.
	Coronária esquerda anômala da artéria pulmonar (ALCAPA) com reimplante coronariano.
	Doença da valva aórtica em pacientes submetidos à cirurgia de Ross.
	Aneurismas da aorta ascendente em pacientes que necessitam de substituição da raiz da aorta proximal.
**Compressão da artéria coronária**	Origem anômala da artéria coronária direita ou esquerda no seio oposto, com trajeto interarterial/intramural.
	Colocação de *stent* na artéria pulmonar proximal ou implante de válvula pulmonar percutânea comprimindo uma artéria coronária.
	Válvula percutânea (TAVI) na posição aórtica, obstruindo o óstio coronário.
	Ponte intramiocárdica.
**VD sistêmico**	TGA corrigida.
	TGA com reparo de switch atrial (Mustard ou Senning).
**Fístulas coronarianas**	Fístula coronária.
	Atresia pulmonar com septo ventricular íntegro, VD hipoplásico e fístula coronariana para VD.
**Síndrome de Williams**	Aortopatia supra-aórtica com estreitamento da artéria coronária.

CC: cardiopatia congênita; VD: ventrículo direito: TGA: transposição das grandes artérias; DAC: doença arterial coronariana; TAVI: implante percutâneo de válvula.

A DAC aterosclerótica na população pediátrica geralmente está associada a situações que causam aterosclerose prematura:

1. Hipercolesterolemia familiar, distúrbio genético autossômico dominante do metabolismo do colesterol. Afeta 1:250 indivíduos em sua forma heterozigótica, causando aterosclerose prematura em adolescentes e adultos jovens.^59 , 883 , 884^
2. Doença renal crônica (DRC) avançada principalmente na insuficiência renal terminal/dialítica. A calcificação coronariana é frequente, sendo associada a uremia, metabolismo anormal, aumento do fator de crescimento de fibroblastos 23 (FGF23) e deficiência do fator Klotho. Crianças com DRC apresentam alta prevalência de fatores de risco para DCV aterosclerótica, igualmente ao observado em adultos. A *American Heart Association* estratifica os pacientes pediátricos com DRC na categoria de alto risco para o desenvolvimento de DCV precoce e DAC aterosclerótica manifesta antes dos 30 anos de idade.^885 , 886^
3. Lúpus eritematoso sistêmico (LES) é uma doença autoimune com padrão de inflamação sistêmica (em crises), sendo o dano tecidual causado por autoanticorpos, criação de imunocomplexos e/ou deposição de autoanticorpos. O LES está associado a aterosclerose acelerada, DAC, doença arterial periférica (DAP), valvopatia, miocardite, disfunção do VE (nas crianças com LES ativo) e risco aumentado de eventos CV. A aterosclerose precoce decorre da hiperleptinemia e de anormalidades da regulação imunológica, da função das células endoteliais e do reparo vascular. A DAC pode ocorrer em qualquer estágio do LES, sendo os indivíduos mais jovens os de maior risco.^887 – 890^

Marcadores de alto risco para isquemia miocárdica em crianças/adolescentes com queixa de dor torácica: exame físico CV anormal (exemplos: sopro cardíaco, cianose, alterações de pulso periférico etc.); dor torácica ou síncope aos esforços; dor precordial associada a palpitações; anormalidades eletrocardiográficas; história familiar de arritmias, morte súbita ou distúrbios genéticos; histórico de cirurgia ou intervenções cardíacas; TCard; história de DK; história de hipercolesterolemia familiar; diagnóstico de DRC e LES.^20^

O ECG de repouso visa evidenciar arritmias, alterações da condução e do segmento ST/onda T (podem sugerir pericardite, miocardite ou alteração coronariana) e sinais de HVE. A presença de BRE, WPW e MP interferirão na análise das alterações da repolarização quanto à isquemia durante o TE.^7^

Particularidades do TE/TCPE na isquemia miocárdica:

– Indicado na investigação de dor torácica em crianças e adolescentes com alto risco de eventos CV isquêmicos ( Tabela 40 ).
– Os parâmetros como VO_2_pico, anormalidades no pulso de O_2_, inclinação VE/VCO_2_ e relação VO_2_/carga de trabalho (ΔVO_2_/ΔWR) ajudam no diagnóstico do comprometimento miocárdico, decisões terapêuticas e liberação/prescrição de atividades físicas.^179^
– As anomalias congênitas das artérias coronárias são causas conhecidas e frequentes de isquemia esforço-induzida: origem anômala da aorta ou da artéria pulmonar, orifício anormal, curso arterial intra ou intermural (entre a aorta e a artéria pulmonar).^891^
– Após correção cirúrgica dessas anomalias, é indicado para estratificação de risco e ajustes terapêuticos: aumento do orifício coronariano, reimplante com e sem extensão das artérias coronárias, translocação da artéria pulmonar e revascularização miocárdica.^891^
– Após procedimentos de troca arterial e procedimento de Ross, para estratificação de risco de isquemia pós-operatória precoce e disfunção miocárdica. A isquemia coronariana tardia pode exigir reoperação.^891 , 892^
– Em pacientes com síndrome do coração esquerdo hipoplásico e cirurgia de Fontan, a incidência de ISTE foi de 48%, sem registro de mortes em acompanhamento por ≈2 anos. Os pacientes investigados adicionalmente não apresentaram defeitos de perfusão reversíveis ou DAC obstrutiva.^520^
– Pacientes avaliados quanto a lesão residual da artéria coronária após cirurgia corretiva [devido a TGA, origem anômala ou origem anômala da artéria coronária esquerda da artéria pulmonar ( *anomalous origin of the left coronary artery from the pulmonary artery* – ALCAPA)], o ISTE apresentou sensibilidade de 100% e especificidade de 81% para lesão grave residual (>50%). Os marcadores de risco para lesão grave foram dor torácica esforço-induzida (RR: 4,72; IC95%: 1,23-18,17) e via intramural inicial da coronária (RR: 4,37; IC95%: 1,14-16,81).^893^
– Crianças com ponte miocárdica e CMH apresentaram menor tempo de esforço, menor PASpico (redução média 17±27 mmHg), maior dispersão do QTc (104±46 ms) e ISTE (mediana 5 mm). No seguimento de 7,1±5,4 anos, observou-se dor torácica em 60% dos pacientes, TV em 80% e parada cardíaca com ressuscitação em 50%.^894^

### 5. Lesões Valvares

#### 5.1. Estenose Aórtica Congênita

A estenose valvar aórtica (EAo) congênita é um defeito cardíaco que causa obstrução hemodinamicamente fixa e significativa da via de saída do VE. Corresponde a ≈3 a 6% das CC, sendo mais frequente no sexo masculino (proporção entre 3:1 e 5:1). Cerca de 15 a 20% dos pacientes com EAo apresentam outras CC associadas, sendo as principais a PCA, CoAo e DSV.^895^

A valva aórtica unicúspide é frequentemente observada na EAo crítica, possuindo um orifício excêntrico com comissura patente ou orifício central sem comissura. As valvas aórticas bicúspides geralmente estão associadas à dilatação da aorta ascendente, ocorrendo aumento de tamanho e alterações degenerativas com o envelhecimento.^370 , 896^

A EAo no início da infância geralmente é grave (crítica) e está associada à insuficiência do VE, sinais de baixo débito cardíaco, IC, cardiomegalia, edema pulmonar, palidez cutânea ou pele acinzentada, hipotensão e dispneia. A maioria das crianças e adolescentes com EAo discreta permanece assintomática, apresentando crescimento e desenvolvimento normais. Sintomas de dispneia, angina ou síncope, particularmente aos esforços, ocorrem em ≈10% da população pediátrica entre 5 e 15 anos. O início de sintomas requer avaliação imediata por causa do risco de morte súbita (≈1 a 10% nos pacientes com EAo moderada/grave). Cerca de 2 a 4% de todos os atletas jovens com MSC apresentavam EAo.^181^

A EAo congênita está associada ao desenvolvimento de HVE e ao aumento do risco de DCV. A EAo supravalvar, mais comumente associada à síndrome de Williams, pode conferir risco CV aumentado devido à sua associação com estenoses coronarianas, isquemia miocárdica, síncope esforço-induzidas e estenose das artérias renais, que pode causar hipertensão secundária.^81^

No ECG de repouso, as alterações eletrocardiográficas não são diagnósticas de EAo nem sensíveis para determinação do seu grau de gravidade. Entretanto, a observação de HVE e ISTs ≥2 mm é indicadora relativamente sensível de EAo grave. Na EAo moderada/grave, é comum a observação de arritmias ventriculares. A dispersão do QT é prolongada em crianças (particularmente nas com arritmia), sendo o grau de prolongamento relacionado com o gradiente de pressão e o índice de massa do VE.^388 , 897 , 898^

Particularidades do TE/TCPE na EAo congênita:

– Na EAo moderada/grave sintomática, está contraindicado.
– É indicado na EAo que apresenta gradiente médio em repouso <30 mmHg ou gradiente de pico <50 mmHg.
– EAo moderada para avaliação pré-participação de atividades esportivas devendo: atingir nível de esforço compatível com a atividade desejada, demonstrar ACR satisfatória, resposta normal da PAS e ausências de sintomas, de ISTs e de taquiarritmias ventriculares.^181^
– Pacientes assintomáticos com EAo moderada/grave geralmente apresentam comprometimento da ACR, principalmente se o gradiente sistólico do VE ≥30 mmHg. O grau de comprometimento relaciona-se com a área valvar aórtica em repouso.^436 , 899^
– A maioria dos pacientes assintomáticos com EAo moderada apresenta aumento moderado na PAS (<25 mmHg).
– A variação da PAS entre esforço e basal (ΔPAS) depende do grau da estenose, sendo menor na EA grave (ΔPAS = 21,6 mmHg) do que na moderada (ΔPAS = 32 mmHg).^900^
– Na EAo moderada/grave, pode ocorrer ISTE, queda ou aumento inadequado da PAS e arritmias esforço-induzidas.^437^
– A gravidade da EAo está associada ao ISTE com *odds ratio* (OR) 12,0 (IC95%: 3,0-49,0). O ISTE está relacionado à pressão sistólica do VE, ao gradiente de fluxo de saída do VE (principalmente se ≥70 mmHg) e relação comprometida da oferta-demanda de O_2_.^436 , 518 , 899^
– Na EAo supravalvar, geralmente ocorrem arritmias ventriculares complexas e acentuação de ISTs com o esforço (indicativa de isquemia miocárdica).^434^
– Após tratamento cirúrgico, observam-se redução do ISTE, aumentos do ΔPAS e da ACR.^519^

#### 5.2. Insuficiência Aórtica

Na insuficiência aórtica (IAo), ocorre aumento do volume diastólico final do VE, elevação do estresse na parede e hipertrofia miocárdica compensatória. Raramente ocorre como lesão isolada, estando frequentemente associada à EAo (inclusive após intervenção cirúrgica ou transcateter) ou DSV. A válvula aórtica bicúspide é a causa mais comum de IAo.^896^

A IAo crônica geralmente é bem tolerada, sendo que a maioria das crianças permanecem assintomáticas. Entretanto, na IAo moderada/grave, é comum o desenvolvimento de sintomas significativos e/ou disfunção do VE, exigindo intervenção cirúrgica. A IAo grave resulta em volumes sistólicos e diastólicos finais do VE muito aumentados, geralmente evoluindo com disfunção progressiva. Na IAo grave, as pressões diastólicas reduzidas na raiz da aorta podem prejudicar o fluxo coronário.^901 – 905^

O ECG de repouso na IAo moderada/grave geralmente apresenta padrão de HVE e, na fase crônica, alterações do segmento ST e onda T.^388^

Particularidades do TE/TCPE na IAo:^903 , 904 , 906^

– Indicado para avaliação de sintomas, ACR, isquemia esforço-induzida, ajustes terapêuticos e liberação/prescrição de exercícios físicos.
– Pacientes que desenvolvem sinais ou sintomas de IC, isquemia esforço-induzida e/ou declínio da função do VE geralmente necessitam de intervenção cirúrgica.
– Pacientes com IAo moderada ou grave apresentam comprometimento das respostas cronotrópica e pressórica (inclusive com queda pressórica intraesforço). Verifica-se também maior incidência de ectopias e ISTE.
– Em atletas, é indicado para confirmar eventuais sintomas, avaliar a tolerância ao esforço e resposta da PA, sendo esses parâmetros relevantes para eventual liberação para prática esportiva. O TE deve pelo menos atingir nível de atividade compatível com a prática esportiva pretendida.
– Atletas assintomáticos, com IAo discreta a moderada, VE sem disfunção e TE normal podem participar de todos os esportes competitivos (GR-NE: I-C).
– A participação em atividades esportivas recreativas na IAo moderada/grave poderá ser considerada para pacientes assintomáticos com a fração de ejeção do ventrículo esquerdo (FEVE) >50%, VE não dilatado (<35 mm/m^2^) e TE normal (GR-NE: IIB-C).

#### 5.3. Válvula Aórtica Bicúspide

A válvula aórtica bicúspide (VAoB) é uma malformação congênita que pode ocorrer tanto como lesão isolada quanto em associação com CC. A prevalência de VAoB isolada na população geral é de cerca de 1 a 2%, enquanto, nos pacientes com CoAo, é de 50 a 85% e, na síndrome de Turner, de 15 a 30%. A VAoB é comum em doenças cromossômicas tais como a síndrome de Down (trissomia 21), DiGeorge (22q11), síndrome de Edwards (trissomia 18) e também em outras síndromes genéticas: síndrome de Williams, de Holt-Oram, Marfan (4,7%) e de Loeys-Dietz (8,8%).^139 , 330 , 907^

Anormalidades da raiz aórtica, junção sinotubular e aorta ascendente ocorrem como parte dessa malformação. As dilatações da raiz e da aorta ascendente são comuns, mesmo em pacientes que não apresentam estenose ou insuficiência. Na EAo, é maior o risco de desenvolver dilatação aórtica grave na adolescência e início da idade adulta. Na síndrome de Marfan com VAoB e dilatação da aorta, há maior risco de ruptura aórtica espontânea. A maioria das crianças com VAoB é assintomática até a vida adulta. Em coortes pediátricas selecionadas com VAoB, mas sem estenose grave ou CC concomitante, <5% requerem intervenções valvares antes da vida adulta.^139 , 330 , 907^

Particularidades do TE/TCPE na VAoB:

– Está indicado para avaliação de sintomas e ACR de pacientes que evoluíram com EAo moderada/grave, IAo ou CoAo associadas.^370^
– Adolescentes com VAoB e síndrome de Williams geralmente apresentam: tempo total de esforço reduzido; resposta cronotrópica acelerada; resposta hipertensiva da PAS ao esforço; ausência de ISTE.^908^
– As indicações para valvoplastia por balão incluem EAo grave, pico do gradiente sistólico em repouso ≥50 mmHg sem sintomas ou ≥40 mmHg com angina, síncope e alterações do segmento ST em repouso ou esforço-induzidas.^909^

#### 5.4. Estenose Pulmonar

A estenose pulmonar (EP) é um estreitamento da válvula pulmonar, geralmente por fusão dos seus folhetos, com obstrução da via de saída do VD e redução do fluxo sanguíneo para as artérias pulmonares. É a forma mais comum de obstrução da via de saída do VD (90% dos casos).^910 , 911^

A gravidade da EP determina as condutas terapêuticas, inclusive necessidade de intervenção cirúrgica e/ou transcateter. Classificação da EP através do gradiente de pressão do VD para a artéria pulmonar: discreta entre 10 e 30 mmHg; moderada entre >30 e 60 mmHg; grave >60 mmHg ou pressão em VD maior que a pressão sistêmica.^910 , 912 , 913^

Crianças com EP discreta, com septo interventricular íntegro (EP isolada), geralmente não apresentam sintomas, mantêm ACR normal, podendo ocorrer regressão espontânea da estenose com a idade. Pacientes com EP moderada, especialmente sintomáticos, evoluem com piora da hipertrofia de VD, da obstrução da via de saída e disfunção ventricular, necessitando tratamento intervencionista. A EP grave ocorre principalmente na infância, frequentemente evoluindo com disfunção do VD, IC, insuficiência tricúspide e cianose, requerendo tratamento intervencionista precoce.^370 , 911^ Na EP isolada, em acompanhamento por 13,5 anos, ocorreu aumento de mortalidade geral (RR: 4,67; IC95%: 3,61-5,99). Pacientes com diagnóstico precoce (0 a 1 ano) tiveram o maior risco de mortalidade (RR: 10,99; IC95%: 7,84-15,45).^910 , 912 , 913^

Após intervenção valvular, a sobrevida livre de eventos a longo prazo é >90%. As complicações incluem insuficiência valvar pulmonar com possível sobrecarga de volume do VD (≈⅓ dos pacientes) e reestenose em 5 a 10% dos pacientes, especialmente no primeiro ano após a intervenção.^911 , 914 , 915^

O ECG de repouso na EP isolada discreta geralmente é normal, entretanto, crianças podem apresentar inversão de ondas T nas derivações precordiais direitas. Na moderada/grave, geralmente observa-se padrão de hipertrofia do VD, sobrecarga atrial direita (onda P *pulmonale* ), desvio do eixo QRS para direita e BRD.^388 , 911^

Particularidades do TE/TCPE na EP isolada:

– É útil na avaliação pré-participação em programas de exercícios físicos, auxilia na avaliação de sintomas fornecendo informações diretas sobre a capacidade do VD em manter o débito cardíaco durante condições de aumento da carga de trabalho. A pressão sistólica do VD, avaliada através de ecocardiografia de estresse físico, normalmente é elevada em repouso, aumentando durante o esforço.^87 , 906^
– Quanto à ACR geralmente: na estenose discreta está normal; na moderada cursa com redução; na grave evolui com redução acentuada, sendo sintomática e com pior qualidade de vida; melhora após a intervenção.^916 – 918^
– A resposta cronotrópica geralmente é normal, independente da gravidade da estenose.^87^
– É muito rara a ocorrência de ISTE e podem ocorrer arritmias esforço-induzidas.^919^
– O TCPE após ≈8 anos de valvoplastia pulmonar por balão em EP grave demonstrou comportamento normal do VO_2_pico (32,63±8,38 mL/kg/min), FCpico (174,88±5,01 bpm), queda da FC 1 ° minuto da recuperação (28,04±4,70 bpm), PASpico (164,02±11,03 mmHg), PADpico (84,42±7,63 mmHg), CVF (2,56±0,39 L) e VEF1 (2,43±0,34 L).^380^ Arritmia ventricular monomórfica esforço-induzida ocorreu em 10,9% e nenhuma criança apresentou alteração do segmento ST.^900^

#### 5.5. Insuficiência Pulmonar

A insuficiência pulmonar (IP) costuma ser assintomática e bem tolerada na infância. Entretanto, raramente, a IP pode agravar-se progressivamente causando dilatação e disfunção do VD, intolerância aos exercícios, TV e MSC. Pacientes com IP discreta/moderada geralmente são assintomáticos. Na IP grave, frequentemente observa-se intolerância aos esforços com dispneia, devido à incapacidade em aumentar o débito do VD. Se houver insuficiência do VD, pode ocorrer congestão hepática, ascite e edema de membros inferiores. A remodelação atrial e ventricular direita confere maior risco de arritmia com tontura e/ou síncope. Sintomas esforço-induzidos, intolerância progressiva aos esforços, IC e arritmias sustentadas são marcadores de má evolução que indicam a necessidade de intervenção/reparo da válvula.^97 , 709 , 920^

O ECG de repouso pode revelar desvio do eixo dos complexos QRS para direita, padrão de hipertrofia do VD e BRD. Na IP grave, é comum a ocorrência de arritmias.^177^

Particularidades TE/TCPE na IP:

– Em coorte retrospectiva, crianças submetidas à cirurgia de troca valvar pulmonar e/ou revisão do conduto valvulado, com melhor ACR no pré-operatório (VO_2_pico previsto ≥70%), evoluíram com menor tempo de internação.^921^
– Em coorte retrospectiva, a substituição da válvula pulmonar após a correção tardia de ToF demonstrou melhora do volume do VD. Cerca de 28% dos pacientes atingiram a normalização do volume sistólico final do VD, mas sem melhora significativa da ACR.^712^
– Após implante percutâneo da válvula pulmonar, em pacientes com IP associada a outras CC, foi demonstrado que não houve melhora do VO_2_pico, QR e pulso de O_2_. Na análise multivariada, a redução do gradiente da via de saída do ventrículo direito foi o único preditor de melhora do VO_2_pico.^922^
– Pacientes com IP grave, assintomáticos, sem sobrecarga significativa do volume do VD, sem arritmias, com função sistólica do VD e TE normal podem ser considerados para a prática esportiva recreativa.^681^

#### 5.6. Estenose Mitral

Os defeitos específicos da válvula mitral na estenose valvar mitral (EM) são divididos com base na relação com seu anel, incluindo componentes valvares, supravalvares e subvalvares (cordas tendíneas e músculos papilares). A apresentação clínica varia com base no grau da obstrução valvar e sua associação com insuficiência mitral (IM), HAP secundária, doenças pulmonares e/ou outras lesões cardíacas.^923^

A EM congênita raramente ocorre na forma isolada, geralmente associando-se com CoAo, EAo e CC (anomalia de Ebstein, *cor triatriatum* , ToF etc.). Se a estenose for moderada a grave, os sintomas geralmente aparecerão no primeiro ou segundo ano de vida: déficit de crescimento, sibilância e grau variado de dispneia e palidez.^924^

No ECG de repouso, geralmente observam-se padrão de hipertrofia VD, desvio do eixo dos complexos QRS para direita e ondas P bífidas ou pontiagudas indicativas de SAE. A ocorrência de FA é muito rara.

Particularidades TE/TCPE na EM:

– Pacientes com EM discreta a moderada podem ser assintomáticos mesmo em exercícios extenuantes.
– Na EM não corrigida, é indicado na avaliação pré-participação para a confirmação do estado assintomático, devendo atingir pelo menos o nível da atividade compatível com a prática esportiva pretendida.^681^
– Na EM moderada, para a liberação de atividades de baixa e moderada intensidade, o TE deve ser normal. Recomenda-se acompanhamento anual com o TE.^681^
– Em EM moderada/grave, o aumento da FC e do débito cardíaco ao esforço podem acarretar aumento do gradiente, das pressões dos capilares pulmonares e da HAP, causando baixa tolerância ao esforço, piora de sintomas e, eventualmente, edema agudo de pulmão.^46^
– Após 6 meses de valvoplastia, verificou-se melhora na ACR e aumento do débito cardíaco.^47^

#### 5.7. Insuficiência Mitral

A insuficiência mitral (IM) é uma lesão valvar com fluxo retrógrado sanguíneo do VE para o átrio esquerdo e subsequente sobrecarga de volume no VE. Para a manutenção do débito cardíaco, podem ocorrer alterações compensatórias, como aumento da força contrátil e HVE. A IM pode progredir, causar remodelamento ventricular e, eventualmente, dilatação difusa e disfunção do VE. A sobrecarga crônica do átrio e VE prejudica a drenagem sanguínea pelas veias pulmonares, causando congestão pulmonar e sintomas de IC. A IM congênita é uma doença rara na infância, com frequente associação a outras lesões cardíacas (em até 60% dos casos).^132 , 925^

A IM discreta não produz sintomas, sendo o único sinal anormal a ausculta de sopro holossistólico apical. A insuficiência grave resulta em sintomas que podem aparecer em qualquer idade, incluindo baixo desenvolvimento físico, infecções respiratórias frequentes, fadiga aos esforços, edema pulmonar e IC congestiva.

A liberação/recomendação de exercícios físicos/prática esportiva depende da gravidade da IM, do grau de dilatação do VE, da função sistólica do VE e da HAP. Exercícios estáticos com grandes aumentos da PA podem resultar em aumento do volume regurgitante e das pressões capilares pulmonares, sendo potencialmente deletérios.^681 , 926^

O ECG de repouso na IM moderada/grave geralmente mostra ondas P bífidas (SAE) e sinais de HVE. Nos casos mais graves, é possível observar padrão de HVD.

Particularidades do TE/TCPE na IM:

– Discreta, geralmente não provoca comprometimento da ACR.
– Discreta/moderada compensada, geralmente os pacientes são assintomáticos, com boa tolerância aos esforços e ACR normal, podendo permanecer assim por anos.^927^
– Adolescentes com IM grave, assintomáticos, poderão ser liberados para atividades de baixa intensidade, caso apresentem TE normal, função do VE preservada em repouso, pressão arterial pulmonar <50 mmHg e ausência de arritmia ventricular esforço-induzida.^681^
– IM grave com disfunção de VE cursa com sintomas de IC, intolerância ao esforço e baixa ACR. O TCPE auxilia na estratificação de risco, ajustes terapêuticos e na eventual indicação de transplante cardíaco.
– Após troca ou reparo valvar para avaliação da ACR, ajustes terapêuticos e liberação/prescrição de atividades físicas incluindo reabilitação.

#### 5.8. Prolapso da Válvula Mitral

O prolapso da válvula mitral (PVM) caracteriza-se por protrusão sistólica dos folhetos da válvula mitral no átrio esquerdo, com ou sem IM. Tem predisposição genética, podendo ser primário ("não sindrômico") ou secundário ("sindrômico") a distúrbios do tecido conjuntivo: síndrome de Marfan, síndrome de Loeys-Dietz, síndrome de Ehlers-Danlos, osteogênese imperfeita, pseudoxantoma elástico e síndrome de osteoartrite. Pode ser também observado na CMH. Na população pediátrica, é frequentemente considerado benigno e assintomático. Quando sintomático, as principais queixas são: palpitações, tontura, dor torácica, dispneia, pré-síncope e síncope.^132 , 928^

O PVM em atletas adolescentes e adultos jovens, com degeneração mixomatosa da válvula, é causa relevante de MSC arritmogênica (PVM arritmogênico), com incidência anual de ≈0,2 a 1,9%. A presença de prolapso nos dois folhetos da valva, IM moderada/grave e arritmia ventricular são marcadores de maior risco de eventos. Adolescentes e mulheres jovens com espessamento dos folhetos mitrais e/ou prolapso de ambos os folhetos podem apresentar predisposição aumentada para arritmias complexas e MSC arritmogênica.^929 – 931^

O ECG de repouso é normal na maioria dos pacientes, entretanto, pode apresentar ondas T negativas em derivações de parede inferior, EVs com padrão de BRD e prolongamento do QTi (principalmente em atletas). Nos pacientes com IM crônica, pode ser observado padrão de SAE, sobrecarga ventricular esquerda (SVE) e ISTs.^931 – 933^

Particularidades do TE no PVM:

– É útil para avaliação de sintomas, determinação da tolerância ao esforço, detecção de arritmias esforço-induzidas e liberação/prescrição de exercícios físicos (incluindo atividades esportivas competitivas).^926 , 934^
– Intolerância ao esforço e ACR reduzida são frequentes.^935^
– Mesmo quando a ACR é normal, pacientes apresentam menor DP no pico do esforço.^935 , 936^
– As arritmias ventriculares esforço-induzidas com padrão de BRD e/ou complexas são marcadores de risco nos pacientes com suspeita de PVM arritmogênico.^934 , 937^
– Cerca de 38% dos adolescentes atletas com arritmias ventriculares apresentaram EVs com morfologia do BRD em repouso e/ou esforço-induzidas.^938^
– Na IM moderada/grave associada, há risco aumentado de morbimortalidade quando a função sistólica do VE e a ACR estão comprometidas. Nesses pacientes, deve ser considerado o reparo ou substituição da válvula.^370^

### 6. Dispneia e Intolerância aos Esforços

#### 6.1. Dispneia Esforço-Induzida

A dispneia esforço-induzida (DEI) é uma manifestação clínica muito comum em crianças e adolescentes, caracterizada por falta de ar, esforço respiratório, aumento da FR e desconforto torácico. É uma sensação subjetiva que pode ter várias etiologias subjacentes mesmo na ausência de doenças detectáveis. Representa motivo para interrupção do esforço em ≈52% das crianças. Mais de 14% dos adolescentes aparentemente saudáveis experimenta episódio de DEI anualmente.^178 , 939 – 941^

Os mecanismos e a fisiopatologia da dispneia envolvem interações entre o sistema cardiorrespiratório e respostas neurais. Acredita-se que a dispneia seja causada pela discordância entre a ventilação e o impulso respiratório neural. Inicialmente, as alterações respiratórias decorrentes do esforço ocorrem predominantemente por meio de aumentos do volume corrente (VC) e após atingir aproximadamente 50% da capacidade vital pelo aumento da FR. A taquipneia inicia-se quando se atinge o platô do VC. Fatores ventilatórios, incluindo desconforto torácico, trabalho respiratório intenso e distúrbios ventilatórios (com ruídos audíveis como estridor e chiado) podem contribuir para a sensação de dispneia e sua gravidade.^78 , 939^

Principais causas de DEI: asma, broncoespasmo, obstrução laríngea e disfunção das cordas vocais esforço-induzidas; anormalidades restritivas da parede torácica; doenças metabólicas (exemplos: doença de McArdle, hipotireoidismo etc.); miastenia grave; doenças CV incluindo CC, cardiomiopatias, IC, HAS, valvopatias e arritmias.^78 , 939 , 942^

Particularidades do TE/TCPE na DEI:

– Indicado para esclarecimento de sintomas e mecanismos envolvidos na dispneia, avaliação da ACR, decisões terapêuticas e para liberar/prescrever exercícios físicos.
– Sugere-se a utilização de escala visual de dispneia de Dalhousie e escala de percepção de esforço, visando quantificar o grau de comprometimento e repercussão da dispneia.^178 , 943 , 944^
– A percepção da dispneia deve ser correlacionada com a carga de esforço, VO_2_ e ventilação pulmonar em que surgiram e também no momento de sua intensidade máxima.^945^
– No TCPE, para investigação diagnóstica, deve-se realizar espirometria basal seguida de protocolo de esforço incremental máximo, com repetição da espirometria na recuperação.
– A saturação arterial de oxigênio deve ser monitorada continuamente via oximetria de pulso (SpO_2_), sendo as reduções >5% indicativas de hipoxemia esforço-induzida.
– Se associada a sibilância ou ruído respiratório audível, a DEI está frequentemente associada a asma ou broncoespasmo esforço-induzido.
– DEI com dor torácica, redução acentuada da eficiência ventilatória, com relações VE/VO_2_ e VE/VCO_2_ elevadas, indica trocas gasosas anormais, geralmente associadas a HAP.^631^
– DEI devido a doenças pulmonares restritivas associam-se a ACR reduzida (VO_2_ baixo no LV1 e no pico do esforço) e reserva ventilatória relativamente baixa.^630^
– Dispneia inexplicada com sensação de sufocamento, associada à hiperventilação, sem dessaturação ou alterações das trocas gasosas, geralmente está associada a distúrbio psicogênico e/ou transtorno do pânico.^629 , 946^

#### 6.2. Broncoespasmo Esforço-Induzido

O broncoespasmo esforço-induzido (BEI) é um fenômeno obstrutivo agudo e transitório do fluxo aéreo. Manifesta-se, geralmente, 5 a 15 minutos após a interrupção do esforço. Os sintomas são inespecíficos e de leve a moderada intensidade: aperto no peito, dor torácica, dor abdominal, tosse isolada, sibilância e dispneia. Muito raramente, ocorrem episódios graves com insuficiência respiratória que pode causar morte.^947 , 948^

Embora anteriormente tenha se usado o termo "asma esforço-induzida" (AEI) como sinônimo da BEI, não é mais recomendado fazê-lo, pois tratam-se de entidades diferentes, inclusive quanto aos critérios de diagnóstico e tratamento. Na AEI, ocorre hiperatividade brônquica e inflamação crônicas, enquanto o BEI representa o estreitamento transitório das vias aéreas (sempre associado aos esforços físicos), podendo ocorrer, inclusive, em pacientes não asmáticos. A AEI se beneficia do tratamento com corticosteroides para controlar a inflamação crônica subjacente, enquanto o BEI, na maioria dos casos, requer administração de um β2-agonista de ação curta antes dos esforços físicos.^948 , 949^

Na população pediátrica, os fatores de risco para BEI são: dermatite atópica; sensibilização a alérgenos internos; níveis elevados de IgE (sazonal e perene); fatores ambientais (exposição ao ar frio, altas pressões atmosféricas, umidade e poluentes); em crianças asmáticas, a inflamação eosinofílica das vias aéreas e os níveis de fração de óxido nítrico exalado [FeNO >20 partículas por bilhão (ppb) em pacientes sem uso de corticoide e >12 ppb nos que estão em uso].^947 , 948 , 950^

O BEI é observado em 40 a 90% das crianças asmáticas, especialmente naquelas com asma grave não controlada farmacologicamente. A prevalência na população pediátrica varia entre 7% e 35% e, nos adolescentes atletas, é de ≈23,1%. A associação de BEI e obstrução laríngea esforço-induzida ocorre em 4,8% dos adolescentes, sendo mais prevalente no sexo masculino (64,7%).^164 , 951^

Particularidades do TE/TCPE no BEI:

– O TCPE está indicado para diagnóstico do BEI, avaliação da ACR e determinação dos fatores limitantes ao esforço, avaliação da gravidade da hiperinsuflação dinâmica e avaliação da resposta às intervenções terapêuticas.^952^
– O TCPE realizado com a finalidade específica de diagnóstico do BEI é também conhecido como teste de provocação brônquica por exercício. Geralmente, é realizado em esteira ergométrica, na qual há maior propensão ao BEI.
– Recomenda-se utilizar protocolo com carga fixa de esforço em alta intensidade para provocar aumento rápido da ventilação e evitar a refratariedade ao desenvolvimento de broncoespasmo. Inicia-se com uma inclinação de 5,5% e aumento rápido da velocidade para atingir em 2 minutos pelo menos 80% da capacidade máxima prevista, devendo então ser mantida a carga de esforço. Esforço incremental utilizado nos protocolos de Bruce (esteira) ou Godfrey (bicicleta) são menos efetivos para desencadear o BEI.^953^
– Buscar atingir a carga máxima de esforço e/ou 80 a 90% da FCmax estimada entre 6 e 8 minutos. Em relação às condições do ambiente da sala, manter a temperatura entre 20 e 25 °C e a umidade relativa <50% (ar seco).^952 , 954^
– Cerca de 50% dos pacientes asmáticos sem história de BEI e ≈40% de atópicos (sem asma) podem apresentar BEI ao TE.
– É recomendável o uso de broncodilatadores antes do TE/TCPE nos casos de avaliação terapêutica.
– O diagnóstico e a quantificação da gravidade do BEI são estabelecidos pelas alterações da função pulmonar esforço-induzidas, independentemente da ocorrência de sintomas.
– O VEF1 deve ser medido no repouso e na recuperação (em 5, 10, 15 e 30 minutos após o esforço). Diferença >10% entre o valor de VEF1 de repouso e o menor valor de VEF1 nos primeiros 30 minutos após o esforço estabelece o diagnóstico de BEI.^163^
– A gravidade do BEI pode ser classificada baseada na queda percentual do VEF1 em relação ao nível de repouso: leve, se for ≥10% mas <25%. Moderada se ≥25% mas <50%; grave se ≥50%.^163^
– Pacientes com BEI leve geralmente necessitam de mais de um exame para confirmar o diagnóstico.^950 , 955^
– Caso ocorram sintomas moderados/intensos durante ou após o esforço, mesmo na ausência de queda significativa do VEF1, preconiza-se o uso de broncodilatador, o qual também pode ser necessário ao final do exame caso o VEF1 não retorne a um valor de queda menor que 10% do VEF1 de repouso.^953^

#### 6.3. Obstrução Laríngea Esforço-Induzida

A obstrução laríngea esforço-induzida (OLEI) é caracterizada como obstrução transitória das vias aéreas superiores, que ocorre tipicamente a nível supraglótico, seguida frequentemente por acometimento glótico, causando redução do fluxo de ar e dispneia aos esforços. A causa da OLEI é desconhecida, sendo os fatores de risco mais relevantes: asma; doença do refluxo gastroesofágico; doenças/fatores anatômicos das vias aéreas superiores (exemplo: disfunção de cordas vocais); hereditariedade; fatores ambientais (piora em ar frio e úmido); estresse psicológico; atividade física/esportiva em alta intensidade. É causa importante de problemas respiratórios e disfunção das vias aéreas superiores em atletas adolescentes. Para o manejo e tratamento adequados, é necessário afastar outras possíveis causas dos sintomas, tais como asma, BEI e hiper-reatividade das vias aéreas.^956 – 958^

Em geral, a prevalência de OLEI varia com a idade (mais frequente entre 11 e 18 anos), sexo (maior no sexo feminino na proporção 3:1) e nível atlético (maior em atletas competitivos de alto rendimento). Em adolescentes atletas, a prevalência é de 8,1%, sendo comum a associação com AEI (em 14 a 38% dos atletas).^164 , 951 , 959^

Os pacientes geralmente apresentam aos esforços: dispneia; desconforto respiratório; aperto na garganta; sensação de asfixia; aperto na parte superior do tórax; dor torácica; respiração ruidosa e com estridor; alterações na voz e rouquidão; tosse; inspiração prolongada; ataques de hiperventilação; reações de pânico. Atletas podem referir apenas a sensação de "respiração mais difícil".^960 , 961^

Particularidades do TE/TCPE na OLEI:

– Em relação aos exames para confirmação diagnóstica de OLEI, recomenda-se a realização em ambiente hospitalar com equipe multidisciplinar (incluindo otorrinolaringologista) e condições adequadas para atendimento das possíveis complicações.
– TE com laringoscopia nasal flexível, contínua e em esforço de alta intensidade é reconhecido como o "padrão-ouro" para o diagnóstico da OLEI. Envolve a colocação de um videolaringoscópio flexível (com gravação contínua) para a visualização da laringe em tempo real. Além do diagnóstico, permite avaliar a gravidade do fechamento laríngeo no momento mais sintomático e avaliar a eficácia terapêutica.^962 , 963^
– Idealmente em atletas, o ergômetro e protocolo de esforço devem ser o mais compatível com a atividade esportiva praticada, visando a alcançar a capacidade máxima de esforço, atingindo a maior ventilação possível.
– O exame será positivo se o paciente reproduzir seus sintomas laríngeos (associado idealmente a um platô no VO_2_ e/ou da resposta da FC – teste máximo) e imagem registrando a presença, o local e o grau da obstrução laríngea. Na presença de obstrução supraglótica e glótica concomitante, o local em que a obstrução ocorrer primeiro deve ser especificado.^956 , 964^
– O TCPE combinado com a laringoscopia contínua permite a avaliação simultânea das variáveis respiratórias e metabólicas, contribuindo para o diagnóstico diferencial de outras causas de DEI.^958 , 965^
– Principais variáveis no TCPE a serem registradas na suspeita de OLEI: ventilação pulmonar, VO_2_pico, QR e *loops* de volume de fluxo.^956^
– Geralmente, os sintomas ocorrem próximo ao pico do esforço, são mais evidentes durante a fase inspiratória, podendo estar associados a estridor (sibilância/assobio ao inspirar). Geralmente desaparecem dentro de 2 a 3 minutos após a interrupção do esforço, podendo persistir por mais tempo nos pacientes que mantiverem a hiperventilação.^956 , 963^
– Caso os sintomas/sinais iniciais não sejam reconhecidos ou haja atraso na interrupção do esforço, pode ocorrer laringoespasmo, que resulta do fechamento exacerbado da glote, impedindo totalmente a ventilação. É uma situação muito rara, ocorrendo tardiamente aos sintomas da OLEI que cursa com dessaturação, bradicardia e cianose central, requerendo tratamento imediato.

#### 6.4. Asma Esforço-Induzida

A asma é uma doença inflamatória crônica heterogênea caracterizada por uma limitação do fluxo das vias aéreas, reversível espontaneamente ou após tratamento. As principais queixas são chiado/sibilância, falta de ar, aperto no peito e tosse, sendo frequentemente desencadeados por emoções, poeira e/ou exposição a alérgenos. Frequentemente, também se observa redução da ACR, DEI, fadiga e redução da qualidade de vida. A prevalência de sintomas de asma entre adolescentes no Brasil é de ≈20 a 23%, uma das mais elevadas do mundo, sendo que apenas 12% têm diagnóstico prévio de asma.^966 – 968^

A asma esforço-induzida (AEI) é uma restrição das vias aéreas em pacientes que já apresentam uma hiperatividade brônquica e inflamação persistente (pacientes asmáticos), enquanto, no BEI, a restrição das vias aéreas é temporária, principalmente em não asmáticos. A AEI é desencadeada pelo ar frio e seco durante exercícios, causando desidratação da mucosa das vias aéreas com o aumento da osmolaridade, contração da musculatura lisa brônquica, influxo de eosinófilos/mastócitos e liberação de mediadores inflamatórios (leucotrienos, histamina, IL- 8, triptase e prostaglandinas). A AEI é observada em ≈40 a 90% das crianças asmáticas, especialmente naquelas com asma grave não controlada farmacologicamente. Principais queixas: tosse, chiado, aperto no peito e falta de ar incomum ou excesso de muco ocorrendo após exercício aeróbico extenuante e contínuo. Os sintomas geralmente começam a se manifestar 5 a 8 minutos após o início do exercício contínuo ou em 2 a 5 minutos nos casos de exercícios de alta intensidade. A AEI costuma ser confirmada em espirometria, realizada antes e após o TE/TCPE.^948 , 949 , 969^

A AEI frequentemente acarreta limitações significativas nas atividades físicas/esportes, entretanto, o exercício regular nos pacientes com controle adequado da asma é recomendado, inclusive para evitar a obesidade e outros fatores agravantes da asma. Em pacientes com AEI, é aconselhável o uso de um β2-agonista de ação curta, 5 a 20 minutos antes dos exercícios. Adicionalmente, para o tratamento da asma, pode ser necessário o uso diário de corticosteroides inalados, antagonistas dos receptores de leucotrienos ou drogas estabilizadoras de mastócitos.^947 , 969^

Particularidades do TE/TCPE na asma e AEI:

– A resposta dos asmáticos ao esforço depende do grau de obstrução das vias aéreas e da reversibilidade. Durante o esforço, a VEmin sofre aumento para atender as demandas metabólicas musculares. O aumento no VC é o mecanismo dominante na ventilação baixa a moderada. Aumentos adicionais da VEmin em altos níveis de esforço são devidos principalmente ao aumento na FR.^947 , 969^
– Na asma controlada, o esforço geralmente é interrompido devido à fadiga periférica, embora certo grau de limitação do fluxo expiratório também possa ocorrer. Normalmente, a RV não é esgotada e o fluxo máximo não é alcançado, mesmo durante o esforço máximo.^969 , 970^
– Em ≈30% dos pacientes com asma grave ocorre restrição ventilatória significativa e comprometimento da ACR. Pacientes com VEF1 <80% apresentam menor RV. A queda percentual do VEF1 correlaciona-se com valores aumentados do VE/VO_2_ e VE/VCO_2_.^967 , 971 , 972^
– A maioria dos pacientes não apresenta hipoxemia ou hipercapnia clinicamente significativa.
– Os aumentos da desigualdade ventilação/perfusão, da tensão alvéolo-arterial de oxigênio e do espaço morto fisiológico parecem estar associados à presença de broncoespasmo.^947 , 969^
– Pacientes com obstrução grave e pouco reversível das vias aéreas podem apresentar restrição mecânica na ventilação e sintomas esforço-induzidos que mimetizam doença pulmonar obstrutiva crônica (DPOC).^954 , 969^
– Pacientes com asma e/ou BEI associado geralmente apresentam estridor/sibilos expiratórios com a dispneia/sintomas atingindo sua maior intensidade entre 3 e 15 minutos após a interrupção do esforço. Por essa razão, alterações anormais da função pulmonar necessárias para estabelecer o diagnóstico de asma e/ou BEI são avaliadas em espirometria na fase pós-esforço através principalmente do VEF1.
– O TCPE é realizado como parte do teste de provocação brônquica por exercício. Geralmente, é realizado em esteira ergométrica, na qual há maior propensão à AEI. Na população pediátrica, tem-se preferido o critério de redução do VEF1 ≥12% (em vez de ≥10%) por sua maior especificidade, com VPP do exame de 94% e acurácia de 70%.^966 , 968^

### 7. Anemia/Doença Falciforme

Doença falciforme (DF) é uma hemoglobinopatia genética, autossômica recessiva, resultante de defeitos na estrutura da hemoglobina (Hb), associados ou não a defeitos em sua síntese. As mutações herdadas podem ser: homozigóticas (SS, genótipo denominado como anemia falciforme); heterozigótico simples (traço falciforme) com um gene normal da Hb associado a um gene variante; heterozigótico composto com gene variante (SC, SD, SE, S betatalassemia, S alfatalassemia ou S mut) em combinação com defeito estrutural ou de síntese da Hb, denominada genericamente de talassemia. Estima-se que 4% da população brasileira tenha o traço falciforme e que 25.000 a 30.000 pessoas tenham a anemia falciforme (SS) ou talassemia.^973^

Na DF, a Hb defeituosa hipossolúvel (HbS), quando desoxigenada nos leitos capilares leva à falcização dos glóbulos vermelhos, causando hemólise, anemia crônica normocítica e episódios de vaso-oclusão com isquemia associada. A DF apresenta alta taxa de morbimortalidade, com eventos agudos potencialmente letais: crises vaso-oclusivas (crises falcêmicas) com dor intensa, lesões isquêmicas teciduais e possíveis danos em todos os órgãos, incluindo acidente vascular cerebral (AVC), nefropatia, retinopatia, úlceras em membros inferiores, priapismo, necrose avascular etc.; e a síndrome torácica aguda (STA), cujas principais causas no adulto são embolia gordurosa, infecção pulmonar, crise asmática, infarto do arcabouço ósseo torácico e trombose in situ/embolia da artéria pulmonar, que geralmente precede os desfechos fatais.^974^

Nas crianças, é comum a observação de hipoxemia crônica persistente com SpO_2_ <94%. Quando adequadamente diagnosticadas e tratadas, quase todas as crianças com anemia falciforme sobrevivem até a idade adulta, mas com redução na expectativa de vida (≈20 anos).

A DF geralmente cursa com intolerância aos exercícios e redução da ACR devido a:^168 , 975^

– Baixos níveis de atividade física por dores articulares crônicas.
– Exacerbação de resposta pró-inflamatória em consequência de exercícios intensos.
– Redução da capacidade de transporte de O_2_ relacionada ao baixo nível de Hb.
– Disfunção cardíaca resultante de anemia crônica.
– Disfunção do parênquima pulmonar causada por episódios repetidos de STA.
– Doença vascular pulmonar e HAP.
– Doença vascular periférica/miopatia devido à oclusão microvascular frequente e repetida.

A DF pode evoluir com cardiomiopatia restritiva (CMR), caracterizada por disfunção diastólica do VE com função sistólica normal e dilatação do átrio esquerdo. Essa combinação resulta em HAP secundária leve, velocidade elevada do jato regurgitante da valva tricúspide e aumento de mortalidade. Lesões isquêmicas do sistema de condução, fibrose e extensa dilatação das câmaras cardíacas são potenciais etiologias para arritmia e MSC na CMR.^976 , 977^

Indicações do TE/TCPE na DF em crianças e adolescentes:

– O TCPE permite avaliar ACR, eventuais limitações aos esforços e prescrever exercícios físicos, inclusive na RCV.^978 , 979^
– A avaliação da função pulmonar (incluindo o VEF1 e relação VEF1/CVF) deve ser feita a cada 1 a 3 anos devido à alta prevalência de disfunção pulmonar restritiva (em ≈26% dos pacientes), obstrutiva (em ≈35 a 39%) e hiper-reatividade das vias aéreas (em 70%). Intervalos mais curtos de repetição devem ser adotados, especialmente nos pacientes com dispneia persistente, história de asma e/ou sibilos recorrentes ou elevações acentuadas dos marcadores hemolíticos.^980 , 981^
– Episódios dolorosos agudos durante o exame são raros (0,43 a 1% dos pacientes).^979^
– É comum a ocorrência de alterações isquêmicas transitórias e dessaturação durante o exame, mas que não resultam em arritmias ou outras complicações.^979^ Geralmente, metade dos pacientes apresenta ISTE, dos quais 31% com DAC.
– Pacientes com anemia geralmente apresentam FC elevada, VE/VCO_2_ aumentado, anormalidade do pulso de O_2_, reduções VO_2_ no LV1 e no pico do esforço.
– Em ⅔ dos pacientes que evoluem com doença vascular pulmonar apresentam limitação ao esforço com anormalidades nas trocas gasosas: diferença alvéolo-arterial da tensão de oxigênio (PaO_2_) >30 mmHg, relação anormal entre espaço morto e VC (VD/VT) e valores muito altos de VE/VCO_2_.
– Observa-se declínio do VEF1 de 0,3% a cada ano, independente do sexo, presença de asma, concentração de Hb, incidência de dor aguda intensa, episódios de STA e de terapia com hidroxiuréia.^982^
– Crianças com STA geralmente apresentam menor capacidade pulmonar total (CPT) e redução de VEF1. A idade e o sexo masculino estão associados a menores valores de VEF1 e da relação VEF1/CVF.^981^
– Geralmente, observa-se recuperação lenta da FC no 1 ° ao 5 ° minutos pós-esforço, independentemente da ACR. A recuperação lenta da FC sugere comprometimento da atividade vagal, que piora com o aumento da idade.^983^
– Pacientes com Hb-SS apresentaram saturação média de oxigênio, CVF e %VEF1 menores, sendo o resultado anormal da espirometria em ≈70,4% dos pacientes causado por defeitos predominantemente restritivos.^984^
– A oximetria de pulso geralmente subestima a saturação arterial, mas em valores clinicamente insignificantes. Esse achado em parte é devido à carboxiemoglobina (COHb) e metemoglobina (MetHb) elevadas na DF. A co-oximetria de pulso não invasiva pode auxiliar na medição dos níveis de COHb e MetHb e melhorar a precisão da determinação da saturação.^985^
– A dessaturação de oxigênio esforço-induzida é observada em ≈18% das crianças com talassemia e em ≈34% das crianças com anemia falciforme.^986^

## Parte 4 – Teste Ergométrico Associado aos Métodos de Imagem em Cardiologia

### 1. Estresse Cardiovascular Associado aos Métodos de Imagem em Cardiologia

#### 1.1. Imagem Nuclear/Cintilografia Perfusional Miocárdica

A cardiologia nuclear permite a avaliação de perfusão e viabilidade miocárdica, função ventricular, perfusão pulmonar e detecção de processos inflamatórios na população pediátrica.^214 , 987^

A utilização da cintilografia perfusional miocárdica (CPM; do inglês *Single Photon Emission Computed Tomography "SPECT")* é limitada devido à radiação ionizante e seu potencial impacto ao longo da vida, particularmente em portadores de CC. O aumento de risco de câncer dá-se pela radiossensibilidade inerente às crianças.^988^

A evolução tecnológica na última década e o desenvolvimento de protocolos com baixas doses de radiação dedicados às crianças abrem novas perspectivas para o uso de imagem nuclear em pediatria.^214^

É cada vez mais crescente o emprego da ressonância magnética cardíaca (RMC) associada à perfusão miocárdica na população pediátrica. A RMC é considerada o método de escolha para quantificação dos volumes biventriculares e da função ventricular, especialmente do VD. A viabilidade e a isquemia também podem ser avaliadas pela tomografia por emissão de pósitrons/tomografia cardíaca (PET/CT).^988 , 989^

A história do paciente e o planejamento da aquisição das imagens são essenciais para garantir a viabilidade e valor diagnóstico dos exames. Detalhes da anatomia cardíaca, procedimentos cirúrgicos e percutâneos prévios ajudam a distinguir achados normais dos patológicos. A dose de radionuclídeo é baseada no peso da criança, no protocolo e nos métodos de aquisição de imagem. Preferencialmente, deve-se realizar primeiro a geração de imagens de estresse. Recomenda-se a utilização de câmeras para SPECT, PET ou imagem híbrida de última geração.^987 , 990^

**Figura 7:**
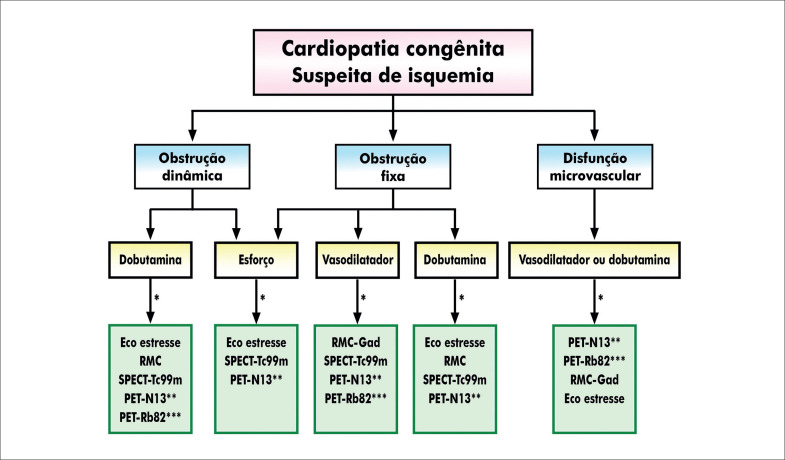
Seleção do método de imagem cardiovascular e protocolo de estresse em pacientes pediátricos com cardiopatia congênita e suspeita de isquemia. Exemplos de causa de obstrução dinâmica: artérias coronárias anômalas, compressão de stents, pontes miocárdicas e vasoespasmo. Exemplos de causa de obstrução fixa: obstrução coronária aterosclerótica, estreitamento cirúrgico dos óstios coronários e espessamento da íntima. Exemplos de causa de disfunção microvascular: manipulação cirúrgica das artérias coronárias na cirurgia de troca arterial, hipercolesterolemia familiar e lúpus eritematoso sistêmico. * Métodos de imagem cardiovascular apresentados sequencialmente, conforme a escolha do estressor. ** Disponível no Brasil somente em pesquisa; *** Não disponível no momento no Brasil. Eco estresse: ecocardiografia sob estresse; RMC: ressonância magnética cardíaca; PET: tomografia por emissão de pósitrons; SPECT: tomografia computadorizada por emissão de fóton (do inglês Single Photon Emission Computed Tomography); N-13 = amônia N-13; Rb-82: rubídio-82; Tc-99m: tecnécio 99m; Gad: gadolínio. Adaptado de: Partington SL et al. Clinical applications of radionuclide imaging in the evaluation and management of patients with congenital heart disease.^987^

Em nosso meio, são utilizadas as modalidades de estresse físico ou farmacológico (dipiridamol, adenosina ou dobutamina), que apresentam sensibilidade e especificidade semelhantes na análise das imagens de perfusão. A escolha da modalidade de estresse depende principalmente da idade da criança e de limitações ou contraindicações para a realização de esforço físico ( Figura 7 ). As principais contraindicações das modalidades de estresse são apresentadas na Tabela 41 . O monitoramento por oximetria de pulso é recomendado nos pacientes com CC, particularmente nos casos de *shunt* direita-esquerda e/ou malformações arteriovenosas pulmonares.^214 , 243 , 991 , 992^

**Tabela 41:** Contraindicações das modalidades de estresse cardiovascular em população pediátrica^214 , 243 , 991 , 992^

Estresse	Contraindicações
Estresse físico	Vide contraindicações absolutas para TE/TCPE – Quadro 2.
Vasodilatadores (dipiridamol/adenosina)	Bloqueios atrioventriculares avançados; hipotensão; hipertensão acentuada; bradicardia sinusal; doença broncoconstritiva ou broncoespástica ativa com uso regular de inaladores; hipersensibilidade conhecida aos vasodilatadores.
Dobutamina	Hipertensão grave; angina instável; estenose valvar aórtica grave; arritmias complexas; cardiomiopatia hipertrófica obstrutiva; miocardite; endocardite; pericardite.
Atropina	Glaucoma de ângulo estreito; miastenia grave; uropatia obstrutiva; distúrbios gastrointestinais.

Metodologia do estresse físico para a CPM:^993^

– É feito através do TE ou TCPE, adicionando maior valor diagnóstico e prognóstico aos métodos de imagem por abordar parâmetros clínicos, hemodinâmicos, metabólicos e eletrocardiográficos.
– As escolhas do ergômetro e do protocolo seguem os mesmos critérios utilizados nos exames de TE/TCPE em crianças e adolescentes, que constam nesta diretriz.

Metodologia dos estresses farmacológicos para a CPM:

– As doses dos estressores farmacológicos (dipiridamol, dobutamina e adenosina) para crianças são as mesmas utilizadas em adultos. A realização dos estresses deve seguir as orientações gerais para adultos.^1^
– Independentemente do estressor, deve-se monitorizar os sinais e sintomas clínicos e registrar ECG, PA e FC durante todo o exame.
– A adenosina é um estressor farmacológico que causa vasodilatação coronariana, administrado por via intravenosa (infusão contínua, 140 μg.kg^-1^.min^-1^, por 4 a 6 min). Seus efeitos colaterais geralmente são leves e desaparecem rapidamente após a interrupção/término da infusão: broncoespasmo, devido à ativação dos receptores A2B e A3; BAV, devido à ativação de receptores A1; vasodilatação periférica, por ativação dos receptores A2B; rubor, dispneia e náusea.^990 , 994 , 995^ A metil-xantina (cafeína) contida em alimentos, bebidas e medicamentos interfere com a adenosina (vide Anexo 5 ), devendo ser suspensa pelo menos 12 horas antes do exame.^995^
– O dipiridamol é um vasodilatador coronariano que age por inibição da enzima adenosina-deaminase, degrada a adenosina endógena, bloqueia a recaptação da adenosina pela membrana celular com aumento da concentração extracelular, causando a vasodilatação coronária e sistêmica. A dose preconizada é de 0,56 mg.kg^-1^, até o máximo de 60 mg diluídos em 50 mL de soro fisiológico, com administração intravenosa em 4 minutos, podendo ser realizada manualmente (sem bomba de infusão). Sua meia-vida biológica é de ≈45 minutos. Os principais efeitos colaterais são a dor torácica, cefaleia e tontura, podendo ser revertidos com a administração de aminofilina intravenosa, feita somente 2 minutos após a injeção do radiotraçador.^993 , 995 – 998^ As metil-xantinas (vide Anexo 5 ) devem ser suspensas pelo menos 24 horas antes do exame.^995^
– A dobutamina promove a elevação do consumo de oxigênio miocárdico, com administração intravenosa em bomba de infusão. A dose inicial é de 5 a 10 μg.kg^-1^.min^-1^ em 3 minutos, sendo seguida por doses incrementais de 20 *μ* g.kg^-1^.min^-1^, 30 *μ* g.kg^-1^.min^-1^ até o máximo de 40 *μ* g.kg^-1^.min^-1^.^999 , 1000^ Nos pacientes que não alcançarem a FC submáxima e sem evidências de isquemia, pode-se associar atropina intravenosa na dose de 0,01 mg.kg^-1^ (dose unitária máxima de 0,25 mg).^996^ O radiotraçador deve ser injetado na FC alvo (geralmente definida como 85% da FCmax para a idade) mantendo-se a infusão de dobutamina por mais 1 minuto. A reversão dos efeitos adversos é feita com betabloqueadores de ação curta (por exemplo: metoprolol ou esmolol), injetados via intravenosa após o primeiro minuto da administração do radiotraçador.^1001^
– Avaliar a necessidade de restrição de volume a ser infundido em paciente com IC, cardiomiopatias, CC complexas e insuficiência renal.
– Além do médico habilitado responsável pelo exame, sugere-se o acompanhamento por pediatra.

Particularidades na perfusão miocárdica:

1. **TGA:** as mortalidades precoce e tardia estão associadas às complicações coronarianas.^1002 , 1003^ No seguimento pós-operatório, a indicação de reintervenção baseia-se mais na presença de isquemia pela CPM do que nos achados angiográficos.^220 , 1004^
  Defeitos de perfusão diagnosticados pela CPM ocorrem em 5 a 24% dos pacientes após correção cirúrgica, podendo persistir por mais de 10 anos ( Figura 7 ). Lesões angiográficas após correção nem sempre estão associadas a processo estenótico em evolução.^1005 , 1006^
  A CPM inicial permite triar os pacientes quanto à evolução: se normal, geralmente ocorre estabilização/resolução da isquemia com o tempo; se anormal geralmente corresponde à piora da isquemia.^22 , 1007 , 1008^
2. **DK:** a CPM é útil e segura no seguimento da progressão da estenose coronariana, com sensibilidade de 90% e especificidade entre 85 e 100% na detecção de isquemia.^218 , 998^ Cerca de 12 a 19% das crianças com aneurismas coronários têm padrão perfusional anormal (fibrose e/ou isquemia).^216 , 219 , 398^
  A CPM está indicada no acompanhamento tardio (de 1 a 5 anos) de crianças com aneurismas coronarianos (incluindo aneurismas pequenos e/ou resolvidos) e/ou sintomas/disfunção ventricular ( Figura 7 ).^18 , 37 , 214^
  Em adolescentes com história de DK na infância, a PET com amônia N-13 demonstrou diminuição na reserva coronariana por disfunção endotelial de longo prazo.^1009 , 1010^
3. **Cardiomiopatias:**
  – Na CMH, a CPM auxilia na pesquisa de isquemia, estratificação de risco e manejo terapêutico.^223^ A isquemia miocárdica pode estar relacionada à diminuição da perfusão subendocárdica nos segmentos hipertrofiados, compressão de pequenos vasos intramurais e ponte miocárdica.^214 , 1011^ Acredita-se que a isquemia microvascular esteja envolvida na disfunção sistólica e diastólica.^223 , 1012^ Os defeitos de perfusão miocárdica na CPM com 99mTc-MIBI podem refletir um processo isquêmico, sendo importante preditor de eventos clínicos adversos e morte.^223^
  – Na cardiomiopatia dilatada, a CPM é raramente empregada, pois a etiologia isquêmica é rara em crianças. Em situações especiais, como na anemia falciforme, a avaliação da função microvascular pode auxiliar na identificação do possível mecanismo de dano ventricular (dilatação e/ou disfunção do VE).^214 , 1013^
4. **Transplante cardíaco:** a principal complicação a longo prazo após o transplante é a doença vascular do enxerto (DVE), causa relevante de morte e retransplante. Na DVE, a CPM permite avaliar o envolvimento de artérias coronárias (distais e proximais) na disfunção sistólica e o aumento das pressões de enchimento do VE.^224 , 1014^

#### 1.2. Ecocardiografia sob Estresse

A ecocardiografia sob estresse (EcoE) é uma técnica de imagem cardiovascular que fornece imagens cardíacas em tempo real permitindo avaliar: anatomia cardíaca; função sistólica e diastólica; áreas de isquemia miocárdica; reserva coronariana; e estratificar o risco nas valvopatias, IC e CC (reparadas ou não). As principais indicações da EcoE na prática da cardiologia pediátrica encontram-se na Tabela 12 .

Vantagens da EcoE: disponível em nosso meio; na maioria dos pacientes, pode ser realizada sem sedação; não gera exposição à radiação, situação relevante no monitoramento periódico de CC. Principais limitações: janelas acústicas inadequadas em crianças com déficit de crescimento (secundário a CC) ou por alterações torácicas pós-cirúrgicas; arritmias cardíacas complexas (exemplos: TV, BAVT etc.); necessidade de uso de medicamentos que podem afetar os parâmetros do exame (betabloqueadores, diuréticos, antiarrítmicos etc.). A Tabela 41 apresenta as principais contraindicações das modalidades de estresse CV utilizados na EcoE.

Para a adequada aquisição e interpretação das imagens ecocardiográficas, recomenda-se avaliar a existência de cardiopatias (principalmente CC), o quadro clínico do paciente, história prévia de cirurgias e utilização de MP/CDI.

Os principais estressores utilizados na população pediátrica são o físico (TE) e o farmacológico. O agente farmacológico (dobutamina) é mais utilizado em crianças menores, enquanto o exercício físico é preferido em crianças ≥8 anos, cooperativas e com habilidades para se exercitar em esteira ou bicicleta ( Tabela 42 ). Independente do estressor, deve-se monitorizar os sinais e sintomas clínicos, bem como registrar ECG, PA e FC durante todo o exame.^234 , 235^

**Tabela 42:** Vantagens e desvantagens das diferentes modalidades de estresse em população pediátrica^234 , 235^

	Esforço (TE) *	Dobutamina
Idade preconizada	≥8 anos	Qualquer idade
Anestesia/sedação	Não	Se indispensável, em <6 anos
Resposta da frequência cardíaca	Geralmente submáxima	FC alvo (geralmente submáxima)
Resposta da pressão arterial	Resposta máxima	Variável
Inotropismo máximo	Sim	Sim
Retorno venoso	Aumento	Sem aumento/diminuição
Aquisição de imagens	Possibilidade de artefatos **	Mais fácil
Aptidão cardiorrespiratória	Sim	Não
Repercussão funcional	Sim	Não
Risco de complicações	Baixo	Baixo
Disponibilidade da modalidade de estresse	Moderada	Alta

* Teste ergométrico em esteira, cicloergômetro de mesa ou bicicleta ergométrica.

** Artefatos respiratórios e por movimentação.

Achados indicativos para interrupção do estresse: aparecimento de sintomas (exemplo: angina limitante); ocorrência ou piora de anormalidade contrátil da parede ventricular; ISTs ≥2 mm; queda da PAS >15 mmHg; arritmia complexa e/ou com repercussão hemodinâmica; alcançar FC alvo; atingir a dose máxima do estressor farmacológico; outros efeitos adversos de agente farmacológico.

##### 1.2.1. Metodologia do Estresse Farmacológico

###### 1.2.1.1. Dobutamina^229 , 234 , 1015 , 1016^

A dobutamina é o agente farmacológico mais utilizado na população pediátrica. Tem efeito inotrópico e cronotrópico positivo, aumentando a demanda de O_2_ pelo miocárdio. Quando essa demanda não é atendida, causa isquemia miocárdica e anormalidades da motilidade da parede ventricular. Em contraste com o estresse físico, não acarreta aumentos do retorno venoso e da pré-carga, gerando maior alteração nas dimensões diastólicas finais do VE. Permite uma recuperação mais lenta da FC, com aquisição de imagens por tempo maior.^243^

Em crianças <8 anos, a EcoE com dobutamina pode requerer anestesia geral ou sedação profunda. As imagens são adquiridas no repouso e após cada aumento na dose do estressor.

Os protocolos da EcoE com dobutamina são semelhantes aos protocolos para adultos. Geralmente, a infusão de dobutamina começa com 5 *μ* g.kg^-1^.min^-1^ com aumento da dose em intervalos de 3 a 5 minutos (para 10, 20, 30, 40 e 50 *μ* g.kg^-1^.min^-1^). A FC alvo geralmente é definida como 85% da FCmax para a idade. Caso não se atinja a FC alvo com a dose máxima de dobutamina, pode-se administrar simultaneamente, a cada 1 a 2 minutos, atropina na dose de 0,01 mg.kg^-1^ (limites: 0,25 mg por dose; dose máxima total de 1 a 2 mg).^243^ Na avaliação da reserva contrátil cardíaca, a dobutamina pode ser usada em doses baixas/moderadas (entre 5 e 20 *μ* g.kg^-1^.min^-1^) em infusão contínua.^229^

Os efeitos colaterais incluem: palpitações, náuseas, cefaleia, calafrios, urgência urinária, ansiedade, angina, hipotensão, hipertensão e arritmia. Os efeitos colaterais geralmente desaparecem com o término/suspensão da infusão, devido à meia-vida curta da dobutamina. Esmolol (dose de 0,5 mg.kg^-1^) deve estar disponível para reverter reações adversas mais intensas e/ou isquemia.^249^

###### 1.2.1.2. Vasodilatadores^229 , 234 , 1016^

A EcoE com vasodilatador (adenosina ou dipiridamol) induz o aumento do fluxo coronariano, sendo utilizada na avaliação de motilidade miocárdica, isquemia e viabilidade miocárdica. A adenosina é infundida em uma dose máxima de 140 *μ* g.kg^-1^.min^-1^, com imagens simultâneas durante 4 minutos.

O dipiridamol é administrado em duas etapas, com imagens contínuas: a primeira etapa com uma dose de 0,56 mg.kg^-1^ durante 4 min; a segunda etapa é realizada se não houver efeitos adversos, utilizando dose de 0,28 mg.kg^-1^ em 2 minutos. A aminofilina deve estar disponível para reverter reações adversas ao dipiridamol.

##### 1.2.2. Metodologia do Estresse Físico^1015 , 1017^

A EcoE com estresse físico é realizada em crianças ≥8 anos, capazes de realizar o TE. O esforço físico é um estressor fisiológico, devendo ser o método preferencial, sempre que possível.^229^ O estresse físico permite aumentar a FC, a função contrátil, a PA e o retorno venoso ao coração e determinar o VO_2_ e débito cardíaco.

Os ergômetros mais utilizados na EcoE são a esteira ergométrica e cicloergômetro (vertical, supino e semissupino) utilizando protocolos específicos. A ecocardiografia basal deve ser obtida em decúbito dorsal e também na posição em que será realizado o estresse físico. Quando utilizada a esteira, a aquisição de imagens ecocardiográficas é realizada antes do esforço e imediatamente após o término do esforço (dentro de 60 a 90 s). No caso do cicloergômetro, é realizada antes e durante todas as fases do esforço (incluindo o pico). A aquisição de imagens durante o esforço é mais desafiadora em relação a artefatos de movimento e respiração.

Além disso, como a FC em crianças pode cair muito rapidamente durante a recuperação, a interpretação dos resultados pode ser comprometida. Para obter informações durante o esforço, o teste em bicicleta é mais adequado.

Além dos achados indicativos para interrupção do estresse apresentados no início desta sessão, recomenda-se observar os critérios de interrupção do esforço constantes na Tabela 42 .

## ANEXOS

### Anexo 1 Principais leis e resoluções pertinentes ao TE e TCPE em crianças e adolescentes

**Table:**

Aspectos legais	Referência
O médico guardará sigilo a respeito das informações de que detenha conhecimento no desempenho de suas funções, com exceção dos casos previstos em lei. É vedado: – Delegar a outros profissionais atos ou atribuições exclusivas da profissão médica. – Deixar de assumir responsabilidade sobre procedimento médico que indicou ou do qual participou, mesmo quando vários médicos tenham assistido o paciente. – Acumpliciar-se com os que exercem ilegalmente a medicina ou com profissionais ou instituições médicas com prática de atos ilícitos. – Deixar de obter consentimento do paciente ou de seu representante legal após esclarecê-lo sobre o procedimento a ser realizado, salvo em caso de risco iminente de morte. – Deixar de garantir ao paciente o exercício do direito de decidir livremente sobre sua pessoa ou seu bem-estar, bem como exercer sua autoridade para limitá-lo. – Deixar de elaborar prontuário legível para cada paciente. – Revelar sigilo profissional relacionado a paciente criança ou adolescente, desde que esses tenham capacidade de discernimento, inclusive a seus pais ou representantes legais, salvo quando a não revelação possa acarretar dano ao paciente. – Deixar de obter do paciente ou de seu representante legal o termo de consentimento livre e esclarecido para a realização de pesquisa envolvendo seres humanos, após as devidas explicações sobre a natureza e as consequências da pesquisa. § 1° No caso de o paciente participante de pesquisa ser criança, adolescente, pessoa com transtorno ou doença mental, em situação de diminuição de sua capacidade de discernir, além do consentimento de seu representante legal, é necessário seu assentimento livre e esclarecido na medida de sua compreensão.	Código de Ética Médica – Resoluções N ° 2.217/2018, 2.222/2018 e 2.226/2019 do CFM.^1018 – 1020^
Determina que, para a área de atuação em ergometria, é necessária: formação de 1 (um) ano; ter concluído Residência Médica em Cardiologia para realizar a formação; após formação, realizar concurso da AMB/Sociedade Brasileira de Cardiologia para obtenção do Título de Atuação; pré-requisito para o concurso, além da formação, ter o Título de Especialista em Cardiologia da AMB.	Resolução N ° 2.380/2024 do CFM/Portaria CME N ° 1/2024.^1021^
Considerando ser recomendável a obtenção prévia de termo de consentimento livre e esclarecido assinado pelo paciente ou seu representante legal, no caso de menores de 18 anos de idade. Considerando que, em se tratando de menores de idade, o seu representante legal deva permanecer na sala de exame. O teste ergométrico deve ser individualizado e realizado, em todas as suas etapas, por médico habilitado e capacitado para atender a emergências cardiovasculares, tornando imprescindível, para tal, sua presença física na sala. Por ser ato médico privativo, caracteriza-se como falta ética a delegação para outros profissionais da realização do teste ergométrico. As condições adequadas para a realização do teste ergométrico estão previstas no Manual de Fiscalização do CFM.	Resolução N ° 2.021/13 do CFM.^1022^
Critérios norteadores da propaganda em medicina, conceituando os anúncios, a divulgação de assuntos médicos, o sensacionalismo, a autopromoção e as proibições referentes à matéria.	Resolução N ° 2.336/2023 do CFM.^1023^
Garantir a privacidade e a confidencialidade dos dados e informações armazenadas digitalmente dos pacientes; organizar bancos de dados seguros e confiáveis; garantir a transmissão de dados e informações em segurança; fazer cópia de segurança na medida da possibilidade.	Resolução N ° 1.821/2007 do CFM.^272^
Art. 226. A família, base da sociedade, tem especial proteção do Estado. § 4° Entende-se, também, como entidade familiar a comunidade formada por qualquer dos pais e seus descendentes. Art. 229. Os pais têm o dever de assistir, criar e educar os filhos menores, e os filhos maiores têm o dever de ajudar e amparar os pais na velhice, carência ou enfermidade.	Constituição da República Federativa do Brasil.^1024^
"Art. 5. A menoridade cessa aos 18 anos completos, quando a pessoa fica habilitada à prática de todos os atos da vida civil. Parágrafo único. Cessará, para os menores, a incapacidade: pela concessão dos pais, ou de um deles na falta do outro, mediante instrumento público, independentemente de homologação judicial, ou por sentença do juiz, ouvido o tutor, se o menor tiver 16 anos completos; pelo casamento; pelo exercício de emprego público efetivo; pela colação de grau em curso de ensino superior; pelo estabelecimento civil ou comercial, ou pela existência de relação de emprego, desde que, em função deles, o menor com dezesseis anos completos tenha economia própria." "Art. 186. Aquele que, por ação ou omissão voluntária, negligência ou imprudência, violar direito e causar dano a outrem, ainda que exclusivamente moral, comete ato ilícito."	Código Civil Brasileiro – Lei 10.406 de 2002.^1025^
Capítulo III, Art. 6° – São direitos básicos do consumidor: A proteção da vida, saúde e segurança contra os riscos provocados por práticas no fornecimento de produtos e serviços considerados perigosos ou nocivos; A educação e divulgação sobre o consumo adequado dos produtos e serviços, asseguradas a liberdade de escolha e a igualdade nas contratações; A informação adequada e clara sobre os diferentes produtos e serviços, com especificação correta de quantidade, características, composição, qualidade, tributos incidentes e preço, bem como sobre os riscos que apresentem.	Código de Proteção ao Consumidor – Direitos Básicos do Consumidor – Lei N ° 8.078 de 11 de setembro de 1990.^1026 , 1027^

Art.: artigo; CFM: Conselho Federal de Medicina; CME: Comissão Mista de Especialidades; AMB: Associação Médica Brasileira.

### Anexo 4 Dados relacionados à aptidão cardiorrespiratória (VO2max previsto), duplo-produto e OUES em população pediátrica aparentemente saudável e com cardiopatia

**Table:**

Material	Faixa etária (anos)	Localização na publicação	DOI da publicação	Disponível em
**Aparentemente saudável:**				
Gráficos de percentis de VO_2_max por sexo e idade.^176^	8-18	Figura 2	10.1513/AnnalsATS.201611-912FR	https://www.atsjournals.org/doi/10.1513/AnnalsATS.201611-912FR
Gráficos de percentis de VO_2_max por sexo e idade.^1028^	12-18	Figura 2 e Figura 3	10.1016/j.amepre.2011.07.005	https://linkinghub.elsevier.com/retrieve/pii/S0749-3797(11)00491-0
Tabela de VO_2_max por sexo e faixa etária na população brasileira.^1029^	7-12 e 13-19	Tabela 6	10.5935/2359-4802.20190057	https://www.scielo.br/j/ijcs/a/x8bB3qQHQKCXHRbZRbpXMrm/?lang=en
Tabela DP em repouso e DP pico em altitude moderadamente elevada.^1030^	4-18	Tabela 3	10.1016/j.acmx.2013.04.003	https://www.elsevier.es/es-revista-archivos-cardiologia-mexico-293-articulo-cardiopulmonary-exercise-testing-in-healthy-S1405994013000621
Gráfico de percentis de OUES (por sexo e idade) e equações de previsão.^622^	8-19	Figura 2 e Tabela 2	10.1177/2047487315611769	https://academic.oup.com/eurjpc/article-lookup/doi/10.1177/2047487315611769
Gráfico do comportamento médio de OUES (por sexo e idade).^614^	7-18	Figura 1	10.1123/pes.22.3.431	https://journals.humankinetics.com/doi/10.1123/pes.22.3.431
**Cardiopata:**				
Gráfico e tabelas por sexo dos percentis de VO_2_max/VO_2_pico e %VO_2_previsto em pacientes com corações univentriculares, tetralogia de Fallot, transposição das grandes artérias e outras cardiopatias.^1031^	6-18	Tabela 1 , Tabela 2 e Figura 2	10.1007/s00431-022-04648-9	https://www.ncbi.nlm.nih.gov/pmc/articles/PMC9829639/
Tabela e gráficos de associação entre VO_2_max e FCmax em crianças e adolescentes com CC.^80^	6-18	Tabela 1 , Figura 2 e Figura 4	10.5935/abc.20170125	https://www.ncbi.nlm.nih.gov/pmc/articles/pmid/28876372/
Tabela e gráficos do VO_2_pico de crianças e adolescentes saudáveis e com CC.^1032^	8-16	Tabela 7 , Figura 3 e Figura 4	10.1007/s004210050612	https://link.springer.com/article/10.1007/s004210050612
Gráfico e equação de predição de valores de DP nas duas primeiras décadas de vida e em comparação com pacientes com coarctação de aorta reparada.^428^	12,6±2,96 e 13,0±3,2 anos	Tabela 2 e Figura 3	10.1080/14779072.2017.1385392	https://www.tandfonline.com/doi/epdf/10.1080/14779072.2017.1385392?needAccess=true
Tabela com comportamento do DP (repouso e pico do esforço) em relação a sobrevida de crianças com insuficiência cardíaca secundária à cardiomiopatia dilatada idiopática.^1033^	8,6±1,9 anos	Tabelas 2 e 3	10.1016/j.ejheart.2008.04.009	https://onlinelibrary.wiley.com/doi/epdf/10.1016/j.ejheart.2008.04.009
Tabela e gráficos do comportamento de OUES por sexo e corrigido pelo peso na população pediátrica aparentemente saudável e em 10 cardiopatias congênitas.^624^	5-18	Tabela 2 , Figura 2 e Figura 3	10.1136/archdischild-2019-317724	https://adc.bmj.com/lookup/pmidlookup?view=long&pmid=32732318
Tabela de valores de referência de OUES/kg por idade na diferenciação entre capacidade funcional preservada ou anormal, em crianças e adolescentes, com e sem CC.^623^	4-21	Tabela 5 e Tabela 6	10.1177/2047487318807977	https://academic.oup.com/eurjpc/article-lookup/doi/10.1177/2047487318807977

VO_2_max: consumo máximo de oxigênio; DP: duplo-produto; DP pico: duplo-produto no pico do esforço; OUES: inclinação da eficiência da captação do oxigênio (OUES: do inglês oxygen uptake efficiency slope); FCmax: frequência cardíaca máxima; CC: cardiopatia congênita ; VO_2_pico: maior VO_2_obtido no teste ergométrico que não houver as características de um esforço máximo.

### Anexo 5 Principais bebidas, alimentos e medicamentos que contêm cafeína

**Table:**

**Cafés**
Café Expresso Café mocha Café descafeinado
**Chás - em geral**
Chá preto Iced Tea Chá verde Lemon Iced Tea garrafa Lipton chá descafeinado (preto ou verde)
**Refrigerantes e sucos**
Pepsi Coca-Cola, Coca Zero ou Diet Pepsi Coca-Cola Plus Diet Coke Fanta, Sprite, 7-Up Guaraná Suco de acerola
**Energéticos – em geral**
Monster Energy Red Bull Fusion TNT
**Petiscos Cafeinados**
Bolacha de chocolate Alguns tipos de batata chips Algumas balas e gomas
**Sorvetes**
Starbucks ice cream café Sorvetes de café Sorvete de café Häagen-Dazs
**Chocolates e bebidas**
Chocolate quente Barra de chocolate Barra de chocolate ao leite
**Medicamentos**
Tylenol DC Ormigrein Dipirona + cafeína Neosaldina Miorrelax Miosan cafeína Dorflex Benegrip Suplementos e pílulas de cafeína

Nota: Os produtos e marcas listados são os mais frequentemente disponíveis no mercado brasileiro. Os mesmos cuidados se aplicam a produtos similares brasileiros. Adaptado de: Henzlova MJ et al. ASNC imaging guidelines for SPECT nuclear cardiology procedures: Stress, protocols, and tracers.^995^
