# Supplementary material for: Diretriz Brasileira de Ergometria em Crianças e Adolescentes – 2024
Source: Arq Bras Cardiol. 2024 Sep 3;121(8):e20240525. [Article in Portuguese] doi: 10.36660/abc.20240525 (PMC11495813; doi:10.36660/abc.20240525)
Supplement: Translation

# Translation: Brazilian Guideline for Exercise Testing in Children and Adolescents – 2024

**DOI:** 10.36660/abc.20240525i

**Authors:** Tales de Carvalho, Odilon Gariglio Alvarenga de Freitas, William Azem Chalela, Carlos Alberto Cordeiro Hossri, Mauricio Milani, Susimeire Buglia, Andréa Maria Gomes Marinho Falcão, Ricardo Vivacqua Cardoso Costa, Luiz Eduardo Fonteles Ritt, Maria Eulália Thebit Pfeiffer, Odwaldo Barbosa e Silva, Rodrigo Imada, José Luiz Barros Pena, Antônio Carlos Avanza, Carlos Alberto Cyrillo Sellera

**Affiliations:** 1 Florianópolis SC Brazil Clínica de Prevenção e Reabilitação Cardiosport, Florianópolis, SC – Brazil; 2 Florianópolis SC Brazil Universidade do Estado de Santa Catarina, Florianópolis, SC – Brazil; 3 Belo Horizonte MG Brazil Minascor Centro Médico, Belo Horizonte, MG – Brazil; 4 São Paulo SP Brazil Instituto do Coração do Hospital das Clínicas da Faculdade de Medicina da Universidade de São Paulo (InCor-HCFMUSP), São Paulo, SP – Brazil; 5 São Paulo SP Brazil Sociedade Beneficente de Senhoras do Hospital Sírio-Libanês, São Paulo, SP – Brazil; 6 São Paulo SP Brazil Hospital do Coração (HCOr), São Paulo, SP – Brazil; 7 São Paulo SP Brazil Instituto Dante Pazzanese de Cardiologia, São Paulo, SP – Brazil; 8 Brasília DF Brazil Universidade de Brasília (UnB), Brasília, DF – Brazil; 9 Hasselt Belgium Hasselt University, Hasselt – Belgium; 10 Hasselt Belgium Jessa Ziekenhuis, Hasselt – Belgium; 11 Rio de Janeiro RJ Brazil Hospital Pró-Cardíaco, Rio de Janeiro, RJ – Brazil; 12 Salvador BA Brazil Escola Bahiana de Medicina e Saúde Pública, Salvador, BA – Brazil; 13 Salvador BA Brazil Instituto D’Or de Pesquisa e Ensino, Salvador, BA – Brazil; 14 Salvador BA Brazil Hospital Cárdio Pulmonar, Salvador, BA – Brazil; 15 Rio de Janeiro RJ Brazil Instituto Estadual de Cardiologia Aloysio de Castro, Rio de Janeiro, RJ – Brazil; 16 São José dos Campos SP Brazil Faculdade de Ciências Médicas de São José dos Campos, São José dos Campos, SP – Brazil; 17 São Paulo SP Brazil Hospital Sírio-Libanês, São Paulo, SP – Brazil; 18 Belo Horizonte MG Brazil Faculdade Ciências Médicas de Minas Gerais, Belo Horizonte, MG – Brazil; 19 Belo Horizonte MG Brazil Hospital Felício Rocho, Belo Horizonte, MG – Brazil; 20 Vila Velha ES Brazil Universidade Vila Velha, Vila Velha, ES – Brazil; 21 Santos SP Brazil Universidade Metropolitana de Santos (UNIMES), Santos, SP – Brazil

## Abstract

**Classes of Recommendation:**

**Class I:** Conditions for which there is conclusive evidence and, failing that, general agreement that a given procedure is safe and useful/effective.
**Class II:** Conditions for which there is conflicting evidence and/or a divergence of opinion about the safety and usefulness/efficacy of a procedure.
**Class IIa:** Weight or evidence/opinion in favor of the procedure. Most approve.
**Class IIb:** Safety and usefulness/efficacy less well established, with divergence of opinions.
**Class III:** Conditions for which there is evidence and/or general agreement that a procedure is not useful/effective and, in some cases, may be harmful.

**Levels of Evidence:**

**Level A:** Data derived from multiple large, concordant randomized trials and/or robust meta-analyses of randomized trials.
**Level B:** Data derived from less robust meta-analyses, from a single randomized trial and/or from observational studies.
**Level C:** Data derived from consensus opinion of experts.

**Table:** 

Brazilian Guideline for Exercise Testing in Children and Adolescents – 2024	
The report below lists declarations of interest as reported to the SBC by the experts during the period of the development of these statement, 2023-2024	
Expert	Type of relationship with industry
Andréa Maria Gomes Marinho Falcão	Nothing to be declared
Antonio Carlos Avanza Júnior	Nothing to be declared
Carlos Alberto Cordeiro Hossri	Nothing to be declared
Carlos Alberto Cyrillo Sellera	Nothing to be declared
José Luiz Barros Pena	Nothing to be declared
Luiz Eduardo Fonteles Ritt	Financial declaration A - Economically relevant payments of any kind made to (i) you, (ii) your spouse/partner or any other person living with you, (iii) any legal person in which any of these is either a direct or indirect controlling owner, business partner, shareholder or participant; any payments received for lectures, lessons, training instruction, compensation, fees paid for participation in advisory boards, investigative boards or other committees, etc. From the Brazilian or international pharmaceutical, orthosis, prosthesis, equipment and implants industry: - Boehringer Lilly: Jardiance; Novonordis: researcher in studies; AstraZeneca; Novartis; Bayer; Bristol; Pfizer. B - Research funding under your direct/personal responsibility (directed to the department or institution) from the Brazilian or international pharmaceutical, orthosis, prosthesis, equipment and implants industry: - MDI Medical. Other relationships Funding of continuing medical education activities, including travel, accommodation and registration in conferences and courses, from the Brazilian or international pharmaceutical, orthosis, prosthesis, equipment and implants industry: - Novo Nordisk: Ozempic.
Maria Eulália Thebit Pfeiffer	Nothing to be declared
Mauricio Milani	Nothing to be declared
Odilon Gariglio Alvarenga de Freitas	Nothing to be declared
Odwaldo Barbosa e Silva	Nothing to be declared
Ricardo Vivacqua Cardoso Costa	Nothing to be declared
Rodrigo Imada	Nothing to be declared
Susimeire Buglia	Nothing to be declared
Tales de Carvalho	Nothing to be declared
William Azem Chalela	Nothing to be declared

## Part 1 - Indications, Legal Aspects, and Training in Exercise Testing

### 1. Introduction

Exercise testing (ET) is a diagnostic modality in which a person is subjected to a planned, individualized degree of physical effort with the purpose of evaluating their clinical, hemodynamic, autonomic, electrocardiographic, indirect metabolic and, occasionally, enzymatic responses to physical exertion. When ET also includes the assessment of ventilatory parameters and analysis of exhaled gases, it is called cardiopulmonary exercise testing (CPET or CPX). The term *cardiac stress test* encompasses both ET and CPET.^1^

In children and adolescents, ET and CPET are useful for diagnostic and prognostic evaluation as well as for assessment of cardiorespiratory performance in a wide range of clinical scenarios. These procedures are considered safe and have proven cost-effective in the pediatric population.^1^

In Brazil, congenital disorders and cardiovascular diseases (CVD) are, respectively, the second and ninth leading causes of death in children. In adolescents, CVD are the third leading cause of death, and congenital disorders, the eighth leading cause. These data highlight the importance of cardiovascular (CV) health care for the pediatric population.^2^

The Brazilian Ministry of Health follows the definition of adolescence proposed by the World Health Organization (WHO), which characterizes it as the period of the life cycle between the ages of 10 and 19 years. However, Brazilian legislation considers all persons from the ages of 12 to 18 to be adolescents. The scientific literature also adopts other age ranges for children (1-13 years) and adolescents (13-18 years).^3 – 5^

This Guideline seeks to consolidate the most recent information from the scientific literature on ET/CPET in children and adolescents, covering indications, contraindications, risks, methodology, hemodynamic and electrocardiographic responses, diagnostic criteria, as well as particular aspects in specific diseases which afflict the pediatric population. Ventilatory and metabolic variables derived from exhaled breath analysis in CPET and ET/CPET combined with imaging methods are also addressed.

Throughout the document, we highlight the particularities of exams depending on the patient's age range, sex, body composition, physical fitness level, and baseline cardiovascular and pulmonary health status.^6 – 12^

This Guideline should become a relevant work of reference for the general cardiologist and, we hope, encourage widespread uptake of pediatric ET/CPET with a view to improving the CV health of children and adolescents.

### 2. Indications and Contraindications for ET and CPET in Children and Adolescents

#### 2.1. General Indications for ET

In the pediatric population, ET is a tool that contributes to the diagnosis and assessment of the impact of CVD (congenital/genetic and acquired), risk stratification, prognostication, optimization of therapy, and medical clearance for physical activity/exercise prescription, including cardiovascular rehabilitation (CVR).

The general indications and purposes of ET in the pediatric population are:^6 – 12^

1. Assessment of exercise-induced symptoms.
2. Assessment of hemodynamic parameters (blood pressure, heart rate, double product, peripheral arterial resistance, etc.).
3. Detection of abnormal responses to exertion in children and adolescents with congenital and acquired cardiovascular diseases (valvular heart disease, cardiomyopathies, etc.), lung disease, or diseases of other organ systems.
4. Detection of myocardial ischemia resulting from congenital anomalies of the coronary arteries, atheromatosis (very rare), or in the context of Kawasaki disease.
5. Recognition of cardiac arrhythmias and elucidation of their type, density, and complexity.
6. Assessment the behavior of pre-excitation and channelopathies during exercise.
7. Establishment of prognosis for certain CVD, including through serial ET/CPET.
8. Indication and optimization of therapy.
9. Assessment of aerobic fitness, exercise tolerance, and physical conditioning.
10. To inform medical clearance for physical activity/exercise prescription, including CVR and participation in sports.

#### 2.2. Specific Clinical Indications for ET

The following section describes specific clinical settings in which ET has its effectiveness studied, tested, and proven, allowing us to determine the class of recommendation and level of evidence established in the literature.

##### 2.2.1. Suspected Myocardial Ischemia and Coronary Artery Disease

In children and adolescents, myocardial ischemia and coronary artery disease (CAD) have different etiologies than in adults. In this setting, ET has recognized utility to support the initial diagnostic workup, follow-up, therapeutic decision-making, and risk stratification ( Table 1 ).^6 , 13 , 14^

**Table 1:** Indications for ET in suspected myocardial ischemia and coronary artery disease in children and adolescents

Indication	Class of recommendation	Level of evidence
In Kawasaki disease, for investigation of exercise-induced arrhythmias, functional assessment, quantification of the impact of coronary lesions, risk stratification, and medical clearance/exercise prescription.^15 – 18^	I	B
Diagnostic investigation of typical (anginal) chest pain.^6 , 13 , 14 , 19 , 20^	I	C
Assessment of residual myocardial ischemia and exercise tolerance after surgical correction of transposition of the great arteries.^21 – 23^	I	C
After coronary artery surgery (arterial switch procedure, Ross procedure, ascending aorta replacement, repair of Bland-White-Garland syndrome), for detection of myocardial ischemia and exercise-induced arrhythmias or medical clearance/exercise prescription (including CVR).^6 , 24 – 27^	IIa	B
In anomalous coronary arteries, for screening, investigation of stress-induced ischemia, indication of surgical repair, and medical clearance/exercise prescription.^28 , 29^	IIa	B
Assessment of functional capacity and therapeutic decision-making for patients in whom myocardial ischemia has been detected by another diagnostic modality.^6 , 30^	IIb	B
In myocardial bridging, to investigate symptoms and exercise-induced arrhythmias and for risk stratification.^31 , 32^	IIb	B
In the follow-up of patients with Takayasu arteritis or systemic lupus erythematosus, to investigate secondary coronary artery disease.^33 , 34^	IIb	C
Diagnosis of CAD in patients with LBBB, WPW, pacemaker rhythm, and under digitalis therapy.	III	B
Investigation of non-anginal typical chest pain.^13 , 35 , 36^	III	B
In Kawasaki disease, to evaluate myocardial ischemia (ET alone).^37^	III	C

CAD: coronary artery disease; LBBB: left bundle branch block; WPW: Wolff-Parkinson-White syndrome; ET: exercise test; CVR: cardiovascular rehabilitation.

##### 2.2.2. Indications for ET in Hypertension

ET allows assessment of the blood-pressure (BP) response to exercise and diagnosis of hypertension (HTN) in pediatric patients with and without CHD. BP behavior during ET has additional predictive power over office measurements ( Table 2 ).^38^

**Table 2:** Indications for ET in children and adolescents with hypertension

Indication	Class of recommendation	Level of evidence
Assessment of BP response to exercise (in patients with or without heart disease) for diagnosis of HTN.^41 – 43^	I	C
Preparticipation physical assessment of patients with known HTN seeking to engage in competitive sports.^44 , 45^	I	B
Suspected white coat hypertension.^41 , 46^	IIa	B
Assessment of treatment response and risk stratification in patients with known HTN.^4 , 47 , 48^	IIa	B
After correction of coarctation of the aorta, for assessment of BP behavior, post-exercise ankle-brachial index * , and stratification of HTN risk.^8 , 49 – 51^	IIa	B
Suspected masked hypertension in adolescents.^41 , 52 , 53^	IIa	C
Assessment of patients with decompensated HTN.^11^	III	C

BP: blood pressure; HTN: hypertension.

* Diagnostic test performed additionally with ET on a treadmill to assessment of the ankle-brachial index at rest and post-exercise.

Pediatric hypertension is associated with increased risk of CVD, atherosclerosis, left ventricular hypertrophy (LVH), and renal failure in adulthood.^39 , 40^

##### 2.2.3. Indications for ET in Asymptomatic Patients

Studies carried out in recent years have elucidated the role of ET in the assessment of asymptomatic pediatric patients, specifically its utility for risk stratification and prognostication ( Table 3 ).

**Table 3:** Indications for ET in asymptomatic children and adolescents

Indication	Class of recommendation	Level of evidence
Screening and monitoring of genetic hyperlipidemias and/or carotid atherosclerosis.^54 – 56^	IIa	B
Family history of sudden unexplained death in young individuals.^57 , 58^	IIa	B
Assessment of cardiorespiratory fitness in asymptomatic patients with cardiometabolic risk factors. * ^59 – 61^	IIa	C
Preparticipation physical assessment of asymptomatic, apparently healthy children and adolescents seeking to engage in leisure exercise and/or recreational sports.^62^	III	C

* See Pre-ET Risk Stratification (see part 2, section 2).

##### 2.2.4. Indications for ET in Athletes

In child and adolescent athletes, ET allows assessment of cardiorespiratory fitness (CRF) and the hemodynamic response to exercise, as well as diagnosis of CVD and their potential implications ( Table 4 ).^8 , 63 , 64^

**Table 4:** Indications for ET in child and adolescent athletes

Indication	Class of recommendation	Level of evidence
Investigation of exercise-related symptoms.^8 , 64^	I	A
Diseases and conditions with high risk of sudden cardiac death.^64 – 66^	I	C
Diagnosis and follow-up of exercise-induced asthma.^67 , 68^	IIa	B
Diagnosis and follow-up of hypertension.^44 , 53 , 69^	IIa	B
In type I diabetes, for symptom investigation, assessment of cardiorespiratory fitness, and risk stratification.^70 – 72^	IIa	B
Diagnosis and follow-up of cardiomyopathies.^73 , 74^	IIa	B
Pre-participation screening of competitive sport, in asymptomatic, without risk factors and apparently healthy.^8^	III	B

##### 2.2.5. Indications for ET in Congenital Heart Disease

The worldwide prevalence of congenital heart disease (CHD) ranges from 2.4 to 13.7 per 1,000 live births, with the majority of patients (85%) reaching adulthood.^75 , 76^Usually, children with CHD – even after repair – are less physically active, including due to family overprotection.^77 , 78^Up to 15-20% of patients with CHD have some valve involvement, with the most common (when occurring in isolation) being aortic (bicuspid, stenotic) and pulmonary.^79 – 81^

ET is recommended for clinical assessment, determination of cardiorespiratory fitness, treatment decision-making, follow-up, risk stratification/prognosis and medical clearance/prescription of exercise programs, including CVR ( Table 5 ).^7 , 9 , 82 – 85^

**Table 5:** Indications for ET in children and adolescents with congenital heart disease

Indication	Class of recommendation	Level of evidence
Assessment of cardiorespiratory fitness and risk stratification/prognosis in acyanotic CHD, before and after corrective surgery.^7 , 10 , 86 , 87^	IIa	B
Assessment of cardiorespiratory fitness and risk stratification/prognosis in cyanotic CHD, after corrective surgery.^7 , 86 , 87^	IIa	B
Assessment of arrhythmia behavior and risk stratification.^82 , 83 , 88^	IIa	B
Prescription and optimization of an exercise program, including a cardiovascular rehabilitation program.^89 , 90^	IIa	B
Assessment of cardiorespiratory fitness and risk stratification/prognosis after Fontan procedure.^84 , 91 , 92^	IIa	B
In compensated HF after interventional treatment, to optimization of therapy, risk stratification/prognosis, and clearance/prescription of cardiopulmonary rehabilitation.^93 , 94^	IIa	B
Assessment of symptoms triggered or worsened by exertion.^95 , 96^	IIa	C
In asymptomatic patients after tetralogy of Fallot repair, to assess the possibility of pulmonary valve replacement.^97 , 98^	IIb	B
Risk stratification/prognosis after tetralogy of Fallot repair.^99 , 100^	IIb	B
Assessment of cardiorespiratory fitness and risk stratification/prognosis after correction of transposition of the great arteries.^101 , 102^	IIb	B
Assessment of cardiorespiratory fitness in Fabry disease.^103 , 104^	IIb	B
Assessment of the degree of desaturation with exertion in clinically stable cyanotic CHD. * ^7^	IIb	B
CHD with decompensated HF.	III	C
Decompensated cyanotic CHD.	III	C

CHD: congenital heart disease; HF: heart failure.

* Additional non-invasive oximetry monitoring performed additionally/concomitantly with the ET is advised.

##### 2.2.6. Indications for ET in Arrhythmias and Conduction Disorders

In the setting of arrhythmias and conduction disorders in children and adolescents, ET is indicated for evaluation of symptoms, diagnosis of arrhythmias, definition of management approaches, risk stratification, and prescription of physical exercise ( Table 6 ).^9 , 11 , 105 – 109^

**Table 6:** Indications for ET in the context of arrhythmias and conduction disorders in children and adolescents

Indication	Class of recommendation	Level of evidence
Palpitations, syncope, pre-syncope, syncope equivalents, undefined malaise, or pallor associated with physical exertion and/or immediately following exertion.^105 , 110 , 111^	I	B
Suspicion of catecholaminergic polymorphic ventricular tachycardia.^112 , 113^	I	B
In congenital heart block, for assessment of ventricular response and subsequent indication of pacemaker placement.^114 , 115^	I	B
In congenital heart block, for selection of pacemaker type according to the atrial rate response.^116 , 117^	I	C
Assessment and diagnosis of sinus node dysfunction secondary to CHD or after surgical correction of CHD.^118 , 119^	IIa	B
Assessment of pharmacotherapy responses and/or ablation for stress-induced arrhythmias.^7 , 120 , 121^	IIa	B
In patients with known and controlled arrhythmia, to medical clearance/exercise prescription (including cardiovascular rehabilitation).^122 , 123^	IIa	B
In long QT syndrome, for diagnostic confirmation, risk stratification, assessment of arrhythmogenic potential, and optimization of therapy.^57 , 109 , 124^	IIa	B
In suspected Brugada syndrome, to aid diagnosis and risk stratification.^125 – 127^	IIa	B
Assessment of heart rate response in patients with a rate-adaptive pacemaker.^7 , 116 , 120 , 128^	IIa	C
Assessment of accessory pathway behavior (pre-excitation) and arrhythmogenic potential.^7 , 120 , 121^	IIb	B
Patients with fixed-rate pacemakers.	III	B
Non-congenital complete heart block.	III	B
Assessment of isolated atrial and/or ventricular ectopic beats in apparently healthy children and adolescents with no comorbidities or complaints.^7 , 120 , 129^	III	C
Uncontrolled arrhythmia, symptomatic or with hemodynamic instability.	III	C

CHD: congenital heart disease.

##### 2.2.7. Indications for ET in Children and Adolescents with Valvular Heart Disease

Valvular heart disease accounts for a significant percentage of heart diseases in the pediatric population, whether congenital or acquired. Rheumatic heart disease (RHD) with subsequent valvular involvement is one of the leading causes of cardiac morbidity and mortality among children in underdeveloped and developing countries. In 2019, approximately 40 million cases of RHD are known worldwide, with 2,789,443 new cases and 305,651 deaths reported annually.^130 , 131^

Valvular heart disease can cause hemodynamic disorders, depending on the severity of valvular and myocardial involvement. Stenotic lesions generally result in pressure overload of the affected chamber, while regurgitant lesions cause volume overload. Many lesions, however, are mixed, resulting in both pressure and volume overload, with the potential for development of heart failure (HF). Valvular heart disease secondary to acquired cardiomyopathies, myocarditis, and HF is also common. Over time, increased stress on the ventricular wall causes myocardial stretching and fibrosis, resulting in formation of scar tissue with arrhythmogenic potential. Arrhythmias can complicate the clinical picture of valvular heart disease and increase morbidity and mortality in affected children and adolescents.^132 – 134^

Table 7 lists the indications for ET in children and adolescents with specific forms of valvular heart disease and their respective classes of recommendation.

**Table 7:** Indications for ET in children and adolescents with valvular heart disease

Indication	Class of recommendation	Level of evidence
In mild/moderate valvular heart disease, for assessment of symptoms, arrhythmias, cardiorespiratory fitness, and medical clearance/exercise prescription.^81 , 135^	I	B
In AS, SVAS, and subaortic stenosis, for assessment of symptoms, risk stratification, and definition of interventional treatment.^81 , 134 , 136^	IIa	B
In compensated, moderate to severe AI, for assessment of symptoms and cardiorespiratory fitness, indication of interventional treatment, and medical clearance/exercise prescription.^137 , 138^	IIa	B
In bicuspid aortic valve, for assessment of the inotropic response and risk stratification.^62 , 139^	IIa	B
After valve repair or replacement, for assessment of symptoms and cardiorespiratory fitness, prognosis, and medical clearance/exercise prescription.^140 , 141^	IIa	B
Symptomatic patients with severe mitral or aortic stenosis, for assessment of cardiorespiratory fitness.	III	B

AI: aortic insufficiency; AS: aortic stenosis; SVAS: supravalvular aortic stenosis.

##### 2.2.8. Indications for ET in Children and Adolescents with Acquired Heart Diseases and Cardiomyopathies

Cardiomyopathies in children include a wide range of conditions that may be primary or secondary to systemic diseases (i.e. neuromuscular disorders, AIDS, COVID-19).^142 – 144^

The estimated annual incidence of cardiomyopathy is 1.1 to 1.5 cases per 100,000 persons aged 0-18 years.^145^These patients may present with progressive systolic and/or diastolic HF. HF affects 0.87 to 7.4 per 100,000 children, and has a 5-year mortality rate of 40%.^146^In these patients (and in those recovering from myocarditis), ET is indicated for clinical monitoring, therapeutic decision-making, and prescription or adaptation of an exercise program ( Table 8 ).^6 , 143 , 147 , 148^

**Table 8:** Indications for ET in children and adolescent with acquired heart disease and cardiomyopathies

Indication	Class of recommendation	Level of evidence
Young patients 6 months after recovery from myocarditis (including viral), for evaluation of exercise-induced arrhythmias.^62 , 149 , 150^	IIa	B
In compensated HF secondary to cardiopathy for prognostic assessment, optimization of therapy, and medical clearance/exercise prescription (including cardiovascular rehabilitation).^151 – 153^	IIa	B
In hypertrophic cardiomyopathy, for assessment of cardiorespiratory fitness and prognostic markers (symptoms, ventricular arrhythmias, inotropic response).^154 – 157^	IIa	B
In compensated cardiomyopathies (i.e. Chagas disease and amyloidosis), for follow-up and optimization of therapy.^158 , 159^	IIb	B
After heart transplantation, for assessment of cardiorespiratory fitness and medical clearance/exercise program (including cardiovascular rehabilitation).^160 , 161^	IIb	B
In acute myocarditis and pericarditis or decompensated HF.	III	B
For diagnosis of HF.	III	C
Selection for heart transplantation (based on estimated, not measured, VO_2_values).	III	C

HF: heart failure; VO_2_: oxygen consumption; ET: exercise testing.

##### 2.2.9. Indications for ET in Other Clinical Scenarios

ET is indicated to assist in diagnosis, assess cardiorespiratory fitness and hemodynamic status, inform therapeutic decision-making, and stratify risk in several other specific diseases and conditions ( Table 9 ).

**Table 9:** Other indications for ET in children and adolescents

Indication	Class of recommendation	Level of evidence
In patients with suspected exercise-induced asthma and exercise-induced laryngeal obstruction, to adapt the exercise prescription.^162 – 164^	IIa	B
At least 6 months after recovery from myocarditis or pericarditis (including due to COVID-19), for preparticipation physical assessment/medical clearance to engage in sports.^150 , 165 , 166^	IIa	B
In sickle-cell anemia, for elucidation of symptoms, assessment of cardiorespiratory fitness, and medical clearance/exercise prescription.^167 , 168^	IIa	C
In primary pulmonary arterial hypertension, for assessment of symptoms, cardiorespiratory fitness and risk stratification/prognosis.^169 , 170^	IIb	B
In patients on dialysis and renal transplant recipients, for exercise prescription and optimization (including cardiovascular rehabilitation programs).^171 – 173^	IIb	B
Risk assessment and prognosis in cancer patients with suspected cardiotoxicity.^104 , 174 , 175^	IIb	B
At least 6 months after recovery from multisystem inflammatory syndrome (including myocarditis and pericarditis) secondary to COVID-19.^150 , 165^	IIb	C

#### 2.3. Indications for CPET in Children and Adolescents

In addition to the information provided by conventional ET, CPET allows evaluation of lung volumes (ergospirometry) and analysis of gases in exhaled air, including direct measurement of oxygen consumption (VO_2_) and carbon dioxide production (VCO_2_).^100 , 176 , 177^Thus, CPET can help elucidate the pathophysiology of unexplained dyspnea, identify specific pathophysiological features of certain diseases, and provide relevant information to inform therapeutic decision-making.^11 , 178^

The general indications for CPET in children and adolescents are:^100 , 176 – 178^

1. All indications for ET described elsewhere in this Guideline, when additional direct quantification of ventilatory and metabolic variables is necessary.
2. Improved assessment of exercise-induced cardiorespiratory signs and/or symptoms (dyspnea, laryngeal stridor, wheezing, etc.).
3. Improved assessment of heart diseases (CHD, valvular heart disease, HF, cardiomyopathies, arrhythmias), lung diseases, and diseases affecting other organ systems (sickle cell anemia, renal failure, neurodegenerative diseases, etc.).
4. Contribution in the indication and follow-up of specific surgical procedures.
5. Assessment of treatment efficacy and optimization of therapy.
6. Assessment of cardiorespiratory fitness when selecting candidates for heart transplantation.
7. Preparticipation physical assessment and follow-up of patients seeking to engage in competitive sports.
8. Prognostic assessment in cardiovascular, pulmonary, and other diseases.
9. Preparticipation physical assessment and follow-up of patients undergoing cardiovascular rehabilitation.

Specific indications for CPET and their respective classes of recommendation and levels of evidence are given in Table 10 .

**Table 10:** Key specific indications for CPET in children and adolescents

Indication	Class of recommendation	Level of evidence
Assessment of cardiorespiratory fitness.^176 , 179^	I	A
Adjustment of aerobic training intensity in competitive athletes.^63 , 149 , 177 , 180 , 181^	I	A
Medical clearance for and prescription of exercises within the context of a cardiovascular rehabilitation program.^141 , 182 – 184^	I	A
Selection of candidates for heart transplantation. * ^185 , 186^	I	A
Assessment of exercise-induced dyspnea or asthma.^178 , 179 , 187 , 188^	I	B
Cyanotic CHD. ** ^81 , 100 , 189^	I	B
Follow-up after heart transplantation. ** ^190 – 192^	I	B
In stable Kawasaki disease and Takayasu arteritis, for assessment of cardiorespiratory fitness and medical clearance/exercise prescription, including cardiovascular rehabilitation.^15 , 17 , 18 , 193^	I	C
Asymptomatic right-to-left shunt. ** ^184 , 194 , 195^	IIa	A
Pulmonary arterial hypertension. ** ^169 , 196 – 198^	IIa	A
Patients with asymptomatic moderate to severe regurgitant valve lesions. ** ^81 , 199 , 200^	IIa	B
Patients with asymptomatic severe aortic stenosis. ** ^81 , 199 , 200^	IIa	B
In cystic fibrosis, for assessment of cardiorespiratory fitness and prognosis.^67 , 201 , 202^	IIa	B
In neuromuscular diseases (multiple sclerosis, Becker and Duchenne muscular dystrophy), for assessment of cardiorespiratory fitness and exercise prescription within the context of rehabilitation.^203 – 206^	IIa	B
Patients with asymptomatic moderate hypertrophic obstructive cardiomyopathy. ** ^207 – 209^	IIa	B
In patients undergoing anticancer therapy, for assessment of suspected cardiotoxicity, risk stratification, and medical clearance/exercise prescription.^104 , 174 , 175 , 210^	IIa	B
Mild to moderate obstructive right heart lesions. ** ^81 , 199 , 211^	IIb	B
After surgical correction of CHD, in asymptomatic, hemodynamically stable patients. ** ^212 , 213^	IIb	B

CHD: congenital heart disease.

* In individuals with the age, body size, ability to understand and adapt/collaborate that are essential for the correct performance of the exam.

** For assessment of cardiorespiratory fitness, therapeutic decision-making, and determination of prognosis.

#### 2.4. Indications for Cardiac Stress Imaging

##### 2.4.1. Myocardial Perfusion Imaging

Myocardial perfusion scintigraphy in the pediatric population has an established role in the assessment of myocardial perfusion/viability and ventricular function. It can be useful in identifying residual or inducible ischemia and ventricular wall motion abnormalities, as well as in risk stratification ( Table 11 ).

**Table 11:** Indications for myocardial perfusion imaging in children and adolescents

Indication	Class of recommendation	Level of evidence
Long-term follow-up of Kawasaki disease with coronary involvement (symptomatic or asymptomatic).^18 , 214 – 217^	I	C
Postoperative follow-up of transposition of the great arteries for investigation of myocardial ischemia, risk stratification, and indication of reintervention.^22 , 214 , 220 – 222^	IIa	B
Long-term follow-up of Kawasaki disease without coronary involvement.^18 , 30 , 214 , 218 , 219^	IIb	B
In hypertrophic cardiomyopathy, for detection of ischemia, risk stratification, and therapeutic management.^214 , 223^	IIb	B
After heart transplantation, for evaluation of graft vascular disease.^214 , 224 , 225^	IIb	C
In tetralogy of Fallot, after Fontan procedure, to identify residual ischemia.^225 , 226^	IIb	C
Identification of myocardial ischemia/fibrosis in patients with anomalous origin of the left coronary artery from the pulmonary artery (ALCAPA).^225 , 227 , 228^	IIb	C

##### 2.4.2. Indications for Stress Echocardiography

In the pediatric population, stress echocardiography is most commonly used to detect ischemia in patients with CAD, Kawasaki disease, or anomalous origin of coronary arteries. Other pediatric indications include: status post heart transplantation; congenital heart diseases (to evaluate hemodynamic and myocardial response); early detection of myocardial dysfunction in specific populations (i.e. patients receiving anthracyclines); and evaluation of the functional response of the right ventricle and pulmonary artery pressure ( Table 12 ).^229 – 233^

**Table 12:** Indications for stress echocardiography in children and adolescents with CVD or symptoms thereof^234 , 235^

Indication	Class of recommendation	Level of evidence
Late follow-up of childhood heart transplant recipients, for detection of coronary insufficiency.^160 , 236 – 238^	IIa	B
Long-term follow-up of Kawasaki disease with coronary involvement.^239 , 240^	IIa	B
In patients who have undergone the Jatene procedure, for abnormal origin and course of the coronary arteries and coronary-cameral fistula.^241 , 242^	IIa	B
Assessment of ventricular function in cardiomyopathies and mitral and aortic regurgitation.^229 , 232 , 243^	IIa	B
In patients with suspected cardiotoxicity from anticancer therapy, for assessment of ventricular function.^233 , 244 , 245^	IIa	B
After heart transplantation, for assessment of ventricular function.^160 , 237 , 238 , 246^	IIa	B
Patients at risk of early atherosclerotic coronary disease (homozygous familial hypercholesterolemia, type 1 diabetes mellitus, etc.).^247 , 248^	IIb	B
In pulmonary atresia with intact ventricular septum or supravalvular aortic stenosis, for detection of coronary insufficiency.^229 , 249^	IIb	B
In hypertrophic cardiomyopathy and pulmonic or aortic stenosis, for assessment of pressure gradient.^229 , 231 , 250 , 251^	IIb	B
Postoperative evaluation of atrial switch procedures for transposition of the great vessels and correction of tetralogy of Fallot.^222 , 252 , 253^	IIb	B

#### 2.5. Relative and Absolute Contraindications for ET and CPET in Children and Adolescents

ET/CPET in the pediatric population is not risk-free; there is potential for complications or adverse events. In some clinical settings, the risk is such that it outweighs the benefit of any information that would be obtained, thus contraindicating ET/CPET. ET in the pediatric population has low morbidity and mortality; the overall incidence of complications ranges from 0.5 to 1.79%.^7 , 11 , 254 , 255^The most common are chest pain (0.69%), dizziness or syncope (0.29%), hypotension (0.35%), and serious arrhythmias (0.46%).^254^In children and adolescents with CHD, the incidence of ventricular tachycardia (VT) has ranged from 1.9 to 7.3%.^256 , 257^

##### 2.5.1. Relative Contraindications for ET and CPET in Children and Adolescents

These are high-risk clinical situations ( Chart 1 ) in which ET/CPET must only be carried out in a hospital setting, with a specialist pediatric emergency physician on standby and additional special precautions. These precautions include: use of modified protocols and target loads; continuous monitoring of oxygen saturation; having personnel and equipment available if urgent pacemaker or implantable cardioverter-defibrillator (ICD) reprogramming is required, etc.^6 – 11^

**Chart 1:** Relative contraindications and special precautions for ET and CPET in children and adolescents^6 – 11^

Hospital setting + Special precautions
Severe asymptomatic valve stenosis *
Severe asymptomatic valve regurgitation *
Complex congenital heart defects (cyanotic or acyanotic)
Moderate to severe pulmonary hypertension
Known inherited arrhythmia syndromes (long QT, catecholaminergic polymorphic ventricular tachycardia and Brugada syndrome)
Suspected complex arrhythmias (tachyarrhythmias and bradyarrhythmias)
Syncope of probable arrhythmogenic etiology or suspected exercise-induced high-grade or third-degree (complete) AV block
Arrhythmogenic right ventricular cardiomyopathy ** ^258 , 259^
Pacemaker or implantable cardioverter-defibrillator (ICD)
Dilated/restrictive cardiomyopathy with HF or arrhythmia (if clinically stable)
Asymptomatic patients with obstructive hypertrophic cardiomyopathy with resting gradient indicating severe disease *
Asymptomatic patients with severe right ventricular or left ventricular outflow tract obstruction *
Stable congestive heart failure (NYHA Class II or III)
CPET for selection of candidates for heart transplantation
Dialytic renal failure
Suspected severe airway obstruction *
SpO_2_>85% at rest, on room air (with supplemental oxygen) *

NYHA: New York Heart Association; HF: heart failure; CPET: cardiopulmonary exercise testing; SpO_2_: arterial oxygen saturation.

* Risk/benefit ratio of CPET must be carefully evaluated.

** In case of suspicion and/or for diagnostic confirmation and further investigation of disappearance of ventricular arrhythmia.

##### 2.5.2. Absolute Contraindications for ET and CPET in Children and Adolescents

Clinical situations presented in Chart 2 are considered absolute contraindications, and ET/CPET should never be performed in children and adolescents.^7 , 9 , 11 , 105 , 181 , 188 , 260^

**Chart 2:** Absolute contraindications for ET and CPET in children and adolescents^7 , 9 , 11 , 105 , 181 , 188 , 260^

Absolute contraindications
Acute febrile or serious illness
Mental or physical disability resulting in inability to adherence to the exercise protocol adequately
Drug intoxication
Recent retinal detachment (during recovery phase)
Uncorrected fluid-electrolyte and metabolic disturbances
Uncontrolled hyperthyroidism
Decompensated diabetes mellitus
Symptomatic patient with severe valve stenosis
Symptomatic patient with severe valve regurgitation
Decompensated congenital heart disease
Decompensated heart failure
Recent myocardial infarction
Acute pulmonary embolism or infarction *
Active rheumatic fever, with or without carditis
Acute myocarditis, pericarditis, or endocarditis
Acute stage of Kawasaki disease
Uncontrolled cardiac arrhythmia
Marfan syndrome with suspected aortic dissection
Aortic or other artery aneurysm requiring intervention
Uncontrolled severe hypertension
Hypertrophic cardiomyopathy with history of syncope and/or complex arrhythmia
End-stage cystic fibrosis
Single-chamber, ventricular, non-rate response pacemaker (VVI pacing mode)
SpO_2_≤85% at rest, on room air (with or without supplemental oxygen)

SpO_2_: arterial oxygen saturation.

* Recent or in chronic phase with major clinical/hemodynamic repercussions.

### 3. Legal Aspects Involved in the Practice of ET and CPET in Children and Adolescents

In addition to the legal and ethical aspects of ET and CPET already presented in the Brazilian Guideline for Exercise Test in the Adult Population, aspects specific to the pediatric population (described below) must also be considered.^1^

#### 3.1. Legal Aspects Involved in the Practice of ET and CPET

ET and CPET are widely accessible, reproducible, noninvasive methods with a low risk of complications in unselected populations. As their performance in Brazil is strictly limited to physicians, they are governed by the Code of Medical Ethics and, therefore, the physician must be aware of their possible ethical and legal implications, duly addressed in the Code of Medical Ethics of the Federal Medical Council (CFM; from portuguese: *Conselho Federal de Medicina* ), Brazilian Civil Code, Consumer Protection Code, and other applicable laws ( Appendix 1 ).^261 – 264^

#### 3.2. Essential Conditions for Performing ET and CPET in Children and Adolescents

Based on the foregoing, some essential conditions must be met when performing ET/CPET in children and adolescents:

1. Exercise testing and cardiopulmonary exercise testing are medical procedures under the exclusive responsibility of a qualified, board-registered physician, who must be fit to practice. The Department of Exercise Testing, Sports, Exercise, Nuclear Cardiology and Cardiovascular Rehabilitation of the Brazilian Society of Cardiology (SBC/DERC) recommends that the physician be board-certified in Cardiology by the Brazilian Medical Association and hold a focused practice designation in Exercise Testing, both duly registered with the Federal Medical Council, as well as additional training in ET/CPET in the pediatric population.
2. Whenever a test with potential risk of complications (including death, however rare) is performed on minors or legally incapacitated patients, one of the parents or legal guardians is advised to remain in the room. The performing physician must recognize adolescents – between 12 and 18 years of age – as having potential legal capacity and provide care to them accordingly, respecting their individuality and maintaining an attitude centered on providing guidance and ensuring the adolescent's participation.
3. Informed consent must be obtained in writing, via an informed consent form (ICF) signed by at least one parent or legal guardian. When the patient is an adolescent, he or she should preferably be included in the decision-making process by obtaining an appropriated informed assent form (IAF). If the patient and/or parent or legal guardian refuses to sign the ICF and/or IAF, the test cannot proceed. In scientific research settings, the ICF and IAF are mandatory. The term "assent" is used to distinguish this process from "consent", which can only be obtained from adults who are legally fully capable of making their own decisions.
4. Testing must be carried out on equipment adapted to the pediatric population, and the testing site must be stocked with all essential supplies (equipment/medicines) needed to provide emergency care to this population, as stated elsewhere in this Guideline.^265 – 268^
5. The physician performing the test must expressly follow all recommendations of public health authorities and medical societies regarding any ongoing endemics, epidemics, and pandemics, as well as the applicable rules and regulations of the patient safety system.^269 – 271^
6. All procedures relevant to ET and CPET described in this guideline must be followed and complied with.
7. ET and CPET should only be performed upon formal medical request.
8. Relative and absolute contraindications to ET/CPET must be assessed.
9. If serious adverse events arise during the test, the physician responsible for the test will provide the necessary support until the attending physician and/or emergency medical services are able to effectively take over or transfer to the emergency department can be completed. If the event is fatal, the physician responsible for the test is advised to notify the Regional Medical Council and request an opinion from its Ethics Committee.
10. After the test, the patient's parent(s) or legal guardian(s) should be instructed to return to the requesting physician for further management. If the patient or his/her parents, legal guardian, or proxy inquires as to the result of the test, the physician performing the test must provide any relevant information.
11. Compensation for the test should include a fair physician's fee and cover all operating costs.
12. ET and/or CPET involves obtaining and processing sensitive patient data, and exercise testing services must therefore respect the Brazilian General Data Protection Law (LGPD; from portuguese: *Lei Geral de Proteção de Dados Pessoais* ) and other relevant legislation and CFM ordinances.^272 – 274^

#### 3.3. Informed Consent and Assent for ET and CPET in Children and Adolescents

Informed consent form (ICF) for ET/CPET and the consenting process itself must follow the guidelines of the Brazilian Code of Medical Ethics and CFM Recommendation No. 1/2016, and must be signed by at least one parent or legal guardian.^275^As noted above, if the patient is an adolescent, obtaining informed assent is also recommended.

### 4. Essential Conditions for Training in Pediatric ET/CPET

ET/CPET in the pediatric population is different from that performed in adults due to the specific prevalence of CVD (including CHD), the need for adjustment of protocols and parameters, and several age-specific aspects involved in interpretation, diagnosis, and prognosis.

It is recommended that cardiologists undergo specific training in pediatric ET/CPET, as follows:^276 , 277^

1. May take place during (concurrently) or after (consecutively) training in Exercise Testing as an area of focused practice (see part 1, section 4 of the Brazilian Guideline for Exercise Testing in the Adult Population – 2024), in an additional and complementary fashion, incorporating the workloads and requirements described below. Such training does not replace training in Exercise Testing as an area of focused practice, does not grant any additional qualifications, and does not constitute a new area of focused practice.^1^
2. Must take place at an educational facility with an active, formally constituted pediatric Exercise Testing service, registered with all relevant public authorities, with regular and up-to-date paperwork (including Department of Health clearance). The facility may be subject to registration, assessment, and accreditation by DERC/SBC.
3. As a mandatory prerequisite for training, candidates must have completed a medical residency in Cardiology or be board-certified in Cardiology and registered as such with the Brazilian Medical Association/CFM, and must either be in training or have completed training toward a focused practice designation in Exercise Testing in accordance with Brazilian Medical Association/CFM regulations.
4. Training should allow the cardiologist to acquire the necessary experience in ET and CPET in the pediatric population (children and adolescents) to be responsible for the performance, interpretation, and organization of pediatric ET/CPET services. Programs shall be theoretical and practical, with a minimum workload of 100 hours.
5. The theoretical portion can be carried out at the facility itself or in partnership with DERC/SBC, and the theoretical syllabus must include, at a minimum, all topics and subjects covered in this Guideline. Furthermore, it is recommended that the theoretical syllabus include the following additional content:
  – A review of CVD in children and adolescents (including CHD), their treatment and workup.
  – A review of medications commonly used in the pediatric population and the necessary dosage adjustments.
  – A review of cardiovascular physiology and exercise physiology both in the healthy pediatric population and in children and adolescents with heart disease (including unrepaired, repaired, and palliatively treated CHD).
6. The practical portion shall correspond to at least 80% of the total program workload and must cover both ET and CPET. It must take place under the direct, on-site supervision of a preceptor, who must be board-certified in Cardiology, hold a focused practice designation in Exercise Testing and have experience in performing ET/CPET in the pediatric population. Practical training must have a minimum ratio of one preceptor to two participants or fewer.
7. Periodic training in emergency care is recommended. This training should correspond to completion of Pediatric Advanced Life Support (PALS) and Advanced Cardiovascular Life Support (ACLS) courses.
8. The training facility shall be responsible for conducting and submitting evaluations of each participant, during and/or at the end of the training program. Transparency in assessments can be ensured by predefining a set of objective criteria that must be met by participants. When candidates fail the program, it is suggested that the training facility provide additional training options to remedy any pending issues, followed by a reassessment. The training facility must provide an official certificate to all approved candidates, as well as a declaration of compliance with all requirements of theoretical and practical training listed herein.
9. After completion of the training program, periodic participation in scientific events/refresher programs in ET and CPET in children and adolescents, at the national and/or international level, is essential for continuous education and improvement of the knowledge acquired during training.

## Part 2 – The Exercise Test

### 1. ET Methodology in Children and Adolescents

#### 1.1. Core Conditions for ET/CPET

##### 1.1.1. Team

ET/CPET must be performed by a qualified, experienced physician, with expertise in exercise testing of children and adolescents and PALS training.

Any other health care providers (registered nurse, nurse technician, or nursing assistant) who are assisting the performing physician must have been specifically trained in the care of children and adolescents, as well as in how to manage emergencies in the pediatric population.^265 – 268^

In patients with complex CHD or at increased risk of complications (see Chart 1 ), it is recommended that ET/CPET be carried out in a hospital setting, with a specialist pediatric emergency physician on standby.

The facility and/or the physician should properly guide and train any other providers potentially involved in the ET/CPET regarding the scheduling of the test, patient education, cleaning of equipment, cleaning of the examination room, and patient care/transport.

##### 1.1.2. Physical Infrastructure

ET/CPET must be performed in a planned, well-lit and well-ventilated environment, large enough to accommodate all ET/CPET equipment (including an examination table or stretcher/gurney, patient chair, and a crash cart) and any additional equipment needed for exercise testing of children and adolescents, while also allowing circulation of at least four people (at least 10 m^2^), at a controlled ambient temperature of 18-22°C and a relative humidity of at least 40%. A parent or legal guardian must be present in the exam room.^264 , 278 – 284^

##### 1.1.3. Equipment

Recommended essential equipment: ergometer; exercise testing system for monitoring electrocardiogram (ECG); printer (or print server access); calibrated sphygmomanometer; stethoscope; wall thermometer and hygrometer; fingertip pulse oximeter; armchairs for patient, chaperone, and physician; examination table or stretcher/gurney; crash cart (if there is only one examination room); oxygen cylinder (next to crash cart) or wall-mounted oxygen port in each ET/CPET room; portable suction device (next to crash cart) or wall-mounted suction in each ET/CPET room; waste receptacles (for common and hospital waste).^149 , 264 , 278 – 280 , 285 , 286^

All equipment must be customized for the pediatric population:

1. Ergometers must be adapted for the age, height, and weight of children/adolescents:
  – Treadmills should incorporate safety side handrails and a height-adjustable front rail to give smaller children somewhere to hold onto. They should also start at a lower speed, consistent with the walking pace of younger children.
  – A padded mat should be placed on the floor immediately behind the treadmill to protect the child.
  – Cycle ergometers must allow adjustment of seat height, handlebar height and position, and pedal strap length, and should exert less braking force, consistent with the pedaling cadence of younger children.
  – For the youngest children, use of a safety harness (consisting of a set of interconnected straps wrapped around the torso and waist and attached to the treadmill or to a secure attachment point) is recommended.
2. For CPET, a special pediatric interface (face mask or mouthpiece) allowing for the necessary adjustments should be used.
3. Age-appropriate cardiac monitoring electrodes should be used. Pediatric electrodes/pads are appropriate for children and smaller adolescents; adult electrodes/pads are fine for taller adolescents and those with a larger chest circumference.
4. A wide-ranging set of blood pressure (BP) cuffs of various sizes should be available for pediatric BP monitoring.^287^
5. The settings of the ergometry system should incorporate specific criteria and parameters for the pediatric population, and should allow magnification of the ECG waveform for adequate visualization.
6. If noninvasive oximetry is performed simultaneously with or in addition to ET/CPET, pediatric sensors should be used.

##### 1.1.4. Emergency Supplies

A pediatric crash cart stocked with basic and advanced life support supplies must be available on site wherever pediatric ET and/or CPET are performed. This guideline recommends that facilities adopt the standardized crash cart composition given in the Brazilian Society of Cardiology Guideline for Cardiopulmonary Resuscitation and Emergency Cardiovascular Care (Chart 17.3 – *Padronização do carro de emergência pediátrica na unidade de internação, terapia intensiva e pronto-socorro* ).^266^

##### 1.1.5. Guidelines for Patients and Parents/Guardians when Scheduling ET/CPET

At the time of scheduling the ET/CPET, patients and their families should be provided guidance (preferably in writing) on the pre-test preparations needed to ensure proper conduct of the test. When very young children are to be tested, parents should be instructed to explain the recommendations to the child as necessary in order to obtain maximum cooperation.

The recommendations are as follows:^7 , 177^

1. The patient must come to the facility well-rested (physical exertion is to be avoided on the day of the test).
2. Avoid fasting or eating to excess before the test; have a light meal 2 hours before. Refrain from drinking any caffeinated beverages (including soft drinks/sodas) on the day of the test.
3. The patient should wear comfortable clothes: shorts, a T-shirt, and appropriate footwear (preferably sneakers/tennis shoes; open-toed shoes and sandals should be avoided). Female teenagers should wear a regular or sports bra.
4. Bring the ET request or order form.
5. Bring the reports of any previous ETs/CPETs.
6. Whether to withhold or continue any medication remains at the discretion of the patient's attending physician.
7. One parent or legal guardian must serve as chaperone for the child/adolescent at all times.
8. In case of CPET, the patient should be advised that they will have to wear a face mask/mouthpiece, but that it will not interfere with their breathing.

Note: adolescents should be screened for alcohol and/or tobacco intake (regardless of the legality of these substances for minors) and, when applicable, advised to discontinue use before the test.

##### 1.1.6. Guidelines for Patients and Parents/Guardians at the Time of ET/CPET

When the child/adolescent and their parents (or legal guardian) arrive for the test, the entire procedure must be explained in language they can both understand. The child and parents must be allowed to ask as many questions as they wish to clarify any potential uncertainty regarding the test.^11^

The team should then show the child/adolescent how to use the ergometer and make it clear that the examination usually does not cause any pain and can even be a fun experience. Parents should be informed that:

– The child/adolescent will be asked to exercise (walk on a treadmill or cycle), within his or her abilities and capacities, and may stop at any time if he or she wishes or needs to stop.
– The doctor and other team members will carry out various procedures needed to monitor the patient and record the test data.
– The child/adolescent may experience symptoms such as fatigue resulting from exercise, as well as other symptoms associated with his or her underlying condition.

##### 1.1.7. Guidance Regarding Medication Intake

Unlike in adults, discontinuation of medications before ET/CPET is rarely indicated in children and adolescents. The pediatric population generally only takes medications that are proven necessary to keep any underlying diseases under control. If discontinuation is deemed necessary, this should be indicated by the child/adolescent's attending physician, taking into account the risks involved. The timing of discontinuation must consider the washout time of each medication and the extent to which this can vary in the pediatric age group.^6 – 12^

In patients with asthma, whether or not to withhold medication depends on the indication for exercise testing.^288^Discontinuation of other medications must consider possible impacts on physical performance, chronotropic response, ischemia and angina thresholds, ST-segment response, exercise-induced arrhythmias, etc.

#### 1.2. Basic ET Procedure

ET/CPET in children is more challenging than in adolescents, especially in those with chronic health conditions. Difficulties in testing children are mainly attributable to three reasons:^289^

1. Very small body size even when the equipment is adapted for the pediatric population.
2. Greatly reduced physical capacity, making adaptation difficult, even with the use of protocols with small increments in effort load.
3. Generally shorter attention span, reduced motivation during prolonged activities and poor cooperativeness, which make it difficult to distinguish limited exercise capacity from lack of cooperation.

##### 1.2.1. Pre-test Phase, Initial Assessment, and Targeted and Specific Physical Examination

It is recommended that the performing physician evaluate the stated indication for the test and the patient's current symptoms, ascertain whether the pre-test recommendations were followed, obtain a detailed history, and perform a targeted physical examination focusing on the cardiovascular and respiratory systems ( Chart 3 ).^264 , 279 , 280^

**Chart 3:** Recommendations regarding targeted history and physical examination in pediatric patients^264 , 279 , 280^

History	Physical Examination
Current symptoms *	General condition (anemia, syndromic facies, pallor, cyanosis)
Past medical/surgical history **	HR/BP *3
Family history of sudden death or early coronary artery disease **	Heart and lung auscultation
Risk factors (see part 2, section 2: Pre-test risk stratification) **	Noninvasive oximetry *4
Current medications **	Peripheral pulses and ankle-brachial index *5
Exercise tolerance *	
Inquire if the patient has undergone ET/CPET before. If so, whether any abnormality was identified **	

ET: exercise test; CPET: cardiopulmonary exercise test; HR: heart rate; BP: blood pressure.

* Ask both the child/adolescent and his/her parents/legal guardian.

** Verified mainly through information given by parents/legal guardian.

*3 Use a cuff suitable for the upper arm circumference.

*4 Additional monitoring. Mainly indicated in cases of cyanotic congenital heart disease, heart failure, valvular heart disease, and post-COVID condition.

*5 Additional monitoring. Indicated as part of the diagnostic workup of coarctation of the aorta, Takotsubo syndrome, and lower-extremity claudication.

It is critical that potential relative and absolute contraindications for ET/CPET be identified. Information on previous treatments must also be obtained (especially in cases of CHD). The use of adult pre-test clinical scores is not recommended, as they are not validated for the pediatric population.

##### 1.2.2. Electrocardiographic Monitoring and Recording System

Continuous ECG monitoring and recording are mandatory at all stages of the ET: rest, stress, and recovery. Ideally, a computerized stress testing system, including software that allows continuous ECG monitoring, data collection, recording, and interpretation specific for the pediatric population, should be used. Hypoallergenic long-term monitoring electrodes with extra tacky (diaphoretic) adhesive are recommended; in young children, special pediatric electrodes should be used.^264 , 279 , 280^

The number of leads to be used (12 or 13) and positioning of the electrode array should follow the Brazilian Guideline for Exercise Testing in the Adult Population – 2024. In 12-lead systems, use the classic Mason-Likar lead system or its modified version (without substitution of the CM5 lead). The 13-lead system is obtained using the classic Mason-Likar lead system by adding the CM5 bipolar lead. Three-lead systems are no longer recommended, given the established superiority of systems with 12, 13, or more leads.^1^

Skin preparation procedures are similar to those for adults, which, in male adolescents, can include shaving to remove excess body hair in areas where electrodes will be placed. In young children, alcohol skin prep should be performed with great care to avoid excess skin abrasion. It is also important to reassure the child that no injection will follow the procedure (many children associate alcohol pads with injections). A mesh vest or shirt can be used to help keep the electrodes and wires firmly in place.

##### 1.2.3. Ergometers

The choice of ergometer should be made on a case-by-case basis, taking into account the child or adolescent's age, height, ability to adapt, safety, and potential physical limitations. There are three main types of ergometer used in ET/CPET: cycle ergometer, treadmill, and upper body ergometer (arm machine or arm cycle). Both the cycle ergometer and treadmill produce adequate, reliable, and reproducible maximum loads, allowing the collection of diagnostic and physical performance information in the pediatric population.^290^

###### 1.2.3.1. Conventional Cycle Ergometer

The conventional lower-limb cycle ergometer (stationary bicycle) is most commonly used in children over 6 years of age. Children who are not used to cycling often experience:

– Early muscle fatigue in the lower limbs, which may not reach maximum effort.
– Difficulty maintaining a pedaling cadence between 40 and 70 rpm.
– Difficulty keeping their feet on the pedals, even when these are appropriately child-sized.

To properly accommodate the child or the adolescent, the cycle ergometer must have adjustable seat height, handlebar height and position, and pedal strap lengths. Children and adolescents with a height of ≥125 cm can perform ET/CPET on a standard adult cycle ergometer or stationary bicycle.^200^Use of a cycle ergometer is preferred when a more accurate assessment of blood pressure is required.

###### 1.2.3.2. Treadmill

ET/CPET on a treadmill is possible in children from 3 years of age, as they are more familiar with walking quickly and even running. However, exercise on a treadmill does not replicate natural walking, and clinicians are advised to first assess the child's capacity to adapt to and coordinate walking on the ergometer. The height of the front handrail should be adjusted as appropriate for the child's height.

In ET of very young or limited children, the following special precautions are suggested:^6 – 12^

– Use of a safety harness to protect the child in the event of sudden collapse or loss of balance.
– Safety side rails and a padded mat placed on the floor at the end of the treadmill to protect the child.
– Having an extra team member positioned immediately behind the treadmill to assist and protect the child as necessary during the test.

Treadmill ET generally yields maximum oxygen consumption (VO_2_max) values ≈10% higher than those obtained on a cycle ergometer.^291 – 293^

###### 1.2.3.3. Upper Body Ergometer

Upper body ergometers are rarely used in children and adolescents. Their use is generally restricted in patients with impaired lower-body mobility caused by lesions of the thoracic or upper lumbar spine, lower-limb amputation, meningocele, spina bifida, etc.^294 , 295^

Nevertheless, ET with an arm ergometer using a validated ramp protocol allows adequate assessment of cardiorespiratory fitness in children and adolescents.^295 , 296^

##### 1.2.4. Choice of Protocol

The choice of protocol should be individualized, taking into account the indication for ET, the patient's level of daily physical activity, and possible physical limitations, aiming at an ideal exercise time of approximately 10 minutes (ranging from 6 to 12 minutes). The protocol must also respect the patient's individual characteristics (age, body size, ability to adapt to incremental load, etc.).^6^

Exercise testing protocols are divided according to the mode of effort exerted:

1. Incremental (gradual increase in load):
  – Step (or stepwise): load is increased in stages (in a stepwise manner, as the name implies) at predetermined time points (every one or more minutes per stage).
  – Ramp: small, frequent load increments (tending to a linear increase) at very short time intervals (increments in seconds, always less than 1 minute).
2. Fixed-load: there is no increase in load at any point during the test. When performed on a treadmill, speed and grade (incline) are simply kept constant.^1 , 12 , 285^

###### 1.2.4.1. Cycle Ergometer Protocols

The main ET protocols for cycle ergometers are listed in Table 13 . The workload performed on a cycle ergometer is generally expressed in watts (W). Most protocols require a pedaling cadence between 50 and 60 rpm, with variation limited to 40-70 rpm.

**Table 13:** Comparison of major cycle ergometer protocols

Protocol	Indications	Initial load	Load increase
Balke	Healthy children and adolescents *	25W	25W/2 minutes
Astrand^299^	Children and adolescents	25W	25W/3 minutes
McMaster * ^300^	Children ** and adolescents ***	12.5W to 25W	5 increment modes, depending on height and sex, at a regular interval of 2 minutes
James * ^301 , 302^	Active children and adolescents *	33W	3 increment modes, depending on BSA, at a regular interval of 3 minutes
Godfrey * ^303 , 304^	Children * and adolescents	10W to 20W	3 increment modes, depending on height, at a regular interval of 1 minute
Rampa^297 , 305^	All populations; ideal for athletes **	10W to 20W	5 to 20W/1 minute. Subdivide increment into equal amounts and increase at regular intervals (<60 seconds) ****

* See Table 14 for a detailed description of each protocol.

** Based on height, only for children aged 6 years and older.

*** Patients with heart, lung, or muscle diseases may require reductions in initial workload.

**** Example: ramp protocol with increment of 15 W/minute = increase load by 5W every 20 seconds.

The Balke and Astrand protocols have the disadvantage of not taking body size into account, and may be too intense for younger children (especially those with heart disease).

Conversely, in the McMaster, James, and Godfrey protocols, initial loads and subsequent increments are dependent on body size (height or body surface area [BSA]) and/or sex ( Table 14 ). In adolescents, their high loads can be a limiting factor for sedentary or very ill patients (i.e. cardiopaths, pneumopaths, etc.).^297 , 298^

**Table 14:** Godfrey, McMaster, and James cycle ergometer protocols^300 – 304^

	Godfrey Increments every: 1 min Pace: 60 rpm			McMaster Increments every: 2 min Pace: 50 rpm					James Increments every: 3 min Pace: 60-70 rpm		
	Height <120 cm *	Height 120-150 cm *	Height >150 cm *	Height ≤119.9 cm *	Height 120-139.9 cm *	Height 140-159.9 cm *	Height ≥160 cm (male)	Height ≥160 cm (female)	BSA <1.0 *	BSA 1.0-1.2 *	BSA >1.2 *
Time (min)	10 W/st	15 W/st	20 W/st	12.5 W/st	25 W/st	50 W/st	50 W/st	25 W/st	16.5 W/st	33 W/st	49.5 W/st
**0**	10 W	15 W	20 W	12.5 W	12.5 W	25 W	25 W	25 W	33 W	33 W	33 W
**1**	10 W	15 W	20 W								
**2**	20 W	30 W	40 W	25 W	37.5 W	50 W	75 W	50 W			
**3**	30 W	45 W	60 W						49.5 W	66 W	82.5 W
**4**	40 W	60 W	80 W	37.5 W	62.5 W	75 W	125 W	75 W			
**5**	50 W	75 W	100 W								
**6**	60 W	90 W	120 W	50 W	87.5 W	100 W	175 W	100 W	66 W	99 W	132 W
**7**	70 W	105 W	140 W								
**8**	80 W	120 W	160 W	62.5 W	112.5 W	125 W	225 W	125 W			
**9**	90 W	135 W	180 W						82.5 W	132 W	181.5 W
**10**	100 W	150 W	200 W	75 W	137.5 W	150 W	275 W	150 W			
**11**	110 W	165 W	220 W								
**12**	120 W	180 W	240 W	87.5 W	162.5 W	175 W	325 W	175 W	99 W	165 W	231 W
**13**	130 W	195 W	260 W								
**14**	140 W	210 W	280 W	100 W	187.5 W	200 W	375 W	200 W			
**15**	150 W	225 W	300 W						115.5 W	198 W	280.5 W
**16**	160 W	240 W	320 W	112.5 W	212.5 W	225 W	425 W	225 W			
**17**	170 W	255 W	340 W								
**18**	180 W	270 W	360 W	125 W	237.5 W	250 W	475 W	250 W	132 W	231 W	330 W
**19**	190 W	285 W	380 W								
**20**	200 W	300 W	400 W	137.5 W	262.5 W	275 W	525 W	275 W			

BSA: body surface area – square meters (m^2^); st: stage; W: watts; min: minute; rpm: revolutions per minute; cm: centimeter.

* For both sexes.

###### 1.2.4.2. Treadmill Protocols

####### 1.2.4.2.1. Step Protocols

######## 1.2.4.2.1.1. Bruce Protocol

The Bruce protocol is most widely used step protocol ( Table 15 ). It is most appropriate for ET in children without severe heart disease and (apparently) healthy children and adolescents, including preschoolers. It can also be used in serial exercise testing to compare data as the child grows. Potential disadvantages:

**Table 15:** Most common graded exercise protocols for the pediatric population and their characteristics^1 , 7 , 306^

Stage	Bruce			Modified Bruce			Ellestad			Balke			Naugthon			Modified Naugthon		
	Min	mph/km/h	%G	Min	mph/km/h	%G	Min	mph/km/h	%G	Min	mph/km/h	%G	Min	mph/km/h	%G	Min	mph/km/h	%G
**01**	3	1.7/2.7	10	3	1.7/2.7	0	3	1.7/2.7	10	1	3.5/5.6	1	2	1.0/1.6	0	2	3.0/4.8	0
**02**	6	2.5/4.0	12	6	1.7/2.7	5	5	3.0/4.8	10	2	3.5/5.6	2	4	2.0/3.2	0	4	3.0/4.8	2.5
**03**	9	3.4/5.5	14	9	1.7/2.7	10	7	4.0/6.4	10	3	3.5/5.6	3	6	2.0/3.2	3.5	6	3.0/4.8	5.0
**04**	12	4.2/6.7	16	12	2.5/4.0	12	10	5.0/8.0	10	4	3.5/5.6	4	8	2.0/3.2	7.0	8	3.0/4.8	7.5
**05**	15	5.0/8.0	18	15	3.4/5.5	14	12	6.0/9.7	15	5	3.5/5.6	5	10	2.0/3.2	10.5	10	3.0/4.8	10
**06**	18	5.5/8.8	20	18	4.2/6.7	16	14	7.0/9.6	15	6	3.5/5.6	6	12	2.0/3.2	14.0	12	3.0/4.8	12.5
**07**	21	6.0/9.7	22	21	5.0/8.0	18	16	8.0/11.2	15	7 to 21	3.5/5.6	+1%/min *	14	2.0/3.2	17.5	14	3.0/4.8	15.0
**08**	24	6.5/10.5	24	24	5.5/8.8	20	18	9.0/12.8	15	22	3.5/5.6	22.0	16	2.0/3.2	21.0	16	3.0/4.8	17.5

Min: minute; mph: miles per hour; km/h: kilometers per hour; %G: treadmill grade/elevation (in %); MET: metabolic equivalent of task.

* Grade/elevation is increased by 1% every minute (speed remains constant).

– In younger or more limited children, the load increments between stages can be very abrupt, which often leads to dropout within the first minute of a new stage.
– Can be too long (to the point of boredom) for active children and adolescents/trained youth athletes.

######## 1.2.4.2.1.2. Modified Bruce Protocol

The modified Bruce protocol, which begins with no slope/grade, is more suitable for younger or more physically limited children. It can be used in children aged 3 years and older with known heart disease or lung disease. The most serious limitation is that it involves abrupt load increments (similar to those of the unmodified Bruce protocol) after the third stage.

######## 1.2.4.2.1.3. Ellestad Protocol

This protocol employs marked increases in speed and is preferably reserved for physically active teenagers and trained athletes. The main limitations of this protocol are that it begins at fairly high speeds, making adaptation difficult for subjects who are not used to running, and that it hinders BP measurement somewhat.

######## 1.2.4.2.1.4. Balke Protocol

Balke protocol incorporates a constant treadmill speed (3.5 mph) with an incremental grade (1% every minute). It is most suitable for obese, very young, chronically ill, or greatly physically limited children.^7 , 306^

One disadvantage is that, in physically active patients, the test duration is extremely long. For these patients, a modified ("running Balke") version of the protocol, which uses a faster constant speed aiming to keep the test duration between 8 and 10 minutes, is preferred.

######## 1.2.4.2.1.5. Naughton Protocol

There are several adaptations of the Naughton protocol for the pediatric population, with variations in initial speed and grade and smaller load increments per stage, allowing better adaptation of younger children and/or those with physical limitations. The Naughton protocol should not be used in healthy children and adolescents, as it prolongs the test unnecessarily.^307^

####### 1.2.4.2.2. Ramp Protocol

Ramp protocols can be fully individualized in terms of speed, grade (initial and final), and duration to meet the needs of each child/adolescent. They allow better determination of maximum oxygen consumption (direct or estimated), ventilatory thresholds (direct), and maximum power (measured or estimated), as well as better assessment of the causes of exercise intolerance, ischemia, and arrhythmias. The target test duration should remain at 8 to 12 minutes, with the slope of the ramp adjusted to the child's size and physical abilities.

For children with heart disease, this guideline suggests starting the protocol at a speed of 1 km/h and 0% grade, with subsequent small, constant increments in intensity.

Table 16 presents an individualization of the ramp protocol based on a study of the Brazilian pediatric population, in which this protocol proved to be more comfortable than the Bruce protocol.^121^

**Table 16:** Individualization of the ramp protocol by sex and age group based on a study of the Brazilian pediatric population

Age range (years)	Females					Males				
	Speed (km/h)		Grade (%)		VO_2_max	Speed (km/h)		Grade (%)		VO_2_max	
	Baseline	Final	Baseline	Final	Mean ± SD	Baseline	Final	Baseline	Final	Mean ± SD
**4-7**	3.0	6.5	4.0	14.0	39.4±4.7	3.5	7.5	5.0	15.0	45.3±9.2
**8-11**	3.5	7.0	5.0	15.0	43.9±6.2	4.0	8.0	5.0	15.0	48.6±7.9
**12-14**	4.0	8.0	5.0	15.0	48.3±7.3	4.0	8.5	6.0	16.0	53.2±9.0
**15-17**	4.0	8.0	5.0	15.0	47.8±10.1	4.5	9.0	6.0	16.0	55.1±9.4

km/h: kilometers per hour; VO_2_max: maximum oxygen consumption; SD: standard deviation. The expected test duration is 10 minutes. Adapted from: Silva OB et al. *Teste ergométrico em crianças e adolescentes: maior tolerância ao esforço com o protocolo em rampa* .^121^

##### 1.2.5. Heart Rate Monitoring

Heart rate (HR) should be monitored and measured directly from the ECG waveform during all phases of ET/CPET. HR should be recorded (at the very least): before exercise; at the end of each stage of a step incremental protocol or every 2 minutes with a ramp incremental protocol; and during recovery (at 1, 2, 4, and 6 minutes). Measurements should be continued for as long as is necessary during the recovery period.

Performing a conventional 12-lead ECG before the ET/CPET is also advised. Conventional ECG is a supplemental test for assessment of the patient's cardiac condition, and can even help uncover potential contraindications to ET/CPET. A conventional 12-lead ECG is considered a medical procedure, and as such is covered in the Brazilian Hierarchical Classification of Medical Procedures (code 4.01.01.01-0).^1 , 278 , 308^

In ET, HR is conceptualized as follows:^95 , 264^

– Maximal heart rate (HRmax): that reached at the point of exhaustion.
– Peak heart rate (HRpeak): the highest HR observed at peak exertion, even if the subject has not reached the point of exhaustion.

It is important to emphasize that, in apparently healthy children, HRmax remains essentially unchanged throughout childhood, and the use of regression equations to estimate HRmax in the pediatric population is limited (less accurate prediction, average dispersion 5-10 bpm). In adolescence, around the age of 16, HRmax begins to decline at a rate of 0.7 or 0.8 bpm per year of advancing age.^177^

It is therefore suggested that an average predicted HRmax value of 197 bpm and a predicted submaximal HR of 180 bpm (which corresponds to -2 standard deviations) be adopted for the entire pediatric age group (children and adolescents).^309 , 310^

If, nevertheless, equations are used to estimate HRmax in the pediatric population, the following factors should be taken into account:^309 , 311^

– The Karvonen equation (HRmax = 220 – age) generally overestimates HRmax.^312 , 313^
– The Tanaka equation (HRmax = 208 – [0.7 x age]) may underestimate or overestimate HRmax, but is considered the most precise equation.^311 , 314 , 315^
– The Nikolaidis equation (HRmax = 223 – [1.44 x age]), which was developed for adolescent athletes, has proven inadequate.^315 , 316^

##### 1.2.6. Blood Pressure Monitoring

Blood pressure (BP) measurement must be performed during all stages of the ET (pre-test, exercise, and recovery) by a duly trained physician experienced in caring for pediatric patients.

Manual measurement performed with an aneroid sphygmomanometer is still the most common. Semiautomated and/or automated devices are available, but may not provide accurate measurements under certain circumstances, due to:^317 , 318^

– Excess movement and vibration (especially in younger children).
– Some devices work by measuring mean BP and calculating systolic and diastolic BP algorithmically. In younger children, this method may present limitations in the assessment of diastolic BP (DBP), as it fails to distinguish between Korotkoff sounds phases IV and V.^319 , 320^
– Most automated equipment has not been validated in the pediatric population for measurements at rest and during intense exertion.^4^

Regardless of the measurement method adopted, use a Velcro® cuff of the appropriate size for the patient's arm circumference. The cuff width should be at least 40% of the upper-arm circumference and cover 80 to 100% of the arm length.^4 . 321^We advise use of the cuff dimensions recommended in the "Brazilian Guidelines for In-Office and Out-of-Office Blood Pressure Measurement – 2023" and "Brazilian Guidelines of Hypertension – 2020" ( Table 17 ).^319 , 322^

**Table 17:** Cuff dimensions according to upper-arm circumference

Upper-arm circumference	Cuff width	Bladder length
≤6 cm	3 cm	6 cm
6-15 cm	5 cm	15 cm
16-21 cm	8 cm	21 cm
22-26 cm	10 cm	24 cm
27-34 cm	13 cm	30 cm
35-44 cm	16 cm	38 cm

Adapted from: Feitosa ADM et al. Brazilian Guidelines for In-office and Out-of-office Blood Pressure Measurement – 2023.^319^

The sphygmomanometer and cuffs must be cleaned and inspected regularly to prevent technical issues that might affect the quality and accuracy of measurements.^323^

When measuring manually, listen for Korotkoff sounds while bearing in mind that:

– SBP corresponds to the reappearance of blood flow (Korotkoff phase I).
– DBP corresponds to the point at which sounds become muffled (Korotkoff phase IV). Phase IV is used instead of phase V (the point at which sounds disappear) because in children, most of the time, Korotkoff sounds can be heard all the way down to 0 mmHg.

Pre-test BP measurements should preferably be obtained:

– At rest, in the seated position, with the arm supported at heart level.
– In whichever position the child/adolescent will perform the exercise.

HR should be measured at the end of each stage of a step incremental protocol or every 2 minutes with a ramp incremental protocol, as well as during recovery (at 1, 2, 4, and 6 minutes). Measurements should be repeated for as long as is necessary during the recovery period. BP should be reassessed whenever there are any discrepancies or a measurement is deemed unreliable or otherwise questionable.

Arm BP measurement is contraindicated in case of arteriovenous fistula, history of lymph node dissection, thrombosis, lymphedema, and/or coarctation of the aorta.

### 2. Pretest Risk Stratification

Studies and guidelines have provided new evidence regarding cardiovascular risk factors in childhood, their relationship with atherosclerosis and premature CVD, and disease-specific risk scores (i.e. body mass index,^324^Kawasaki disease,^37^systemic lupus erythematosus^325^). CV risk in children and adolescents can also be described in relation to the magnitude of the risk of atheriosclerotic disease in the overall population.^59 , 61 , 326^

Pretest CVD risk stratification in the pediatric population based on the presence of underlying diseases is recommended ( Table 18 ).^59^Disease-specific indices and scores should be used when deemed relevant.

**Table 18:** Risk stratification of the pediatric population based on the presence of underlying diseases

Category	Condition
**High risk**	Homozygous FH, T1DM, T2DM, end-stage renal disease, Kawasaki disease with persistent aneurysms, allograft vasculopathy, childhood cancer survivors (hematopoietic cell recipient recipients).
**Moderate risk**	Severe obesity, heterozygous FH, known hypertension, coarctation, high Lp(a), predialytic CKD, AS, childhood cancer survivors (history of radiation to chest).
**Low risk**	Obesity, insulin resistance with comorbidities (dyslipidemia, NAFLD, PCOS), white coat hypertension, HCM and other cardiomyopathies, pulmonary artery hypertension, chronic inflammatory conditions (JRA, SLE, IBD, HIV/AIDS), status post repair of anomalous origin of coronary artery or transposition of the great vessels, childhood cancer survivors (history of cardiotoxic chemotherapy only), Kawasaki disease with regressed aneurysms (zMax ≥5).

FH: familial hypercholesterolemia; T1DM: type 1 diabetes mellitus; T2DM: type 2 diabetes mellitus; Lp(a): lipoprotein (a); CKD: chronic kidney disease; AS: aortic stenosis; NAFLD: non-alcoholic fatty liver disease; PCOS: polycystic ovary syndrome; HCM: hypertrophic cardiomyopathy; JRA: juvenile rheumatoid arthritis; SLE: systemic lupus erythematosus; IBD: inflammatory bowel disease; HIV/AIDS: human immunodeficiency virus/acquired immunodeficiency syndrome; zMax: maximum Z-score at any time during the course of the disease. Adapted from: de Ferranti et al. Cardiovascular Risk Reduction in High-Risk Pediatric Patients: A Scientific Statement From the American Heart Association.^59^

In adolescents, in addition to risk stratification by underlying disease, checking for traditional cardiovascular risk factors is also advised: lipid profile; smoking; family history of early CAD in first-degree relatives (men aged ≤55 years; women aged ≤65 years); blood pressure; body mass index (BMI); fasting blood glucose; and history of physical activity.^59^

Studies have highlighted the relevance of several cardiometabolic risk factors in the pediatric population: SBP, DBP, waist circumference, BMI, sum of four skinfolds, triglycerides (TG), total cholesterol (TC), high-density lipoprotein cholesterol (HDL-C), low-density lipoprotein cholesterol (LDL-C), TC/HDL-C ratio, blood glucose, insulinemia, homeostatic model assessment of insulin resistance score (HOMA-IR), and cardiorespiratory fitness (mL/kg/min, estimated or measured).^60^Determining cardiorespiratory fitness increases the precision of risk stratification, and is recommended especially when other risk factors are present.^327 – 329^

Most CHD are associated with an increased risk of early CVD (from childhood to young adulthood) (see Table 19 ). Children and adolescents with these conditions are at greater risk of complications during ET/CPET.^59 , 82 , 83 , 88 , 119 , 330 – 333^

**Table 19:** Risk of developing cardiovascular disease associated with congenital heart diseases

CHD	Coronary artery disease	Cerebrovascular disease	Peripheral vascular disease	Cardiac arrhythmia
**ASD/VSD (repaired)**	Unknown if there is increased risk.	Increased risk if there is residual shunt.	Unknown if there is increased risk.	Increased risk of junctional tachycardia and ventricular arrhythmia.
**Bicuspid aortic valve**	Potential risk after Ross procedure with coronary artery reimplantation.	Unknown if there is increased risk.	Increased risk related to aortic aneurysm.	Potential risk of ventricular arrhythmia.
**Coarctation of the aorta**	Increased risk related to accelerated atherosclerosis and late hypertension.	Increased risk related to residual hypertension or intracranial aneurysms.	Increased risk associated with residual coarctation or aortic aneurysm.	Risk of malignant arrhythmias and sudden death at 10-year follow-up.
**Ebstein anomaly**	Unknown if there is increased risk.	Increased risk if there is interatrial shunt.	Unknown if there is increased risk.	Increased risk of atrioventricular reentry tachycardia.
**Tetralogy of Fallot**	Increased risk may be related to coronary anomalies.	Increased risk if there is residual intracardial shunt.	Increased risk related to aortic root dilation.	Increased risk of atrial tachyarrhythmias, junctional tachycardia, and ventricular arrhythmias, which can develop decades after surgery.
**TGA**	Increased risk related to reduced coronary flow reserve, proximal intimal thickening, and coronary anomalies.	After atrial switch, increased risk if there is residual leakage from the repair.	After atrial switch, increased risk may be related to previous catheterizations. After arterial switch, increased risk related to neoaortic root dilation.	Risk of malignant arrhythmias and sudden death Increased risk of ventricular arrhythmia and sudden cardiac death in adult patients with corrected TGA.
**Fontan**	Increased risk related to coronary anomalies.	Increased risk if there is Fontan fenestration.	Increased risk related to Fontan venous pressures and previous catheterizations.	Increased risk of atrial flutter in the first 30 days after surgery In the late postoperative period, atrial tachycardias (flutter and fibrillation, intra-atrial reentrant tachycardia) are common due to a reentry mechanism Increased risk of ventricular arrhythmia.
**Cyanotic CHD**	Reduced potential risk.	Increased risk related to secondary erythrocytosis and hyperviscosity syndrome.	Increased risk related to secondary erythrocytosis and hyperviscosity syndrome.	Increased risk of prolonged QTc and ventricular arrhythmia.
**Eisenmenger syndrome**	Reduced potential risk.	Increased risk related to secondary erythrocytosis and hyperviscosity syndrome.	Increased risk related to secondary erythrocytosis and hyperviscosity syndrome.	Increased risk of arrhythmias and sudden death.

ASD: atrial septal defect; VSD: ventricular septal defect; QTc: corrected QT Interval; CHD: congenital heart disease; TGA: transposition of the great arteries. Adapted from: Ferranti et al. Cardiovascular Risk Reduction in High-Risk Pediatric Patients: A Scientific Statement from the American Heart Association.^59^

### 3. Clinical, Hemodynamic, and Electrocardiographic Responses to Exercise

#### 3.1. Clinical Responses

##### 3.1.1. Exercise Tolerance

Determination of exercise tolerance allows the intensity of physical exertion and resulting symptoms (fatigue, dyspnea, lower limb fatigue, etc.) to be quantified. Exercise tolerance can be quantified objectively in any age group by the power generated in watts, by the duration of exercise or by the metabolic equivalent of task (MET) achieved. Compared to adults, children tolerate short-duration exercise better and are less susceptible to fatigue during dynamic exercises.^334^

Exercise tolerance can be quantified subjectively using scales of perceived exertion, such as the Borg scale, modified Borg scale, Pictorial Children's Effort Rating Table (P-CERT), or OMNI scale.^335 , 336^All such scales have limitations related to the degree of cognitive development of children and adolescents:^337 , 338^

– Children aged 0 to 3 years cannot adequately assess their perceived exertion, even during activities of daily living.
– From ages 4 through 7, children become progressively able to evaluate peripheral sensory changes resulting from exercise, but quantification of perceived exertion remains inaccurate.^339^
– From ages 8 through 12, children are able to estimate the intensity of exertion and distinguish the origin of sensory changes relative to different parts of their bodies. The type of exercise and the scale of perceived exertion used can influence the reported response, especially in intense exercise.^337 , 340 – 342^
– During adolescence, perceived exertion is a useful measure, but its relationship with actual achieved HR is less pronounced than in adults.^341 , 343 , 344^

The P-CERT was designed to evaluate perceived exertion in children aged 6 to 9 years, using a perceptual scale containing both text and illustrative pictures, to improve the correlation with achieved HR; however, it is of limited utility in children who cannot read.^345 – 347^

The OMNI Picture System of Perceived Exertion uses illustrations of children, of both sexes, performing various physical exercises (walking, cycling, climbing stairs, swimming, etc.) at various intensities, which facilitates understanding and cooperation by the child.^348 – 350^

##### 3.1.2. Cardiorespiratory Fitness/Functional Capacity

The assessment of cardiorespiratory fitness (CRF)/functional capacity in children and adolescents is an important clinical tool for quantification of symptoms, prognostic assessment, and evaluation of treatment response. It can also quantify cardiovascular and pulmonary dysfunctions and their repercussions in children with congenital or acquired heart disease.^80^

CRF can be assessed:

– Indirectly in ET, through estimated VO_2_max (expressed in METs) and its respective percentage in relation to the predicted value for age.
– Directly in CPET, through measured VO_2_and its respective percentage in relation to the predicted value for age.^351^

Healthy children exhibit cardiorespiratory and metabolic responses different from those observed in adults. Normally, during maximum exertion, they exhibit a higher chronotropic response, lower inotropic response, and lower cardiovascular and ventilatory efficiency. However, children have greater metabolic efficiency and similar levels of exercise capacity compared to adults.^352 , 353^

CRF is influenced by age, sex, level of daily physical activity, obesity, presence of heart and lung diseases, current treatments, etc.^80 , 91 , 354^

Children with congenital or acquired heart disease often experience reduced CRF, regardless of their status (preoperative, postoperative, or long-term follow-up). This impairment may be associated with primary heart disease, treatments for said heart disease, reduced activity/sedentary lifestyle, and behavioral factors (such as overprotection by parents). Adolescents with CHD may have misconceptions about safe and desirable levels of physical activity, which perpetuates the vicious cycle of sedentariness.^355 – 357^

Figure 1 presents pediatric diseases, pathophysiological factors and clinical situations (i.e. comorbidities, treatments, etc.) that compromise the specific components of the Fick equation used to determine CRF (VO_2_max).^355^

**Figure 1:** Pediatric diseases that affect specific components of the Fick equation and thus compromise cardiorespiratory fitness. SVmax: maximum stroke volume during exercise; HRmax: maximum heart rate; (CaO_2_-CvO_2_)max: arteriovenous oxygen difference; CaO_2_: arterial oxygen content; CvO_2_: venous oxygen content; HF: heart failure; LV: left ventricular; CHD: congenital heart disease; AVB: atrioventricular block. Adapted from: Bar-Or O. Pathophysiological Factors Which Limit the Exercise Capacity of the Sick Child.^355^

##### 3.1.3. Symptoms, Inspection, and Auscultation

Clinical observation of symptoms, visual inspection, and physical examination during ET/CPET are essential in children and adolescents ( Chart 3 ) for the following reasons:

– Younger children have limited ability to perceive exertion and interpret peripheral sensory changes.^337 , 338 , 358^
– A complaint of fatigue is the main reason for cessation of exercise in the pediatric population, but correlation with physical findings (breathing pattern, accessory muscle use, dyspnea, etc.) is necessary, not least to determine exercise tolerance and functional class.
– The development of exercise-induced chest pain requires detailed assessment and characterization to assist in the differential diagnosis of possible noncardiac origins (i.e. exercise-induced asthma). Typical chest pain is generally associated with anomalous origin of coronary arteries, aortic stenosis, and Kawasaki disease.^15 , 35 , 37 , 359 , 360^
– Before the test, especially in children with CHD and valvular heart disease, the femoral and peripheral pulses must be palpated to identify changes in amplitude, radiofemoral delay, and possible obstructions.^361 , 362^
– Cardiac auscultation performed immediately after peak exertion will allow assessment of new heart murmurs or changes in existing murmur patterns as compared to pre-test auscultation. Children and adolescents often have an audible third heart sound on baseline auscultation; if one appears during exercise, is generally considered a physiological adaptation which does not correlate with structural heart disease.^363 – 365^Conversely, the occurrence of a systolic murmur and/or split S3 are generally associated with CHD and valvular heart disease.^362 , 366^
– On auscultation of the respiratory system, rhonchi and lung wheezing may indicate exercise-induced bronchospasm associated with asthma.^367^Auscultation of inspiratory stridor and/or wheezing in the upper chest and trachea can aid in the diagnosis of exercise-induced laryngeal obstruction. In these cases, visualization of the laryngeal structures through laryngoscopy is recommended, as it contributes to diagnosis of the type of laryngeal obstruction and management of the obstructive crisis.^368 , 369^

Aspects of symptomatology, visual inspection, and auscultation during ET and CPET which are specific to the pediatric population:^177 , 361 , 362 , 370^

– Sedentary children and adolescents may present a disproportionate increase in respiratory frequency (RF) in relation to the intensity of exertion and dyspnea. Physical examination is usually unremarkable, with no signs of restrictive or obstructive causes of dyspnea.^286 , 371^
– Children with chest wall abnormalities (i.e. scoliosis, *pectus excavatum* , *pectus carinatum* ) may present with exercise-induced dyspnea and, depending on the severity of the deformity, a restrictive process.^372 – 375^
– In muscular dystrophy and other myopathies, dyspnea and low exercise tolerance associated with restrictive lung disease and respiratory muscle impairment are common.^203 – 206^
– Children with hypertrophic obstructive cardiomyopathy may experience exercise-induced chest pain associated with myocardial ischemia. Generally, during pre-test cardiac auscultation, a more perceptible heart murmur is heard in the standing position or after a Valsalva maneuver.^13^
– Children with pulmonary artery hypertension (PAH) may present with exercise-induced chest pain, which is the most common initial symptom of idiopathic PAH.^196 , 376 , 377^
– In dilated cardiomyopathies, chest pain may occur, generally associated with intense fatigue on exertion. Patients must also be watched closely for possible desaturation and cyanosis.^378 , 379^
– Severe pulmonic stenosis can cause crushing chest pain associated with myocardial ischemia.^87 , 380 , 381^
– Aortic, supra-aortic, and subaortic valve stenosis can cause exercise-induced chest pain, dizziness, and fatigue. These children usually have a harsh ejection murmur, sometimes accompanied by an ejection click, from a bicuspid aortic valve.^134 , 382 , 383^
– In children, supraventricular and ventricular tachycardias generally present as palpitations (which can be exacerbated by exertion), but may also present as brief, sharp chest pain.^361 , 370 , 384^

#### 3.2. Hemodynamic Responses

##### 3.2.1. Heart Rate

###### 3.2.1.1. Resting Heart Rate

Resting HR, at baseline, decreases with increasing age and varies from an average of 85 bpm at 4 years of age to 60 bpm at 16 years of age. This reduction in HR is directly related to the decline in metabolic rate as the child ages.^385 – 387^Resting HR values (minimum and maximum) should be correlated with those predicted for pediatric age groups.

In the pediatric population, resting bradycardia is often seen in highly trained athletes, secondary to medications (particularly beta-blockers), hypothyroidism, and sinus node dysfunction.^370 , 388 , 389^Resting sinus tachycardia usually occurs as a result of hot weather conditions, hyperthyroidism, anemia, obesity, pre-test anxiety, and inappropriate sinus tachycardia, rarely associated with supraventricular tachyarrhythmia.^390 – 393^

In children with dilated cardiomyopathy, a higher resting HR is associated with risk of death and need for heart transplantation. Pharmacological control of HR has been associated with improvement in ventricular function and disease progression.^394 – 396^

###### 3.2.1.2. Chronotropic Response

Assessment of the chronotropic response is essential during exercise and in the recovery phase. In children and adolescents, during an incremental ET, HR increases linearly and proportionally to VO_2_, from baseline levels to peak HR. HRmax is generally not affected by cardiorespiratory fitness level or sex, remaining constant throughout childhood and adolescence. However, in serial ET, as the child grows, a reduction in submaximal HR is observed for a same given workload.^8 , 177 , 397 , 398^

During recovery, there is normally a progressive decline in HR with a return to baseline by the sixth minute. In the 1^st^minute of recovery, apparently healthy adolescents show a reduction of ≈44 bpm (males) and ≈36 bpm (females). Boys also tend to have a greater reduction in HR in the 1^st^minute than girls.^302 , 352 , 399^Children who are overweight and/or have less exercise tolerance generally exhibit a slower HR recovery in the 1^st^minute.^400 , 401^

Patients with sinus node dysfunction (SND), or after CHD surgery, may not adequately increase their HR with exercise and may exhibit a lower peak HR. An slow increase in HR as work intensity increases is typically observed in young trained athletes. A blunted or depressed chronotropic response in the pediatric population generally occurs secondary to high vagal tone, sinus node dysfunction, status post CHD surgery, and certain medications (i.e. beta-blockers, calcium channel blockers, and antiarrhythmics).^177 , 389 , 400^

Table 20 presents definitions referring to HR behavior during ET/CPET in the pediatric population, as well as the respective criteria and possible interpretations.

**Table 20:** Definitions, criteria, and interpretation of the HR response to ET/CPET in children and adolescents

Term		Criteria	Interpretation
**HR behavior on resting ECG**			
	Normal HR behavior	HR ranging from minimum to maximum HR predicted for the corresponding age group ( Table 24 ) on resting ECG.	Children and adolescents in sinus rhythm.
	Sinus bradycardia at rest	HR below the minimum expected for the corresponding age group ( Table 24 ) on resting ECG.	Common in adolescent athletes and asymptomatic young adults with increased vagal tone. If secondary to beta-blockers or antiarrhythmics, mention in report. In patients not on negative inotropic medications, evaluate the possibility of sinus node dysfunction or other secondary causes (i.e. hypothyroidism). Rule out second-degree and high-grade AV block.
	Sinus tachycardia at rest	HR above the maximum expected for the corresponding age group ( Table 24 ) on resting ECG.	Usually found in obese patients, those with severe anxiety, hyperthyroidism, anemia, or after excess caffeine or alcohol intake.
**HR behavior on exertion**			
	Normal chronotropic response	Predicted submaximal HR of 180 bpm (which corresponds to -2 standard deviations) reached between 8 and 12 minutes of exercise. * or If, nevertheless, equations are used to estimate HRmax (by Tanaka equation or Karvonen equation), reach ≥80% of the estimated HRmax between 8 and 12 minutes of effort. **	The predicted mean values for the entire pediatric age group (children and adolescents) of maximum HR is 197 bpm and submaximal HR is 180 bpm, they are constant, not being affected by the level of cardiorespiratory fitness, sex and age.^8 , 177 , 397 , 398 , 402 , 403^
	HR drop during exercise	HR decline with progression of exercise, associated with signs and symptoms suggestive of low cardiac output (extreme fatigue, dizziness, drop in SBP, etc.).	This is a test cessation criterion.^7 , 11^
	Impaired chronotropic response or chronotropic incompetence ***	HRmax <175 bpm (treadmill ET) or <170 bpm (cycle ergometer ET), ** or Failure to achieve 80% of predicted HR for age,^404^or <2.5^th^percentile of chronotropic index for age and sex,^405^or Chronotropic index <0,80.	Relatively common in children after surgical correction of CHD. Associated with reduced exercise tolerance, worse quality of life, and higher morbidity in patients with heart disease.^177 , 405 – 409^If occurring after Fontan procedure, it is associated with sinus node dysfunction.^401^
	HR plateau during exercise	HR unchanged across 1 to 2 stages of ET (asymptomatic), subsequently increasing as exercise progresses.	May occur in apparently healthy children; has no clinical significance.
**HR response during recovery**			
	Normal HR response to recovery	During recovery, there is normally a progressive drop in HR with a return to the baseline pattern by the 6^th^minute.	In sinus rhythm. Boys also tend to have a greater reduction in HR in the 1^st^minute than girls.^302 , 352 , 399^
	Slow HR recovery post-exercise	Defined as ΔHR 1^st^min = HRmax during exercise – HR in the 1^st^minute of recovery. There is no consensus value of ΔHR; ≤35 bpm is generally considered abnormal.^302 , 352^	Common after Fontan surgery.^410^In children with CHD, may be associated with chronotropic incompetence. Can be explained by slow reactivation of vagal activity, late withdrawal of sympathetic activity, and/or poor cardiovascular fitness.^410 – 415^
	Sudden, sharp drop in HR during recovery	Generally asymptomatic. There is no consensus reference value, but generally corresponds to a >55 bpm drop in the 1^st^minute of recovery.	Common finding in younger children and pediatric athlete.^416 , 417^

ECG: electrocardiogram; ET: exercise test; HR: heart rate; HRmax: maximal heart rate; bpm: beats per minute; SBP: systolic blood pressure; AV: atrioventricular; CHD: congenital heart disease.

* Submaximal HR predicted for the entire pediatric age range (children and adolescents).

** Maximal HR values may present significant individual variation between 5 and 10 bpm.

*** Describe the use of medications that may affect HR response.

##### 3.2.2. Blood Pressure Response

The blood pressure response is an important ET/CPET variable in the pediatric population, as it reflects the adaptations of cardiac output and peripheral vascular resistance to exertion.^418 , 419^

For pre-test (resting) BP measurement, this guideline recommends adoption of the BP criteria given in Table 21 , based on the Brazilian Guidelines of Hypertension, which take into account the age, sex, and height of children and adolescents (see Appendices 2 and 3 ).^322^

**Table 21:** Definitions of resting blood pressure for ET/CPET according to age group^322^

Children aged 1 to <13 years	Children aged ≥ 13 years
**Normal resting BP:** <P90 for age, sex, and height	**Normal resting BP:** <120/<80 mmHg
**Elevated resting BP:** - SBP ≥P90 and/or DBP ≥P95 for age, sex, and height	**Elevated resting BP:** - BP ≥120/≥80 mmHg

BP: blood pressure; P: percentile; SBP: systolic blood pressure; DBP: diastolic blood pressure. See Appendices 2 and 3 for percentiles.

The normal BP response to exertion entails a progressive increase in systolic blood pressure (SBP), which contributes to the increase in cardiac output, the magnitude of which is directly related to the intensity of exercise. The SBP values reached at peak exertion (SBPpeak), even if not associated with physical exhaustion (maximum effort), are also proportional to age (the older the age, the higher the SBPpeak), body surface area (the larger the area, higher is the SBPpeak), and SBP in the pre-test phase (resting). Maximum SBP (SBPmax) is considered the SBP measured at maximum effort. Occasionally, apparently healthy pediatric patients may present only a slight increase in SBP with exertion.^403 , 420^

Body surface area (BSA) has been used as a criterion for defining percentiles of normality and evaluating SBP response to exercise. For example, children of the same sex and age with different BSAs will exhibit differences in maximum SBPmax behavior: a child with a BSA of 1.25m^2^will have a SBPmax of 140 mmHg, while another with a BSA of 1.75m^2^will reach 160 mmHg.^421 , 422^

SBPpeak/SBPmax, or that measured immediately after cessation of exertion, are considered the standard for assessing cardiac inotropic capacity. Changes in BP behavior are useful for diagnosis, definition of treatment, and risk stratification in children and adolescents with CHD, valvular heart disease, HF, or suspected hypertension.^53 , 418 , 419^

During the recovery period, a progressive decline in SBP is observed, returning to resting levels in approximately 6 minutes. SBP generally remains below pre-exercise levels for several hours thereafter.^423^

Diastolic blood pressure (DBP) normally remains unchanged with exertion, regardless of age and sex, due to exercise-induced vasodilation. Minor fluctuations (±10 mmHg) may occur. In apparently healthy children, a slight drop in DBP may be observed.^424^

A study on the BP response to ET in normotensive Brazilian adolescents found an increase in SBP and a decline in DBP during exercise in all age groups and both sexes.^425^Other studies found that the increase in SBP and chronotropic response were significantly lower in children with complex CHD and dilated cardiomyopathy.^155 , 395 , 426^

Failure of SBP to increase with exertion may be indicative of possible cardiac dysfunction. A persistent drop in SBP with progression of exercise may be secondary to HF or left ventricular outflow tract obstruction (i.e. severe aortic stenosis, asymmetric hypertrophic cardiomyopathy).

National and international studies have sought to evaluate the BP response in children and adolescents undergoing ET and define reference values and predictive equations for BP behavior in this setting. Due to great heterogeneity across the studied populations and the results obtained, a single "normal" BP response to ET has, so far, proved impossible to define.^38 , 305 , 403 , 419 , 422 , 425 , 427 , 428^

To assess SBPmax, we suggest using:

– A predictive equation based on sex and age ( Table 22 ) for the age group 7 to 17 years; or
– A nomogram based on sex and body surface area ( Figure 2 ) for the age group 6 to 15 years.

**Table 22:** Predicted peak SBP values based on a linear regression model for age and sex

SBP peak (mmHg)						
Age	Male			Female		
	P90	P95	Predicted Mean	P90	P95	Predicted Mean
**7**	161	167	132	169	174	142
**8**	166	171	136	170	175	143
**9**	170	176	141	172	177	145
**10**	175	180	145	173	178	146
**11**	179	185	150	174	179	147
**12**	184	189	154	176	181	149
**13**	188	194	159	177	182	150
**14**	192	198	163	178	184	151
**15**	197	203	168	180	185	153
**16**	201	207	172	181	186	154
**17**	206	212	177	183	188	156
**Formula** *	SBP P95 = 135.40 + 4.48 × age SBP P90 = 129.75 + 4.48 × age Predicted mean SBP = 100.39 + 4.48 × age			SBP P95 = 164.39 + 1.37 × age SBP P90 = 159.21 + 1.37 × age Predicted mean SBP = 132.27 + 1.37 × age		

P: percentile; SBP: systolic blood pressure.

* Age in years. Adapted from: Sasaki et al. Blood Pressure Response to Treadmill Cardiopulmonary Exercise Test in Children with Normal Cardiac Anatomy and Function.^424^

**Figure 2:** Nomograms of maximum systolic blood pressure behavior based on sex, ethnicity, and body surface area. The solid line represents the 50th percentile (P50) of systolic blood pressure. The upper dashed line represents the 95th percentile (P95), while the lower dashed line represents the 5th percentile (P05). Body surface area (m²). Adapted from: Alpert et al. Responses to Ergometer Exercise in a Healthy Biracial Population of Children.^422^

Recommended criteria for evaluating and describing the BP response to ET in children and adolescents are given in Table 23 .

**Table 23:** Blood pressure response to ET/CPET in children and adolescents

Term	Criteria **
**Normal resting BP**	– Age 1 to <13 years: Normal resting BP: <P90 for age, sex, and height. – Age ≥13 years: Normal resting BP: <120 / <80 mmHg (Table 21). *
**Increased resting BP**	– Age 1 to <13 years: - SBP ≥P90 and/or DBP ≥P95 for age, sex, and height. – Age ≥13 years: BP ≥120 / ≥80 mmHg (Table 21). *
**Normal BP response to exercise and recovery** **	Normal resting BP. During exercise: *** – SBP <P95 (according to age- and sex-based reference tables), or – SBP <P90 in the 7-17 age range (Table 22), or – SBP lower than the maximum predicted values for age and sex: 12-13 years = ♀ and ♂ 172; 14-15 years = ♀174.7 / ♂177.3; 16-17 years = ♀178.5 / ♂201.3 mmHg.^38 , 41^ – DBP variation no greater than ±10mmHg (♀ and ♂). Normal BP during recovery: gradual drop in SBP to resting value at approximately 6 minutes.
**Pretest hypertension with normal BP response to exercise**	Normal resting BP. During exercise: *** – SBP <P95 (according to age- and sex-based reference tables), or – SBP <P90 in the 7-17 age range (Table 22), or – SBP lower than the maximum predicted values for age and sex: 12-13 years = ♀ and ♂ 172; 14-15 years = ♀174.7 / ♂177.3; 16-17 years = ♀178.5 / ♂201.3 mmHg.^38 , 41^ – DBP variation no greater than ±10mmHg (♀ and ♂).
**Hypertensive/exaggerated response to exercise**	Resting BP may be normal or elevated. During exercise: *** – SBP >P95 (according to age- and sex-based reference tables for BP during exercise), or – SBP >P90 in the 7-17 age range (Table 22), or – SBP equal to or greater than the maximum predicted values for age and sex: 12-13 years = ♀ and ♂ 172; 14-15 years = ♀174.7 / ♂177.3; 16-17 years = ♀178.5 / ♂201.3 mmHg.^38 , 41^ – DBP elevation ≥15mmHg (♀ and ♂).
**Hypotension drop in BP during exercise** ****	SBP during exercise lower than the resting SBP,^8^or Initial rise in SBP followed by a drop in SBP ≥20 mmHg.
**Depressed BP response**	The systolic pressure reserve (ΔSBP) is defined as the difference between SBPpeak during exercise and resting SBP.^424^ ♂: age 7-11 years = ΔSBP <10 mmHg; age 12-17 years = ΔSBP<20 mmHg. ♀: ΔSBP <10 mmHg (age 7-17 years).
**Normal recovery** **BP response**	– Progressive reduction in SBP. SBP and DBP return to resting values by the 6th minute of recovery.

BP: blood pressure; SBP: systolic blood pressure; DBP: diastolic blood pressure; ΔSBP: change in SBP with exertion; P: percentile; ♂ = male; ♀ = female.

* See Appendices 2 and 3 for percentiles.

** Describe whether the BP response occurred during use of drugs with an antihypertensive effect.

*** Note which reference table and percentile value were used.

**** Rarely, children and adolescents without clinically significant cardiac disease will experience exercise-induced hypotension; this may be due to dehydration, an inadequate dose of antihypertensive therapy, or prolonged strenuous exercise.

Particular features of the BP response to ET specific to the pediatric population:

– **White coat hypertension:** patients with this condition generally present with an exaggerated SBP response to exercise, which may represent a pre-hypertensive stage.^46^
– **Future risk of hypertension** : there is evidence that an exaggerated BP response to exercise in apparently healthy children and adolescents is a predictor of future hypertension.^429 , 430^
– **Association with** **left ventricular hypertrophy (LVH):** a hypertensive SBP and/or DBP response in normotensive children and adolescents (especially those with a family history of hypertension) correlates with the degree of LVH.^47 , 431 – 433^
– **Aortic stenosis:** As aortic valve stenosis (subvalvular or supravalvular) becomes more severe, the increase in SBP during exercise is significantly reduced. In severe stenosis, the increase in SBP is generally between 10 and 20 mmHg.^134 , 383 , 434 , 435^Rarely, a drop in SBP during exercise may occur, which is associated with impaired ventricular function (gradient >70 mmHg).^436^ΔSBP on exertion ≥35 mmHg has been associated with better prognosis.^437^
– **Hypertrophic cardiomyopathy:** ΔSBP on exertion <20 mmHg or a drop in SBP >20 mmHg in children and adolescents is associated with increased risk of cardiac death.^155 , 438^
– **Coarctation of the aorta:** After successful surgical repair, up to one third of patients remain or become hypertensive. A hypertensive response to exercise is common, even in the absence of significant residual obstruction.^49 , 439 , 440^
– **Athletes:** in physically active children and adolescents, the increase in SBP in response to exertion is usually slower than in sedentary and obese subjects.^441^Apparently healthy, highly trained adolescents generally have higher ΔSBP than untrained youth. Equations for predicting SBP in athletes (aged 10 to 18 years) at any time during ET:^442^

**Male:** SBP during exercise (mmHg) = –1.92 × age + 0.55 × workload + 120.84

**Female:** SBP during exercise (mmHg) = –0.88 × age + 0.48 × workload + 111.22

Note: age in years; workload in watts (W).

##### 3.2.3. Double Product

The double product (DP), or rate pressure product, expresses myocardial oxygen consumption. It is calculated by multiplying the HR by the SBP at any time during ET/CPET:


DP(bpm.mmHg)=HR x SBP

In children and adolescents, resting DP is generally influenced by sex (lower in females), anthropometric parameters (BMI, waist-to-hip ratio, and body fat percentage), and level of CRF. Annex 4 presents information on DP values at rest and at peak exertion in an apparently healthy pediatric population, in patients with HF and with coarctation of the aorta.^428 , 443 , 444^

DP response:

– In apparently healthy children, correlates positively with age.
– In the second stage of incremental protocols and at peak exertion, it is a useful predictor of systolic hypertension in adolescence, regardless of resting SBP and conventional cardiovascular risk factors.^445^
– Patients with Kawasaki disease have a significantly lower maximum DP.^15^

#### 3.3. ECG Responses

For proper analysis, description, and interpretation of ECG responses to ET in the pediatric population, the following factors should be taken into account:

– Check proper electrode placement and attachment to minimize errors and artifacts.^446 , 447^
– Consider the effects of any ECG filters applied (high, medium, low) for baseline stabilization and reduction of muscle and electrical artifacts. For teenagers, use high-frequency filters (at least 150 Hz); for children, up to 250 Hz. Filters with lower frequencies may interfere with capture of pacemaker spikes.^308 , 448 , 449^
– It is suggested to use automated measurement systems for intervals, durations, and amplitudes of ECG waves and segments, adapted and validated for the pediatric population.^11 , 188 , 450^
– Follow the standard ECG reporting guidance of the Brazilian Society of Cardiology Guidelines on the Analysis and Issuance of Electrocardiographic Reports – 2022 and the reference values for key ECG parameters adjusted for the various age ranges of the pediatric population ( Table 24 ).^308 , 451^
– Review any automated measurements to rule out errors due to possible interference, artifacts, or abnormalities in the underlying tracing.^452 , 453^
– Provide a detailed, contextualized description in the ECG record, adapted for the pediatric population and its diseases.

**Table 24:** Reference values of key ECG parameters at rest in children and adolescents

	1-3 years		3-5 years		5-8 years		8-12 years		12-16 years	
	Min	Max	Min	Max	Min	Max	Min	Max	Min	Max
HR (bpm)	89	152	73	137	65	133	62	130	60	120
P amplitude, lead III (mV)	0.07	0.25	0.03	0.25	0.04	0.25	0.03	0.25	0.03	0.25
P duration (ms)	63	113	67	102	73	108	78	117	78	122
SâP	-12	19	-13	69	-54	72	-17	76	-24	76
PR interval, lead ll (ms)	80	150	80	160	90	160	90	170	90	180
SâQRS	7	102	6	104	10	139	6	116	9	128
QRS V5 (ms)	30	80	30	70	30	80	40	90	40	90
Q aVF (mV)	0.00	0.32	0.00	0.29	0.00	0.25	0.00	0.27	0.00	0.24
Q V1 (mV)	0.00	0.00	0.00	0.00	0.00	0.00	0.00	0.00	0.00	0.00
Q V6 (mV)	0.00	0.28	0.01	0.33	0.01	0.46	0.01	0.28	0.00	0.29
R V1 (mV)	0.20	1.80	0.10	1.80	0.10	1.40	0.10	1.20	0.10	1.00
R V6 (mV)	0.60	2.30	0.80	2.50	0.80	2.60	0.90	2.50	0.70	2.30
S V1 (mV)	0.10	2.10	0.20	2.20	0.30	2.30	0.30	2.50	0.30	2.20
S V6 (mV)	0.00	0.70	0.00	0.60	0.00	0.40	0.00	0.40	0.00	0.40
T V1 (mV)	-0.60	-0.10	-0.60	0.00	-0.50	0.20	-0.40	0.30	-0.40	0.30
T V6 (mV)	0.10	0.60	0.15	0.70	0.20	0.75	0.20	0.70	0.10	0.70
R/S V1	0.10	4.30	0.03	2.70	0.02	2.00	0.02	1.90	0.02	1.80
R/S V6	0.30	27.00	0.60	30.00	0.90	30.00	1.50	33.00	1.40	39.00
QTc (ms)	381	455	377	448	365	447	365	447	362	449

Min: minimum; Max: maximum; HR: heart rate; ms: milliseconds; mV: millivolts; bpm: beats per minute; QTc: corrected QT interval; SâP: P wave axis; SâQRS: axis of the QRS complexes. Adapted from: Samesima N et al. Brazilian Society of Cardiology Guidelines on the Analysis and Issuance of Electrocardiographic Reports – 2022.^308^

The following factors must be taken into account regarding ECG in the pediatric population:^451^

– The ECG must be interpreted according to the child's age. Younger children have a precordial pattern with right ventricular dominance; as they age, the waveform takes on the adult ECG pattern, with physiological left ventricular predominance.
– In CHD, the ECG reflects any anatomic changes and their hemodynamic repercussions on the chambers of the heart.
– Chest deformities, cardiac malposition, and/or changes in heart rhythm will hinder interpretation.

##### 3.3.1. Resting ECG

In children and adolescents, certain resting ECG abnormalities are associated with pathological conditions, increased risk of complications during ET/CPET, and risk of sudden death ( Table 25 ). These abnormalities can interfere with the interpretation of exercise-induced changes.

**Table 25:** Resting ECG abnormalities in children and adolescents known to be associated with pathological conditions, increased risk of complications during ET/CPET, and risk of sudden death^64 , 462^

ECG component	Change	Associations
P wave	Enlargement, left atrial hypertrophy: negative portion of the P wave in lead V1 with depth ≥0.1 mV and duration ≥0.04 s.	VHD; CHD.
	Enlargement, right atrial hypertrophy: sharp P wave in leads II and III or V1 with amplitude ≥0.25 mV.	VHD; CHD.
QRS complex	Frontal plane axis deviation: right ≥120° or left −30° to −90°.	CHD; DCM; intraventricular conduction disorders.
	Increased amplitudes: R or S wave amplitude in limb leads ≥2 mV, S wave in lead V1 or V2 ≥3 mV, or R wave in lead V5 or V6 ≥3 mV.	CHD; LVH; VHD.
	Abnormal Q waves (duration ≥0.04 s or ≥25% of subsequent R wave height) or QS pattern in two or more leads.	HCM; DCM; LVNC; myocarditis; history of IM.
	Right or left bundle branch block with QRS duration ≥0,12 s.	DCM; HCM; LVNC; sarcoidosis; myocarditis; CHD.
	Epsilon wave (positive deflection at the end of QRS in leads V1 and V2).	ARVC.
ST segment, T wave and QTc interval	ST segment depression.	CHD; HCM; DCM; LVNC; ARVC; myocarditis.
	T wave inversion or flattening in two or more leads (lateral leads).	HCM; DCM; LVNC; ARVC; myocarditis.
	Prolongation of QTc (corrected for heart rate) >0.44 s in males and >0.46 s in females.	Long QT syndrome.
Rhythm and conduction abnormalities	Premature ventricular contractions or more serious ventricular arrhythmias.	HCM; DCM; LVNC; ARVC myocarditis; sarcoidosis.
	Supraventricular tachycardias, atrial flutter, or atrial fibrillation.	Cardiomyopathy or electrical heart disease.
	Short PR interval (<0.12 s) with or without delta wave.	WPW; short PR syndrome.
	Sinus bradycardia with resting heart rate ≤40 bpm.	CHD; sinus node dysfunction; cardiomyopathy or electrical heart disease.
	First-degree (PR interval ≥0.21 s), second-, or third-degree AV block.	Cardiomyopathy or electrical heart disease; congenital atrioventricular block.

ARVC: arrhythmogenic right ventricular cardiomyopathy; MI: myocardial infarction; DCM: dilated cardiomyopathy; HCM: hypertrophic cardiomyopathy; LVNC: left ventricular noncompaction; CHD: congenital heart disease; VHD: valvular heart disease; WPW: Wolff-Parkinson-White syndrome; LVH: left ventricular hypertrophy; QTc: corrected QT interval.

The early repolarization pattern (ERP) is common in the pediatric population and must be interpreted in context:^454 , 455^

– The diffuse ascending pattern is common among the young, in those of European ethnicity, found equally in both sexes and has no apparent correlation with atrial or ventricular arrhythmias.^456^
– Pediatric athletes often present with a notched J-point and a rapidly ascending, concave ST segment, especially in the inferolateral leads. Other changes include resting sinus bradycardia, increased R wave voltage in precordial and peripheral leads, and an increased Sokolow-Lyon index.^457^
– In athletes aged ≥14 years, use of the Seattle criteria is recommended for improved diagnosis.^458 – 460^

Other causes of ERP include: juvenile T wave pattern; hypothermia or hyperthermia; hypocalcemia; hyperkalemia; pericardial disease (pericarditis, pericardial cyst, pericardial tumor); myocardial tumor (lipoma); hypertensive cardiomyopathy; myocardial ischemia; thymoma; arrhythmogenic right ventricular cardiomyopathy; Takotsubo cardiomyopathy; myocarditis; and Chagas disease.^458 , 459 , 461^

##### 3.3.2. Responses to Exercise and Recovery

In the healthy pediatric population, the ECG responses to ET/CPET (exercise and recovery) are generally different from those observed in adults, including in terms of criteria for diagnosis of ischemia; these differences will be presented below.

###### 3.3.2.1. P Wave and PR Interval

On the resting ECG, P waves represent atrial depolarization and are best visualized in leads II and V1. Normal sinoatrial node conduction will result in a positive P wave in leads I, II, and aVF. The maximum amplitude of the P wave does not change significantly during childhood ( Table 24 ), and its duration is usually <100 ms. An amplitude >0.25 mV (2.5 mm) in lead II is considered abnormal at any age.^450 , 463 – 465^

In children, the amplitude and duration criteria for atrial hypertrophy should only be applied in sinus rhythm with a P wave axis between 0 and 90°. A P wave amplitude >0.25 mV (2.5 mm) suggests right atrial enlargement. A broad, notched (bifid) P wave (duration >110 ms) in lead II and/or a biphasic P wave in lead V1 with a terminal negative deflection >40 ms suggests left atrial enlargement.^466 , 467^

P wave dispersion (Pdis, Pd or PWD) corresponds to the difference between the maximum and minimum duration values of the P waves in the ECG leads. Pdis and maximum P wave duration on the resting ECG are useful for evaluating the sinus impulse propagation pattern and intra- and interatrial conduction times, and has PPV for arrhythmias in children with CHD.^468 , 469^

The PR interval varies with age range (see Table 24 ). Its lower limit is between 80 and 90 ms, and the upper limit, between 150 and 180 ms. Key changes in PRi duration:

– Prolonged: generally associated with CHD, myocarditis, and hyperkalemia.
– Short: associated with Wolff-Parkinson-White (WPW) syndrome and its pre-excitation variations, and glycogen storage diseases.

During ET, the following are generally observed:

– Increased P wave amplitude. In apparently healthy children of both sexes aged 5 to 12 (mean age 10.3 years), the P wave amplitude at peak exertion can be as high as 2.57±0.76 mm (resting ECG: 1.84±0.48 mm; p<0.001).^470^
– Progressive decrease in PRi with increasing HR, due to accelerating propagation of potentials through the atria and atrioventricular node (sympathetic activation). At peak exertion, the PRi generally ranges from 100 to 140 ms.^177^
– During recovery, an increase in PRi duration is often observed, with a concomitant decrease in HR; this may be associated with sinus arrhythmia, short runs of junctional rhythm, and ectopic atrial rhythm.

Abnormal responses to ET:

– Prolonged P wave duration, increased maximum P wave duration, and increased PDW on resting ECG have been described in association with ostium secundum atrial septal defect in otherwise healthy children, atrial hypertrophy, pulmonic stenosis, tetralogy of Fallot, Eisenmenger syndrome, status post Fontan procedure, interatrial block, chemotherapy-induced cardiomyopathy, arrhythmias, hypertension, and viral infections.^464 , 468 , 471 – 475^
– Ectopic atrial rhythm (inverted P waves in leads II and/or aVF) on resting ECG usually returns to sinus rhythm with exertion/increasing HR. Persistence of ectopic atrial rhythm is generally observed in patients with CHD.^476 , 477^
– In children and adolescents with marked first-degree AV block (extremely prolonged PRi) on resting ECG and persistence of this abnormality as the test progresses, exercise intolerance, palpitations, and pre-syncope/syncope often occur; this is associated with atrioventricular dissociation and is diagnostic of the pseudo-pacemaker syndrome.^478 . 479^

###### 3.3.2.2. Q Wave

Q-wave behavior differs as the child grows. In newborns, it is normally absent or of small amplitude. Children aged 6 months to 3 years may have abnormal Q waves (in leads III and V6) of up to 0.6-0.8 mV. Q wave amplitude in the first months of life reaches peak around 3 to 5 years of age, with a subsequent decrease, but without normalizing.^450 , 463 , 480 , 481^

In apparently healthy children between 8 and 16 years of age, Q waves in lead V6 can reach up to 0.23-0.5 mV. In adolescents, it is suggested that, instead of the Seattle Criteria, the International Criteria for pathological Q waves be adopted:^482 , 483^

– In the Seattle Criteria, pathological Q waves are defined as those >3 mm in depth or >40 ms in duration in two or more leads (excluding leads III and aVR).^460^
– In the International Criteria, pathological Q waves are defined as a Q/R ratio ≥0.25 or a Q wave ≥40 ms in duration in two or more leads (excluding III and aVR).^484^
– Adoption of the International Criteria led to an ≈84% reduction in false-positive ECGs due to pathological Q waves, as they reduce the effects of increased QRS complex voltage secondary to athletic training and/or low impedance in lean adolescents.^485 , 486^

Particular features of the Q wave:

– During pre-test evaluation, abnormal Q waves on a resting ECG suggest an accessory pathway to be confirmed. Isolated pathological Q waves in leads V1 and V2 are generally due to inadequate electrode placement. The finding of pathological Q waves in two or more contiguous leads may be associated with dilated cardiomyopathy, hypertrophic cardiomyopathy, left ventricular noncompaction, and past myocardial infarction (due to Kawasaki disease, anomalous origin of coronary arteries, etc.).^476 , 484 , 487 , 488^
– In a case-control study of 44 patients with Kawasaki disease (age 7.7±4.8 years), 22 patients underwent ET to investigate myocardial ischemia, of whom 50% exhibited ischemic changes (7 with abnormal Q waves) with significant CAD on coronary angiography. The coronary lesion severity score on SPECT was significantly higher in those with abnormal Q waves (51.0±38.8 versus 20.0±12.1, p<0.05).^489^

###### 3.3.2.3. R Wave and S Wave

In children >3 years of age (as in adults), normal ventricular activation is observed in the horizontal plane (precordial leads), with a dominant S wave in V1, similar R and S amplitudes in V2 and V3, and dominant R waves from V4 to V6.^490^

In apparently healthy children, the amplitude of the R wave in leads in which it is normally prominent (V5 and V6) generally decreases by an average of 5 mm with exercise. However, the amplitude of the R wave may remain unchanged or even increase.^470 , 491^R wave amplitude responses appear to have no diagnostic significance, unlike in the adult population.

On the pediatric resting ECG, the finding of an R wave >25 mm in V6, a Q wave >5 mm in V6, and an S wave >20 mm in V1 suggests left ventricular hypertrophy.

During exertion, S-wave amplitude generally remains unchanged or increases slightly, while during recovery there is usually an increase.^470^

Particular features of R and S waves in the pediatric population:

– A study of 170 apparently healthy Black children aged 7 to 14 years (mean age 10.5 years; 56% female), designed to determine the pattern of ECG response to exertion, found that R wave amplitude decreased by 27±8 to 22±8mm (p<0.01) while S-wave amplitude increased from 6.9±4.4 to 7.8±5 mm (p<0.01).^491^
– A study of 46 adolescents (average age 16.1 years; all male) designed to evaluate the change in R wave amplitude in lead V5 during ET found that, in normotensive subjects, there was a progressive reduction in R wave amplitude (up to −3.8mm), while in hypertensive patients there was no such reduction (p<0.001).^492^
– A study of 55 adolescents (average age 15.9 years; 29 with HTN), designed to evaluate the effect of pharmacotherapy on the R wave amplitude response during ET, found that, after 16 weeks of antihypertensive treatment, the amplitude showed reduction and pattern similar to that observed in normotensive subjects.^493^
– QRS duration usually remains unchanged or decreases slightly during progressive exertion.

###### 3.3.2.4. T Wave and U Wave

In childhood, the T wave pattern – particularly in the precordial leads – is different from that seen in adults, with a progressive change in the T wave axis with age. Persistence of a positive T wave in V1 or V3R beyond the first week of life usually occurs in right ventricular hypertrophy (RVH). The T wave generally remains inverted in V1 and V3R from age 12 to 16 years.^494 , 495^

In early childhood, the T wave is often inverted in leads V2 and V3, progressing to positivity with advancing age. Only 5 to 10% of 8-to-12-year-olds have inverted T waves in V2.^496 – 499^In V5 and V6, the T wave is generally positive in all age groups.^388 , 500^

Presence of negative T waves (NTW), or T wave inversion, on resting ECG:

– Is considered abnormal if seen in two or more contiguous leads (excluding V1, aVR, and III) and with a depth of ≥1 mm. In lateral leads (II, III, aVF, V4-V6), it is usually associated with hypertrophic cardiomyopathy and LV hypertrophy.^501 – 503^In adolescent athletes, inverted T waves in lateral leads are also usually associated with hypertrophy and apical displacement of the papillary muscles, which can be considered normal.^504 – 506^
– Asymmetric or biphasic inverted T waves without ST-segment depression in leads V1-V4 are relatively common in asymptomatic adolescents (age <16 years) and Black youth athletes.^484 , 496 , 501 , 507^
– NTW in anterior leads preceded by J-point elevation with ST-segment elevation are present in up to 25% of young Afro-Caribbean athletes and is considered particularly characteristic of "the Black athlete's heart".^501 , 508 , 509^However, the finding of ST-segment elevation without J-point elevation preceding inverted T waves may associate to cardiomyopathy.^508 , 510^
– NTW in inferior and anterior leads (from V1 to V3) followed by positive T waves in V5 (the T wave discontinuity phenomenon) are generally associated with arrhythmogenic right ventricular cardiomyopathy (ARVC).^511 , 512^

Particular features of the T wave in ET:

– In healthy children, T wave duration decreases progressively with increasing exertion. While the amplitude generally decreases during light exercise, it subsequently increases with progression of exertion, and may exceed the baseline amplitude at peak exertion (in V5, 4.8mm at rest to 7.3mm at V5).^177 , 470 , 491 , 500^
– ET is normally used to evaluate NTW behavior and associated exercise-induced arrhythmia, including in adolescent athletes.^462 , 484 , 504 , 513^
– In the pediatric population with NTW, asymptomatic and in the absence of heart disease, pseudonormalization of the T wave (positive T wave) is common, either complete (in all leads) or partial (in lateral leads). This is a generally benign phenomenon and is not associated with a risk of cardiac events.^514 , 515^
– In young athletes with NTW, development of ventricular tachycardia or increased density of exercise-induced ventricular ectopic beats is considered suggestive of arrhythmogenic cardiomyopathy.^516 , 517^
– In congenital long QT syndrome, T wave alternans may occur, with chronotropic incompetence, ventricular tachyarrhythmias, and paradoxical QTi behavior (increasing instead of decreasing).^6^

###### 3.3.2.5. ST Segment/ST Segment Depression

Exercise-induced ST segment changes have been used to identify myocardial ischemia in children, adolescents, and adults. In the pediatric population, the criteria for ischemia are different from those applied in adults, corresponding to ST-segment depression, horizontal or downsloping (>1 mm below baseline), measured at the Y point (at 60 ms after the J point).^7 , 11 , 177 , 300^

In this population, two baseline definition criteria are used to measure ST segment depression ( Figure 3 ):^7 , 11^

**Figure 3:** Isoelectric baseline definition methods for measuring ST segment changes. Regardless of the method used, the resulting baselines yield similar points for measuring/quantifying possible St-segment depression or elevation. * Point on which the measurement of depression or elevation should be based when using the PR isoelectric line method.

1. PR method – the baseline (P-R isoelectric line) is superimposed on the P-R segment of the QRS complex to identify the J point.
2. PQ-PQ method – the baseline is defined by connecting the P-Q points of at least three consecutive QRS complexes to identify the J point.

Exercise-induced isolated J-point depression (without ST depression) has no bearing in the diagnosis of ischemia. In the asymptomatic, apparently healthy pediatric population, J-point depression in relation to a PQ isoelectric line was observed in 9% of boys and 18% of girls, while by the PR isoelectric line method, it was seen in 2.3% of both sexes.

In the apparently healthy pediatric population, exercise-induced ST depression is considered a normal, non-ischemic finding ( Figure 4 ) under the following circumstances:^7 , 11 , 300^

**Figure 4:** ST segment behavior and types of ST depression. ST-seg.: ST segment; ms: milliseconds. ST segment depression <1mm of any morphology, especially if there is early normalization (in the first minute of recovery), is considered a normal, non-ischemic finding. *Upsloping (J point depression followed by rapidly ascending depression of the ST segment and no depression at the Y point, measured at 60 ms from the J point) or slow ascending (decreased J point with the ST segment slowly ascending beyond the Y point) are considered normal and non-ischemic. **Horizontal or downsloping (ST segment depression >1mm measured at point Y) is considered abnormal and indicative of ischemia.

– Morphology upsloping (J point depression followed by rapidly ascending depression of the ST segment and no depression at the Y point, measured at 60 ms from the J point) or slow ascending (decreased J point with the ST segment slowly ascending beyond the Y point).
– Any morphology if <1 mm, especially if there is early normalization (in the 1^st^minute of recovery).

The following situations render interpretation of repolarization changes useless for diagnosis of ischemia: Wolff-Parkinson-White syndrome; variants of the pre-excitation syndromes; left bundle branch block; artificial ventricular pacemaker; ST segment depression >1mm on resting ECG; digitalis therapy; and unsatisfactory technical quality of the ECG tracing.^7^

Particular features of ST-segment depression in the pediatric population:

– Exercise-induced ST depression not associated with ischemia due to CAD may occur due to hyperventilation, fluid-electrolyte imbalance, anemia, *pectus excavatum* , and mitral valve prolapse.^177 , 286^
– In acquired aortic stenosis, exercise-induced ST-segment depression occurs in ≈83% of patients and is associated with LV systolic pressure, LV outflow gradient, and O_2_supply-demand imbalance. After surgical correction of severe aortic stenosis, exercise-induced ST-segment depression usually improves or disappears altogether.^434 , 518 , 519^
– Exercise-induced ST-segment depression is also common in congenital aortic stenosis. However, after Ross surgery there is no significant reduction in exercise-induced ST-segment depression. An increase in exercise-induced ST-segment depression has been observed after aortic valvuloplasty (surgical or balloon).^140^
– After Fontan procedure in hypoplastic left heart syndrome (HLHS), exercise-induced ST depression – which occurs in ≈48% of patients – is not associated with ventricular dysfunction, CAD, or anomalous origin of coronary arteries.^520^
– Exercise-induced ischemia in patients with HCM is associated with a higher risk of sudden cardiac death (RR: 3.32; 95% CI: 1.27-8.70) and a composite of all-cause mortality and/or transplantation (RR: 4.86; 95% CI: 1.69-13.99).^157^

###### 3.3.2.6. ST Segment Elevation

Exercise-induced ST-segment elevation is defined as an ST-segment elevation ≥1.0 mm (≥0.10 mV) at 60 ms after the J-point, occurring in two or more leads, regardless of the presence of a Q wave ( Figure 5 ).^1 , 7 , 521^

**Figure 5:** ST-segment elevation patterns, including early repolarization. ST-seg.: ST segment; ms: milliseconds. * Exercise-induced ST-segment elevation (≥1.0 mm measured at 60 ms after the J-point). ** In the early repolarization pattern, ST-segment elevation should be measured 100 ms after the Jt point, and the pattern of elevation (upsloping, horizontal, or downsloping) should also be assessed.

In the pediatric population, exercise-induced ST-segment elevation is generally associated with: severe myocardial ischemia (usually transmural) in patients with Kawasaki disease, anomalous origin of coronary arteries, and after coronary reimplantation surgery, among others; coronary artery spasm due to vasospastic or Prinzmetal angina; left ventricular aneurysm; and peri-infarction ischemia.^521 – 524^

The following anatomic-topographic correspondences can be used when describing leads showing ischemic manifestations:^1 , 308^

- V1, V2, V3 (likely anteroseptal wall).
- V1, V2, V3, and V4 (likely anterior wall).
- V3, V4, or V3-V5 (likely localized anterior wall).
- V4, V5, V6, lead I, and aVL (likely anterolateral wall).
- V1-V6, lead I, and aVL (likely extensive anterior wall).
- V5 and V6 (likely lateral wall).
- Lead I and aVL (likely high lateral wall).
- Lead II, III, and aVF (likely inferior wall).

Particular features of exercise-induced ST segment elevation:^1 , 7 , 521^

– On a resting ECG, the presence of ST segment elevation is generally associated with ERP, Brugada syndrome, myocarditis/pericarditis, and prior myocardial infarction (with pathological Q wave).
– In ERP and Brugada syndrome, a reduction/disappearance of ST segment elevation is generally observed with exercise.^126 , 525 , 526^
– Exercise-induced ST segment elevation ≥0.3 mV (3 mm) in leads without Q waves mandates test cessation.

###### 3.3.2.7. Early Repolarization

In most patients, early repolarization (ER) is an asymptomatic, benign ECG variant, with elevation of the J point and characteristic elevation of the ST segment. However, some patients exhibit clinical features and specific ER patterns on ECG that are associated with SCD, and thus constitute early repolarization syndrome (ERS). The early repolarization pattern (ERP) is seen in 1% to 13% of the general population.^527 , 528^

Particular features of the resting ECG with ERP include:^529 , 530^

1. QRS complex duration <120 ms.
2. Terminal QRS notching or slurring on the downstroke of a prominent R wave. If there is notching, it must be completely above the baseline. The point of J-wave onset (Jo) must also be above the baseline ( Figure 5 ).
3. The peak of the J-point notch (Jp) must be ≥0.1 mV in two or more contiguous leads of ECG, except V1 through V3.^531^
4. Pediatric athletes often present with a notched J-point and a rapidly ascending, concave ST segment, especially in the inferolateral leads. Other changes include resting sinus bradycardia, increased R wave voltage in precordial and peripheral leads, and an increased Sokolow-Lyon index.^457^
5. In athletes aged ≥14 years, use of the Seattle criteria is recommended for improved diagnosis.^458 – 460 , 484 , 501 , 532^

In ERP, ST-segment elevation should be measured 100 ms after the Jt point (termination of the J-point notch). In addition to the magnitude of elevation, the pattern should be described:^454 , 455^

– "Early repolarization with upsloping ST segment", when the ST segment is ascending (inclined upwards) and followed by a vertical T wave.
– "Early repolarization with horizontal or downsloping ST segment", when the ST segment is horizontal or descending (inclined downwards).

Behavior and significance of ERP in ET:

– Common in adolescents. In this setting, usually reduces progressively with increasing exertion, and may disappear altogether at moderate loads. ERP with rapidly upsloping ST segment elevation in the anterolateral leads has been reported in athletes.^533^
– Persistent ERP, sustained ventricular arrhythmia, and/or unexplained syncope has been observed in ET after aborted sudden cardiac death.^534^
– Exercise-induced polymorphic VT is a marker of high risk for SCD.^527 , 535^
– In the general population, the ERP usually reappears progressively and slowly during recovery.^536 , 537^

###### 3.3.2.8. QT Interval

The QT interval (QTi) represents the total duration of ventricular electrical activity. It is measured from the start of the QRS complex to the end of the T wave.^538 – 540^

Assessment of QTi during exercise and recovery is beset with challenges in children and adolescents:

– Accurate measurement of QTi is often hindered by irregular return of the terminal portion of the T wave to baseline.
– At high HRs, fusion of the T and P waves is common, making the end of the T wave difficult to identify.

The increase in ventricular myocardial repolarization velocity associated with exertion is reflected in the progressive shortening of QTi until maximum exertion and linear widening of the interval during recovery.^541^

Due to the variation of QTi with HR, correction of QTi for HR (QTc) by Bazett's formula is recommended:

$$QTc=\frac{QTi}{\sqrt{RR}}$$

*QT measured in milliseconds and distance between RR in seconds.

Table 24 presents QTc reference values for each pediatric age group.

The ideal formula for QTc adjustment in the setting of ET remains controversial. Interpretation of the QTc and comparison of its values with results published in the literature depend on the formula used for correction.^124 , 542 – 544^

In studies investigating repolarization changes (for example, in long QT syndromes, congenital heart defects, or new drug safety trials), Bazett's formula has limitations for HR <60 bpm or >90 bpm; in these situations, use of the Fridericia or Framingham formulas is recommended instead:^308 , 544 – 546^


QTc(Fridericia'sformula)=QT/RR3QTc(Framinghamformula)=QT+0.154(1-RR)

In children aged 1 to 15 years, a QTc >440 ms is considered borderline/upper limit of normal, while a QTc >460 ms is considered prolonged (irrespective of sex). QTc is considered short when its duration is <340 ms.^308 , 451^

Assessment of QT behavior is important in the diagnosis of congenital long QT syndrome, in which QTc prolongation may occur during exercise and recovery alike.

Particular features of the QTi and QTc in the pediatric population:

– During recovery, the QTi lengthens as HR decreases, by ≈15 ms for each 10-beat reduction in HR, returning to baseline (resting pattern) in approximately 4 to 5 minutes.^547^
– In children with borderline QTc/intermediate Schwartz score, ET allows risk stratification, selecting those who should undergo selective genetic testing.^57 , 547 – 549^
– Absolute QTc ≥460ms during recovery or a paradoxical increase in QTc (ΔQTc = QTc recovery – QTc baseline, with value ≥30ms) can distinguish patients with LQT1 manifest vs. hidden.^542^
– In screening for LQTS in children, use of Bazett's formula is associated with a high number of false positives, especially if the HR is increased. In these cases, the Fridericia formula must be used instead.^544^

##### 3.3.3. Disorders of Atrioventricular Conduction, Intraventricular Conduction, and Impulse Formation in the Pediatric Population

###### 3.3.3.1. Atrioventricular Conduction Disorders

In children and adolescents, first-degree atrioventricular (AV) block and type I second-degree (Mobitz I) AV block are generally manifestations of marked parasympathetic activity. They are common in highly trained adolescents and individuals with increased vagal tone. It is observed on resting ECG in 0.65 to 1.1% of children and up to 12% of apparently healthy adolescents.^550 , 551^These phenomena generally disappear with progressive exertion due to withdrawal of vagal activity and increased sympathetic activity. They are rarely triggered by exertion.^105^

Type II second-degree AV block usually represents disease of the AV (infranodal) conduction system. Rarely, it is found in apparently healthy young athletes. It may be associated with bundle branch block and occur secondary to cardiac surgery. ET may be useful in identifying the anatomical level of AV blockade, as well as in risk stratification.^552 , 553^

In the pediatric population, third-degree AV block (or complete heart block):^108 , 115 , 554 , 555^

– In congenital total atrioventricular block (CAVB), definitive PM is indicated: symptomatic; resting HR <55 bpm or <70 bpm when associated with structural heart disease. Table 28 presents the main causes of CAVB.^107 , 114^
– ET can be performed in individuals with congenital AV block if there are no comorbidities (congenital or otherwise) that would jeopardize patient safety.
– ET is used to document symptoms, assess increased ventricular escape response, ascertain whether ectopy is present, and assess the hemodynamic repercussions of the block.
– Many patients may exhibit normal functional capacity.
– VO_2_max and HRmax prediction equations should not be used.
– There is considerable variability in the escape HR that can be generated by the ventricular pacemaker (usually between 50 and 145 bpm).
– The natural history of congenital complete heart block consists of a progressive decline in ventricular rates throughout life. On resting ECG, between the ages of 6 and 10 years, the average HR is 50 bpm; between 16 and 20 years, 45 bpm; and over age 40 years, 38 bpm.
– Fatigue, dyspnea, dizziness, and exercise-induced ventricular ectopy accounted for 26.5% of pacemaker placements.^556^
– Exercise-induced ventricular ectopy is common (50-70% of patients) and is associated with an increased risk of sudden death.
– In patients with complete heart block and severe cardiac structural abnormalities, sudden death is generally associated with complex ventricular arrhythmia. Complete heart block located within the His-Purkinje system carries a worse prognosis.^116 , 557 , 558^

###### 3.3.3.2. Intraventricular Conduction Disorders

Intraventricular conduction disorders may be associated with systemic disease or underlying heart disease.

Right bundle branch block (RBBB) is common in apparently healthy children (between the ages of 6 and 17), with an incidence ranging from 0.16% to 2.9%, and is most common in females. RBBB can also occur in Ebstein's anomaly (prevalence 80-95%), ostium secundum ASD (prevalence ≈90-100%), arrhythmogenic RV dysplasia, and after surgery to correct ToF (≈11%) or VSD (≈6%). RBBB with left anterior fascicular block (LAFB) occurs mainly in CHD with endocardial cushion defects. RBBB on the baseline ECG invalidates the interpretation of ST changes on exertion, but only in leads V1 to V3.^1 , 7 , 279 , 370 , 553^

Left bundle branch block (LBBB) on resting ECG must be distinguished from Wolff-Parkinson-White syndrome (right free wall accessory pathway). As an isolated finding, LBBB in adolescents is rare and may be associated with progressive disease of the intraventricular conduction system, with or without cardiomyopathy. It can also occur after left ventricular outflow tract surgery. LBBB on baseline ECG poses a challenge for the analysis of ST segment findings as indicative of myocardial ischemia, thus reducing the specificity and accuracy of ET.^7 , 149 , 388 , 559^

Exercise-induced intraventricular conduction disorders, characterized by right bundle branch block or left bundle branch block, rarely occurs in the pediatric population. These phenomena can be observed both in apparently healthy children and in those with structural heart disease.^177^

###### 3.3.3.3. Disorders of Impulse Formation

Development of abnormal heart rhythms during ET is common in pediatric patients with and without CVD. These arrhythmias are often isolated, transient, episodic, and asymptomatic. Their classification in terms of morphology, interrelations, and density is similar to that employed in adults, as described in the Brazilian Guideline for Exercise Testing in the Adult Population – 2024.^1 , 149 , 278 , 280^

Key markers for risk of development of exercise-induced arrhythmias include: severe LV dysfunction; artificial pacemaker; history of arrhythmia or rhythm disorder; non-sinus baseline rhythm; CHD; and CHD correction surgery.^105 , 560 , 561^One study found that 28% of pediatric patients undergoing ET developed abnormal heart rhythms, of which 3% were clinically important (ventricular tachycardia, supraventricular tachycardia, second-degree AV block, atrial fibrillation, etc.); this occurrence was associated with severe LV dysfunction and past history of arrhythmia.^105^

####### 3.3.3.3.1. Ventricular Arrhythmias

In the pediatric population, isolated monomorphic premature ventricular contractions (PVCs) occur with a frequency of 0.3 to 2.2% on resting ECG. In asymptomatic children with no underlying heart disease, a normal ECG, and no family history of sudden cardiac death, this arrhythmia is almost always benign. PVCs tend to disappear as the child grows.^562 – 565^

ET is indicated for the assessment of ventricular arrhythmias in children and adolescents with:

– PVCs (isolated or paired) identified on an ECG performed during medical consultation.
– Palpitation, tachycardia, syncope, seizures, or dizziness during sports or other physical activities.
– Suspected channelopathies, anomalous pathway, or catecholaminergic ventricular tachycardia.

ET provides useful information regarding the behavior and risk of PVCs. These are considered benign when their density is reduced (or they are suppressed altogether) with exertion, as a result of sinus tachycardia.^279 , 390 , 566 , 567^

Apparently healthy children occasionally present with rare isolated exercise-induced PVCs, which could be considered benign. However, the occurrence of frequent, polymorphic, or complex ventricular ectopic beats (ventricular doublets and nonsustained ventricular tachycardia) suggests ventricular electrical instability.

Ventricular tachycardia (VT) is rare in the pediatric population. When present, it is generally associated with structural heart disease (particularly in left ventricular hypertrophy), hereditary conditions (catecholaminergic polymorphic ventricular tachycardia), or electrical disturbances (long QT syndrome), although it may be idiopathic (in apparently healthy young people).

Malignant ventricular arrhythmias generally occur early during exercise, due to electrical excitation triggered by sympathetic activity. In these cases, there is an increased risk of hemodynamically unstable tachyarrhythmias and SCD.

####### 3.3.3.3.2. Supraventricular Arrhythmias

Isolated premature atrial contractions on resting ECG are usually benign and disappear with exertion.^568^Isolated supraventricular extrasystoles (SVES) occur in ≈2% of apparently healthy children and in ≈4% of children with structural heart disease.^569^

Asymptomatic patients with isolated exercise-induced SVES generally have a good prognosis.^568^However, exercise-induced premature atrial contractions in children with a history of syncope or unexplained tachycardia require closer investigation, as they may trigger an episode of supraventricular tachycardia.

The incidence of paroxysmal supraventricular tachycardia (PSVT) in children is 0.1 to 0.4%. The most common presentations, according to age, are described in Table 26 .

**Table 26:** Prevalence of PSVT in children and adolescents according to age^570^

Type	>1 to <10 years	10 to 18 years	>18 years
Anomalous pathway	60-65%	50-60%	40-50%
Nodal reentry	15-20%	20-50%	50-70%
Atrial ectopic	4-6%	3-4%	3%

In children, exercise-induced PSVT (EI-PSVT) is rare, generally associated with reentry via ventricular conduction within the AV node or via extranodal accessory pathways (ventricular pre-excitation, WPW syndrome). In symptomatic children and adolescents, PSVT occurs in 12% of ETs.^569^

Proper diagnosis of EI-PSVT on a background of elevated HR is challenging due to the difficulty in identifying changes in P waves, even with normal QRS complexes ( Table 27 and Figure 6 ). In the pediatric population, the initial presentation of PSVT it is associated with the unexpected and abrupt increase in HR and/or other inadequate HR responses with changes in the exercise load.

**Table 27:** Electrocardiographic characteristics of sinus and supraventricular tachycardias in the pediatric population^570 , 571^

Type	P wave behavior	PR>RP’	HR (bpm)	AV block	Type	Age at onset	Particular features
**Sinus tachycardia**	Sinus	—	>HRmax predicted for age	No	NP	Any	HRmax reference values in Table 24 .
**IST**	Sinus pattern	—	>100	No	P or PE	>15 years	Chief complaint of palpitations; associated with anxiety, dizziness, pre-syncope, and syncope.
**Focal atrial tachycardia / EAT**	Inverted and notched P wave in V1 with P wave duration >90 ms	No	Atrial rate >150% predicted mean HR	Yes	I	≈7 years	May progress to tachycardiomyopathy, which is generally reversible with control of the arrhythmia.^572 , 573^
**Multifocal atrial tachycardia**	Various morphologies	—	>100	No	P	Any	Presence of at least 3 P wave morphologies and 3 different PR intervals.
**Junctional tachycardia** *	May present with no visible P waves (within or after the QRS)	—	>100	No	P or I	≈1 year	Rarely congenital; is in patients with no history of cardiac surgery, can be treatment-refractory, with high morbidity and mortality rates. Occurs in up to 5% of patients after cardiac surgery.^574^
**SANRT**	Sinus pattern	No	170-300	Yes	P	Any	—
**AAVRT**	Not visible	Yes	170-200	No	P	>6 years	Resting ECG usually shows delta wave. QRS during tachycardia is widened, aberrant, and may mimic ventricular tachycardia.
**Orthodromic atrioventricular reentry**	Diverse morphology depending on the location of the accessory pathway	Yes	220-360	No	P	<3 months or >6 years	QRS of tachycardia is usually narrow and the P wave is retrograde.
**PJRT**	Negative in inferior leads	No	<170	No	I	≈6 years	—
**Intra-atrial reentrant**	Flutter-like	—	160-220	Yes	I	≈12 years	—

SANRT: sinoatrial nodal reentrant tachycardia; AAVRT: antidromic atrioventricular reentrant tachycardia; PJRT: permanent junctional reciprocating tachycardia; EAT: ectopic atrial tachycardia; NP: non-paroxysmal (accelerates and ends gradually); P: paroxysmal; I: incessant; PE: persistent.

* Also known as junctional ectopic tachycardia (JET).

**Figure 6:** Diagnosis of non-sinus rhythm tachycardias in the pediatric population.^570 , 571^Tachyc.: tachycardia; Multif.: multifocal; Inap: inappropriate; AVNRT: atrioventricular nodal reentry tachycardia; AV: atrioventricular; PJRT: permanent junctional reciprocating tachycardia; SANRT: sinoatrial nodal reentrant tachycardia.

Atrial flutter and atrial fibrillation are relatively common in children with cardiomyopathies and CHD. Atrial flutter can be conducted to the ventricles at a 1:1 ratio (ventricular rate >300 bpm) or a 2:1 ratio (rate 150-200 bpm). Atypical atrial flutter (with slower, rounded, lower-voltage P waves separated by an isoelectric line) is a potentially lethal arrhythmia, generally present only in complex heart diseases.

Exercise-induced AF is uncommon in children; it can occur paroxysmally and asymptomatically in patients with heart disease.

####### 3.3.3.3.3. Bradyarrhythmias and Sinus Node Dysfunction

Bradyarrhythmia in the pediatric population is defined as a HR below the lower limit of normal for age (see Table 24 ). It commonly manifests as sinus bradycardia, junctional (escape) rhythm, or AV block (second-degree, advanced/high-grade, or complete).^370 , 575 – 578^

About 15-25% of healthy, asymptomatic children may present with sinus arrhythmia, ectopic atrial rhythm, multifocal atrial rhythm, and junctional rhythm. Junctional rhythm is common in children and adolescents with increased vagal tone, occurring in ≈45% of children aged 7-10 years, ≈13% of boys aged 10-13 years (during sleep), and ≈20% of adolescent athletes.^579^

Sinus node dysfunction (SND) is characterized by the spectrum of electrocardiographic and electrophysiological disorders involving the sinoatrial node and its connections with one or more of the following ECG changes: sinus bradycardia, junctional bradycardia, sinus arrest or pause, sinoatrial block, substitution rhythms etc. Children with SND may be completely asymptomatic or may experience weakness, pallor, presyncope/syncope, or HF. Symptomatic SND typically requires pacemaker implantation.^576 , 580 – 582^

The main causes of bradyarrhythmias in the pediatric population are given in Table 28 . In the pediatric population, bradyarrhythmias can trigger chest pain (including typical chest pain), fatigue, dyspnea, exercise intolerance, palpitations, dizziness, syncope, and HF during exertion.^577 , 578 , 582^

**Table 28:** Etiology of bradyarrhythmias in the pediatric population^575 , 578 , 583 – 585^

Sinus/Junctional bradycardia	
Origin	Causes
Adaptive	Highly trained athletes and individuals with increased vagal tone.
Respiratory	Hypoxia; apnea/bradycardia of prematurity.
Cardiac	Sinus node dysfunction (hereditary or secondary); CHD; atrial septal defect; after cardiac surgery/transcatheter repair.
Genetics	Progressive hereditary cardiac conduction disorders: SCN5A, TBX5, SCN1B-LOF, CASQ2, HCN4, etc.
Neurocardiogenic	Increased vagal tone; Bezold-Jarisch reflex; situational (cough, breath-holding attacks, sleep, etc.); esophageal, nasopharyngeal, peritoneal, or rectal stimulation.
Neurological	Increased intracranial pressure; Chiari malformation.
Psychiatric	Anorexia nervosa.
Endocrine	Hypothyroidism.
Pharmacological	Beta-blockers; alpha-2 agonists; fentanyl; phenylephrine; methoxamine.
Miscellaneous	Hypothermia. Hypoglycemia. Electrolyte abnormalities: hypo/hyperkalemia; hypo/hypercalcemia; hypomagnesemia.
**Third-degree AV block / Complete heart block**	
**Origin**	**Causes**
Cardiac	Congenital; CHD; genetic disorders; long QT syndrome; transposition of the great arteries; cardiac surgery; coronary artery disease.
Immune	Maternal connective tissue disease; systemic lupus erythematosus; Sjögren's syndrome.
Infectious	Myocarditis; endocarditis; Lyme disease; Chagas disease; diphtheria; rubella; mumps; trichinosis; Rocky Mountain spotted fever; HIV/AIDS; acute rheumatic fever.
Metabolic	Kearns-Sayre syndrome; carnitine deficiency; glycogen storage diseases.
Miscellaneous	Muscular dystrophy; eosinophilic cardiomyopathy; idiopathic.

Markers of high risk of morbidity and mortality in the pediatric population with bradyarrhythmias:^576 , 578 , 582^

– History of heart murmur or CHD.
– Syncope, especially if triggered by exertion, loud noises (startle), fear, or extreme emotional stress.
– Presyncope or syncope without premonitory symptoms or precipitating factors.
– Chest pain, palpitations, or dyspnea.
– Family history of SCD, long QT syndrome, sensorineural hearing loss, and pacemaker implantation.
– Taking medications that may cause bradycardia.

Particular features of ET/CPET in bradyarrhythmias and SND in the pediatric population:

– Provides information about the ability of the sinus node and AV node to respond to increased adrenergic activity in response to exertion.^43^
– Allows assessment of exercise-induced symptoms, the chronotropic response to exertion, associated arrhythmias, cardiorespiratory fitness, and risk stratification.^43 , 404 , 586^
– In patients with resting bradycardia, the finding of a normal chronotropic response helps rule out SND.
– Patients with complex CHD generally present with comorbid SND, chronotropic incompetence, and impaired CRF.^189 , 587 , 588^
– Patients with ASD generally present with chronotropic incompetence after transcatheter or surgical repair.^589 , 590^
– After ToF repair, chronotropic incompetence and severe sinus node dysfunction are common, occurring in ≈4% of patients.^591^
– After Fontain procedure, chronotropic incompetence occurs in up to 62% of patients and contributes to impaired CRF.^592 , 593^

#### 3.4. Indirect Metabolic Assessment

##### 3.4.1. VO_2_/Cardiorespiratory Fitness/Functional Classification

In ET, the indirect determination (estimate) of oxygen consumption (VO_2_) is considered the main metabolic assessment of effort. VO_2_is one of the main parameters of CHD severity, being relevant for risk stratification and prognosis. It is recommended to present VO_2_results in mL/kg/min (mL.kg^-1^.min^-1^is also acceptable). It can also be expressed through the metabolic equivalent of task - MET. Each 1 MET corresponds to 3.5 mL/kg/min of VO_2_.^1^

Maximum oxygen consumption (VO_2_max) expresses the greatest amount of oxygen extracted from the air inspired during the performance of ET considered maximal effort (examples: signs or symptoms of physical exhaustion; inability to continue the effort, etc.). In ET that do not have the characteristics of a maximum effort, the VO_2_obtained must be called VO_2_peak.^1^

Up to 12 years of age, there are no significant sex differences in VO_2_peak. After this age, male adolescents can reach VO_2_values up to 25-30% higher than those achieved by females.^22^

Cardiorespiratory fitness (CRF)/functional classification by ET/CPET involves stratification of physical performance based on oxygen consumption, or uptake (estimated by ET; measured directly by CPET). Maximal oxygen consumption (VO_2_max) expresses the highest amount of oxygen extracted from inspired air during dynamic exercise involving a large muscle mass.

To obtain the predicted VO_2_max, we suggest using reference tables specific for the pediatric age group (children and adolescents), based on sex, age and BMI. The use of specific reference tables in CHD and/or lung disease is also useful, and contributes to risk stratification in these conditions ( Appendix 4 ).

If equations are needed to estimate predicted VO_2_max in the pediatric population, the following are recommended:

1. For cycle ergometer, step protocol:^594^
  – Males: predicted VO_2_max = weight × (50.75 – 0.372 × age)
  – Females: predicted VO_2_max = (weight + 43) × (22.78 – 0.17 × age)
  Where: age is in years; height, in centimeters; weight, in kg. When the actual weight is greater than predicted for age and sex, the predicted weight should be used in the equations: predicted weight for sex: males = (0.79 × height) – 60.7; females = (0.65 × height) – 42.8.
2. For treadmill, incremental protocol:^594^
  predicted VO_2_max = (0.046 × height) – (0.021 × age) – (0.62 × sex) – 4.31
  Where: male = 0 and female = 1; age is in years; height, in centimeters.
3. For cycle ergometer, ramp protocol: Healthy children and adolescents aged 12 to 17 years:^595 , 596^
  – Males: predicted VO_2_max = (−0.297 × height^2^) + (105.9 × height) + (36.6 × body mass) – 8,660
  – Females: predicted VO_2_max = (−0.24 × height^2^) + (86.8 × height) + (14.7 × body mass) – 6,424
  Where: height is in centimeters and body mass in kilograms. If BMI is ≤ the 85^th^percentile for age, use actual body mass. If BMI is > the 85^th^percentile for age, use corrected body mass by estimating the body mass value corresponding to the 85^th^percentile for age.

Due to the great heterogeneity of the pediatric population, including across countries, it has not yet been possible to establish a unified classification of normality for VO_2_and cardiorespiratory fitness.^595^

In children with cardiomyopathy, CHD, HF, and VHD, a pre-test evaluation with one of the following scales is suggested to determine their functional status according to age group: modified Ross (children <6 years old) or NYHA (children >6 years old) – see Table 29 .

**Table 29:** Functional classifications based on clinical manifestations, by age group^379 , 793^

Class	Modified Ross (for children aged <6 years)	NYHA (for children aged >6 years)
I	No limitations or symptoms.	No limitations on physical activity.
II	Infants: mild tachypnea or sweating when feeding. Older children: dyspnea on exertion.	May experience fatigue, palpitations, dyspnea, or angina on moderate exertion, but not at rest.
III	Infants: tachypnea or profuse sweating when feeding Prolonged feeding times, failure to thrive. Older children: marked dyspnea on exertion.	Symptoms with minimal exertion. Marked limitation of physical activity.
IV	Symptoms such as tachypnea, retractions, grunting or sweating at rest.	Unable to perform any physical activity because of HF symptoms at rest, which worsen with even minimal exertion.

NYHA: New York Heart Association; HF: heart failure.

Table 30 presents a proposal for a national classification of cardiorespiratory fitness by VO_2_max and sex for Brazilian population aged 10 to 14 years.^597^ Table 31 describes the behavior of cardiorespiratory fitness in the most common CHD and cardiomyopathies in the pediatric population.

**Table 30:** Classification of cardiorespiratory fitness by VO_2_(mL/kg/min) measured directly in CPET for children aged 10 to 14 years

	Females	Males
Very poor	<33.0	<38.7
Poor	33.0-36.4	38.7-43.3
Fair	36.5-38.7	43.4-47.9
Good	38.8-42.4	48.0-52.2
Excellent	42.5	52.3

Adapted from: Rodrigues AN et al. Maximum oxygen uptake in adolescents as measured by cardiopulmonary exercise testing: a classification proposal.^597^

**Table 31:** Cardiorespiratory fitness in children and adolescents with common congenital heart diseases and cardiomyopathies^10 , 79 , 80 , 95^

Congenital heart disease/Cardiomyopathy	Cardiorespiratory fitness
ASD or VSD, small and unrepaired	Normal.
Large ASD, unrepaired	Slightly reduced.
ASD or VSD, repaired	Normal or slightly reduced.
ASD or VSD, large and repaired	Normal or slightly reduced.
Left ventricular outflow tract obstruction	Normal, except in severe cases.
ToF, repaired	Slightly to moderately reduced.
TGA, arterial switch	Normal or slightly reduced.
TGA, atrial switch	Moderately reduced.
PDA with PAH	Moderately to markedly reduced.
Eisenmenger syndrome	Markedly reduced.
Univentricular heart	Moderately to markedly reduced.
Status post Fontan procedure	Moderately to markedly reduced.
Congenital complete AV block	May be normal, or mildly to moderately reduced.
HCM	Mildly to markedly reduced.
Moderate to severe congenital AS	Mildly to markedly reduced.
Bicuspid aortic valve *	Mildly to markedly reduced.
Mitral valve prolapse	May be normal, or mildly to moderately reduced.

ASD: atrial septal defect; VSD: ventricular septal defect; ToF: tetralogy of Fallot; TGA: transposition of the great arteries; PDA: patent ductus arteriosus; PAH: pulmonary artery hypertension; HCM: hypertrophic cardiomyopathy; AV: atrioventricular; AS: aortic stenosis.

* With moderate/severe AS and associated aortic insufficiency or coarctation of the aorta.

### 4. Test Cessation Criteria (Clinical, Hemodynamic, and Electrocardiographic)

The main test cessation criteria for the pediatric population are given in Table 32 .^7 , 11 , 176 , 177^Test cessation may also be justified in other situations not described herein, but considered to pose a risk of serious complications; any such intercurrent events should be described in detail in the test report.

**Table 32:** ET/CPET cessation criteria for the pediatric population^7 , 11 , 176 , 177^

Parameter	Criteria
**Test objective met**	The diagnostic findings have been established and continued exercise will not provide additional relevant information.^7^
**Symptoms** *	The following signs and symptoms indicate that continued exertion may be detrimental to the patient's welfare: – Physical exhaustion. – Lower-limb muscle pain and/or exhaustion. – Lower-limb claudication (limiting), ataxia. – Persistent (limiting) vertigo, nausea, presyncope, syncope. – Increasing chest discomfort or chest pain with increasing work load (limiting), typical angina (moderate to severe). – Early dyspnea disproportionate to the intensity of exertion. – Intolerable feeling of tachycardia.
**Physical examination/cardiovascular and respiratory variables**	– Pallor (skin and mucous membranes), diaphoresis (profuse, disproportionate sweating), poor peripheral perfusion. – Tachypnea (disproportionate to exertion), bronchospasm, bilateral basal crackles. – Progressive, persistent decline in systolic blood pressure with increasing load. ** – Marked elevation of SBP (≥250 mmHg).^7 , 200 , 418^ *** – Elevation of DBP ≥125 mmHg. *** – Symptomatic desaturation (a decline of at least 10 percentage points in relation to resting saturation) or SpO_2_<85% regardless of symptoms.
**ECG findings**	– ST segment changes: depression (horizontal and downsloping) or elevation ≥0.3 mV (3.0 mm). – Nonsustained supraventricular tachycardia, symptomatic or with hemodynamic repercussions. – Sustained supraventricular tachycardia (≥30 seconds) even if asymptomatic or with no hemodynamic repercussions. – Exercise-induced paroxysmal atrial fibrillation or flutter. – Increased density and complexity of ventricular arrhythmias as the test progresses. – Nonsustained ventricular tachycardia (≥3 beats/<30 seconds) or any episode of polymorphic NSVT. – Sustained ventricular tachycardia (≥30 seconds). – Ventricular fibrillation. – 2^nd^or 3^rd^degree AV block. – QTc prolongation >500 ms. – Exercise-induced bundle branch block which cannot be distinguished from ventricular tachycardia. – Patients with ICDs (terminate test at 10 bpm below the defibrillator firing threshold). – Persistent drop in HR with increasing load, especially in the presence of symptoms of low cardiac output.
**Other**	– At the patient's request, regardless of the occurrence of any abnormal findings. – Failure or malfunction of the ECG monitoring/recording system. – Failure to adapt to and/or coordinate with the chosen ergometer.

SBP: systolic blood pressure; DBP: diastolic blood pressure; HR: heart rate; ICD: implantable cardioverter/defibrillator; SpO_2_: oxygen saturation by fingertip oximetry.

* Children, especially between the ages of 3 and 7, may have limited capacity (associated with the degree of cognitive development) to assess peripheral sensory changes resulting from exertion, the intensity of perceived exertion, and associated symptoms.

** SBP drops during exercise with values below resting SBP or initial rise in SBP followed by a drop in SBP ≥20 mmHg.

*** In apparently healthy children and adolescents experiencing no complications or symptoms during exertion. In children and adolescents with heart disease (including CHD), special attention to hemodynamic repercussions and symptoms is warranted, especially if SBP exceeds 200 mmHg or DBP exceeds 110mmHg.

### 5. ET Reporting

ET reports for children and adolescents must follow the exact same structure and minimum requirements recommended for adults, as given in the Brazilian Guideline for Exercise Testing in the Adult Population – 2024:^1^

1. Description of general ET data.
2. Observed, measured, and recorded data.
3. Descriptive report of the ET.
4. Conclusions.
5. ECG recordings.

Additionally, the following practices are recommended:

– Do not use pre- and post-test risk scores designed for the adult population in pediatric patients; these scores have not been validated and cannot be extrapolated to the pediatric population.
– Make note of any adjustments made to the ET protocol, test cessation criteria, and test variables due to patient characteristics such as underlying diseases, age, sex, BMI, body surface area, current medications, etc.
– Preferably, present reference ranges or values for all measured variables.
– When relevant and available, comment on any findings in relation to the patient's underlying diseases, including prognostic impact and risk.
– In case of serial ETs, comment on the progression of test findings over time if possible.

### 6. CPET in Children and Adolescents

#### 6.1. Metabolic, Ventilatory, and Gas-exchange Responses in Children and Adolescents

##### 6.1.1. Cell Metabolism, and Physiological and Hormonal Responses to Exercise

Children and adolescents have metabolic responses to exercise that are different from those observed in adults. Adenosine triphosphate (ATP) and phosphocreatine reserves are unrelated to age. Muscle glycogen levels at rest are lower in children, reaching adult levels by adolescence.^598 , 599^

Compared to adults, children have a smaller muscle mass, with differences in utilization of energy sources and metabolic/hormonal adaptations, such as a greater dependence on fat oxidation, resulting in greater mobilization of free fatty acids. The release of glycerol and increase in growth hormone in pre-adolescent children corroborate these findings.^600 , 601^

The immaturity of anaerobic metabolism (reduced glycolytic activity) in children is due to:^598 , 599 , 602^

– Differences in skeletal muscle fiber types, with a greater proportion of slow-twitch (type I) fibers than in untrained adults.
– Anaerobic lactic pathway for ATP resynthesis is generally reduced in young individuals during high-intensity exercise.
– In prepubertal children, there is reduced activity of the enzymes phosphofructokinase-1 and lactate dehydrogenase, with limited production of muscle lactate compared to adults.

Therefore, children and adolescents adapt well to prolonged, moderate-to-intense exercise, showing rapid recovery after exertion.^603 , 604^

In children, hormonal adaptations in energy expenditure during prolonged exercise are associated with a smaller reduction in insulin levels and an increase in catecholamines and glucagon. This response corresponds to less effective regulation of blood glucose levels and a greater risk of hypoglycemia.^598 , 605 , 606^

The pubertal growth spurt is characterized by release of hormones (i.e. somatotropin, insulin-like growth factors, and sex steroids) responsible for changes in body composition and an increase in lean body mass, resulting in improved fitness and physical performance, particularly for anaerobic exercise.^599 , 607 – 609^

##### 6.1.2. Pulmonary Ventilation, Expired Gas Analysis, Spirometry, and Derived Variables

The key CPET variables (metabolism, pulmonary ventilation, expired air gases, spirometry) and derived variables in the pediatric population, as well as their respective units and interpretations, are given in Table 33 . The differences in behavior, or response, of these variables to exercise between children and adults are described in Table 34 .^11 , 176 , 179 , 610^

**Table 33:** Key CPET variables and their respective interpretations^11 , 176 , 179 , 610 , 611^

CPET parameter	Acronym/abbreviation	Unit of measure	Interpretation
Oxygen consumption	VO_2_	mL/kg/min	Defined as the volume of O_2_extracted from the air inspired over a given period of time. Can be obtained by the Fick equation ( Figure 1 ).
Predicted maximum oxygen consumption	VO_2_max predicted	mL/kg/min	We suggest the use of predicted VO_2_max tables which have been compiled for each age group and sex, both in the apparently healthy population and in subjects with heart disease ( Appendix 4 ).
Peak oxygen consumption	VO_2_peak	mL/kg/min	Peak VO_2_level measured during an incremental exercise test. Can be expressed as % of predicted VO_2_max for age and sex. Values >20 mL/kg/min are considered normal. Values >80% of predicted denote adequate cardiorespiratory fitness.
Maximal oxygen consumption	VO_2_max	mL/kg/min	Plateau VO_2_reached despite increasing intensity of exertion (maximum effort). Can be expressed as % of predicted VO_2_max.
First ventilatory threshold or anaerobic threshold	VT1/AT	mL/kg/min or %VO_2_max predicted	VT1 corresponds to the value of VO_2_above which energy production relies increasingly on anaerobic metabolism. Can be expressed as % of predicted VO_2_max (normally occurs at >40% of VO_2_peak). Represents the point from which a disproportionate increase in VE and VCO_2_in relation to VO_2_occurs.
Minute ventilation	VE	L/min	Ventilation (based on tidal volume and respiratory frequency) during exertion. In healthy individuals, VE value is more than enough to maintain PaCO_2_under any workload. In heart failure, lung perfusion is altered and VE increases, which correlates with poor prognosis.
Ventilatory equivalent for oxygen	VE/VO_2_	-	Ventilatory equivalent of oxygen: the number of liters of air breathed for each 1 liter of O_2_absorbed.
Ventilatory equivalent for carbon dioxide	VE/VCO_2_	-	Ventilatory equivalent of carbon dioxide: the number of liters of air breathed to dispose of 1 liter of CO_2_. Normal values are generally <30.
Ventilatory efficiency (ventilation/CO_2_production)	VE/VCO_2_slope	-	During normal incremental exercise testing, VE correlates linearly with VCO_2_. The VE/VCO_2_slope in normal individuals is approximately 25 to 30. Also known as ventilatory efficiency, this parameter is increased in heart failure, pulmonary artery hypertension, and/or intrinsic lung diseases, and correlates with prognosis.
Oxygen uptake efficiency slope	OUES	L/min	Logarithmic relationship between VO_2_and VE during incremental exercise (VO_2_= a log10 VE + b, where a = OUES). The steeper the slope, the better the ventilatory efficiency. OUES depends on age and body surface area. It is best expressed as a function of body surface area or body weight/mass. An OUES/BSA ≥1,200 or OUES ≥35/body weight (kg) correlates with VO_2_peak >80% of predicted. OUES is decreased significantly in children with CHD and pulmonary vascular disease.
Oxygen pulse	OP or O_2_pulse	mL/kg/min/bpm	Obtained by dividing the VO_2_by the heart rate (VO_2_/HR). It reflects the amount of O_2_transported with each cardiac systole and is directly related to stroke volume, allowing assessment of LV function. The normal absolute O_2_pulse value is >80%.
Respiratory quotient	RQ	-	Also known as the respiratory exchange ratio (RER), this is the ratio between VCO_2_and VO_2_. It allows identification of exercise intensity and of which macronutrient is being consumed to generate energy: the RQ is generally >1.1 during a maximal exercise test.
Pulse oximetry	SpO_2_	%	Must remain >95% throughout the exercise test. A decline in oxyhemoglobin levels <90% indicates impaired ability to adequately increase alveolar-pulmonary capillary oxygen transfer in response to exertion. A decrease ≥4% is known as desaturation, and occurs more commonly in patients with impaired pulmonary diffusion. Other lung abnormalities, such as right-to-left shunts or ventilation-perfusion mismatch, can also result in exertional desaturation.
Work rate to oxygen consumption ratio	ΔVO_2_/ΔWR	mL/min/W	Reflects the capacity of the muscles to extract O_2_and generate ATP. A decline <10 mL/min/W on exertion or sharp flattening of the ΔVO_2_/ΔWR curve at a particular point during exercise suggests impaired O_2_transport (myocardial ischemia or ventricular dysfunction).
End-expiratory partial pressure of carbon dioxide	PETCO_2_	mmHg	Derived from measurement of FECO_2_, this parameter reflects the alveolar and arterial partial pressure of carbon dioxide (PaCO_2_). PETCO_2_measured at VT1 correlates with cardiac output and, in patients with chronic HF, reflects disease severity. During exertion, PETCO_2_increases by 3 to 8 mm and subsequently decreases slightly until maximum exertion. In the absence of lung disease, PETCO_2_ranges from 36 to 42 mmHg. Lower values are indicative ventilation/perfusion mismatch, reflecting the severity of cardiac or pulmonary disease and portending a worse prognosis. PETCO_2_<36 mmHg is found in CHD with right-to-left shunting, tachypneic ventilatory patterns, and in HF with a blunted cardiac output response to exercise.
Maximal voluntary ventilation	MVV	L/min	MVV is the maximum volume of air at rest mobilized during voluntary exertion in 1 minute. It can also be calculated as follows: in females = FEV1 × 35; in males = FEV1 × 40.
Ventilatory Reserve	VR	-	Reflects the relationship of MVV at rest to maximal VE during exercise. Values <30% suggest limited ventilatory capacity, and are useful in the differential diagnosis of dyspnea associated with HF and chronic respiratory diseases. Healthy children have a VR of at least 11 L/min or 20% to 40% of their MVV.
Forced expiratory volume in one second	FEV1	%	FEV1 is the volume of exhaled air measured in the first second during a forced vital capacity (FVC) maneuver, which corresponds to the volume obtained in a single maximum inspiration followed by maximal forced expiration. FEV1 is one of the most important variables in the diagnosis of obstructive ventilation disorders (exercise-induced asthma, exercise-induced bronchospasm, etc.).

**Table 34:** Comparison of cardiovascular, ventilatory, and metabolic CPET variables between children and adults, during any exertion, submaximal exercise, and maximal exercise^1 , 11 , 176 , 179 , 599 , 610^

Variable		Comparison vs. adults *	Submaximal exertion		Maximal exertion	
			Children	Adults	Children	Adults
**Cardiovascular**						
	VO_2_peak (ml/kg/min)	Higher	↑↑	↑	↑↑	↑
	HR peak (bpm)	Higher	↑↑	↑	↑↑	↑
	Stroke volume (ml/bpm)	Lower	↑	↑↑	↑	↑↑
	Cardiac output (l/min)	Lower	↑	↑↑	↑	↑↑
	Arteriovenous O_2_difference	Higher in submaximal exertion	↑↑	↑	↑	↑↑
	Systolic and diastolic blood pressure	Lower	↑	↑↑	↑	↑↑
**Pulmonary**						
	Respiratory rate (bpm)	Higher	↑↑	↑	↑↑	↑
	Tidal volume (L)	Lower	↑	↑↑	↑	↑↑
	VE peak (L/min)	Lower	↑	↑↑	↑	↑↑
	VE/VCO_2_ **	Higher	↑↑	↑	↑↑	↑
	VE/VO_2_ **	Higher	↑↑	↑	↑↑	↑
**Metabolic**						
	Fat oxidation	Higher	-	-	-	-
	Carbohydrate oxidation	Lower	-	-	-	-
	Peak blood lactate	Lower	-	-	-	-
	Glycolytic capacity	Lower	-	-	-	-
	Alactic capacity	Lower	-	-	-	-
	Lactate clearance	Same	-	-	-	-

↑ = increased;

↑↑ = markedly increased; HR: heart rate.

* Irrespective of the intensity of exertion.

** Ventilatory equivalents that determine ventilatory efficiency.

###### 6.1.2.1. Oxygen Consumption (VO_2_)

Assessment of cardiorespiratory fitness (CRF) through direct measurement of VO_2_peak or VO_2_max in a CPET is considered the main metabolic variable during exertion. The VO_2_at the ventilatory thresholds (particularly the first ventilatory threshold, VT1) has diagnostic and prognostic importance in children and adolescents. VO_2_at VT1 and VO_2_max are generally higher than those observed in adults.^1 , 177 , 286 , 594^

In a maximal test, CRF can be assessed by the VO_2_peak (mL/kg/min), considered to be within normal limits when ≥2 SD. In adolescents, adoption of 80% of predicted VO_2_max as the lower limit of normality is not recommended, as this value may be overestimated.

The anaerobic capacity of children is lower than that of adults, even when expressed per unit of total or lean body mass.

It is not always possible to assess cardiorespiratory fitness based on VO_2_peak in submaximal tests. Other CPET parameters, such as VT1 and OUES, can be used to provide a better indication of fitness.

Physical deconditioning is generally defined as reduced oxygen transport capacity by the cardiovascular system and/or reduced efficiency in peripheral oxygen extraction, leading to an early VT1. A VT1 at <50% of predicted VO_2_max is associated with physical deconditioning; at <40% of predicted, it generally denotes underlying disease with significant impairment of CRF.^1 , 177 , 286 , 594^

###### 6.1.2.2. Oxygen Pulse

The oxygen pulse (OP or O_2_pulse; OP = VO_2_/HR) is a noninvasive variable that reflects cardiac output. It is useful in the assessment of ventricular dysfunction, with or without associated ischemia. Under normal circumstances, OP increases with exertion due to the linear increase in HR and VO_2_, plateauing close to maximum effort.^1 , 177 , 286 , 594^

A decrease in OP (normal value: ≥2 SD) at submaximal loads suggests ventricular dysfunction, and is indicative of reduced stroke volume. When combined with a drop in ΔVO_2_/ΔWR, such a reduction indicates severe ventricular dysfunction, often of ischemic etiology.

During CPET, the combination of decreased OP (<2 SD of predicted) at peak exercise, early VT1 (<40-50% of predicted VO_2_max), decreased VO_2_peak, and rapid increase in HR may be associated with physical deconditioning.

###### 6.1.2.3. Respiratory Quotient (VCO_2_/VO_2_Ratio)

In the pediatric population, the respiratory quotient (RQ; also known as RER) at rest ranges from 0.70 to 0.85. During progressive exertion, once VT1 is crossed the VCO_2_increases disproportionately in relation to VO_2_, which translates into an increase in RQ due to changes in energy substrates. It is essential that RQ be evaluated at the point of VO_2_peak, as it continues to increase after cessation of exertion, including in the early recovery stage. Once the RQ is ≥1.1, the exercise test can be considered maximal.^1 , 177 , 286 , 594^

In pediatric populations, HRpeak and RQ at peak exertion (RQpeak) are recommended as objective criteria to assess the level of exertion achieved. The following are considered optimal:

– HR ≥180 beats/min (or at least at ≥95% of predicted HRmax) at VO_2_peak.
– RQ of at least 1.00 at VO_2_peak. This value represents the lower limit of normal for CPET performed on a conventional cycle ergometer.

RQ ≥1.00 at VO_2_peak denotes exclusive use of carbohydrate (glucose) as a source of energy, through predominantly anaerobic metabolism. RQ values <1.00 at VO_2_peak may indicate submaximal exertion or may be pathological, indicating conditions such as lung disease, decompensated cyanotic CHD, or glycogen storage diseases. In apparently healthy children and adolescents, RQ values will decrease within 2 to 3 minutes of recovery.^1 , 177 , 286 , 594^

###### 6.1.2.4. Oxygen Uptake Efficiency Slope (OUES)

The oxygen uptake efficiency slope (OUES) reflects a nonlinear relationship of the ventilatory response to exertion, corresponding to the absolute increase in VO_2_associated with increased VE. It expresses the efficiency of alveolar O_2_extraction in ventilated air. OUES values are best presented relative to body surface area, weight, or fat-free body mass.^612 – 621^ Appendix 4 provides information on OUES values/percentiles and predictive equations for the apparently healthy pediatric population.^622^

A Brazilian study involving healthy children and children with CHD suggested the use of weight-indexed OUES (OUES/Kg), and proposed OUES values >35 as indicative of normal functional capacity.^623^An international multicenter trial found cutoff points of 38.4 for boys and 31.0 for girls.^624^

Submaximal OUES correlates with VO_2_peak, VEpeak, and VO_2_at VT1, and is thus a valid measure for determination of CRF and risk stratification in submaximal tests.^613 , 625 , 626^

###### 6.1.2.5. Ventilatory Equivalents of Oxygen and Carbon Dioxide

During CPET, the ventilatory equivalents of O_2_(VE/VO_2_) and of CO_2_(VE/VCO_2_) indicate, respectively, the VE required to consume 1 L/min of O_2_and produce/dispose of 1 L/min of CO_2_. During progressive exertion, the VE/VO_2_ratio decreases up to VT1, at which point it progressively increases, with positive inflections at VT1 and VT2. The VE/VCO_2_ratio decreases up to VT2, then increases thereafter.

The ventilatory equivalents contribute to the assessment of cardiorespiratory efficiency, help identify ventilatory thresholds, and have diagnostic and prognostic value in pediatric patients with CHD, HF, and pulmonary artery hypertension. Cardiocirculatory conditions with low cardiac output are associated with a steeply sloping VE/VCO_2_curve. The VE/VO ratio_2_is usually elevated in pulmonic regurgitation and HF.

In a study of 700 apparently healthy patients (aged 5 to 18 years) with CHD, the slope of the VE/VCO curve_2_was significantly higher in patients with heart disease (greatest increase seen in patients with RV outflow tract obstruction). This study suggests a value of 29 as the cutoff for normality.^624 , 627^

###### 6.1.2.6. Other Considerations Regarding Ventilatory and Metabolic Parameters^1 , 177 , 286 , 594^

Minute ventilation (VE) increases with progressive exertion, in a manner dependent on the intensity of the effort exerted and the subject's physical fitness, and correlates with VO_2_and VCO_2_.

An elevated respiratory frequency (RF) may be indicative of a sedentary lifestyle or abnormalities in ventilatory mechanics. The normal RF in children is usually higher than in adults: ≈65 breaths/min in children aged 5 to 8 years and ≈50-55 breaths/min in children >11 years.

Compared to adults, children have a closer relationship between RF and tidal volume (VT), generally associated with reduced ventilation/perfusion. This phenomenon is commonly observed in some forms of cyanotic CHD.

Ventilatory limitation is traditionally defined as a ventilatory reserve (VR) <20% during exertion. Healthy children have a VR ≥11 L/min, or 20% to 40% of their maximum voluntary ventilation (MVV).^179 , 628^

VR prediction equations:

MVV=FEV1×35VR=MVV-VEmaxMVVx100

MVV: maximal voluntary ventilation

FEV_1_: forced expiratory volume in one second

VR: ventilatory reserve

VEmax: maximal exercise ventilation

VR contributes to the differential diagnosis between heart disease and lung disease. Low VR is characteristic of primary lung disease and obstructive pulmonary disease, while elevated VR occurs in cardiovascular conditions that limit physical performance.^629^

Generally, children with restrictive lung diseases have reduced exercise capacity (low VO_2_peak and low VO_2_at VT1) and increased tidal volume (50% of vital capacity and/or 80% of inspiratory capacity), with relatively low VR.^630^Any further increase in VE is due to an increase in RR. If there is ventilation limitation during exertion, SpO_2_decreases with increasing workload.^1 , 177 , 286 , 594^

In PAH, there is a marked reduction in ventilatory efficiency, with elevated VE/VO_2_and VE/VCO_2_ratios, indicating abnormal gas exchange in the lungs.^631^

PETO_2_and PETCO_2_reflect arterial gas tensions. A combination of low PETCO_2_and elevated PETO_2_and RQ is indicative of hyperventilation.

A ≥5% drop in SpO_2_during ET/CPET is defined as exercise-induced hypoxemia. A decline of at least 10 percentage points in relation to resting saturation plus symptoms or an SpO_2_<85% regardless of symptoms are test cessation criteria. Desaturation is considered serious when SpO_2_is <80% and accompanied by signs and symptoms of severe hypoxemia; this generally occurs in children with severe lung disease or HF.^260 , 594^

### 7. CPET Reporting in Children and Adolescents

CPET reports for children and adolescents must follow the same structure recommended for adults, as given in the Brazilian Guideline for Exercise Testing in the Adult Population – 2024.^1^

The CPET report must cover an overview of all main ergospirometric variables (hemodynamic, ventilatory, and metabolic), a description of any abnormalities that led to cessation of the test, and diagnostic and prognostic hypotheses.

The report must include:

– A description of the HR, BP, ECG behavior, VO_2_, and metabolic equivalents of task (MET) achieved, in relation to the predicted values for age and sex.
– A description of the first ventilatory or anaerobic threshold (VT1), standardized for body mass (expressed as a percentage of actual VO_2_peak reached and predicted VO_2_max) and in relation to HR and work load.
– Measured cardiorespiratory fitness and its repercussions, considering the indication for CPET and other test findings.
– When relevant, the normal (reference) values used for sex, age, weight, and BMI, as well as a note on the presence or absence of underlying diseases.

Note: the parameters listed above carry great diagnostic and prognostic relevance and can be used to inform the exercise prescription, particularly for CVR purposes.

## Part 3 – Particular Aspects of ET/CPET in Specific Clinical Conditions

### 1. Congenital and Acquired Heart Diseases

Among the most common indications for ET/CPET in children, adolescents and young adults is the clinical, hemodynamic, and electrocardiographic workup of patients with CHD, especially after partial or complete correction or repair of a congenital heart defect. Cardiorespiratory fitness may be poor in patients with complex CHD (even in those apparently asymptomatic), especially if PAH and chronic HF are present.^77 , 632 , 633^

Table 35 describes the behavior of key ET/CPET variables in the most prevalent CVD across the pediatric age group.

**Table 35:** Key ET/CPET variables and their behavior in common cardiovascular diseases in the pediatric population^6^

Variable	Interpretation	Cardiovascular diseases
HRmax	↓ in chronotropic incompetence	Corrected CHD, LQTS, heart transplant.
	↓ beta-blocker/antiarrhythmic therapy	CHD/CM with heart failure, arrhythmia.
Systolic blood pressure	↓ in ventricular dysfunction	CHD, CM, HCM, PAH.
	↑ in hypertensive response	Coarctation of the aorta, essential hypertension.
ECG	Exercise-induced arrhythmia	CHD, primary arrhythmias, third-degree (complete) AV block.
	Ischemic repolarization changes	Kawasaki disease, coronary anomalies (congenital or post-repair).
	Other exercise-induced changes	LQTS, Brugada syndrome, WPW.
VO_2_peak	↓ in cardiopulmonary dysfunction and/or physical deconditioning	CHD, CM, PAH, potential transplant recipients, third-degree (complete) AV block.
Oxygen pulse	↓ in ventricular dysfunction and myocardial ischemia	CHD, HF, TCPC, CM, Kawasaki disease, coronary and valve defects.
Oxygen saturation	↓ in lung disease, cardiac and/or pulmonary shunts	Cyanotic CHD, HF, bronchial asthma, pulmonary fibrosis.
VE/VO_2_and VE/VCO_2_	↑ in ventilatory inefficiency (ventilation/perfusion abnormalities)	CHD with heart failure or right-to-left shunt, corrected ToF, PAH.

↓ = decreased;

↑ = increased;

CHD: congenital heart disease; CM: cardiomyopathy; HRmax: maximal heart rate; ECG: electrocardiogram; LQTS: long QT syndrome; PAH: pulmonary arterial hypertension; TCPC: total cavopulmonary connection; VT1: ventilatory (anaerobic) threshold; VE: minute ventilation; VCO_2_: carbon dioxide production; VO_2_: oxygen consumption; WPW: Wolff-Parkinson-White syndrome. Adapted from: Massin MM. The role of exercise testing in pediatric cardiology.^6^

#### 1.1. Atrial Septal Defects

Most patients with atrial septal defects (ASD) remain asymptomatic throughout childhood, even in the presence of a major left-to-right shunt. There are five main types of ASD: ostium secundum, ostium primum, sinus venosus, coronary sinus defects, and patent foramen ovale. These defects will be treated as a single entity (DSA) for the purposes of this guideline because they share similar symptoms, behavior of variables during exercise, and interpretations of ET findings, depending on the predominance of the shunt (whether right-to-left or left-to-right), defect size, and presence of PAH and/or HF.^634 , 635^

Particular features of the resting ECG in pediatric patients with ASD:^370 , 388 , 636 , 637^

– In most patients, P wave amplitude and duration are within normal limits. In ostium secundum ASD, peaked P waves usually occur in lead II due to right atrial enlargement.
– In ostium secundum ASD and significant left-to-right shunts, PRi prolongation (1^st^degree AV block) and intraventricular conduction delay (RBBB pattern) may occur in association with RVH.
– After surgical repair of ostium secundum ASD, there is generally a decrease in P wave duration and dispersion, although not to the point of normality.
– After transcatheter repair, partial or complete regression of ECG abnormalities is observed in most patients.^638 , 639^
– After surgical repair of sinus venosus ASD, relatively high rates of sinus node dysfunction (6%) and atrial fibrillation (14%) are observed.^640^

Particular features of ET/CPET in uncorrected ASD:

– Children generally have preserved cardiorespiratory fitness.^641^
– Adolescents and young adults may experience reduced cardiorespiratory fitness, especially when symptomatic. In these patients, reductions of up to 60% in predicted VO_2_max are observed.^610^
– Asymptomatic patients (with no volume overload and normal RV function at rest) may develop a significant increase in afterload and/or exercise-induced RV dysfunction.^642^
– The VE/VCO_2_slope is generally normal. However, in patients with ASD and HF, RV dysfunction, PAH, and/or lung disease, the slope may increase due to ventilation/perfusion mismatch.^610 , 627^

Particular features seen in ET after ASD repair:

– In early surgical repair, normal cardiorespiratory fitness is observed within 6 months and maintained throughout adult life.^643 – 645^
– Manifestations of exercise-induced arrhythmias and/or exercise-induced dyspnea are rare and determine the severity of CHD.^646 , 647^
– Depressed chronotropic response (chronotropic incompetence) is more common after surgical repair than after transcatheter repair.^589 , 590^
– After surgical repair, aerobic capacity is generally reduced and right ventricular performance is significantly lowered.^648^
– Late surgical repair (i.e. in adolescence) and/or surgical repair once PAH is already established generally results in lower cardiorespiratory fitness and a higher incidence of exercise-induced atrial arrhythmias.^649 , 650^
– Exercise-induced arrhythmias in children after ASD repair are rare, but may manifest as sinus bradycardia, sinus tachycardia, supraventricular tachycardia, premature atrial contractions, premature ventricular contractions, sinus node dysfunction, atrioventricular block, atrial flutter, and atrial fibrillation.^651 – 654^
– Additional features are noted in Table 36 .

**Table 36:** Behavior of key ET/CPET variables in repaired and unrepaired atrial septal defects

ET/CPET parameters	Unrepaired ASD	Repaired ASD
Resting ECG	In general, increases in P wave duration and amplitude and in PRi and QRS duration (RBBB pattern) are observed.^655 , 656^AV block (first- and second-degree) rarely occurs.	– After surgical and transcatheter repair: reductions in P wave duration, P wave dispersion, PRi, QRS duration, and QT dispersion.^656 – 660 , 661^ – AV block in 2 to 4% of patients, including complete heart block.^662^ – Surgical repair often results in cardiac arrhythmias (early and late), which may be benign and/or significant (requiring pharmacotherapy).^663^
Exercise-induced symptoms	Rare in children, but, when present, are associated with severity of PAH and/or HF.^634^ More common in adolescents and adults; may compromise cardiorespiratory fitness.	– Symptoms generally improve. – Persistence depends on residual PAH and HF and/or partially corrected complex CHD.^664^
VO_2_max (mL/kg/min)	Normal in children unless there is physical deconditioning. Decreases depending on the severity of PAH and/or HF.	– Normal >6 months after repair. – Partial improvement in persistent physical deconditioning, PAH, and/or HF.^665 – 667^
HRmax	Normal. In the NKX2.5 mutation (rare), bradyarrhythmia may occur due to sinus node dysfunction and/or atrioventricular node dysfunction.^668^	– Generally normal. – Chronotropic incompetence is rare, occurring usually after surgical repair or in the NKX2.5 mutation.^589 , 651^
Exercise-induced arrhythmia	Rare in children. More common in adolescents and/or PAH.	– Reduced by early correction.^669^ – Persists more often if correction done in adolescence.^670^
Pulse oximetry (SpO_2_%)	Decreased in right-to-left shunt and/or PAH.	– Normal unless there is residual shunting with PAH.
Minute ventilation (VE, L/min)	Increased.	– Normal, unless there is PAH or HF.
Oxygen pulse (mL)	Normal; decreased in HF.	– Normal; decreased if HF persists.
VE/VO_2_	Increased.	– Normal, unless there is PAH or HF.
VE/VCO_2_	Increased.	– Normal, unless there is PAH or HF.
End-expiratory partial pressure of carbon dioxide (PETCO_2_, mmHg)	Normal; decreased in right-to-left shunt.	– Normal; decreased in residual right-to-left shunt.

CCHD: congenital heart disease; CM: cardiomyopathy; ECG: electrocardiogram; PAH: pulmonary arterial hypertension; HF: heart failure; RBBB: right bundle branch block; VE: minute ventilation; VCO_2_: carbon dioxide production; VO_2_: oxygen consumption. Adapted from: Amedro et al. *Atrial septal defect and exercise capacity: value of cardio-pulmonary exercise test in assessment and follow-up* .^610^

#### 1.2. Ventricular Septal Defect

Pre-test cardiac auscultation may allow detection of ventricular septal defects (VSD). Murmurs are typically described as holosystolic (pansystolic). Murmur grade depends on the flow velocity, with smaller defects producing louder murmurs and potentially even a thrill.^671^

Resting ECG generally reflects the degree of hemodynamic instability with VSD:^388 , 672 , 673^

– Normal ECG suggests a small, isolated VSD with a minor left-to-right shunt.
– LVH pattern with left atrial enlargement indicates a moderate-to-severe left-to-right shunt, but no PAH.
– A combined LVH/RVH pattern, with large, biphasic QRS complexes in the peripheral and middle precordial leads (Katz-Wachtel pattern), is often found in patients with a large VSD and variable degrees of PAH.
– In severe PAH (example: Eisenmenger syndrome), there is a predominance of RVH patterns, QRS axis deviation to the right, and evidence of right atrial enlargement.
– Approximately 10% of patients with VSD have RBBB (complete or incomplete).
– Even in patients with small VSDs, the risk of serious arrhythmia and sudden death is greater than in apparently healthy children.
– A minority of patients undergoing transcatheter repair of perimembranous VSD may develop RBBB, LAFB, and complete heart block.

Particular aspects of VSD in ET/CPET:

– Small VSDs in the pediatric population generally present with hemodynamically insignificant left-to-right shunting, including during exercise, and no significant impairment of functional capacity.^674^
– Children with patent or surgically repaired VSDs generally have normal cardiorespiratory fitness, despite slight impairment of the chronotropic response.^9 , 675^
– Young adults with small VSDs not repaired in childhood may develop poor cardiorespiratory fitness related to shunt size and biventricular dysfunction.^676 , 677^
– Surgical repair of major VSDs in the first 2 years of life reduces the risk of symptom persistence and development of cardiopulmonary abnormalities secondary to ventricular dysfunction and/or progressive pulmonary vascular disease.^678^
– Development of PAH before repair and/or persistence of PAH after repair reduces exercise tolerance and worsens quality of life.^679 , 680^
– In Eisenmenger syndrome, there is generally marked impairment of cardiorespiratory fitness and increased risk of sudden death.^681 , 682^
– Additional features are noted in Table 37 .

**Table 37:** Behavior of key ET/CPET variables in repaired and unrepaired VSD

ET/CPET parameters	Unrepaired VSD	Repaired VSD
Hemodynamic changes with increased risk of exercise-induced complications	Large left-right shunt; left ventricular enlargement with compromised LV function; aortic insufficiency; pulmonary vascular disease/PAH; Eisenmenger syndrome.	– Residual shunt; HF; aortic regurgitation; right or left ventricular outflow tract obstruction; persistence of PAH; Eisenmenger syndrome.
Resting ECG	Generally reflects the degree of hemodynamic abnormality due to VSD (see text).	– Transcatheter repair of perimembranous VSD rarely leads to RBBB, LAFB, and complete heart block.^683 , 684^ – RBBB related to RV dysfunction and LV diastolic dysfunction are common after surgical repair.^685 – 687^ – Ventricular arrhythmias are common and their prevalence increases with age at the time of repair and follow-up time.^688^ – Complete heart block is rare after surgical repair and frequent after transcatheter repair.
Exercise-induced symptoms	Small * VSDs without aortic insufficiency are usually asymptomatic.^675^ Large defects and those causing PAH, HF, and/or Eisenmenger syndrome are symptomatic.	– Generally asymptomatic once repaired. – Generally symptomatic in case of persistent PAH, HF, aortic insufficiency, RV or LV outflow tract obstruction, and/or Eisenmenger syndrome.
VO_2_max	Generally normal in small, patent VSDs. Reduced in large defects, PAH, and/or Eisenmenger syndrome.	– Generally normal once repaired. – Transcatheter repair in asymptomatic or minimally symptomatic adolescents prevents deterioration of cardiorespiratory fitness and promotes reverse LV remodeling.^689^ – Reduced in case of persistent PAH, HF, aortic insufficiency, RV or LV outflow tract obstruction, and/or Eisenmenger syndrome.
HRmax	There is usually slight impairment of the chronotropic response. Sinus node dysfunction requiring pacemaker placement occurs in 4% of patients.	– Generally normal after transcatheter repair. – Surgical repair may result in lower peak HR and chronotropic incompetence.^407^
Exercise-induced arrhythmia	Rare in small defects. Common in large defects, HF, PAH, and Eisenmenger syndrome.	– Reduced by early correction. – Common when complex arrhythmia occurs after transcatheter repair, in residual PAH, in persistent ventricular dysfunction, and in post-repair LBB.^683 , 690^
Pulse oximetry	Normal in small defects. Reduced in large defects, right-to-left shunt, Eisenmenger syndrome, and/or PAH.	– Normal, unless there is persistent PAH or Eisenmenger syndrome.
Anaerobic threshold (VT1)	Usually reduced.^691^	– Increases after repair and may become normal. – Remains reduced in case of persistent PAH, HF, aortic insufficiency, and/or Eisenmenger syndrome.
Minute ventilation (VE, L/min)	Usually reduced.	– May remain reduced even after repair due to post-sternotomy restriction of rib cage compliance, prolonged exposure of small airways to high pulmonary blood flow, and changes in the viscoelastic properties of the lung.^691^
VE/VO_2_	Increased in large defects and Eisenmenger syndrome.	– Returns to normal unless there is PAH or HF.
VE/VCO_2_	Increased in large defects and Eisenmenger syndrome.	– Returns to normal unless there is PAH or HF.

CHD: congenital heart disease; CM: cardiomyopathy; ECG: electrocardiogram; PAH: pulmonary arterial hypertension; HF: heart failure; LBBB: left bundle branch block; RBBB: right bundle branch block; VE: minute ventilation; VCO_2_: carbon dioxide production; VO_2_: oxygen consumption; WPW: Wolff-Parkinson-White syndrome.

* Small VSDs with left-to-right shunt <50%, no signs of LV volume overload, and normal pulmonary artery pressure.

#### 1.3. Patent Ductus Arteriosus

The clinical manifestations of patent ductus arteriosus (PDA) depend mainly on the amount of blood flow from the aorta to the pulmonary artery and whether secondary PAH is present.^692^

ET/CPET contributes to clinical follow-up and therapeutic decision-making in the various forms and presentations of PDA:^693 , 694^

– In "silent" (inaudible) and minor cases (slight left-to-right shunt with no hemodynamic repercussions), to confirm the patient is asymptomatic or elucidate any exercise-induced symptoms and ECG changes.
– In those with hemodynamic repercussions or PDA with mild/moderate PAH, every 12-24 months as part of serial clinical follow-up and to inform therapeutic decision-making.
– If there is PAH, exercise testing can be used to check for occurrence of desaturation in the lower limbs, which constitutes a disease severity criterion and may contraindicate PDA closure.^695 – 697^
– Adolescents and young adults with severe PDA (left heart enlargement, severe PAH, and contraindications for ductus closure) and/or Eisenmenger syndrome should undergo exercise testing every 6-12 months for optimization of HF and/or PAH therapy.
– In children and adolescents with significant PDA that has progressed to advanced HF, CPET is particularly helpful in ascertaining whether heart transplantation is indicated.
– After PDA correction to test for persistence of symptoms, residual PDA, residual PAH, and surgical complications, such as obstruction of the left pulmonary artery and coarctation of the aorta.
– For preparticipation physical assessment of patients with silent/small PDAs or who have undergone successful correction, with no PAH, and wish to engage in exercise and sports.^695^

The pre-test physical examination findings of patients with uncorrected PDA vary according to the size of the defect and its repercussions. Silent PDAs present with a normal physical examination.^698^

In PDA, the resting ECG:^370 , 388^

– Is usually normal in smaller shunts.
– In moderate-to-large shunts, sinus tachycardia or atrial fibrillation, left atrial overload, left ventricular hypertrophy, and ST-segment depression are generally observed.^698^
– In large defects with established PAH, often shows evidence of right atrial enlargement and biventricular hypertrophy.
– Sinus rhythm is the norm; first-degree AV block occurs in ≈10% of cases. Second-degree AV block, LBBB, and RBBB are only rarely observed.

Particular features of ET/CPET in children and adolescents with PDA:

– Silent cases are generally asymptomatic, with no hemodynamic or anatomical repercussions, normal lung function, and normal cardiorespiratory fitness. Rarely, these patients may present with exercise intolerance or exercise-induced reactive airway disease.^693^
– PDA with PAH is generally associated with significant impairment of aerobic capacity, a drop in oxygen saturation with exertion (generally >10%), reduction of VO_2_peak and VE/VCO_2_slope values correlating directly with the severity of PAH. The most common exercise-induced symptoms are dyspnea, chest pain, dizziness, and palpitations (ventricular arrhythmia).^197 , 377 , 699^SpO_2_must be monitored in the upper and lower extremities, including to confirm the occurrence of exercise-induced desaturation of the lower limbs.^695^
– After surgical correction, asymptomatic patients generally have a lower HRpeak than apparently healthy subjects. Chronotropic incompetence may occur in some patients.^700^
– Patients who are asymptomatic after PDA correction (transcatheter or surgical), with no evidence of structural heart disease (valvular heart disease, arrhythmia, or ventricular hypertrophy) or pulmonary disease, generally exhibit normal a blood pressure response and normal cardiorespiratory fitness.^700^
– When surgical correction is complicated by left vocal fold paralysis, patients may present with severe laryngeal stridor and exercise-induced laryngeal obstruction.^701 , 702^
– Extremely premature infants (gestational age <28 weeks or birth weight <1,000 g) who have undergone surgical correction may present with reduced lung function and cardiorespiratory fitness on CPET in adolescence.^701^

#### 1.4. Tetralogy of Fallot

As the name implies, classic tetralogy of Fallot (ToF) consists of a constellation of four defects: ventricular septal defect; pulmonic stenosis; RV hypertrophy; and an overriding aorta connected to both the left and right ventricles. There are also variant presentations, which include ToF with pulmonary atresia and pulmonary valve agenesis (absent pulmonary valve).^703 , 704^

The long-term consequences of repaired ToF are many and serious; regular monitoring is required.^705^The incidence of arrhythmic sudden cardiac death is estimated at 1 to 5%. The main associated factors are: QRS duration >180ms; LV systolic or diastolic dysfunction; ventriculectomy; LV end-diastolic pressure ≥12mmHg; history of supraventricular arrhythmia; NSVT; and inducible VT on EP study.^388 , 706 – 708^

ET/CPET plays a relevant role in follow-up, risk stratification, therapeutic decision-making and assessment of the impact of post-surgical complications (residual pulmonary insufficiency, aortic insufficiency, RV dilation and/or dysfunction, residual pulmonary artery stenosis, RV outflow tract obstruction, complex arrhythmias, HF).^100 . 709^

The pre-test physical examination in patients with repaired ToF is important for investigation to suspect residual anatomical lesions and allow evaluation of the potential risk of complications during ET/CPET.^370^

Particular features of the resting ECG in repaired ToF:^388 , 706 – 708 , 710^

– Right atrial enlargement is observed in ≈30 to 50% of patients.
– The most prevalent pattern is RBBB with or without LAFB. RBBB is generally asymptomatic and does not require intervention.^711 , 712^
– QRS duration >150ms is associated with RV dysfunction and significant pulmonary valve insufficiency in the late postoperative period.
– Supraventricular arrhythmias, including disturbances of SA conduction, atrial fibrillation, and atrial flutter, are found in one-third of patients.
– Ventricular arrhythmias, including NSVT, are common.

Particular features of ET/CPET in ToF ( Table 38 ):

**Table 38:** Behavior of key ET/CPET variables in repaired ToF and repercussions thereof^100 , 716^

ET/CPET parameters	Behavior	Interpretation/Repercussions
Postoperative complications with increased risk of adverse events during ET/CPET	Residual pulmonary insufficiency; aortic insufficiency; RV enlargement and/or dysfunction; residual pulmonary artery stenosis; RV outflow tract obstruction; complex arrhythmias; HF.^717^	– Associated with exercise-induced symptoms and risk of complications during ET/CPET: significant desaturation, hypotension, congestion/HF, complex arrhythmias, pre-syncope and syncope.
VO_2_max	VO_2_max and VO_2_at VT1 are usually reduced. * The average % of predicted VO2peak is 68±2.8% (95% CI: 62.3-74%).^716^ When the chronotropic response is normal, VO2max appears to be less reduced. More pronounced reductions are observed when there is persistent PAH, HF, aortic insufficiency, or RV outflow tract obstruction. Residual pulmonary insufficiency and age at repair influence the reduction in VO_2_max.^718^	– Cardiorespiratory fitness may be limited despite improvement in functional class (NYHA) after correction.^719^ – Males generally have worse cardiorespiratory fitness than girls.^720^ – RV and LV dysfunction are linearly related to the reduction in VO_2_max.^721^ – Low or borderline VO_2_peak is useful in risk-stratifying asymptomatic adolescents and young adults being considered for pulmonary valve replacement.^100^
HRmax	Generally lower than in healthy children; chronotropic incompetence is common. Severe sinus node dysfunction occurs in 4% of patients.^591^	– Normal chronotropic response is associated with greater cardiorespiratory fitness, regardless of RV systolic function and/or pulmonary insufficiency.^252 , 714^
Exercise-induced ventricular arrhythmias	Generally due to delayed repair and depressed right ventricular function.	– Associated with significant residual hemodynamic changes and an increased risk of cardiovascular events.
Pulse oximetry	Normal in small residual defects. Reduced in large defects, right-to-left shunt, Eisenmenger syndrome, and/or PAH and HF.	– Associated with higher risk of cardiovascular events and worse prognosis.
Oxygen pulse	Generally remains reduced but stable (in ≈85.3% of cases). Increases in ≈10.3% of patients and reduces further in ≈4.4%.^715 , 716^	– Maintenance of reduction is associated with lower RVEF and smaller biventricular systolic volumes.^722^
VE/VCO_2_slope	Usually slightly increased. Large increase if there is HF.	– This increase is associated with reduced cardiac output, PAH, and worse prognosis.
OUES	Usually only slightly abnormal, but with a significant reduction in VO_2_max. Lower OUES values are associated with ventricular dysfunction.^715^	– When normal, denotes reasonable submaximal exercise capacity.

CHD: congenital heart disease; CM: cardiomyopathy; ECG: electrocardiogram; PAH: pulmonary arterial hypertension; HF: heart failure; LBBB: left bundle branch block; RBBB: right bundle branch block; OUES: oxygen uptake efficiency slope; VE: minute ventilation; VCO_2_: carbon dioxide production; VO_2_: oxygen consumption.

* After complete surgical correction with good outcomes (no residual VSD, RV-pulmonary artery pressure gradient <20 mmHg).

– Patients who have undergone surgical correction with good outcomes (no residual VSD, RV-pulmonary artery pressure gradient <20 mmHg) are generally asymptomatic at rest.
– After complete surgical correction, there are generally no major physical limitations to activities of daily living. However, CPET often shows reduced VO_2_max and VO_2_at VT1 (anaerobic threshold).^713^
– Children and adolescents who maintain a normal chronotropic response have greater cardiorespiratory fitness and HR reserve, even when there is pulmonary insufficiency and RV systolic dysfunction at rest.^714^
– Adolescents present with reduced cardiorespiratory fitness relative to biventricular systolic volumes and LV end-diastolic volume indexed to body surface area. The OUES and peak oxygen pulse are also associated with biventricular stroke volumes.^715^
– After surgical correction, SBP in the upper limbs, central SBP, and the arterial stiffness index show normal responses during exercise.^591^
– In patients who are initially asymptomatic after correction of tetralogy of Fallot and develop severe pulmonic stenosis with decreased cardiorespiratory fitness, valve replacement should be considered.^81^
– Exercise-induced ventricular arrhythmias may occur, generally associated with late repair, RV dysfunction, and increased risk of cardiovascular events.

#### 1.5. Transposition of the Great Arteries

Transposition of the great arteries (TGA) is a severe cyanotic CHD. It is incompatible with life, requiring the presence of an intracardiac shunt (patent foramen ovale, atrial septal defect, or ventricular septal defect) and/or extracardiac shunt (persistent ductus arteriosus or bronchopulmonary collateral circulation).^723^

TGA can be classified as:^724 , 725^

– Simple, with no heart defects other than the shunt.
– Complex, with additional associated lesions. These may include obstruction of the LV outflow tract (≈25% of patients), anomalies of the mitral and tricuspid valves, and coronary artery anomalies; in patients with VSD (≈50%), pulmonic stenosis or atresia, overriding or straddling atrioventricular valve, or coarctation of the aorta may be observed.

TGA requires surgical treatment shortly after birth or within the first few months of life at the latest. Since the late 1980s, arterial switch operation (Jatene procedure) has been recommended instead of the atrial switch (Mustard/Senning) procedure. In cases of complex TGA, other surgical approaches may be necessary (i.e. Rastelli, Nikaidoh, etc.).^724 , 726 , 727^

Patients require long-term follow-up, as complications are frequent: reintervention in up to 25% (due to pulmonic stenosis, coronary artery obstruction, aortic root dilation, and/or aortic insufficiency); RV dysfunction; bradyarrhythmias and tachyarrhythmias; CAD; and sudden death.^726 , 728 , 729^

ET/CPET plays a relevant role in follow-up after TGA repair:^22 , 723 , 724 , 726 , 729 , 730^

– Routinely every 3-5 years as part of ongoing monitoring for asymptomatic myocardial ischemia, especially in patients undergoing arterial switch.
– Investigation of episodes of syncope and palpitations, which general resulting from arrhythmias secondary to myocardial ischemia, RV outflow tract obstruction, and/or LV dysfunction. Arrhythmias occur in 2.4 to 9.6% of these patients, and are associated with risk of sudden cardiac death.
– Investigation of changes in tolerance to activities of daily living or symptoms of chest pain on exertion, which are generally associated with decline in LV function, CAD, and pulmonary artery obstruction.
– For risk stratification, prognostic assessment, and medical clearance/prescription of cardiopulmonary rehabilitation.

Resting ECG findings will vary depending on the repair technique and the patient's symptoms. Sinus node dysfunction, junctional rhythm, atrioventricular conduction disorders, RV hypertrophy, axis deviation to the right, and Q waves in the right precordial leads are commonly seen after atrial switch procedures. After arterial switch, sinus rhythm is generally observed (91.1%); rarely, ectopic atrial rhythm (5.4%) or junctional rhythm (3.6%) may develop. There is usually no evidence of ischemia or ectopic beats.^724 , 731^

Particular features seen in ET/CPET after TGA repair:

– Regardless of the procedure adopted, patients generally present with some impairment of cardiorespiratory fitness (87.5±2.9% of predicted VO_2_peak).^21^However, even with slightly reduced fitness, patients are generally in NYHA functional class I.^723 , 728^
– Arterial switch is associated with better exercise tolerance compared to atrial switch.^723 , 732^
– Patients undergoing arterial switch and VSD repair or who have residual RV outflow obstruction present with greater impairment of cardiorespiratory fitness.^23 , 733^
– In the late follow-up of arterial switch operations, HRmax is usually normal or slightly decreased (HRmax: 92±2% of predicted).^21 . 23^Chronotropic incompetence is a late complication in ≈5 to 34% of patients. Sinus node dysfunction is usually secondary to involvement of the sinus node artery during balloon septostomy or even arterial switch itself.^728 , 733^
– In the late follow-up of arterial switch operations, SBP is generally normal at rest and during exercise. DBP is generally low at rest and at peak exertion.^731^
– In the late follow-up of arterial switch operations, a reduction in oxygen pulse is generally observed, with normal VO_2_peak (no impairment of cardiorespiratory fitness). The good correlation between OUES and VO_2_peak allows use of this parameter in patients who are unable to complete a maximal test.^626 , 734^
– In the late follow-up of atrial switch (Mustard or Senning operation), ventricular arrhythmia (at rest and exertional) is usually observed, as are: reductions in RV ejection fraction (in up to 84% of patients); reduction of oxygen pulse and earlier VT1; slow normalization of the oxygen pulse during recovery; and prolonged CO_2_retention with subsequent hyperpnea.^729 , 735 , 736^
– Serial exercise testing in the late follow-up of atrial switch operations shows a progressive reduction in VO_2_peak and oxygen pulse across childhood and adolescence, suggesting an inability to increase stroke volume.
– In the late follow-up of atrial switch operations, VO_2_peak and oxygen pulse remain relatively stable in young adulthood. However, when RV dysfunction increases, a rapid decline in oxygen pulse, worsening of exercise tolerance, arrhythmias, and clinical deterioration with HF are observed.^737 , 738^
– Onset of arrhythmias in the early postoperative period of atrial switch procedures represents a risk of arrhythmias as a late outcome (RR: 3.8; 95% CI: 1.5-9.5), as well as of development of HF (RR: 8.1; 95% CI: 2.2-30.7).^739^
– In the late follow-up of atrial switch operations, reduced HRpeak and chronotropic incompetence are commonly observed.^735 , 740^
– Regardless of the repair technique, exercise-induced ST-segment depression is rare, but if it occurs and meets criteria for myocardial ischemia, a thorough workup for CAD (generally asymptomatic, affecting 2 to 11.3% of patients) should be pursued.^741^

#### 1.6. Fontan Circulation

Fontan surgery is a palliative procedure for CHDs with a functionally univentricular heart, allowing near-normalization of arterial oxygen saturation and removal of chronic volume overload. The natural history of patients with a Fontan circulation is characterized by a progressive increase in peripheral vascular resistance (PVR), subsequent reduction in cardiac output, chronic venous hypertension, peripheral stasis, and lymphatic congestion. The main complications are cyanosis, exercise intolerance, HF, ascites, arrhythmias, liver dysfunction, protein-losing enteropathy (PLE), plastic bronchitis, and coagulation abnormalities. After a Fontan procedure, HF is common, progressive, and may be systolic, diastolic, or both. Contributing factors for the development of HF include diastolic ventricular dysfunction, increased pulmonary vascular resistance, atrial tachycardia, valve insufficiency, and shunting with volume overload.^742 – 744^

ET/CPET has many uses in monitoring patients with a Fontan circulation:^744^

– Quantification of cardiorespiratory fitness and elucidation of exercise-limiting factors.
– Assessment of respiratory reserve, ventilation/perfusion, SpO_2_, chronotropic response, and arrhythmias, which contribute to exercise intolerance and late complications.
– Optimization of therapy, which includes ascertaining whether closure of fenestration (due to excess systemic hypoxia) and pacemaker implantation (due to sinus node dysfunction/severe chronotropic incompetence) are indicated.^745 , 746^
– Selection of candidates for heart transplantation.
– For risk stratification, prognostic assessment, and medical clearance/prescription of cardiopulmonary rehabilitation.^747 – 750^
– As part of an American Heart Association-recommended intensive surveillance strategy for adolescents, with repeat testing every 1-3 years due to the high risk of HF and of death.^744^

Particular features of ET/CPET in patients with a Fontan circulation:

– In patients with HF and/or low SpO_2_at rest, it is recommended that testing be carried out in a hospital setting, with special measures in place (adapted protocol/work load, SpO_2_monitoring, etc.).
– Cardiorespiratory fitness is largely reduced, with VO_2_max reaching ≈60 to 65% of predicted.^92 , 747 , 751^
– Cardiorespiratory fitness in patients with Fontan circulation can be classified according to the % of predicted VO_2_actually achieved: severely impaired if <50%; moderately impaired, 50 to 60%; slightly impaired, 60 to 80%; borderline, 80 to 90%; normal if >90%.^752^
– Low SpO_2_at rest is common, with levels often <90%. SpO_2_during exercise generally drops below 90% due to decompensation of cyanosis control mechanisms and increased venous return of desaturated blood.^588^
– Children and adolescents commonly present with chronotropic incompetence and reduced HR reserve during exertion. The type of palliative procedure, dominant ventricle subtype, and/or underlying cardiac anatomy all affect the degree of chronotropic incompetence. Generally, HR behavior during recovery is normal.^753 , 754^
– Resting SBP remains unchanged, while DBP increases significantly postoperatively. During exercise, SBP and DBP responses are normal and consistent with the work load, generally reaching >85% of predicted SBP for age.^755^
– Increased P wave duration and dispersion on resting ECG are associated with risk of sustained atrial tachyarrhythmias (including atrial fibrillation and intra-atrial reentrant tachycardia), which affect 9.4 to 20% of patients.^756 , 757^
– PVCs are rare and may be due to worsening ventricular function or secondary to electrolyte disorders/medication use. Around 3% to 12% of patients develop VT as a late postoperative complication.^758 , 759^
– Exercise-induced arrhythmias are rare and generally disappear with cessation of exertion.^751^
– The resting ECG usually shows a pattern consistent with LVH, ventricular overload, and significant ST-segment depression (>1.0mm). This ST-segment depression frequently becomes more pronounced with exertion, but is not associated with CAD.^520 , 760^
– Reductions in oxygen pulse, VT1, pulmonary ventilation, and respiratory quotient (RQ), as well as chronotropic incompetence, are the norm (observed in up to 62% of patients). These changes, associated with impaired systolic ventricular function, correlate with worse functional capacity.^588 , 592 , 593 , 761^
– Reduced cardiac reserve, VO_2_peak, OUES, and chronotropic incompetence identify patients at increased risk of death and need for heart transplantation.^91 , 748 , 749 , 762 , 763^
– In adolescents, exercise oscillatory ventilation (EOV) is associated with an increased risk of death/transplantation (RR: 3.9; 95% CI: 1.5-10.0).^764^
– In adolescents, the following were markers of risk of hospitalization within 2 years (due to HF, arrhythmia, etc. RR: 7.645; 95% CI: 2.317-25.230); VE/VCO_2_slope ≥37 (RR: 10.777; 95% CI: 1.378-84.259).^765^

#### 1.7. Hypertrophic Cardiomyopathy

Hypertrophic cardiomyopathy (HCM) is a genetic disease with an autosomal dominant inheritance pattern (incomplete penetrance and variable expressivity) characterized by hypertrophied, disorganized myocytes separated by areas of interstitial fibrosis. Cardiac hypertrophy is generally asymmetric, most commonly involving the basal part of the interventricular septum, underlying the aortic valve. Occasionally, it is restricted to other cardiac regions, such as the apex, midportion, and posterior wall of the LV. HCM can be classified as primary, if caused by sarcomere gene mutations, or secondary, if associated with a non-sarcomeric cause.^766 , 767^

The average age at onset is 8.9 years, and there is a male predominance. The risk of SCD in pediatric patients is ≈1 to 7% per year. In adolescents with a family history of SCD the average time after diagnosis to first major cardiac event (including death/SCD) or cardiac intervention (myectomy and/or ICD) is ≈18 months.^768 – 770^

Symptoms generally result from four pathophysiological conditions: diastolic ventricular dysfunction, LVOT obstruction, myocardial ischemia, and cardiac arrhythmias.^770^

In this context, ET/CPET is useful for risk stratification and optimization of clinical management, especially in children >7 years, who are at higher risk. Approximately one-third of patients with HCM have LVOT obstruction at rest, which worsens with exertion. Another one-third have exercise-induced obstruction, and the remaining third have LVH without obstruction (at rest or exercise-induced).^771^

In patients with LVOT obstruction, a harsh midsystolic murmur (grade 3-4/6, loudest between the apex and left sternal border) is usually audible. The murmur increases in intensity when LV volume decreases during a Valsalva maneuver, when assuming an upright position, and during and immediately after exertion.^771^

Particular features of the resting ECG in HCM:^708 , 710^

– Abnormal in 75-95% of patients, even when there is little or no LVOT obstruction.
– Evidence of left atrial enlargement.
– The most common abnormalities are the characteristic LVH pattern, deep Q waves, ST segment depression, and T wave changes.
– 2 to 5% of patients exhibit pre-excitation ECG findings, and may present with AV nodal supraventricular arrhythmias and Wolff-Parkinson-White syndrome.^768^

Particular features of ET/CPET in HCM:

– Informs the decision on whether to escalate therapy, especially if symptoms are unclear based on clinical history alone.
– Generally shows poor cardiorespiratory fitness.
– Patients with severe LVOT obstruction generally present with high ventricular diastolic pressure and exercise-induced dyspnea. In the most serious cases, frank acute heart failure may develop.
– Syncope, whether exercise-induced or at the onset of recovery, results from severe LVOT obstruction, with or without associated ventricular arrhythmia.
– Ischemic chest pain is common, and may or may not be typical (anginal).
– Abnormal BP response to exercise, characterized by an increase in SBP <25 mmHg or a drop >10 mmHg, is associated with increased risk of SCD.^154 , 438 , 773^
– Abnormal ET findings are associated with a higher risk of all-cause mortality and/or transplantation: ischemic response (RR: 4.86; 95% CI: 1.69-13.99) and depressed BP response (RR: 3.19; 95% CI: 1.32-7.71). Exercise-induced ischemia is also independently associated with SCD (RR: 3.32; 95% CI: 1.27-8.70).^157^
– Exercise-induced supraventricular and ventricular ectopics are frequent, and NSVT can occur in up to 20-30% of patients.
– Exercise-induced arrhythmia (atrial and/or ventricular), irrespective of density, is associated with an increased risk of heart transplantation, ICD implantation, and SCD (RR: 5.8; 95% CI: 1.3-26.7).^157 , 773^
– Atrial fibrillation occurs in ≈25% of patients with HCM, is poorly tolerated, and is often the culprit behind exertional HF symptoms.
– Cardiorespiratory fitness is poor (VO_2_peak generally <80% of predicted) and correlates with diastolic dysfunction on echocardiography.^207^
– CPET measures cardiorespiratory fitness directly and is relevant in the assessment of patients with severe symptoms, particularly to ascertain whether heart transplantation is indicated.^774^A reduction in VO_2_peak <50% of predicted values for age and sex should be considered in the heart transplant selection process.^775^
– VO_2_peak, oxygen pulse, and HRpeak are generally reduced and worsen gradually over time.^207^A VO_2_peak ≤60% of predicted is a marker of HF and SCD risk.^770^

#### 1.8. Kawasaki Disease

Kawasaki disease (KD) is an acute systemic vasculitis that mainly affects male children (M:F ratio ≈1.5:1) under the age of 5. It is the leading cause of acquired CAD in children, and is most common in Japan.^37 , 776^

The most feared complication of acute KD is the development of vascular abnormalities in small- to medium-sized arteries (mainly in the heart), characterized by three interconnected processes: necrotizing arteritis, subacute/chronic vasculitis, and luminal myofibroblastic proliferation. CAD can develop during the healing phase of the acute episode or later on in the course of the disease. Even children with KD who have no overt coronary lesions exhibit lower coronary flow reserve and greater total coronary resistance.^777 , 778^

The risk of developing coronary artery aneurysms (CAA) is ≈25% in untreated cases and 5% in adequately treated cases. CAAs may initially manifest as enlargement and progress to moderate dilation (5 to 8 mm in diameter), or may even develop as large aneurysms (>8 mm). CAAs are classified by comparing the diameters of coronary arteries indexed in units of standard deviation from the mean by body surface area (Z-score). This classification is recommended for the left main coronary artery, anterior descending artery, and right coronary artery. CAA Z-score define aneurysms as: absent if the score is <2; isolated dilation if 2 to <2.5; small if ≥2.5 to <5.0; medium if ≥5.0 to <10.0 and absolute dimension <8 mm; and large or giant if ≥10.0 (or absolute dimension ≥8 mm). Large and/or giant aneurysms do not regress spontaneously, rarely ever rupture, and almost always contain thrombi, which may calcify or become occlusive.^37 , 776^

Children with CAAs may progress to late-stage KD, with thrombosis, ischemic heart disease, myocardial infarction, and sudden death (≈0.2 to 0.8% in the first 10 years after KD). The most common late complications of KD are ischemic heart disease (4.6 events/1,000 person-years) and ventricular arrhythmias (4.5/1,000 person-years). Patients in late-stage KD require regular monitoring and adoption of specific protocols for risk stratification and prevention of complications; ET/CPET is particularly useful in this context.^779 – 781^

The resting ECG varies depending on the complications resulting from the acute stage of KD. In patients who develop CAAs or MI in the acute stage, pathological Q waves and ST-segment/T wave changes associated with areas of ischemia and/or necrosis are common. QTi dispersion (QTd) should be assessed; when abnormal, it is associated with coronary artery sequelae and a greater risk of ventricular arrhythmia during follow-up.^487 , 782 , 783^

Main indications for ET/CPET in KD:^18 , 37^

– In late-stage disease, to investigate symptoms suggestive of ischemia (Class of Recommendation: I; Level of Evidence: C).
– In the pediatric population, ET/CPET should not be relied on alone for investigation of exercise-induced myocardial ischemia. In such cases, combination with an imaging method is recommended.
– In patients with CAA and suspected ischemic events, exercise-induced symptoms, or low exercise tolerance (Class of Recommendation: I; Level of Evidence: C).
– In patients with CAA seeking to pursue competitive sports or high-intensity exercise, for preparticipation physical assessment, to detect exercise-induced arrhythmias (Class of Recommendation: IIa; Level of Evidence: C).
– In the monitoring of children and adolescents who have undergone myocardial revascularization (surgical and/or percutaneous), for assessment of cardiorespiratory fitness, optimization of therapy, and detection of CAD progression/restenosis.^784^
– For risk stratification, prognostic assessment, and medical clearance/prescription of cardiopulmonary rehabilitation (Class of Recommendation: I; Level of Evidence: B).

Particular features of ET/CPET in KD:

– In symptomatic patients, helps inform the decision to pursue revascularization. Exercise-induced arrhythmias and/or low exercise tolerance (<3 METs) in the presence of symptoms (angina and dyspnea) are considered poor prognostic factors.^18 , 37^
– Patients in late-stage KD with moderate to severe CAD may exhibit sinus node dysfunction and atrioventricular conduction disorders.^785^
– Patients in late-stage KD with CAA z-scores ≥2.0 in the proximal ADA or RCA generally exhibit a reduction in METs achieved proportional to the z-score, lower levels of cardiorespiratory fitness, RER, SBPmax and maximum double-product, when compared to patients with CAA z-scores <2.0.^15 , 786^Reduced cardiorespiratory fitness is generally more severe in adolescents with KD.^16 , 17^
– Patients in late-stage KD with CAAs but no myocardial perfusion defects have HR, SBP, and DBP responses to exercise similar to those of patients without CAA. However, patients with CAAs and myocardial perfusion defects usually exhibit a lower HR in the 1^st^minute of recovery and lower DBP in the 1^st^and 5^th^minutes of recovery, which are findings associated with worse prognosis.^398^
– Exercise-induced ST depression is common in late-stage KD, with low sensitivity and high specificity for obstructive coronary lesions.^18 , 787^
– Patients in late-stage KD rarely exhibit exercise-induced ventricular arrhythmias (associated with CAA z-score ≥5). Complex ventricular arrhythmias and exercise-induced ventricular tachycardia are associated with large CAAs, previous VT, ICD placement, CAD, status post MI (generally >10 years after MI) and status post CABG.^560 , 788^
– QTi dispersion during exercise is generally altered in late-stage KD, regardless of QTd at rest or coronary sequelae. This phenomenon poses a risk of developing exercise-induced arrhythmias.^789^

### 2. Heart Failure and Heart Transplantation

As of 2017, the prevalence of heart failure (HF) in the 5-14 age group in Brazil was 34.1/100,000 children.^790^In the pediatric population with CHD, this prevalence ranges from 6.2% to 39%. HF in the pediatric population is associated with high morbidity, and in-hospital mortality rates ranging from 7-26%.^791 , 792^

The main causes of HF in the pediatric population are given in Table 39 . The clinical presentation of HF is related to age: infants and young children present with difficulty feeding, cyanosis, tachypnea, sinus tachycardia, and diaphoresis; older children and adolescents have fatigue, shortness of breath, tachypnea, exercise intolerance, abdominal pain, oliguria, and edema of the lower limbs. The severity of HF should be classified according to age group, using the modified Ross (children <6 years old) or NYHA (children >6 years old) classifications (see Table 29 ).^379 , 793^

**Table 39:** Leading causes of heart failure in the pediatric population^794 , 795^

Broad class of etiology	Examples
**Genetic mutations**	Lamin A-C, myosin-binding protein C, troponin I, taffazin (Barth syndrome), dystrophin, LAMP2 (Danon disease), mitochondrial disorders, titin, desmin.
**Myocarditis**	Enterovirus, parvovirus, adenovirus, influenza, Epstein-Barr virus, human immunodeficiency virus, cytomegalovirus, varicella zoster virus (chickenpox), mumps, giant cell arteritis, Lyme disease, mycoplasma, Chagas disease.
**Ischemia**	Anomalous origin of coronary artery, Kawasaki disease with coronary aneurysms.
**Metabolic disorders**	Fatty acid oxidation disorders, glycogen storage disorders (i.e. Pompe disease), carnitine deficiency.
**Structural heart disease**	Valvular heart disease, congenital heart disease.
**Endocrine disorders**	Hypothyroidism, thyrotoxicosis, pheochromocytoma, glycogen storage diseases.
**Blood disorders**	Iron deficiency, sickle cell anemia, hemochromatosis, thalassemia.
**Autoimmune diseases**	Systemic lupus erythematosus, dermatomyositis, rheumatic heart disease.
**Exposure to cardiotoxic agents**	Anthracyclines, cyclophosphamide, radiation.

Right HF is not common in children, but may be associated with CHD including tetralogy of Fallot, TGA, ASD, Ebstein anomaly, arrhythmogenic RV cardiomyopathy, and ventricular dysfunction in the functionally univentricular heart. The two leading causes of end-stage HF in the pediatric population are cardiomyopathies and CHD, each accounting for approximately half of cases requiring heart transplantation (HTx). HTx in the pediatric population represents 13% of all transplants, and more than 60% of recipients survive for at least 10 years.^191 , 796 , 797^

Particular features of ET/CPET in pediatric patients with HF:^182 , 797 – 800^

– Assessment of cardiorespiratory fitness and of the behavior of CPET variables provide objective information on the functional status of the heart, lungs, and peripheral muscles, elucidating the natural history of HF and informing therapeutic decision-making.^6^
– CPET should be part of the workup of patients aged ≥6-8 years with cardiomyopathy and HF (Class of Recommendation: IIa; Level of Evidence: C).
– CPET should be used to ascertain the cause of cardiorespiratory limitations to exercise in patients with symptoms of HF (Class of Recommendation: IIa; Level of Evidence: C).
– In patients with stage C HF, the combination of VO_2_peak <50% of predicted and severe exercise limitation constitute the basis for a possible indication of HTx (Class of Recommendation: IIa; Level of Evidence: C).
– Preparticipation medical assessment and risk stratification before a physical training/cardiorespiratory rehabilitation program (Class of Recommendation: I; Level of Evidence: C).
– In patients with a circulatory assist device and/or after HTx for assessment of cardiorespiratory fitness, risk stratification, serial graft evaluation, and prescription of a physical activity program (including rehabilitation and physical education in school) (Class of Recommendation: IIa; Level of Evidence: C).
– In patients with suspected cardiotoxicity secondary to chemotherapy/radiotherapy, in the differential diagnosis of dyspnea, screening for cardiac dysfunction (including subclinical), risk stratification, optimization of therapy, and medical clearance/prescription of physical exercise and rehabilitation.^801^

The resting ECG in HF is nonspecific but often abnormal. LV hypertrophy, RV and/or LV overload, and ST-segment and/or T wave changes may be seen. Rhythm disturbances are common, including sinus tachycardia, supraventricular tachycardia, atrial fibrillation/flutter, atrioventricular block, and VT. Intraventricular conduction disorders or QTc prolongation are generally associated with ventricular dysfunction, HF, and structural heart disease (CHD or advanced cardiomyopathy).^559 , 802^In idiopathic cardiomyopathy, the presence of LBBB and left atrial enlargement correlates with increased risk of mortality.^379 , 803^

Particular features of ET/CPET in HF:

– Usually performed with the patient on their usual medications, including antiarrhythmics and beta-blockers. Discontinuation may trigger clinical deterioration and increase the risk of complications during the test. Patients on antiarrhythmic agents have more severe disease and are at a higher risk of SCD during 5-year follow-up (RR: 3.0; 95% CI: 1.1-8.3).^803^
– Children with HF secondary to idiopathic dilated cardiomyopathy present with the following at VT1 and at peak exercise: significantly lower values of SBP, VT, VO_2_, VCO_2_, and minute ventilation; increased VE/VO_2_and VE/CO_2_values; abnormal oxygen pulse; and a significantly greater VE/VCO_2_slope at peak exertion. CPET variables allow quantification of the reduction in cardiorespiratory fitness and possible mechanisms of exercise limitation.^804^
– Serial CPET in children with dilated cardiomyopathy demonstrated an increased risk of hospitalization due to decompensated HF, circulatory assist/HTx, and death in those who experienced a 10mmHg reduction in SBPpeak (RR: 1.41; 95% CI: 1.12-1.79) or a 10% reduction in VO_2_peak from predicted (RR: 1.59; 95% CI: 1.16-2.17).^805^
– A VO_2_peak <44% of predicted in children with biventricular circulation was associated with higher risk of death or deterioration of HF (RR: 5.1; 95% CI: 1.9-13.5).^806^
– Clinicians should always remain vigilant for the possibility of acute exercise-induced decompensated HF, which, although rare, requires immediate intervention/critical care.^800^
– At late (10-year) follow-up of anthracycline chemotherapy (cumulative dose >300mg/m2), ≈32% of patients had developed compromised cardiorespiratory fitness (VO_2_max <80% of predicted) and subclinical cardiac dysfunction.^798^

Particular features of ET/CPET in the pediatric population requiring or undergoing HTx:

– When evaluating HTx recipients, it is suggested that variables (VO_2_, HRpeak, work load, etc.) be converted into percentages predicted for age, sex, and/or weight, so as to allow longitudinal comparisons with the patient's own serial tests, as well as with data available in the literature.
– VO_2_peak ≤62% of predicted in patients with HF is strongly associated with risk of HTx and death within 2 years (RR: 10.78; 95% CI: 4.04-27.98).^807^
– Children with biventricular circulation are at risk of death, requiring circulatory support, and urgent HTx when VO_2_peak is <50% of predicted (RR: 4.7; 95% CI: 1.8-12.3) and VE/VCO_2_slope is ≥34 (RR: 3.2; 95% CI: 1.2-8.4).^806^
– CPET is part of the detailed workup necessary for the indication of HTx, where VO_2_peak <50% of predicted should be considered a Class I indication.^808^Other indications include VO_2_<14 ml/kg/min (off beta-blockers) or VO_2_<12 ml/kg/min (on beta-blockers).^809^
– After HTx, impairment of cardiorespiratory fitness is generally observed, both in the immediate postoperative period and 3 to 6 years after transplantation, but tends to remain stable. The younger the patient at the time of transplantation, the higher the VO_2_peak values achieved. A decreased maximum work load (Wmax) (<75% of predicted value) is often observed. The serial response of HR (rest, peak, and chronotropic reserve), SBP, and VO_2_peak provide information regarding reinnervation (generally remaining stable or increasing) and graft outcomes; a progressive reduction in VO_2_peak is associated with graft loss due to vasculopathy.^810 , 811^
– HR in HTx recipients is generally higher at rest and lower at peak exertion (ranging from 66-86% of predicted HRmax). HR in the 1^st^and 3^rd^minutes of recovery is reduced in patients with persistent denervation.^191^
– On average, 57% of recipients show evidence of autonomic reinnervation (predominantly sympathetic), which is associated with better cardiorespiratory fitness, greater survival, and graft stability. Patients with autonomic denervation generally develop chronotropic incompetence. Deconditioning and side effects of immunosuppression can also affect cardiorespiratory fitness.^182 , 191 , 409 , 811^
– CPET performed in the immediate postoperative period of HTx (1^st^month) demonstrated reduced VO_2_at VT1 and at peak effort (both with values below predicted) in a case series.^192^
– Serial CPET in the late postoperative follow-up of HTx demonstrated that a VO_2_of 59.3% of predicted and an HRmax 75.8% of predicted were achieved at ≈3 years, and remained below normal in subsequent testing (≈5 years).^810^
– Behavior of other CPET variables in HTx recipients: VEpeak is generally reduced, as is Wmax (ranging from 60-66%); peak aerobic power was on average 56±14% of predicted.^191^

### 3. Cardiac Arrhythmia

#### 3.1. Congenital Long QT Syndrome

Congenital long QT syndrome (LQTS) is a genetic disease characterized by prolongation of the QTc interval (QTc >440 ms in males and QTc >460 ms in females), with a prevalence of 1:2,000 to 1:5,000. It can cause syncope, ventricular arrhythmias, and cardiac arrest. The average age at presentation is 14 years, with an annual rate of sudden cardiac death between 0.33% and 0.9%. LQTS should be investigated in children and adolescents with this clinical picture, a family history of sudden death, and/or a diagnosis of long QT. In type 1 LQTS, the leading trigger of arrhythmias is physical exercise. The Schwartz criteria are recommended for the diagnosis of LQTS in pediatric and adult populations.^812 – 814^

It is recommended that the QT interval be measured in leads II and V5.^82^QTi prolongation on resting ECG is the most common way of diagnosing this syndrome. However, 20-25% of patients with confirmed LQTS have a normal QTc interval at rest.^6 . 308^The ideal formula for adjusting QTc in ET remains controversial (see section "3.3.2.8. QT Interval" of this guideline). Interpretation of the QTc depends on the formula used.^124 , 542 – 544^ Table 24 gives QTc reference values for different pediatric age groups.

According to the Brazilian Guideline on Cardiac Arrhythmias in Children and Congenital Heart Diseases, ET is indicated in:^82^

1. Patients with a Schwartz score of 3.0 (intermediate probability), when prolongation of the QTc interval during the recovery phase of the test adds diagnostic value.^57^
2. Asymptomatic family members with resting QTc <440 ms.
3. Patients without a defined phenotype or genotype, for optimization of therapy.
4. Assessment of nonspecific exertional symptoms.

ET may reveal chronotropic incompetence, wave alternans, ventricular tachyarrhythmias, or paradoxical QT interval response to effort and/or recovery (increasing instead of decreasing).^57 , 813 , 815^

Assessment of QTc in the recovery phase has been recommended due to the difficulty in measuring the QT interval at high HR. QTc is measured at 3-4 minutes of recovery; prolongation ≥30 ms is considered significant.^109 , 542 , 548 , 816^

When evaluating efficacy of beta-blocker therapy in patients with LQTS, the objective is to ascertain whether there is a reduction in the chronotropic response and/or suppression of arrhythmias at maximum exertion.^817 – 819^

#### 3.2. Brugada Syndrome

Brugada syndrome (BrS) is an autosomal dominant hereditary channelopathy caused by a defect in sodium channels in the right ventricular epicardium. Mutations in the *SCN5A* gene are the culprit in most cases. The ECG shows a typical pattern of ST-segment elevation in the right precordial leads (V1-V3), with an increased risk of sudden death. The prevalence of BrS in the pediatric population is low (≈1 in 20,000), but the majority of cases are asymptomatic. Some apparently healthy patients present early expression of the disease with initial manifestations such as SND and atrial arrhythmias. BrS can also manifest with syncope, potentially lethal ventricular arrhythmias (polymorphic VT/VF), and cardiac arrest (often during sleep, and/or triggered by hyperthermia and/or medications).^820 , 821^

Risk factors for recurrent arrhythmic events include: previous history of aborted sudden death or syncope; sinus node dysfunction; atrial arrhythmias; intraventricular conduction disorder; large S wave on ECG lead I; and presence of *SCN5A* mutations in adolescents.^822^Some medications and substances are potentially triggering; these include antiarrhythmics, sodium channel blockers, tricyclic antidepressants, local anesthetics, alcohol, and cocaine. A full list is available online at <www.brugadadrugs.org>.^823 , 824^

BrS should be suspected when the resting ECG observed in leads V1 and V2:^308 , 825^

– Type 1: ST-segment elevation ≥2 mm followed by a downsloping, negative T wave ("coved" ST elevation or "shark fin" sign). These findings are diagnostic for type 1 Brugada syndrome.
– Types 2 and 3: ST-segment elevation with an upsloping, positive T wave ("saddleback" ST segment), 2 mm and <2 mm respectively, suggest the presence of channelopathy. However, additional investigation is required.

Another diagnostic criterion for BrS is a Shanghai score ≥3.5, as long as one or more ECG criteria are met.^824 , 826^

Particular features of ET/CPET in BrS:

– As early as age 6-7, ET is indicated for detection of chronotropic incompetence, which is considered a manifestation of sinus node dysfunction. Chronotropic incompetence occurs in ≈ 7% of patients. Approximately 30% of symptomatic children have a history of AFib and SND.^820 , 827^
– Attenuation of ST-segment elevation is generally observed at peak exertion, followed by reappearance during the recovery phase.^126 , 525 , 526^
– Some patients (usually with *SCN5A* mutation) experience an increase in ST-segment elevation (≥0.05 mV) at peak exertion and, especially, in the initial phase of recovery, associated with increased parasympathetic tone. This phenomenon is considered a risk factor for cardiac events, especially in asymptomatic patients and in those with a history of syncope.^126 , 526 , 828^
– There may be increased density and complexity of ventricular arrhythmias as the test progresses.
– ET/CPET can be considered as part of ongoing follow-up of children with BrS to evaluate symptoms such as syncope and palpitations.^824^

#### 3.3. Catecholaminergic Polymorphic Ventricular Tachycardia

Catecholaminergic polymorphic ventricular tachycardia (CPVT) is a hereditary arrhythmic syndrome (channelopathy) characterized by bidirectional ventricular tachycardia, polymorphic VT, and/or ventricular fibrillation, triggered by adrenergic stimuli (physical exertion or emotional stress). CPVT normally occurs in structurally and functionally normal hearts.^829^Its prevalence is ≈1-5,000/10,000 persons; ≈30% of cases are familial. Its mode of inheritance may be autosomal dominant (mutations in the RyR2 gene) or, more rarely, recessive (mainly mutations in the CASQ2, TRDN, and CALM1-3 genes). The average age at onset is ≈10 years. The most common symptoms are dizziness, palpitations, and pre-syncope, which can progress to syncope, hypotonia, seizures, and sudden cardiac death. Sudden death occurs in 30-50% of patients between the ages of 20 and 30. Up to 30% of patients with CPVT have a family history of exercise-induced syncope, seizure, or sudden death.^830 – 832^

ET/CPET is the most relevant diagnostic tool in suspected CPVT, playing a key role in directing therapy of confirmed cases, including clearance for physical exercise.

The pre-test physical examination is usually normal. Suspicion is warranted in patients with a previous history of syncopal episodes that were characterized as vasovagal event or neurological in nature (usually ascribed to epilepsy), considering that in these cases there may simply have been a delay in establishing the diagnosis of CPVT.^829^

The resting ECG generally shows sinus rhythm with normal HR or sinus bradycardia (≈20% of patients), without atrioventricular or intraventricular conduction abnormalities, and a normal QTc. Some patients may exhibit prominent U waves and supraventricular arrhythmias with sinus node dysfunction.^308 , 833 , 834^

Particular features of ET/CPET in CPVT:

– If suspected, perform the test in a hospital setting, with special precautions (see Chart 1 for contraindications) due to the possibility of exercise-induced complications.^835^
– The development of typical exercise-induced symptoms (dizziness, palpitations, pre-syncope, syncope, and sudden cardiac death) is generally associated with complex ventricular arrhythmias.
– Initially, isolated PVCs occur. As the test progresses, PVCs evolve into ventricular bigeminy, followed by polymorphic complexes. If the test is stopped at this stage, the ventricular complexes are likely to disappear gradually. This arrhythmia may be the only abnormality observed in some patients with mild CPVT. Characteristically, the HR at which PVCs occur is between 100 and 130 bpm, and the arrhythmia is typically reproducible.^829^
– Certain characteristics of PVCs can potentially help distinguish CPVT from ventricular arrhythmias in healthy controls: higher density of PVCs; first onset of PVCs with intense exertion (≥10 METs); PVCs with an LBBB pattern and inferior axis; bigeminy or trigeminy at peak exertion; duration of QRS complexes >120ms; coupling interval >400 ms; disappearance of PVCs in the first minute of recovery.^836 , 837^
– The complexity and density of ventricular arrhythmia can worsen as exertion progresses, with VT generally developing when the HR reaches ≈192 bpm. The occurrence of exercise-induced bidirectional VT, with beat-to-beat 180° rotation of the QRS axis (vector alternans), is highly characteristic of CPVT. Development of polymorphic VT followed by ventricular fibrillation occurs during ≈7% of exercise tests.^838^
– Patients with CPVT and chronotropic incompetence present ventricular arrhythmias of greater density and complexity and experience syncope and/or cardiac arrest more frequently compared to those with a normal chronotropic response.^408^
– Some patients may present with exercise-induced supraventricular tachyarrhythmias (including AFib), but these are nondiagnostic of CPVT.^839^
– Bidirectional or polymorphic VT during ET/CPET is highly predictive of CPVT (97% specificity), and has a significant association with genetic mutations. However, sensitivity is usually ≈50%, which means diagnosis of CPVT cannot be ruled out with a single normal exercise test, especially in early childhood.^112 , 840^
– In patients with suspected CPVT and previous normal ET/CPET, modified "sprint" (high work load from the very start of the test, performed on a cycle ergometer, lasting 3 to 6 minutes) or "burst" (high-intensity exercise from the very start of the test, equivalent to the maximum load achieved in the previous ET) protocols can be used in an attempt to uncover the syndrome. Only 28% of carriers of the pathogenic RyR2 variant exhibit abnormalities during ET with a standard protocol. With modified protocols, 83% of tests are abnormal.^113 , 839 , 841^
– ET is essential for screening of first-degree (and, if possible, second-degree) relatives of persons with confirmed CPVT, due to the severity of clinical manifestations, unfavorable prognosis, and possibility of early identification of asymptomatic carriers who would benefit from specific therapy. Screening is generally done with an attenuated protocol. It is important to stress that some patients with CPVT may have a normal test in early childhood and only show abnormalities on subsequent ETs. Therefore, regular monitoring and serial tests are advised.^82 , 842 – 844^
– Serial monitoring with ET/CPET is mandatory to evaluate the effectiveness of established therapy in controlling ventricular arrhythmias and maintaining HR at levels below the triggering threshold. Tests should always be carried out while patients are on their usual medication (including beta-blockers). In patients who continue to show exercise-induced ventricular arrhythmias in doublets, NSVT, or polymorphic or bidirectional VT, the possibility of additional therapy (with flecainide) should be considered. If exercise-induced ventricular arrhythmia and/or symptoms persist, ICD placement with or without left cardiac sympathetic denervation should be considered.^842 , 845 – 847^
– ET must also be carried out as part of preparticipation assessment to clear patients for leisure exercise. Patients who have been asymptomatic for a minimum period of 3 months (including patients with ICDs in place), with no evidence of ventricular ectopics or arrhythmias on ET, and stable on appropriate drug therapy may be allowed to engage in leisure exercise (low to moderate intensity). During physical exercise, patients must remain below the HR threshold known to trigger arrhythmias. The need to avoid dehydration, electrolyte disturbances, and hyperthermia must also be taken into account.^829 , 848^

#### 3.4. Arrhythmogenic Ventricular Cardiomyopathy/Arrhythmogenic Right Ventricular Dysplasia

Arrhythmogenic ventricular cardiomyopathy (ACM) is a hereditary cardiomyopathy characterized by fibrofatty replacement of ventricular myocytes, resulting in electrical conduction abnormalities, cardiac dysfunction, HF, ventricular arrhythmias, and/or sudden death. Although it manifests predominantly in the RV (ARVC or ARVD), it is in fact a pancardiac disease. In adolescents who become symptomatic, biventricular involvement is most common. The prevalence in the overall population is ≈1:5,000, and males are affected at a ≈3:1 ratio. ACM represents one of the most common causes of juvenile sudden death, especially among athletes.^849 – 851^

In the pediatric population, the presentation of ACM varies with age, sex, and genetic inheritance. The most common manifestations are ventricular fibrillation/sudden cardiac death (SCD), which is generally the presenting manifestation of the disease in adolescents; complaints of palpitations and syncope; and HF, which is generally the first clinical manifestation in prepubertal children (≈37% have biventricular involvement) or at advanced stages of the disease (high prevalence).^852 , 853^

ARVC in child age ≤12 years is associated with unfavorable outcomes, with a high incidence of cardiac events including heart transplantation and severe ventricular arrhythmias. In young people, strenuous physical exercise (adrenergic stimulation) can act as a phenotypic modifier of ACM, becoming a trigger for malignant arrhythmias and SCD.^854 , 855^

In equivocal cases, the revised 2010 International Task Force diagnostic criteria and the so-called "Padua criteria" should be applied. In children, the 2010 International Task Force ECG criteria are less applicable, underestimating the actual occurrence of ACM. The Padua criteria, in turn, improve accuracy in children through the use of CMR, stratifying the disease into phenotypic variants (right dominant, left dominant, and biventricular).^856 – 858^

Particular features of ET/CPET in ACM:

– Corrado et al. proposed an update to the Padua criteria to include ET as part of noninvasive clinical assessment, aiming to record the density and morphology of ventricular arrhythmias. If ventricular arrhythmia occurs during the test, the clinician should record its density, the morphology of ectopic QRS complexes, and behavior during each phase of the test (rest, exercise, and recovery).^856^
– Exercise-induced ventricular arrhythmias are relatively common, with monomorphic VT with LBBB pattern being considered typical of ARVC. However, the absence or suppression of ventricular arrhythmias on exertion does not exclude the diagnosis of ARVC.^258 , 859^
– Further indications: in the initial workup; to inform treatment decisions; for preparticipation assessment of adolescents wishing to engage in sports; to distinguish myocardial changes of ACM from those related to physiological remodeling in athletes; to inform exercise prescription or restriction in patients with a confirmed diagnosis; and for optimization of medical surveillance of asymptomatic carriers of ACM-causing genes.^258 , 516 , 860 , 861^
– ET should be part of periodic assessment (every 6 months) of adolescents and young adults with a confirmed diagnosis who engage in low-to-moderate-intensity recreational sports/physical exercise, for assessment of functional capacity and risk stratification. Testing should not be carried out during the most symptomatic periods of the disease ("hot phases"). The presence of exercise-induced symptoms or arrhythmias should lead to conservative recommendations and greater restrictions on exertion activity.^62 , 862^
– In most patients, abnormalities are found on the resting ECG, which in many cases precede structural changes. Symptomatic patients generally have more markedly altered ECGs than asymptomatic patients. Most common ECG findings in patients aged >14 years: inverted T waves in the right precordial leads (V1-V3 or beyond) in the absence of RBBB, epsilon waves (between 7 and 30% of patients), and ventricular arrhythmias.^863 , 864^
– Exercise intolerance is one of the manifestations of patients with HF, and ET is indicated to assess cardiorespiratory fitness and quantify the degree of impairment.^865^
– ET of adolescents and young adults with ARVC presented: effort-induced symptoms (limiting chest pain, severe dyspnea, pre-syncope and palpitations) in 11.4%; pseudonormalization of T waves in 40.0%; ISTE at 8.6%; increased density of ventricular ectopy in 31.4% and non-sustained VT in 11.4%.^516^
– In patients with exercise-induced palpitations and/or syncope and/or VT, diagnostic investigation for ACM should be pursued urgently.^866^
– Asymptomatic patients with mutations in the *PKP2* gene and normal resting ECG may develop exercise-induced epsilon waves.^861 , 867^
– Exercised-induced ventricular depolarization changes are common in asymptomatic carriers of ACM-associated genes: emergence of epsilon waves occurs in 14%; increased duration of terminal activation of QRS complexes (≥55ms) in 32%; and exercise-induced ventricular arrhythmias with a superior QRS axis in 57%.^861^
– CPET is useful in children and adolescents who develop HF (generally due to biventricular involvement) for prognostic stratification, optimization of therapy, and selection of patients for advanced HF therapies (HTx or ventricular assist devices).^852 , 868^

#### 3.5. Complete Heart Block (Congenital and Childhood)

Complete atrioventricular block (or third-degree AV block) is defined as congenital (CCAVB) if diagnosed in utero, at birth, or within the first month of life, whereas childhood AV block is diagnosed between the first month and 18^th^year of life. Acquired complete heart block results from an acute insult, reversible or otherwise. The prevalence of CCAVB is 1 in 15,000 to 20,000 live births (60% are females), with cardiac malformations seen in ≈25% to 50% of cases.^107 , 869^

More than half of CCAVB cases is immune-mediated, caused by autoantibodies that, in susceptible fetuses, damage cardiomyocytes and AV node conduction tissue. Pregnancies may be asymptomatic and ≈⅓ had a previous diagnosis of rheumatic disease (mainly SLE and rheumatoid arthritis). The mother may be asymptomatic; approximately one-third have a preexisting diagnosis of rheumatic disease (mainly SLE and rheumatoid arthritis). The recurrence rate in subsequent pregnancies is 12 to 25%. CCAVB is associated with a mortality rate of ≈16 to 30% (predominantly in utero and in the first months of life) and development of dilated cardiomyopathy (in 5 to 30% of cases).^107 , 870 , 871^

Childhood complete AV block generally results from previously undiagnosed CCAVB, acquired AV block, or hereditary progressive cardiac conduction diseases (associated with mutations in the *SCN5A* , *SCN1B* , *SCN10A* , *TRPM4* , and *KCNK17* genes); some cases are idiopathic. In most cases, it is not associated with structural heart disease or autoimmunity.^583^

Patients with isolated CCAVB (i.e. with no associated cardiac malformation) require close clinical monitoring. They are initially asymptomatic but usually progress to dilated cardiomyopathy due to ventricular dysfunction secondary to bradycardia, which is the leading cause of morbidity and mortality. Significant bradycardia and/or Stokes-Adams attacks are the main indications for pacemaker (PM) implantation.^872^

Acquired complete heart block generally results from: iatrogenic trauma, whether surgical (occurring in 3 to 8% of patients with repaired CHD) or transcatheter; acute or chronic infectious processes; myocarditis; acute rheumatic carditis; acute rheumatic fever; Chagas disease; metabolic disorders (hypothyroidism); infiltrative processes; or a pathological neurocardiogenic mechanism. Although it is a rare and potentially transient finding, ET/CPET is useful for risk stratification and to inform indications for pacing.^107^

In patients with complete AV block, ET is indicated to help document symptoms, assess increased ventricular escape response, ascertain whether ectopy is present, and assess the hemodynamic repercussions of the block.^114 , 554^

Particular aspects of ET in complete heart block:

– On resting ECG, in complete heart block of supra-Hisian origin, a ventricular escape pattern of QRS complexes with normal duration is observed (in acquired cases, it is similar to that seen on pre-block ECGs), in infra-Hisian blocks, the QRS complexes are wide. QTi prolongation in patients with CCAVB is generally a phenotypic manifestation of latent congenital LQTS, and constitutes a risk factor for syncope and/or sudden death.^115 , 873^
– The natural history of congenital complete heart block consists of a progressive decline in ventricular rates throughout life. On resting ECG, between the ages of 6 and 10 years, the average HR is 50 bpm; between 16 and 20 years, 45 bpm.^556^
– Cardiorespiratory fitness provides relevant information about health status and the ability to perform age-appropriate physical activities. Impaired CVF, with or without stress-induced symptoms, is one of the criteria for PM implantation.
– Key exercise-induced symptoms: exercise intolerance, dyspnea, pre-syncope, syncope, Stokes-Adams attacks (especially if the QTi is prolonged).^556 , 874^
– The use of VO_2_max and HRmax prediction equations is not recommended.
– The increase in sympathetic activity without a corresponding effective increase in HR due to escape rhythm can result in complex ventricular arrhythmias and serious complications, especially on a background of CHD or HF. Chronotropic reserve <50 bpm, whether with or without reduced functional capacity (<7 METs), is associated with poor prognosis and need for PM implantation. Exercise-induced ventricular arrhythmia (EIVA) is common (50-70% of patients); its density and complexity are related to the duration of the QRS complexes and increasing age (independent of the HR response to exertion). Complete heart block located within the His-Purkinje system is associated with the occurrence of exercise-induced ventricular ectopics, with an increased risk of sudden death.^115 , 555 , 557^
– Fatigue, dyspnea, dizziness, and exercise-induced ventricular ectopy accounted for approximately 26.5% of pacemaker placements. In asymptomatic patients, other indications were pronounced, persistent bradycardia (including on exertion) and/or prolonged QTc.^107 , 115^

Particular features seen in ET/CPET after pacemaker implantation for complete heart block:

– The ET allows investigation of exercise-induced symptoms, assessment of cardiorespiratory fitness, assessment of atrial rate response, verification of effectiveness of pacemaker programming (rate response), and assessment of potential exercise-induced pacemaker failure; it also helps inform the decision to upgrade the pacemaker (dual-chamber/transvenous).^872 , 875 , 876^
– After pacemaker implantation, ≈20% of children remain symptomatic and/or with impaired cardiorespiratory fitness. This occurs mainly with pacemakers in VVIR (epicardial) stimulation mode at the RV apex.^877^
– The choice of stimulation site (epicardial or transvenous) will generally depend on the patient's weight. The epicardial approach is necessary in patients weighing <10-15 kg, while the transvenous route can be used in those weighing >20 kg. For patients weighing 15-20 kg, either route is feasible. Dual-chamber transvenous pacing is associated with better outcomes in terms of cardiorespiratory fitness.^872 , 878^
– Children with a single-lead pacemaker positioned at the RV apex may develop LV activation and contraction dyssynchrony, resulting in decreased LV function, reduced cardiorespiratory fitness, and chronotropic incompetence. Chronic RV apical pacing can lead to HF in ≈7% of children.^879^
– Patients with LV apical pacing have higher VO_2_peak, HRpeak, chronotropic index, withstand longer exertion, and experience fewer effort-induced symptoms than patients with RV apical pacing.^877^

### 4. Myocardial Ischemia

Myocardial ischemia in the pediatric population is generally a manifestation of one of many conditions and diseases (congenital or acquired) which can cause obstruction of the coronary circulation (dynamic or fixed) and/or microcirculatory dysfunction (see Table 40 ). Although infrequent, myocardial ischemia is a serious, life-threatening event, requiring proper diagnostic investigation, monitoring and follow-up of its natural history, and elucidation of underlying conditions.^19 , 20 , 880^

**Table 40:** Main causes of myocardial ischemia in the pediatric population^19 , 20 , 880 – 882^

Mechanism	Condition
**Atherosclerosis**	CAD in CHD survivors to older ages.
	CAD in CHD due to increased coronary risk factors (i.e. repaired coarctation with persistent hypertension).
	Early CAD due to familial hypercholesterolemia, advanced chronic kidney disease/end-stage renal disease, and systemic lupus erythematosus.
**Coronary reimplantation surgery**	Arterial switch surgery for transposition of the great vessels.
	Coronary reimplantation for anomalous left coronary artery from the pulmonary artery (ALCAPA).
	Aortic valve disease in patients undergoing Ross surgery.
	Ascending aortic aneurysms in patients requiring proximal aortic root replacement.
**Coronary artery compression**	Anomalous origin of the right or left coronary artery from the opposite sinus, with an interarterial/intramural course.
	Proximal pulmonary artery stent placement or percutaneous pulmonary valve implantation compressing a coronary artery.
	Transcatheter aortic valve (TAVI) placed in the aortic position, obstructing the coronary ostium.
	Myocardial bridge.
**Systemic RV**	Corrected TGA.
	Atrial switch repair (Mustard or Senning) of TGA.
**Coronary fistulas**	Coronary fistula.
	Pulmonary atresia with intact ventricular septum, hypoplastic RV, and coronary fistula to RV.
**Williams syndrome**	Supra-aortic aortopathy with narrowing of the coronary artery.

CHD: congenital heart disease; RV: right ventricle: TGA: transposition of the great arteries.

Atherosclerotic CAD in the pediatric population is generally associated with conditions that cause premature atherosclerosis:

1. Familial hypercholesterolemia, an autosomal dominant genetic disorder of cholesterol metabolism. In its heterozygous form, it affects 1:250 individuals, causing premature atherosclerosis in adolescents and young adults.^59 , 883 , 884^
2. Advanced chronic kidney disease (CKD), especially end-stage renal disease (ESRD)/dialytic CKD. Coronary calcification is common in this setting and is associated with uremia, abnormal mineral metabolism, increased fibroblast growth factor (FGF)-23 levels, and Klotho factor deficiency. Children with CKD have a high prevalence of risk factors for atherosclerotic CVD, similar to those observed in adults with this condition. The American Heart Association stratifies pediatric patients with CKD into the high-risk category for developing early CVD and atherosclerotic CAD before the age of 30.^885 , 886^
3. Systemic lupus erythematosus (SLE), an autoimmune disease characterized by a relapsing-remitting pattern of systemic inflammation with tissue damage caused by formation of immune complexes and/or deposition of autoantibodies. SLE is associated with accelerated atherosclerosis, CAD, PAD, VHD, myocarditis, LV dysfunction (in children with active SLE), and increased risk of CV events. Early atherosclerosis occurs secondary to hyperleptinemia and abnormalities in immune regulation, endothelial cell function, and vascular repair. CAD can occur at any stage of SLE, with younger individuals being at the highest risk.^887 – 890^

Markers of high MI risk in children and adolescents complaining of chest pain include: abnormal cardiovascular findings on physical examination (i.e. heart murmur, cyanosis, peripheral pulse changes, etc.); chest pain or syncope on exertion; chest pain associated with palpitations; abnormal ECG; family history of arrhythmias, sudden death, or genetic disorders; history of cardiac surgery or interventional procedures; heart transplantation; history of Kawasaki disease; history of familial hypercholesterolemia; and a diagnosis of CKD and/or SLE.^20^

The resting ECG should highlight any arrhythmias, conduction, and ST-segment/T wave changes (which may suggest pericarditis, myocarditis, or CAD), as well as evidence of LVH. The presence of LBBB, WPW, or an artificial pacemaker will hinder analysis of repolarization changes as markers of ischemia during ET.^7^

Particular features of ET/CPET in myocardial ischemia:

– Indicated as part of the chest pain workup in children and adolescents at high risk of ischemic cardiovascular events ( Table 40 ).
– Parameters such as VO_2_peak, oxygen pulse, VE/VCO_2_slope, and ΔVO_2_/ΔWR ratio aid in the diagnosis of myocardial function impairment, treatment decisions, and medical clearance for or prescription of physical activities.^179^
– Congenital anomalies of the coronary arteries are a known common cause of exercise-induced myocardial ischemia. They include: anomalous origin of the aorta or pulmonary artery, abnormal ostium, and intra- or intermural arterial course (between the aorta and the pulmonary artery).^891^
– After surgical correction of these anomalies, ET is indicated for risk stratification and optimization of therapy (enlargement of the coronary ostia, reimplantation with or without prolongation of the coronary arteries, translocation of the pulmonary artery, myocardial revascularization).^891^
– After arterial switch procedures and the Ross procedure, ET is indicated to stratify the risk of early postoperative ischemia and myocardial dysfunction. Late coronary ischemia may require reoperation.^891 , 892^
– In patients with hypoplastic left heart syndrome and Fontan palliation, the incidence of exercise-induced ST-segment depression was 48%, with no deaths recorded during ≈2 years of follow-up. Patients further investigated did not exhibit reversible perfusion defects or obstructive CAD.^520^
– In patients evaluated for residual coronary artery lesions after corrective surgery (due to TGA, anomalous origin, or ALCAPA), exercise-induced ST-segment elevation had 100% sensitivity and 81% specificity for severe residual lesions (>50%). The risk markers for serious lesions were effort-induced chest pain (RR: 4.72; 95% CI: 1.23-18.17) and intramural pathway (RR: 4.37; 95% CI: 1.14-16.81).^893^
– Children with myocardial bridging and hypertrophic cardiomyopathy showed shorter exercise time, lower SBPpeak (mean reduction 17±27 mmHg), greater QTc dispersion (104±46 msec), and exercise-induced ST-segment depression (median 5 mm). During 7.1±5.4 years of follow-up, chest pain was observed in 60% of patients, VT in 80%, and cardiac arrest with subsequent resuscitation in 50%.^894^

### 5. Valvular Lesions

#### 5.1. Congenital Aortic Stenosis

Congenital aortic stenosis (AS) is a heart defect that causes hemodynamically fixed, significant obstruction of the LV outflow tract. It accounts for ≈3-6% of CHD cases, and is more common in males (male-to-female ratio 3:1 to 5:1). Approximately 15 to 20% of patients with AS have other associated CHDs, most commonly PDA, coarctation of the aorta, or VSD.^895^

In critical AS, a unicuspid aortic valve is commonly seen, having either an eccentric orifice with a patent commissure or a central orifice with an absent commissure. Bicuspid aortic valves are generally associated with dilation of the ascending aorta, with enlargement and degenerative changes of the valve as the child grows.^370 , 896^

AS in early childhood is usually severe (critical) and is associated with LV failure, signs of low cardiac output, HF, cardiomegaly, pulmonary edema, pallor or gray discoloration of the skin, hypotension, and dyspnea. Most children and adolescents with mild AS remain asymptomatic and have normal growth and development. Dyspnea, angina, or syncope, particularly on exertion, occur in ≈10% of the affected pediatric population aged 5-15 years. The onset of symptoms requires immediate assessment because of the risk of sudden death (≈1-10% in patients with moderate-to-severe AS). Approximately 2 to 4% of all young athletes with SCD have AS.^181^

Congenital AS is associated with development of LVH and an increased risk of CVD. Supravalvular aortic stenosis (most commonly associated with Williams syndrome) may confer increased CV risk due to its association with stenosis of the coronaries (with myocardial ischemia and exercise-induced syncope) and renal arteries (which may cause secondary hypertension).^81^

Abnormal findings on resting ECG are nondiagnostic of AS and are not sensitive enough to determine the degree of severity. However, evidence of LVH and ST-segment depression ≥2mm are relatively sensitive indicators of severe AS. Ventricular arrhythmias are common in moderate/severe AS. QT dispersion is prolonged in children (particularly in those with arrhythmia), and the degree of prolongation is related to the pressure gradient and LV mass index.^388 , 897 , 898^

Particular features of ET/CPET in congenital AS:

– Contraindicated in symptomatic moderate/severe AS.
– Indicated for the assessment of children and adolescents with AS who have a mean gradient at rest <30 mmHg or a peak gradient <50 mmHg.
– Indicated in moderate AS for preparticipation assessment of children and adolescents wishing to take pert in sporting activities. For medical clearance, the child must reach a level of effort during ET consistent with the desired activity, demonstrate satisfactory cardiorespiratory fitness, a normal SBP response to exercise, and complete absence of symptoms, ST-segment depression, or ventricular tachyarrhythmias.^181^
– Asymptomatic patients with moderate/severe AS generally exhibit poor cardiorespiratory fitness, especially if the LV systolic gradient is ≥30 mmHg. The degree of impairment is related to the aortic valve area at rest.^436 , 899^
– Most asymptomatic patients with moderate AS have a moderate increase in SBP (<25 mmHg).
– The change in SBP from baseline during exertion (ΔSBP) depends on the degree of stenosis, being less in severe AS (ΔSBP = 21.6 mmHg) than in moderate AS (ΔSBP = 32 mmHg).^900^
– In moderate/severe AS, exercise-induced ST-segment depression, an inadequate drop or increase in SBP, and exercise-induced arrhythmias may occur.^437^
– The severity of AS is associated with exercise-induced ST-segment depression (odds ratio: 12.0; 95% CI: 3.0-49.0). Exercise-induced ST-segment depression is related to LV systolic pressure, LV outflow gradient (especially if ≥70 mmHg), and the oxygen supply-demand relationship.^436 , 518 , 899^
– In supravalvular AS, complex ventricular arrhythmias and worsening of ST-segment depression with exertion usually occur and are indicative of myocardial ischemia.^434^
– After surgical treatment of AS, there is a reduction in exercise-induced ST-segment depression and increases in ΔSBP and cardiorespiratory fitness.^519^

#### 5.2. Aortic Regurgitation

Aortic regurgitation (AR), or aortic insufficiency (AI), is characterized by an increase in left ventricular end-diastolic volume, increased wall strain, and compensatory myocardial hypertrophy. AR rarely occurs as an isolated lesion; it is often comorbid with AS (including after surgical or transcatheter intervention) or VSD. A bicuspid aortic valve is the most common cause of AR.^896^

Chronic AR is generally well tolerated and most children remain asymptomatic, even with a major lesion. However, in moderate/severe AR, the development of significant symptoms and/or LV dysfunction is common, and surgical intervention is required. Severe AR results in greatly increased LV end-systolic and end-diastolic volumes, generally leading to progressive dysfunction. In severe AR, reduced diastolic pressures at the aortic root can impair coronary perfusion.^901 – 905^

The resting ECG in moderate/severe AR usually presents a LVH pattern and, in the chronic stage, ST-segment and T wave changes.^388^

Particular features of ET/CPET in AR:^903 , 904 , 906^

– Indicated for assessment of symptoms, CRF, exercise-induced ischemia, optimization of therapy, and medical clearance/prescription of physical exercise.
– Patients who develop signs or symptoms of HF and/or exercise-induced ischemia or decline in LV function generally require surgical intervention.
– Patients with moderate or severe AR present with impairment of HRpeak, blood pressure (including intra-exercise pressure drop), and respiratory quotient (RQ). There is also a higher incidence of ectopy and exercise-induced ST-segment depression.
– In athletes, ET it is indicated to confirm possible symptoms and evaluate exercise tolerance and the BP response to exercise, parameters which must be assessed before the patient can be cleared to practice sports. A level of activity comparable to that of the intended sport, or greater, must be achieved during the ET.
– Asymptomatic athletes with mild to moderate AR, no LV dysfunction, and a normal ET can participate in all competitive sports (Class of Recommendation: I; Level of Evidence: C).
– Moderate/severe AI allows participation in recreational sporting activities only if LVEF >50%, the LV is not enlarged (<35 mm/m^2^), and the ET is normal (Class of Recommendation: IIb; Level of Evidence: C).

#### 5.3. Bicuspid Aortic Valve

Bicuspid aortic valve (BAV) is a congenital malformation that can occur both as an isolated lesion and in association with CHD. The prevalence of isolated BAV is approximately 1-2% in the general population, 15-30% in Turner syndrome, and 50-85% in patients with coarctation of the aorta. BAV is common in chromosomal diseases such as Down syndrome (trisomy 21), DiGeorge syndrome (22q11), Edwards syndrome (trisomy 18), and other genetic syndromes, such as Williams syndrome, Holt-Oram syndrome, Marfan syndrome (4.7%), and Loeys-Dietz syndrome (8.8%).^139 , 330 , 907^

Abnormalities of the aortic root, sinotubular junction, and ascending aorta occur as part of this lesion. Dilation of the aortic root and ascending aorta is common, even in patients with no stenosis or regurgitation. In AS, the risk of developing severe aortic dilation in adolescence and early adulthood is greater. In Marfan syndrome with BAV and aortic dilation, there is a greater risk of spontaneous rupture. Most children with BAV are asymptomatic until adulthood. In selected pediatric cohorts with BAV but no severe stenosis or concomitant CHD, <5% require intervention on the valve before adulthood.^139 , 330 , 907^

Particular features of ET/CPET in BAV:

– ET is indicated for assessment of symptoms and of cardiorespiratory fitness in patients who have developed moderate/severe AS, AR, or coarctation of the aorta.^370^
– Adolescents with BAV and Williams syndrome generally present with reduced total exercise time; an accelerated chronotropic response; a hypertensive SBP response to exertion; and absence of exercise-induced ST-segment depression.^908^
– Indications for balloon valvuloplasty include severe AS, peak systolic gradient at rest ≥50 mmHg without symptoms or ≥40 mmHg with angina, syncope, and ST-segment changes, whether at rest or exercise-induced.^909^

#### 5.4. Pulmonic Stenosis

Pulmonic stenosis (PS) is a narrowing of the pulmonic (or pulmonary valve), usually due to fusion of its leaflets, with obstruction of the RV outflow tract and reduced blood flow to the pulmonary arteries. It is the most common form of RV outflow tract obstruction, accounting for 90% of cases.^910 , 911^

The severity of PS determines the treatment strategy, which may include surgical and/or transcatheter intervention. PS is classified on the basis of the right ventricular to pulmonary arterial (RV-PA) pressure gradient: 10 to 30 mmHg, mild; >30 to 60 mmHg, moderate; >60 mmHg or RV pressure greater than systemic pressure, severe.^910 , 912 , 913^

Children with discrete PS, with intact interventricular septum (isolated PS), are generally asymptomatic and exhibit normal CRF. Spontaneous regression of the stenosis may occur with advancing age. Conversely, patients with moderate PS – especially symptomatic ones – develop worsening RV hypertrophy, outflow tract obstruction, and ventricular dysfunction, requiring interventional treatment. Severe PS occurs mainly in childhood and often progresses to RV dysfunction, HF, tricuspid regurgitation, and cyanosis, requiring early interventional treatment.^370 , 911^Over 13.5 years of follow-up, isolated PS (i.e. with an intact interventricular septum) was associated with an increase in overall mortality (RR: 4.67; 95% CI: 3.61-5.99). Patients with early diagnosis (within the first year of life) had the highest risk of mortality (RR: 10.99; 95% CI: 7.84-15.45).^910 , 912 , 913^

After valve intervention, long-term event-free survival is >90%. Complications include pulmonic regurgitation with possible RV volume overload (≈⅓ of patients) and restenosis (5-10% of patients), especially in the first year after intervention.^911 , 914 , 915^

The resting ECG in mild isolated PS is generally normal, but in children there may be T wave inversion in the right precordial leads. In moderate/severe cases, a pattern of RV hypertrophy and right atrial enlargement ("P pulmonale") is generally observed, as well as deviation of the QRS axis to the right and RBBB.^388 , 911^

Particular features of ET/CPET in isolated PS:

– Is useful in preparticipation assessment before enrollment in a physical exercise program and aids in symptom assessment by providing direct information about the ability of the RV to maintain cardiac output during conditions of increased workload. RV systolic pressure, assessed through physical stress echocardiography, is normally elevated at rest, increasing further during exertion.^87 , 906^
– In mild stenosis, CRF it is generally normal; in moderate cases, it is usually impaired; in severe cases, impairment is more pronounced and symptomatic, leading to a worse quality of life, but generally improves after intervention.^916 – 918^
– The chronotropic response is generally normal, regardless of the severity of the stenosis.^87^
– Exercise-induced ST depression is exceedingly rare and exercise-induced arrhythmias may occur.^919^
– CPET performed ≈8 years after balloon valvuloplasty of the pulmonic valve in patients with severe PS showed normal VO_2_peak (32.63±8.38 ml/kg/min), HRpeak (174.88±5.01 bpm), drop in HR in the first minute of recovery (28.04±4.70 bpm), SBPpeak (164.02±11.03 mmHg), peak DBP (84.42±7.63 mmHg), FVC (2.56±0.39 L), and FEV_1_(2.43±0.34 L).^380^Monomorphic exercise-induced ventricular arrhythmias occurred in 10.9% of children, and none exhibited any ST segment changes.^900^

#### 5.5. Pulmonic Regurgitation

Pulmonic regurgitation (PR) or insufficiency (PI) is usually asymptomatic and well tolerated in childhood. However, in rare cases PR may worsen progressively, leading to RV enlargement and dysfunction, exercise intolerance, ventricular tachycardia, and SCD. Patients with mild/moderate PR are generally asymptomatic. In severe PR, exercise intolerance with dyspnea is often observed, due to the patient's inability to increase RV output. If there is right ventricular failure, patients may experience hepatic congestion, ascites, and lower-limb edema. Atrial and right ventricular remodeling confers a greater risk of arrhythmia with dizziness and/or syncope. Exercise-induced symptoms, progressive exercise intolerance, HF, and sustained arrhythmias suggest an unfavorable course and indicate valve intervention/repair.^97 , 709 , 920^

The resting ECG may reveal deviation of the QRS axis to the right, RV hypertrophy pattern, and RBBB. Arrhythmias are common in severe PR.^177^

Particular features of ET/CPET in PR:

– In a retrospective cohort, children undergoing pulmonary valve replacement surgery and/or conduit revision who had better CRF preoperatively (VO_2_peak ≥70% of predicted) had a shorter length of stay.^921^
– In a retrospective cohort, pulmonary valve replacement after delayed ToF correction was associated with improvement in RV volume. Approximately 28% of patients achieved normalization of RV end-systolic volume, but no significant improvement in CRF.^712^
– Percutaneous pulmonary valve replacement in patients with PR associated with other CHDs did not lead to improvement in VO_2_peak, RQ, or oxygen pulse. On multivariate analysis, reduction in the RVOT gradient was the only predictor of improvement in VO_2_peak.^922^
– Patients with severe PR who are asymptomatic, with no significant RV volume overload, no arrhythmias, normal RV systolic function, and a normal ET can be medically cleared for recreational sports.^681^

#### 5.6. Mitral Stenosis

Specific mitral valve defects in mitral stenosis (MS) are classified based on their relationship to its annulus, including valvular, supravalvular, and subvalvular components (chordae tendineae and papillary muscles). The clinical presentation varies depending on the degree of valve obstruction and the presence of mitral regurgitation, secondary PAH, pulmonary diseases, and/or other cardiac lesions.^923^

Congenital MS rarely occurs in isolation; it is usually associated with coarctation of the aorta, AS, and CHDs (Ebstein's anomaly, *cor triatriatum* , ToF, etc.). In moderate-to-severe stenosis, symptoms usually appear in the first or second year of life: failure to thrive, wheezing, and varying degrees of dyspnea and pallor.^924^

The resting ECG generally shows a pattern of RV hypertrophy, QRS axis deviation to the right, and notched/bifid or peaked P waves, indicative of left atrial enlargement. Atrial fibrillation is exceedingly rare.

Particular features of ET/CPET in MS:

– Patients with mild to moderate MS may be asymptomatic even during strenuous exercise.
– In uncorrected MS, ET is indicated as part of the preparticipation assessment to confirm asymptomatic status; subjects must be able to reach at least the level of exertion consistent with the activity they wish to pursue.^681^
– In moderate MS, the ET must be normal if patients are to be cleared for low-to-moderate-intensity exercise. Annual follow-up ET is recommended.^681^
– In moderate/severe MS, the increase in HR and cardiac output upon exertion can increase the gradient, pulmonary capillary pressures, and PAH, causing low exercise tolerance, worsening of symptoms, and, occasionally, acute pulmonary edema.^46^
– 6 months after valvuloplasty, improvement in CRF and cardiac output was noted.^47^

#### 5.7. Mitral Regurgitation

Mitral regurgitation (MR) is a valvular lesion characterized retrograde blood flow from the LV to the left atrium and subsequent LV volume overload. To maintain cardiac output, compensatory changes such as increased contractility and LVH may develop. MR can progress to ventricular remodeling and, eventually, diffuse LV enlargement and dysfunction. Chronic overload of the left atrium and ventricle impairs blood drainage through the pulmonary veins, causing pulmonary congestion and HF symptoms. Congenital MR (CMR) is a rare disease of childhood, and occurs in combination with other cardiac lesions in up to 60% of cases.^132 , 925^

Mild MR produces no symptoms; the only abnormal sign is auscultation of an apical holosystolic murmur. Severe insufficiency, however, results in symptoms that can appear at any age, including physical underdevelopment, frequent respiratory infections, fatigue on exertion, pulmonary edema, and congestive HF.

Clearance for or even recommendation of physical exercise/sport depends on the severity of MR, the degree of LV enlargement, LV systolic function, and PAH. Static exercises causing large increases in BP or HR can result in potentially harmful increases in regurgitant volume and pulmonary capillary pressures.^681 , 926^

In moderate/severe MR, the resting ECG often shows bifid P waves (left atrial enlargement) and evidence of LVH. In the most severe cases, an RVH pattern is visible.

Particular features of ET/CPET in MR:

– Mild MR generally does not cause impairment of CRF.
– Compensated mild/moderate MR is generally asymptomatic, with good exercise tolerance and normal CRF, and may remain so for years.^927^
– Adolescents with severe MR, asymptomatic, may be released for low-intensity activities if they have normal ET, preserved LV function at rest, pulmonary arterial pressure <50 mmHg and absence of effort-induced ventricular arrhythmia.^681^
– Severe MR with LV dysfunction presents with symptoms of HF, exercise intolerance, and poor CRF. CPET aids in risk stratification, optimization of therapy and, in severe cases, to decide if heart transplantation is indicated.
– After valve replacement or repair, for assessment of CRF, optimization of therapy, and medical clearance for physical activity/exercise prescription, including rehabilitation.

#### 5.8. Mitral Valve Prolapse

Mitral valve prolapse (MVP) is characterized by systolic protrusion of the mitral valve leaflets into the left atrium, with or without mitral regurgitation. A genetic predisposition is involved in the pathogenesis of MVP. It can be primary ("nonsyndromic") or secondary ("syndromic") to connective tissue disorders (Marfan syndrome, Loeys-Dietz syndrome, Ehlers-Danlos syndrome, osteogenesis imperfecta, pseudoxanthoma elasticum, and osteoarthritis syndrome). It can also occur in hypertrophic cardiomyopathy. In the pediatric population, it is often considered benign and asymptomatic. When symptomatic, the chief complaints are palpitations, dizziness, chest pain, dyspnea, pre-syncope, and syncope.^132 , 928^

MVP in adolescent and young adult athletes, with myxomatous valve degeneration, is a relevant cause of arrhythmic SCD (arrhythmogenic MVP), with an annual incidence of ≈0.2-1.9%. Prolapse in both valve leaflets, moderate/severe mitral regurgitation, and ventricular arrhythmia are markers of a higher risk of events. Adolescents and young women with mitral leaflet thickening and/or prolapse of both leaflets may have an increased predisposition to complex arrhythmias and arrhythmogenic SCD.^929 – 931^

The resting ECG is normal in most patients. However, it may show inverted T waves in inferior wall leads, PVCs with a RBBB pattern, and QTi prolongation (mainly in athletes). In patients with chronic mitral regurgitation, patterns consistent with LA and LV hypertrophy may be observed, as may exercise-induced ST depression.^931 – 933^

Particular features of ET in MVP:

– ET is useful for assessment of symptoms, determination of exercise tolerance, detection of exertional arrhythmias, and medical clearance for physical activity/exercise prescription (including competitive sports).^926 , 934^
– Exercise intolerance / impaired CRF are common.^935^
– Even when CRF is normal, patients have a lower peak double product.^935 , 936^
– Exercise-induced ventricular arrhythmias with a RBBB and/or complex pattern are markers of risk in patients with suspected arrhythmogenic MVP.^934 , 937^
– During ET, around 38% of adolescent athletes with ventricular arrhythmias developed PVCs with RBBB morphology at rest or exertion.^938^
– If there is concomitant moderate/severe MR, there is an increased risk of morbidity and mortality when LV systolic function and CRF are compromised. In these patients, valve repair or replacement should be considered.^370^

### 6. Dyspnea and Exercise Intolerance

#### 6.1. Exercise-induced Dyspnea

Exercise-induced dyspnea (EID) is a very common clinical manifestation in children and adolescents, characterized by shortness of breath, increased work of breathing, increased respiratory frequency, and chest discomfort. EID is a subjective sensation that can have several underlying etiologies, and may occur even in the absence of any detectable disease. It is the cause of discontinuation or cessation of effort in ≈52% of children. More than 14% of apparently healthy adolescents experience an episode of EID every year.^178 , 939 – 941^

The mechanisms and pathophysiology of dyspnea involve interactions between the cardiorespiratory system and neural responses. Dyspnea is believed to be caused by a mismatch between ventilation and the neural respiratory drive. Initially, respiratory changes resulting from effort occur predominantly through increases in tidal volume (TV) and after reaching approximately 50% of vital capacity through an increase in RR. Tachypnea develops once the VT plateau is reached. Ventilatory factors including chest discomfort, intense work of breathing, and ventilatory disturbances (with audible manifestations such as stridor and wheezing) can contribute to the sensation of dyspnea and its perceived severity.^78 , 939^

The main causes of EID are: exercise-induced asthma; exercise-induced bronchospasm; exercise-induced laryngeal obstruction; exercise-induced vocal cord dysfunction; restrictive chest wall abnormalities; metabolic diseases (i.e. McArdle disease, hypothyroidism, etc.); myasthenia gravis; and cardiovascular diseases, including CHD, cardiomyopathies, HF, hypertension, VHD, and arrhythmias.^78 , 939 , 942^

Particular features of ET/CPET in EID:

– ET is indicated for elucidation of symptoms and mechanisms involved in dyspnea, assessment of cardiorespiratory fitness, to inform treatment decisions, and for medical clearance/prescription of physical exercise.
– Use of the pictorial Dalhousie Dyspnea and Perceived Exertion Scales is recommended to quantify the degree of impairment and impact of dyspnea.^178 , 943 , 944^
– The perception of dyspnea must be correlated with the actual work load, VO_2_, and ventilation at which it developed and also at the moment of maximum intensity.^945^
– In CPET, for diagnostic investigation, baseline spirometry must be performed followed by a maximum incremental effort protocol, with spirometry repeated during recovery.
– Arterial oxygen saturation must be monitored continuously via pulse oximetry (SpO_2_); reductions >5% are indicative of exercise-induced hypoxemia.
– If associated with wheezing or audible adventitious sounds, EID is often associated with exercise-induced asthma or bronchospasm.
– EID with chest pain, marked reduction in ventilatory efficiency, and elevated VE/VO_2_and VE/VCO_2_ratios indicates abnormal gas exchange in the lungs, usually associated with PAH.^631^
– EID due to restrictive lung diseases is associated with reduced cardiorespiratory fitness (low VO_2_at VT1 and at peak exertion), increased tidal volume (50% of vital capacity and/or 80% of inspiratory capacity), and relatively low VR.^630^
– Unexplained dyspnea with a feeling of suffocation, hyperventilation, but no desaturation or changes in gas exchange is generally associated with psychogenic illness and/or panic disorder.^629 , 946^

#### 6.2. Exercise-induced Bronchospasm

Exercise-induced bronchospasm (EIB) is an acute, transient airflow obstruction phenomenon. It generally occurs 5 to 15 minutes after cessation of exertion. Symptoms are nonspecific and mild to moderate in intensity: chest tightness, chest pain, abdominal pain, cough (sometimes as the only symptom), wheezing, and dyspnea. Very rarely, severe episodes with life-threatening respiratory failure may occur.^947 , 948^

Although the term "exercise-induced asthma" (EIA) was previously used as a synonym for EIB, this practice is no longer recommended as they are distinct entities, including in terms of diagnosis and treatment criteria. EIA is characterized by chronic bronchial hyperactivity and inflammation, while EIB represents transient narrowing of the airways (always associated with physical exertion) which can occur even in non-asthmatic patients. EIA benefits from corticosteroid therapy to control underlying chronic inflammation, while EIB, in most cases, requires administration of a short-acting beta2-agonist prior to any physical exertion.^948 , 949^

In the pediatric population, risk factors for EIB include: atopic dermatitis; sensitization to indoor allergens; high IgE levels (seasonal and perennial); environmental factors (exposure to cold air, high atmospheric pressure, humidity, and pollutants); and, in asthmatic children, eosinophilic inflammation of the airways and fraction of exhaled nitric oxide (FeNO) >20 particles per billion (ppb) in patients not on corticosteroid therapy or >12ppb in those on corticosteroids.^947 , 948 , 950^

EIB is observed in 40-90% of children with asthma, especially in those with severe, uncontrolled asthma. The prevalence ranges from 7% to 35% in the pediatric population and is ≈23.1% in adolescent athletes. The combination of EIB and EILO occurs in 4.8% of adolescents, being most prevalent in males (64.7%).^164 , 951^

Particular features of ET/CPET in EIB:

– CPET is indicated for the diagnosis of EIB, assessment of cardiorespiratory fitness, determination of effort-limiting factors, assessment of the severity of dynamic hyperinflation, and assessment of the response to therapeutic interventions.^952^
– CPET performed for the specific purpose of diagnosing EIB is also known as an exercise bronchial challenge test or bronchial provocation test. It is generally performed on a treadmill, as these protocols are more prone to EIB.
– Clinicians are advised to use a protocol with a fixed, high-intensity load to cause a rapid increase in ventilation and avoid refractoriness to the development of bronchospasm. The starting grade/incline should be 5.5% and the speed should increase quickly, with subjects reaching at least 80% of their predicted maximum capacity at 2 minutes, after which the work load must be maintained. Incremental protocols, whether on a treadmill (Bruce) or cycle ergometer (Godfrey), are less effective in triggering EIB.^953^
– Aim to reach the maximum work load and/or 80 to 90% of the estimated HRmax between 6 and 8 minutes of the test. The temperature of the room should be kept between 20-25°C, and relative humidity always <50% (dry air).^952 , 954^
– Around 50% of asthmatic patients without a history of EIB and ≈40% of atopic patients without asthma may develop EIB during ET.
– Administration of bronchodilators before ET/CPET should be considered when the test is being performed to assess treatment response.
– Diagnosis and quantification of the severity of EIB are established by changes in lung function caused by exertion, regardless of the occurrence of symptoms.
– The forced expiratory volume in the 1^st^second (FEV_1_) must be measured at rest and recovery (at 5, 10, 15, and 30 minutes after exertion). A >10% difference between the resting FEV_1_value and the lowest FEV_1_reached in the first 30 minutes after exercise establishes the diagnosis of EIB.^163^
– The severity of EIB can be classified based on the percent drop in FEV_1_in relation to baseline (resting level): ≥10% but <25%, mild; ≥25% but <50%, moderate; and ≥50%, severe.^163^
– Patients with mild EIB generally require more than one ET/CPET to confirm the diagnosis.^950 , 955^
– If moderate/severe symptoms occur during or after exercise, even in the absence of a significant drop in FEV_1_, administration of a bronchodilator is recommended. This may also be necessary at the end of the test if FEV_1_does not return to a value no more than 10% below the resting FEV_1_.^953^

#### 6.3. Exercise-induced Laryngeal Obstruction

Exercise-induced laryngeal obstruction (EILO) is defined as a transient obstruction of the upper airways, typically occurring at the supraglottic level and often followed by glottic involvement, which causes reduced airflow and dyspnea on exertion. The cause of EILO is unknown. The most relevant risk factors are asthma; gastroesophageal reflux disease; diseases or anatomical variants of the upper airways (i.e. vocal cord dysfunction); heredity; environmental factors (worse in cold, humid air); psychological stress; and high-intensity physical activity/sports. It is a major cause of respiratory problems and upper airway dysfunction in adolescent athletes. Adequate management and treatment require ruling out any other possible causes of symptoms, such as asthma, EIB, and airway hyperreactivity.^956 – 958^

Overall, the prevalence of OLEI varies with age (more frequent between the ages of 11 and 18), sex (3:1 female-to-male ratio), and athletic level (more common in high-performance competitive athletes). Among adolescent athletes the prevalence is 8.1%, and an association with exercise-induced asthma is common (14 to 38% of affected athletes).^164 , 951 , 959^

Patients generally present with exertional dyspnea; respiratory discomfort; tightness in the throat; feeling of suffocation; tightness in the upper chest; chest pain; noisy, stertorous breathing; changes in voice and hoarseness; cough; prolonged inspiration; hyperventilation episodes; and panic attacks. The only complaint may be a feeling of "labored breathing".^960 , 961^

Particular features of ET/CPET in EILO:

– Regarding tests to confirm the diagnosis of OLEI, it is recommended that they be carried out in a hospital environment with a multidisciplinary team (including an otorhinolaryngologist) and adequate conditions to deal with possible complications.
– ET with continuous flexible nasal laryngoscopy during high-intensity exercise is recognized as the "gold standard" for the diagnosis of EILO. This involves advancing a flexible video laryngoscope (with continuous recording) through the nose to view the larynx in real time. In addition to diagnosis, this allows assessment of the severity of laryngeal obstruction at the moment of greatest symptom severity, as well as evaluation of treatment efficacy.^962 , 963^
– In athletes, the ergometer and effort protocol should ideally be selected so as to mimic their sport or activity as closely as possible, in order to ensure achievement of maximum effort and the best possible ventilation.
– The test is positive if it reproduces the patient's laryngeal symptoms (ideally with a concomitant plateau in VO_2_and/or HR response in a maximal test) and video laryngoscopy records the presence, site, and severity of laryngeal obstruction. In the presence of concomitant supraglottic and glottic obstruction, the location where the obstruction first occurs must be determined and recorded.^956 , 964^
– CPET combined with continuous laryngoscopy allows simultaneous assessment of respiratory and metabolic variables, contributing to the differential diagnosis of other causes of exertional dyspnea.^958 , 965^
– The main CPET variables to be recorded when EILO is suspected are pulmonary ventilation, VO_2_peak, RQ, and flow volume loops.^956^
– Symptoms generally occur close to peak exertion, are more evident during the inspiratory phase, and may be associated with stridor (wheezing/whistling on inhalation). They generally resolve within 2 to 3 minutes after cessation of exertion, but may persist for longer in patients who continue to hyperventilate during recovery.^956 , 963^
– If the initial symptoms/signs go unrecognized or there is a delay in cessation of exertion, frank laryngospasm may occur due to exacerbated closure of the glottis, preventing ventilation altogether. This is an exceedingly rare situation occurring late in the course of an EILO episode, but will progress to desaturation, bradycardia, and central cyanosis and requires immediate emergency treatment.

#### 6.4. Exercise-induced Asthma

Asthma is a heterogeneous, chronic inflammatory disease characterized by a reversible airway flow limitation, which resolves spontaneously or after treatment. Principal symptoms are wheezing, shortness of breath, chest tightness, and cough. Episodes are often triggered by emotions, dust, and/or exposure to allergens. Impaired cardiorespiratory fitness, exercise-induced dyspnea, fatigue, and a reduction in quality of life are common. The prevalence of asthma symptoms among adolescents in Brazil is ≈20-23%, one of the highest in the world, with only 12% having a known diagnosis of asthma.^966 – 968^

Exercise-induced asthma (EIA) is a condition of airway restriction in patients who already have bronchial hyperactivity and persistent inflammation (i.e. established asthma), while in EIB, the airway restriction is temporary and occurs mainly in non-asthmatics. EIA is triggered by inhalation of cold, dry air during exercise, which causes dehydration of the airway mucosa with increased osmolarity, contraction of bronchial smooth muscles, an influx of eosinophils/mast cells, and subsequent release of proinflammatory mediators (leukotrienes, histamine, IL-8, tryptase, and prostaglandins). EIA is observed in ≈40-90% of children with asthma, especially in those with severe, uncontrolled disease. The main symptoms are coughing, wheezing, chest tightness, and unusual shortness of breath or excess mucus production after strenuous, continuous aerobic exercise. Symptoms generally begin to appear 5 to 8 minutes after the start of continuous exercise, or within 2 to 5 minutes in particularly high-intensity exercise. EIA is usually confirmed by spirometry before and after ET/CPET.^948 , 949 , 969^

EIA often leads to significant limitations in physical activities and hinders participation in sports; however, in patients with adequately controlled asthma, regular exercise is recommended, not least to avoid obesity and other factors that actually make asthma worse. In patients with EIA, administration a short-acting beta2-agonist 5 to 20 minutes before exercise is advised. Additionally, daily use of inhaled corticosteroids, leukotriene receptor antagonists, or mast-cell stabilizers may be necessary for treatment of asthma.^947 , 969^

Particular features of ET/CPET in EIA:

– Asthmatic patients’ response to exertion depends on the degree of airway obstruction and its reversibility. During exertion, minute ventilation increases to meet muscular metabolic demands. An increase in tidal volume is the dominant mechanism in low to moderate ventilation. At high levels of exertion, additional increases in minute ventilation are primarily attributable to increases in RR.^947 , 969^
– In controlled asthma, cessation of effort is generally due to peripheral fatigue, although a certain degree of expiratory flow limitation may also occur. The ventilatory reserve usually is not exhausted and maximal flow is not reached, even at maximal exertion.^969 , 970^
– In severe asthma, significant ventilatory restriction and impaired CRF occur in ≈30% of patients. Patients with FEV_1_<80% have a lower ventilatory reserve. The percent drop in FEV_1_correlates with increased VE/VO_2_and VE/VCO_2_values.^967 , 971 , 972^
– Most patients do not experience clinically significant hypoxemia or hypercapnia.
– Increased ventilation/perfusion mismatch, alveolar-arterial oxygen tension, and physiological dead space appear to be associated with the presence of bronchospasm.^947 , 969^
– Patients with severe and poorly reversible airway obstruction may present with mechanical restrictions to ventilation and exercise-induced symptoms that mimic those of COPD.^954 , 969^
– Patients with asthma and/or comorbid EIB generally exhibit expiratory stridor/wheezing, with dyspnea/other symptoms reaching their greatest intensity between 3 and 15 minutes after cessation of exertion. Therefore, abnormal changes in lung function are evaluated in spirometry in the post-exercise phase, mainly through FEV_1_.
– CPET is performed as a so-called exercise bronchial challenge test. It is generally performed on a treadmill, as these protocols are more prone to EIA. In the pediatric population, the criterion of a ≥12% reduction in FEV1 (instead of ≥10%) has been preferred due to its greater specificity, with a PPV of 94% and test accuracy of 70%.^966 , 968^

### 7. Sickle Cell Anemia/Sickle Cell Disease

Sickle cell disease (SCD) is a genetic, autosomal recessive hemoglobinopathy resulting from structural defects in hemoglobin (Hb), with or without defects in Hb synthesis. Inherited mutations may be homozygous (SS, a genotype known as sickle cell anemia); simple heterozygous (sickle cell trait), with a normal Hb gene combined with a variant gene; or compound heterozygous, with a variant gene (SC, SD, SE, S beta-thalassemia, S alpha-thalassemia, or S mut) combined with a structural or Hb synthesis defect, generically known as thalassemia. It is estimated that 4% of the Brazilian population has the sickle cell trait and that 25,000-30,000 people have frank sickle cell anemia (SCA) or thalassemia.^973^

In SCD, defective hemoglobin (HbS), when deoxygenated in capillary beds, leads to sickling of red blood cells, causing hemolysis, chronic normocytic anemia, and vaso-occlusive crises with associated ischemia. SCD carries a high morbidity and mortality rate, with potentially lethal acute events including vaso-occlusive crises (sickle cell crises) with severe pain; ischemic tissue injury and possible damage to all organs (stroke, nephropathy, retinopathy, leg ulcers, priapism, avascular necrosis, etc.); and acute chest syndrome (ACS), whose main causes in adults are fat embolism, pulmonary infection, asthmatic crisis, infarction of the thoracic bone structure and in situ thrombosis/pulmonary artery embolism and which usually precedes lethal outcomes.^974^

In children, persistent chronic hypoxemia with SpO_2_<94% is commonly observed. When properly diagnosed and treated, nearly all children with SCA survive to adulthood, though with a reduced life expectancy (≈20 years).

SCD patients usually present with exercise intolerance and reduced cardiorespiratory fitness due to:^168 , 975^

– Low levels of physical activity due to chronic joint pain.
– Exacerbation of the proinflammatory response as a result of intense exercise.
– Reduced oxygen transport capacity due to low Hb levels.
– Cardiac dysfunction resulting from chronic anemia.
– Pulmonary parenchymal dysfunction caused by repeated episodes of acute chest syndrome.
– Pulmonary vascular disease and PAH.
– Peripheral vascular disease/myopathy due to frequent, repeated microvascular occlusions.

SCD can lead to restrictive cardiomyopathy (RCM), characterized by LV diastolic dysfunction with normal systolic function and left atrial enlargement. This combination results in mild secondary PAH, increased velocity of the tricuspid valve regurgitant jet, and increased mortality. Ischemic lesions of the conduction system, fibrosis, and extensive enlargement of all chambers of the heart are potential etiologies of arrhythmia and SCD in CMR.^976 , 977^

Indications for ET/CPET in children and adolescents with SCD:

– CPET allows assessment of CRF, possible limitations to exercise, and prescription of physical exercise, including cardiopulmonary rehabilitation.^978 , 979^
– Lung function tests (including FEV_1_and FEV_1_/FVC ratio) should be performed every 1 to 3 years due to the high prevalence of restrictive (≈26% of patients), obstructive (≈35-39%), pulmonary dysfunction and airway hyperreactivity (70%). Shorter intervals between tests should be adopted especially for patients with persistent dyspnea, history of asthma, and/or recurrent wheezing or marked elevations in hemolytic markers.^980 , 981^
– Acute painful episodes during ET/CPET are rare, occurring in 0.43 to 1% of patients.^979^
– Transient ischemic changes and desaturations during ET/CPET are common, but do not result in arrhythmias or other complications.^979^Generally, half of patients present with exercise-induced ST-segment depression, of whom 31% have a definitive ischemic response (CAD).
– Patients with anemia generally have elevated HR and VE/VCO_2_, abnormal oxygen pulse, and reductions in VO_2_at VT1 and at peak effort.
– ⅔ of patients who develop pulmonary vascular disease have exercise limitation with abnormalities in gas exchange: alveolar-arterial oxygen tension [PAO_2_] >30 mmHg, abnormal dead space to tidal volume ratio (VD/VT), and very high VE/VCO_2_.
– A study showed a 0.3% decline in predicted FEV_1_with each year, regardless of sex, presence of asthma, hemoglobin concentration, incidence of severe acute pain, episodes of acute chest syndrome, and hydroxyurea therapy.^982^
– Children with ACS generally have lower total lung capacity (TLC) and reduced FEV_1_. Age and male sex are associated with lower FEV_1_values and a lower FEV_1_/FVC ratio.^981^
– HR recovery is generally slow and occurs in the 1^st^to 5^th^minutes post-exercise, regardless of ACR. This slow recovery of HR suggests impairment of vagal activity, which worsens with increasing age.^983^
– Patients with Hb-SS have a lower mean oxygen saturation, FVC, and %FEV_1_. According to one study, these, as well as abnormal spirometry results (found in 70.4% of patients), are due to predominantly restrictive defects.^984^
– Pulse oximetry generally underestimates arterial oxygen saturation, but the difference is clinically insignificant. This phenomenon occurs partly due to elevated carboxyhemoglobin (COHb) and methemoglobin (MetHb) levels in SCD. Non-invasive pulse co-oximetry can help measure COHb and MetHb levels and improve the accuracy of saturation determination.^985^
– Exercise-induced desaturation is observed in ≈18% of children with thalassemia and in ≈34% of children with sickle cell anemia.^986^

## Part 4 – Exercise Testing Combined with Cardiac Imaging Methods

### 1. Cardiovascular Stress Combined with Cardiac Imaging Methods

#### 1.1. Nuclear Imaging/Myocardial Perfusion Imaging

In the pediatric population, nuclear cardiology allows assessment of myocardial perfusion and viability, ventricular function, and pulmonary perfusion, as well as detection of inflammatory processes.^214 , 987^

The utility of myocardial perfusion scans is limited due to ionizing radiation exposure and its potential lifelong impact, particularly in patients with CHD. Cancer risk is increased, due to the inherent radiosensitivity of children.^988^

Technological progress in the last decade and the development of low-dose irradiation protocols specific for use in children open new perspectives for the use of nuclear imaging in pediatrics.^214^

The use of cardiac magnetic resonance (CMR) with myocardial perfusion imaging in the pediatric population is increasingly popular. CMR is considered the method of choice for quantifying ventricular volumes and function, especially of the RV. Myocardial viability and ischemia can also be assessed by PET/CT.^988 , 989^

The patient's history and image acquisition planning are essential to ensure that scans are feasible and hold diagnostic value. Details of cardiac anatomy and previous surgical and percutaneous procedures help distinguish normal from pathological findings. The radionuclide dose is based on the child's weight and the image acquisition protocol/methods. Preferably, stress imaging should be performed first. The use of state-of-the-art SPECT, PET, or hybrid imaging cameras is recommended.^987 , 990^

In Brazil, physical or pharmacological stress modalities (dipyridamole, adenosine, or dobutamine) are commonly used; both have similar sensitivity and specificity for analysis of perfusion scans. The choice of stress modality depends mainly on the child's age and limitations or contraindications for physical exercise ( Figure 7 ). The key contraindications for each stressor are given in Table 41 . Additional pulse oximetry monitoring is recommended in patients with CHD, particularly in cases of right-to-left shunt and/or pulmonary arteriovenous malformations.^214 , 243 , 991 , 992^

**Figure 7:** Selection of cardiovascular imaging method and stress protocol for pediatric patients with congenital heart disease and suspected ischemia.^987^Examples of causes of dynamic obstruction: anomalous coronary arteries, stent compression, myocardial bridging, vasospasm. Examples of causes of fixed obstruction: atherosclerotic coronary obstruction, surgical narrowing of the coronary ostia, thickening of the intima. Examples of causes of microvascular dysfunction: surgical manipulation of the coronary arteries in arterial switch surgery, familial hypercholesterolemia, systemic lupus erythematosus. Stress echo: stress echocardiography; CMR: cardiac magnetic resonance; PET: positron emission tomography; SPECT: single photon emission computed tomography; N-13:^13^N ammonia; Rb-82:^82^rubidium; Tc-99m:^99m^technetium; Gd: gadolinium. *Cardiovascular imaging methods presented sequentially according to the choice of stressor. **Available in Brazil only for research purposes. ***Not currently available in Brazil.

**Table 41:** Contraindications for cardiovascular stress modalities in the pediatric population^214 , 243 , 991 , 992^

Stressor	Contraindications
Physical stress (ET)	See contraindications for ET/CPET - Chart 2.
Vasodilators (Dipyridamole / Adenosine)	High-grade AV blocks; hypotension; marked hypertension; sinus bradycardia; bronchoconstrictive disease or active bronchospastic disease with regular use of inhalers; known hypersensitivity to vasodilators.
Dobutamine	Severe hypertension; unstable angina; severe aortic valve stenosis; complex arrhythmias; obstructive hypertrophic cardiomyopathy; myocarditis; endocarditis; pericarditis.
Atropine	Narrow-angle glaucoma; myasthenia gravis; obstructive uropathy; gastrointestinal disorders.

Physical stress methods for MPI:^993^

– Generally done as adjunct to ET/CPET, to increase the diagnostic and prognostic value of imaging methods by addressing clinical, hemodynamic, and ECG parameters.
– The choice of ergometer and protocol should follow the same criteria used in ET/CPET for children and adolescents as listed elsewhere in this Guideline.

Pharmacological stress methods for MPI:

– The doses of pharmacological stressors (dipyridamole, dobutamine, and adenosine) for children are the same as those used in adults. Stress imaging should follow the general guidelines for adults, adjusted as follows:^1^
– Throughout the test, clinical signs and symptoms should be monitored and ECG, BP, and HR recorded continuously, regardless of the stressor employed.
– Adenosine is a pharmacological stressor that causes coronary vasodilation when administered intravenously (continuous infusion, 140 *μ* g.kg^-1^.min^-1^, over 4 to 6 min). Its side effects are generally mild and resolve quickly once the infusion is stopped or completed: bronchospasm, due to activation of A2B and A3 receptors; atrioventricular block, due to activation of A1 receptors; peripheral vasodilation, due to activation of A2B receptors; flushing, dyspnea, and nausea.^990 , 994 , 995^Caffeine (methylxanthine) contained in foods, beverages, and drugs interferes with adenosine (see Appendix 5 ), and must be withheld for at least 12 hours before the scan.^995^
– Dipyridamole is a coronary vasodilator that acts by inhibiting the enzyme adenosine deaminase, which degrades endogenous adenosine, in addition to blocking adenosine reuptake by the cell membrane and consequently increasing the extracellular adenosine concentration, which leads to coronary and systemic vasodilation. The recommended dose for MPI is 0.56 mg.kg^-1^, up to a maximum dose of 60 mg, administered intravenously over the course of 4 minutes, diluted in 50 mL of saline solution. Dipyridamole can be injected manually, without an infusion pump. Its biological half-life is ≈45 minutes. The main side effects are chest pain, headache, and dizziness, which can be reversed by administration of intravenous aminophylline, given just 2 minutes after injection of the radiotracer.^993 , 995 – 998^Methylxanthines (see Appendix 5 ) must be withheld at least 24 hours before the scan.^995^
– Dobutamine promotes increased myocardial oxygen consumption. It is administered intravenously via an infusion pump at an initial dose of 5-10 *μ* g.kg^-1^.min^-1^over 3 minutes, followed by incremental doses of 20 *μ* g.kg^-1^.min^-1^and 30 *μ* g.kg^-1^.min^-1^up to a maximum of 40 *μ* g.kg^-1^.min^-1^.^999 . 1000^In patients who do not reach submaximal HR and have no evidence of ischemia, intravenous atropine can be added at a dose of 0.01 mg.kg^-1^(maximum dose 0.25 mg).^996^The radiotracer should be injected at Target HR (generally defined as 85% of HRmax for age), and dobutamine infusion continued for 1 minute thereafter. For reversal of adverse effects, short-acting beta-blockers (i.e. metoprolol or esmolol) can be injected intravenously after the first minute of radiotracer administration.^1001^
– Consider the need for restriction of the volume to be infused in patients with HF, cardiomyopathies, complex CHDs, and renal failure.
– In addition to the qualified doctor responsible for the examination, it is suggested to be monitored by a pediatrician.

Particular aspects of myocardial perfusion imaging:

1. **Transposition of the great arteries:** early and late mortality are associated with coronary complications.^1002 , 1003^At postoperative follow-up, the indication for reintervention is based more on the presence of ischemia on myocardial perfusion scans than on angiographic findings.^220 , 1004^
  Perfusion defects diagnosed by MPI occur in 5 to 24% of patients after surgical correction, and may persist for more than 10 years ( Figure 7 ). After correction, angiographic lesions are not always associated with a progressive stenotic process.^1005 , 1006^
  Initial SPECT allows patients to be screened for progression: if normal, ischemia will usually stabilize or resolve over time; if abnormal, it generally portends worsening ischemia.^22 , 1007 , 1008^
2. **Kawasaki disease (KD):** MPI is useful and safe in monitoring the progression of coronary stenosis. SPECT has 90% sensitivity and 85-100% specificity for detecting ischemia.^218 , 998^About 12 to 19% of children with coronary aneurysms have an abnormal perfusion pattern (fibrosis and/or ischemia).^216 , 219 , 398^
  MPI is indicated for late follow-up (every 1 to 5 years) of children with coronary aneurysms (including small and/or resolved aneurysms) and/or ventricular symptoms/dysfunction ( Figure 7 ).^18 , 37 , 214^
  In adolescents with a history of KD in childhood, positron emission tomography (PET) with (^13^N)-ammonia demonstrated a decrease in coronary reserve due to long-term endothelial dysfunction.^1009 , 1010^
3. **Cardiomyopathies:**
  – In HCM, SPECT is useful for investigation of ischemia, risk stratification, and optimization of therapy.^223^Myocardial ischemia may be related to reduced subendocardial perfusion in hypertrophied segments, compression of small intramural vessels, and myocardial bridging.^214 , 1011^Microvascular ischemia is believed to be implicated in systolic and diastolic dysfunction.^223 , 1012^Myocardial perfusion defects on^99m^Tc-MIBI SPECT may reflect an ischemic process, and are an important predictor of adverse clinical events and death.^223^
  – In dilated cardiomyopathy, SPECT is rarely used, as ischemic etiology is rare in children. In exceptional circumstances, such as sickle cell anemia, assessment of microvascular function can help identify the potential mechanism of ventricular damage (LV enlargement and/or dysfunction).^214 , 1013^
4. **Heart transplantation:** the main long-term complication of heart transplantation is graft vascular disease (GVD), a relevant cause of death and retransplantation. In GVD, MPI allows assessment of coronary artery involvement (distal and proximal) in systolic dysfunction and increased LV filling pressures.^224 , 1014^

#### 1.2. Stress Echocardiography

Stress echocardiography is a cardiovascular imaging technique that provides real-time images of the heart, allowing assessment of cardiac anatomy, systolic and diastolic function, regional myocardial ischemia, and coronary reserve, as well as risk stratification in valvular heart disease, HF, and CHD (repaired or unrepaired). Key indications for stress echo in pediatric cardiology are given in Table 12 .

Main advantages: available in Brazil; can be performed without sedation in most patients; does not expose the patient to ionizing radiation (a relevant concern in periodic follow-up of patients with CHD). Main limitations: inadequate acoustic windows in children with failure to thrive secondary to CHD or post-surgical changes in chest anatomy; complex cardiac arrhythmias (i.e. VT, complete heart block, etc.); patient may need medications that can affect scan parameters (beta-blockers, diuretics, antiarrhythmics, etc.). Table 41 describes the main contraindications for the various cardiovascular stressors used in stress echocardiography.

Proper acquisition and interpretation of echocardiographic images requires evaluation of the patient for existing heart disease (especially CHD), clinical condition at the time of the scan, past surgical history, and pacemaker/ICD placement.

The main stressors used in the pediatric population are physical (ET) and pharmacological. Pharmacological stress with dobutamine is more commonly used in younger children, while physical exercise is preferred in children over age 8 years who are cooperative and capable of exercising on a treadmill or stationary bicycle ( Table 42 ). Throughout the test, clinical signs and symptoms should be monitored and ECG, BP, and HR recorded continuously, regardless of the stressor employed.^234 , 235^

**Table 42:** Advantages and disadvantages of different cardiovascular stress modalities in the pediatric population^234 , 235^

	Exercise *	Dobutamine
Recommended age	≥8 years	Any age
Anesthesia/sedation	No	Only if indispensable in children aged <6 years
Heart rate response	Generally submaximal	Target heart rate (generally submaximal)
Blood pressure response	Maximal	Variable
Maximal inotropism	Yes	Yes
Venous return	Increase	No increase/decrease
Image acquisition	Artifacts possible **	Easier
Allows assessment of cardiorespiratory fitness	Yes	No
Allows assessment of functional impact	Yes	No
Risk of complications	Low	Low
Availability	Moderate	High

* Treadmill, tabletop cycle ergometer or conventional cycle ergometer (stationary bicycle).

** Respiratory and movement artifacts.

Findings which indicate test cessation: onset of symptoms (i.e. limiting angina); emergence or worsening of regional wall motion abnormalities; ST segment depression ≥2mm; drop in SBP >15mmHg; complex arrhythmia and/or any arrhythmia with hemodynamic instability; target HR achieved; maximum dose of pharmacologic stressor reached; adverse events.

##### 1.2.1. Pharmacologic Stress Methods

###### 1.2.1.1. Dobutamine^229 , 234 , 1015 , 1016^

Dobutamine is the most widely used pharmacologic stressor in the pediatric population. It has a positive inotropic and chronotropic effect, increasing myocardial O_2_demand. When this demand is not met, myocardial ischemia and regional wall motion abnormalities arise. In contrast to physical stress, dobutamine does not lead to increases in venous return and preload, generating greater changes in LV end-diastolic dimensions. This allows slower HR recovery and a longer image acquisition time.^243^

In children aged <8 years, a dobutamine stress echo may require general anesthesia or deep sedation. Images are acquired at rest and after each increase in stressor dose.

Dobutamine stress echo protocols for children are similar to those used in adults. Generally, the dobutamine infusion starts at 5 *μ* g.kg^-1^.min^-1^and is increased at 3-5 minute intervals to 10, 20, 30, 40, and 50 *μ* g.kg^-1^.min^-1^. The target HR is generally defined as 85% of HRmax for age. If the target HR is not achieved with the maximum dose of dobutamine, atropine 0.01 mg.kg^-1^can be administered simultaneously every 1-2 minutes (limits: 0.25 mg per dose; maximum cumulative dose 1-2 mg).^243^When evaluating cardiac contractile reserve, dobutamine can be administered in low to moderate doses (5-20 *μ* g.kg^-1^.min^-1^) as a continuous infusion.^229^

Side effects include palpitations, nausea, headache, chills, urinary urgency, anxiety, angina, hypotension, hypertension, and arrhythmia. As dobutamine has a short half-life, these generally resolve upon termination/suspension of the infusion. Esmolol (0.5 mg.kg^-1^) should be available to reverse more severe adverse reactions and/or ischemia.^249^

###### 1.2.1.2. Vasodilators^229 , 234 , 1016^

Stress echo with a vasodilator (adenosine or dipyridamole) induces an increase in coronary flow and is used to evaluate myocardial motility, ischemia, and myocardial viability. Adenosine is infused at a maximum dose of 140 *μ* g.kg^-1^.min^-1^with simultaneous imaging over 4 minutes.

Dipyridamole is administered in two stages, also with continuous imaging: the first stage at a dose of 0.56 mg.kg^-1^over 4 min; the second stage, carried out only if there are no adverse effects, at a dose of 0.28 mg.kg^-1^over 2 minutes. Aminophylline must be available to reverse adverse reactions to dipyridamole.

##### 1.2.2. Exercise Stress Methods^1015 , 1017^

Physical stress echocardiography can be done in children ≥8 years old who are capable of completing an ET. Physical exertion is a physiological stressor, and should be the preferred method whenever possible.^229^Physical stress increases HR, contractile function, BP, and venous return to the heart, and determines VO_2_and cardiac output.

The most commonly used ergometers for stress echo are the treadmill and cycle ergometer (vertical, supine, and semi-supine), with specific protocols. Baseline echocardiography should be obtained in the supine position and in the position in which physical stress will be performed. When using a treadmill, echocardiographic image acquisition is performed before the start of exercise and immediately (within 60-90 s) after the end of the test. When using a cycle ergometer, images are acquired before and during all phases of exercise (including peak exertion). Imaging during exertion is more challenging, due to movement and breathing artifacts.

Furthermore, as HR in children can drop very rapidly during recovery, interpretation of results may be compromised. The cycle ergometer is a more suitable method to obtain information during exercise.

In addition to the findings indicating test cessation listed at the beginning of this section, clinicians are advised to adhere to the test cessation criteria contained in Table 32 as well.

## APPENDICES

### Appendix 1 Core legal and regulatory framework applicable to ET and CPET in children and adolescents in Brazil

**Table:**

Legal aspects – translation realized from original in Brazilian portuguese.	Reference
– The physician shall preserve the confidentiality of any information acquired in the performance of his or her duties, except when legally mandated otherwise. – The physician is forbidden from: – Delegating to other providers acts or duties restricted to the medical profession. – Shirking responsibility for a medical procedure he or she indicated or in which he or she participated, even when the patient was assisted by several physicians. – Aiding and abetting those who practice medicine illegally or with medical professionals or facilities which engage in illicit activities. – Failing to obtain consent from the patient or his or her legal representative after explaining the procedure to be performed, except in case of imminent risk of death. – Failing to safeguard the patient's right to decide freely about his or her person or well-being, or utilizing his or her authority to violate this right. – Failing to keep a legible medical record for each patient. – Breaching physician–patient confidentiality relative to a child or adolescent patient, as long as the patient has legal capacity to discern, including to their parents or legal guardians, except when nondisclosure could cause harm to the patient. – Failing to obtain from the patient or their legal guardian a written informed consent form before carrying out any research involving human beings, after the nature and consequences of the research have been duly explained. § 1 In the event that the research participant is a child, adolescent, person with a mental disorder or illness, or otherwise in a situation of diminished capacity, in addition to the consent of their legal guardian, the participant's free and informed assent to the fullest extent of their understanding is required.	Brazilian Code of Medical Ethics ( *Código de Ética Médica* ), CFM Resolutions No. 2217/2018, 2222/2018, and 2226/2019.^1018 – 1020^
Sets forth the requirements for Focused Practice Designation in Exercise Testing: 1 (one) year of training; having completed Medical Residency in Cardiology before such training; after training, take the Brazilian Medical Association/Brazilian Society of Cardiology board exam to obtain certification; as a prerequisite for sitting the aforementioned exam, in addition to training, holding a current Board Certification in Cardiology from the Brazilian Medical Association.	CFM Resolution No. 2,380/2024; Ordinance No. 1/2024.^1021^
Whereas, it is advisable that written informed consent be obtained from the patient or his/her legal guardian (for patients under 18 years of age); Whereas, in the case of underage patients, a legal guardian must remain in the examination room; The ET must be individualized and carried out, at all stages, by a qualified physician who has been trained to respond to cardiovascular emergencies, and must thus be physically present in the room at all times. As ET is a medical procedure under the sole responsibility of the performing physician, delegating its performance to other providers is considered a violation of medical ethics. The necessary and appropriate conditions for carrying out ET are listed in the CFM Inspection Manual.	CFM Resolution No. 2021/13.^1022^
Guiding criteria for advertising in medicine, conceptualizing advertisements, dissemination of medical matters, sensationalism, self-promotion, and prohibitions related thereto.	CFM Resolution No. 2,336/2023.^1023^
Ensuring the privacy and confidentiality of patients’ data and digitally stored information; organizing secure and reliable databases; ensuring the secure transmission of data and information; maintaining backup copies to the fullest possible extent.	CFM Resolution No. 1821/2007.^272^
Art. 226. The family, which is the foundation of society, shall enjoy special protection from the State. Paragraph 4. The community formed by either parent and their descendants is also considered as a family entity. Art. 229. Parents have the responsibility to assist, up bring and educate their underage children, and adult children have the responsibility to help and assist their parents in old age, need, or sickness.	Constitution of the Federative Republic of Brazil.^1024^
Art. 5. A Legal minority ends at the age of eighteen, at which point a person is entitled to perform all acts of civil life. Sole paragraph. Before said age, legal incapacity can end: upon emancipation granted by the minor's parents, or by one parent in the absence of the other, by means of a public instrument, regardless of judicial approval, or by a sentence of emancipation issued by a judge, having heard the legal guardian, provided the minor is sixteen years of age; through marriage; through the discharge of one's duties as a public servant; upon graduation from an institution of higher learning; upon incorporation of a civil or commercial enterprise, or through the establishment of an employment relationship, provided that the minor is sixteen years of age and, as a result of either, achieves financial independence." Art. 186. Anyone who, by willful action or inaction, negligence, or recklessness, violates a right and causes damage to others, even if exclusively moral, commits a wrongful act.	Brazilian Civil Code (Law No. 10,406/2002).^1025^
Chapter III, Art. 6 – The following are basic consumer rights: the protection of the consumer's life, health, and safety against any risks arising from any practices in the supply of products and services considered harmful or dangerous; education and information about the adequate consumption of products and services, ensuring freedom of choice and equality in transactions; adequate and clear information about different products and services, with correct specification of quantity, characteristics, composition, quality, price, and taxes, as well as the risks presented.	Brazilian Consumer Protection Code. Basic Consumer Rights (Law No. 8,078 of September 11, 1990).^1026 , 1027^

CFM: Brazilian Federal Medical Council (CFM from portuguese: Conselho Federal de Medicina).

### Appendix 4 Markers of cardiorespiratory fitness (predicted VO2max) and OUES in an apparently healthy pediatric population with heart disease

**Table:**

Material	Age	Location	DOI	Available at
**Apparently healthy:**				
VO_2_max percentiles for sex and age.^176^	8-18	Figure 2	10.1513/AnnalsATS.201611-912FR	https://www.atsjournals.org/doi/10.1513/AnnalsATS.201611-912FR
VO_2_max percentiles for sex and age.^1030^	12-18	Figure 2 and Figure 3	10.1016/j.amepre.2011.07.005	https://linkinghub.elsevier.com/retrieve/pii/S0749-3797(11)00491-0
VO_2_max values for sex and age group in the Brazilian population.^1031^	7-12 and 13-19	Table 6	10.5935/2359-4802.20190057	https://www.scielo.br/j/ijcs/a/x8bB3qQHQKCXHRbZRbpXMrm/?lang=en
DP at rest and DPpeak at moderately high altitude.^1030^	4-18	Table 3	10.1016/j.acmx.2013.04.003	https://www.elsevier.es/es-revista-archivos-cardiologia-mexico-293-articulo-cardiopulmonary-exercise-testing-in-healthy-S1405994013000621
OUES percentile chart (for sex and age) and OUES prediction equations.^622^	8-19	Figure 2 and Table 2	10.1177/2047487315611769	https://academic.oup.com/eurjpc/article-lookup/doi/10.1177/2047487315611769
Graph of average OUES behavior by sex and age.^614^	7-18	Figure 1	10.1123/pes.22.3.431	https://journals.humankinetics.com/doi/10.1123/pes.22.3.431
**In heart disease:**				
Charts and tables, stratified by sex, of VO_2_max/VO_2_peak and %VO_2_predicted in patients with univentricular hearts, tetralogy of Fallot, transposition of the great arteries, and other heart diseases.^1032^	6-18	Table 1 , Table 2 , Figure 2 .	10.1007/s00431-022-04648-9	https://www.ncbi.nlm.nih.gov/pmc/articles/PMC9829639/
Charts and tables of association between VO_2_max and HRmax in children and adolescents with CHD.^80^	6-18	Table 1 , Figure 2 , Figure 4 .	10.5935/abc.20170125	https://www.ncbi.nlm.nih.gov/pmc/articles/pmid/28876372/
Charts and tables of VO_2_peak for healthy children and adolescents and those with CHD.^1033^	8-16	Table 7 , Figure 3 , Figure 4 .	10.1007/s004210050612	https://link.springer.com/article/10.1007/s004210050612
Graph and equation predicting DP values in the first two decades of life and in comparison with patients with repaired aortic coarctation.^428^	12.6±2.96 and 13.0±3.2 years	Table 2 and Figure 3	10.1080/14779072.2017.1385392	https://www.tandfonline.com/doi/epdf/10.1080/14779072.2017.1385392?needAccess=true
Table with DP behavior (rest and peak effort) in relation to the survival of children with heart failure secondary to idiopathic dilated cardiomyopathy.^1033^	8.6±1.9 years	Table 2 and Table 3	10.1016/j.ejheart.2008.04.009	https://onlinelibrary.wiley.com/doi/epdf/10.1016/j.ejheart.2008.04.009
Charts and tables of OUES behavior by sex and corrected for weight in the apparently healthy pediatric population and in 10 congenital heart diseases.^624^	5-18	Table 2 , Figure 2 , Figure 3 .	10.1136/archdischild-2019-317724	https://adc.bmj.com/lookup/pmidlookup?view=long&pmid=32732318
Reference values for OUES/kg by age, stratified by normal vs. abnormal functional capacity, in children and adolescents with and without CHD.^623^	4-21	Table 5 and Table 6 .	10.1177/2047487318807977	https://academic.oup.com/eurjpc/article-lookup/doi/10.1177/2047487318807977

VO_2_max: maximum oxygen consumption; DP: double-product; DPpeak: double-product at peak effort; OUES: oxygen uptake efficiency slope; HRmax: maximum heart rate; CHD: congenital heart disease.

### Appendix 5 Key caffeine-containing beverages, foods, and medications

**Table:**

**Coffees**
Coffee Espresso Mocha Decaffeinated coffee
**Teas, general**
Black tea Iced tea Green tea Lemon iced tea (bottled) Lipton Decaffeinated Tea (black or green)
**Soft drinks and juices**
Pepsi Coca-Cola, Coca Zero, Diet Pepsi Coca-Cola Plus Diet Coke Fanta, Sprite, 7-Up Guaraná Acerola juice
**Energy drinks**
Monster Energy Red Bull Fusion TNT
**Caffeinated snack foods**
Chocolate cookies Some potato chips Some candies and gums
**Ice cream**
Starbucks coffee ice cream Coffee ice cream Häagen-Dazs coffee ice cream
**Cocoa and other beverages**
Hot chocolate Candy bars Milk chocolate bars
**Drugs**
Tylenol DC Ormigrein Metamizole/caffeine Neosaldina Miorrelax Miosan Caf Dorflex Benegrip Caffeinated supplements/caffeine pills

Note: The products and trademarks listed above are those most widely available in the Brazilian market. The same information applies to analogous products. Adapted from: Henzlova MJ et al. ASNC imaging guidelines for SPECT nuclear cardiology procedures: Stress, protocols, and tracers.^995^
